# Supplementary material for: Reconstitution of pluripotency from mouse fibroblast through Sall4 overexpression
Source: Nat Commun. 2024 Dec 30;15:10787. doi: 10.1038/s41467-024-54924-5 (PMC11686038; doi:10.1038/s41467-024-54924-5)
Supplement: Supplementary file 1 — Supplementary Information [file 41467_2024_54924_MOESM1_ESM.pdf]

Supplementary Information

Reconstitution of Pluripotency from Mouse Fibroblast through *Sall4* Overexpression

Supplementary information includes:

Supplementary Figure 1-16.

Supplementary Table 1. List of primer sequences for PCR, qRT-PCR and shRNA.

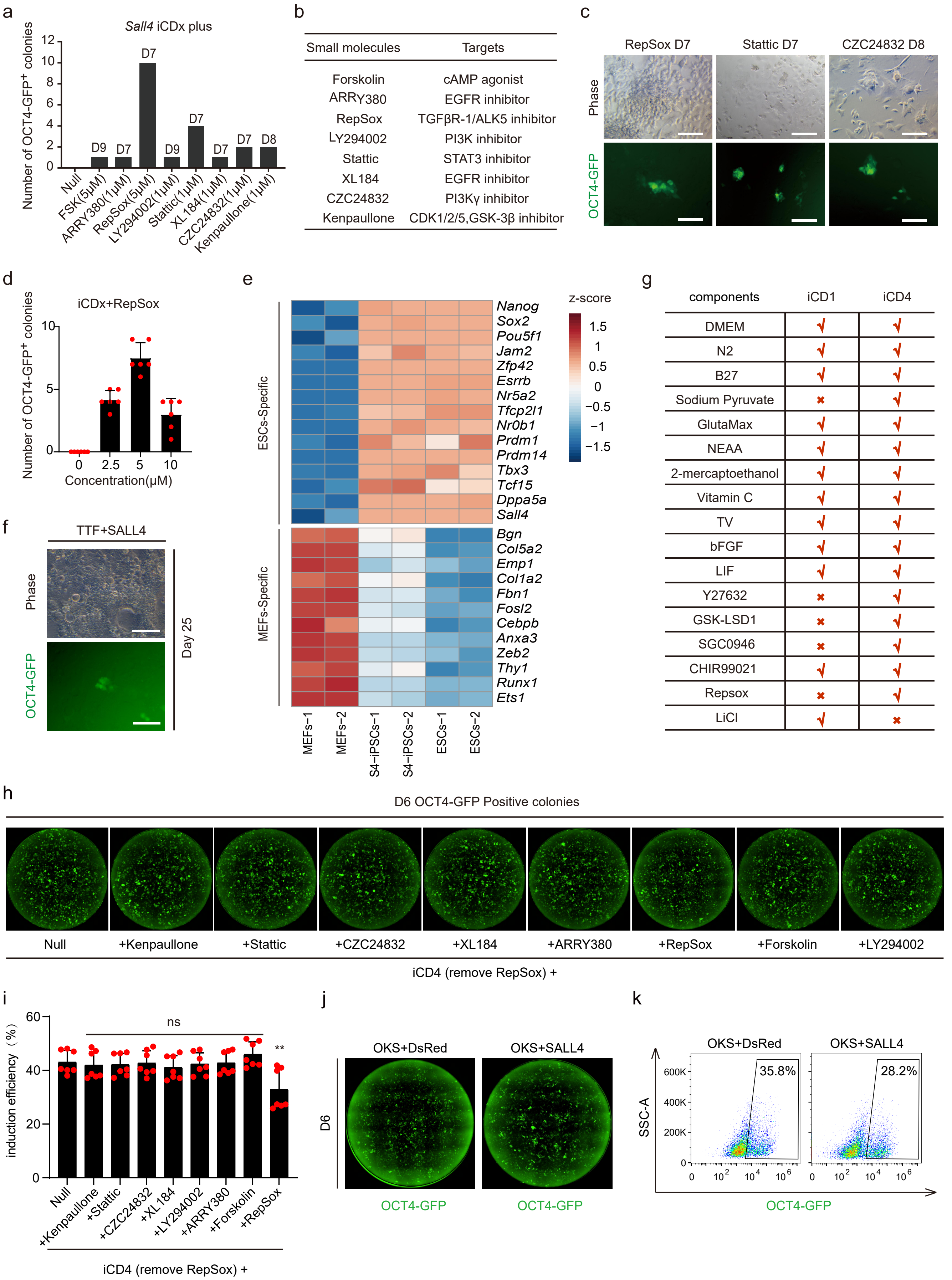
a
Sall4 iCDx plus
Number of OCT4-GFP+ colonies
0
2
4
6
8
10
12
D9
D7
D7
D9
D7
D7
D7
D8
Null
FSK(5μM)
ARRY380(1μM)
RepSox(5μM)
LY294002(1μM)
Stattic(1μM)
XL184(1μM)
CZC24832(1μM)
Kenpaullone(1μM)
b
Small molecules
Targets
Forskolin
cAMP agonist
ARRY380
EGFR inhibitor
RepSox
TGFβR-1/ALK5 inhibitor
LY294002
PI3K inhibitor
Stattic
STAT3 inhibitor
XL184
EGFR inhibitor
CZC24832
PI3Kγ inhibitor
Kenpaullone
CDK1/2/5,GSK-3β inhibitor
c
RepSox D7
Stattic D7
CZC24832 D8
Phase
OCT4-GFP
d
iCDx+RepSox
Number of OCT4-GFP+ colonies
0
5
10
0
2.5
5
10
Concentration(μM)
e
z-score
1.5
1
0.5
0
−0.5
−1
−1.5
ESCs-Specific
MEFs-Specific
Nanog
Sox2
Pou5f1
Jam2
Zfp42
Esrrb
Nr5a2
Tfcp2l1
Nr0b1
Prdm1
Prdm14
Tbx3
Tcf15
Dppa5a
Sall4
Bgn
Col5a2
Emp1
Col1a2
Fbn1
Fosl2
Cebpb
Anxa3
Zeb2
Thy1
Runx1
Ets1
MEFs−1
MEFs−2
S4−iPSCs−1
S4−iPSCs−2
ESCs−1
ESCs−2
f
TTF+SALL4
Phase
OCT4-GFP
Day 25
g
components
iCD1
iCD4
DMEM
N2
B27
Sodium Pyruvate
GlutaMax
NEAA
2-mercaptoethanol
Vitamin C
TV
bFGF
LIF
Y27632
GSK-LSD1
SGC0946
CHIR99021
Repsox
LiCl
h
D6 OCT4-GFP Positive colonies
Null
+Kenpaullone
+Stattic
+CZC24832
+XL184
+ARRY380
+RepSox
+Forskolin
+LY294002
iCD4 (remove RepSox) +
i
induction efficiency（%）
0
20
40
60
ns
**
Null
+Kenpaullone
+Stattic
+CZC24832
+XL184
+LY294002
+ARRY380
+Forskolin
+RepSox
iCD4 (remove RepSox) +
j
OKS+DsRed
OKS+SALL4
D6
OCT4-GFP
OCT4-GFP
k
OKS+DsRed
OKS+SALL4
35.8%
28.2%
SSC-A
600K
400K
200K
0
0
10^2
10^4
10^6
OCT4-GFP

Supplementary Fig.1 Small molecule screening for the determination of induction medium that could driving SALL4 - mediated iPSCs reprogramming successfully

a. The number of OCT4-GFP$^+$ colonies on day 10 from 1.5×10^4 MEFs infected with SALL4 in iCDx plus small molecular compounds. n = 1 well from 1 independent experiments. **b.** Targets of the small molecular compounds that could driving SALL4-iPSCs production. **c.** Morphological diagram of the small molecular induced SALL4-iPSCs. Scale bars, 200μm. The experiments were repeated one times. **d.** The dosage effect of RepSox in driving SALL4-iPSCs reprogramming. Data are mean ± SD; n = 6 well from 3 independent experiments. **e.** Heatmaps for the expression of ESCs specific genes and MEFs specific genes for SALL4-iPSCs, ESCs and MEFs in RNA-seq data. **f.** The morphology of OCT4-GFP$^+$ colonies induced by SALL4 from TTFs. Scale bars, 200μm. The experiments were repeated independently three times with similar results. **g.** The components of iCD1 medium and iCD4 medium. **h.** OCT4-GFP$^+$ colonies collects from the whole wells in 24 well plate shows the OKS-mediated iPSCs induction efficiency using iCD4(remove Repsox) medium added with small molecules in Supplementary Fig.1b. The experiments were repeated independently three times with similar results. **i.** The histogram shows the iPSCs induction efficiency in Supplementary Fig.1h. Data are mean ± SD. n =6 well from 3 independent experiments. **p=0.0093. **j.** OCT4-GFP$^+$ colonies collects from the whole wells in 24 well plate shows the OKS+SALL4-mediated iPSCs induction efficiency using iCD4 medium at day6. The experiments were repeated independently three times with similar results. **k.** Flow cytometry was used to analyze the iPSCs induction efficiency in Supplementary Fig.1j.

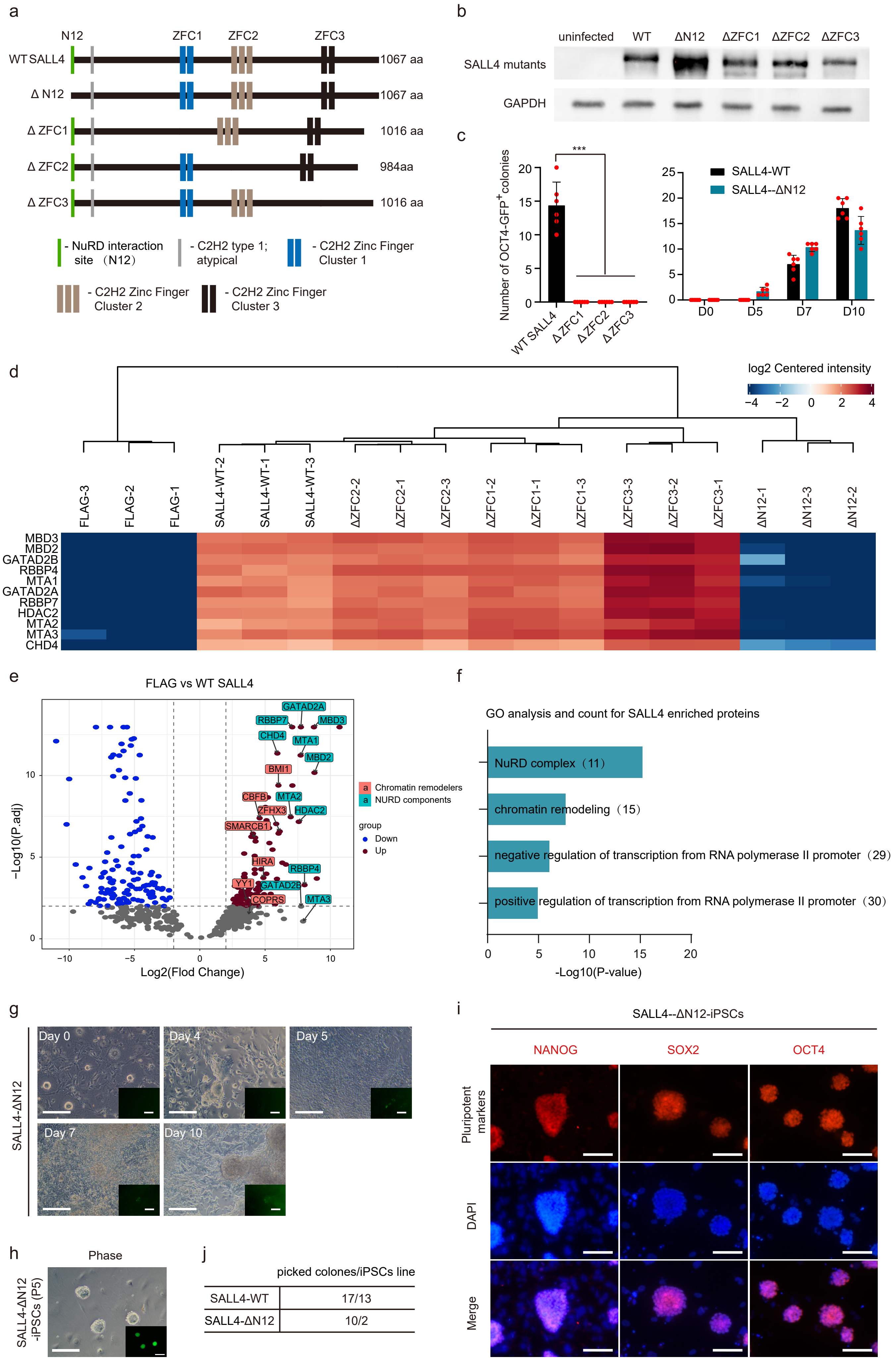

a
N12
ZFC1
ZFC2
ZFC3
WT SALL4
1067 aa
Δ N12
1067 aa
Δ ZFC1
1016 aa
Δ ZFC2
984aa
Δ ZFC3
1016 aa
- NuRD interaction site （N12）
- C2H2 type 1; atypical
- C2H2 Zinc Finger Cluster 1
- C2H2 Zinc Finger Cluster 2
- C2H2 Zinc Finger Cluster 3
b
uninfected
WT
ΔN12
ΔZFC1
ΔZFC2
ΔZFC3
SALL4 mutants
GAPDH
c
Number of OCT4-GFP+colonies
***
WT SALL4
ΔZFC1
ΔZFC2
ΔZFC3
SALL4-WT
SALL4--ΔN12
D0
D5
D7
D10
d
log2 Centered intensity
−4 −2 0 2 4
FLAG-3
FLAG-2
FLAG-1
SALL4-WT-2
SALL4-WT-1
SALL4-WT-3
ΔZFC2-2
ΔZFC2-1
ΔZFC2-3
ΔZFC1-2
ΔZFC1-1
ΔZFC1-3
ΔZFC3-3
ΔZFC3-2
ΔZFC3-1
ΔN12-1
ΔN12-3
ΔN12-2
MBD3
MBD2
GATAD2B
RBBP4
MTA1
GATAD2A
RBBP7
HDAC2
MTA2
MTA3
CHD4
e
FLAG vs WT SALL4
−Log10(P.adj)
Log2(Flod Change)
GATAD2A
RBBP7
MBD3
CHD4
MTA1
MBD2
BMI1
CBFB
MTA2
ZFHX3
HDAC2
SMARCB1
HIRA
RBBP4
YY1
GATAD2B
COPRS
MTA3
Chromatin remodelers
NURD components
group
Down
Up
f
GO analysis and count for SALL4 enriched proteins
NuRD complex（11）
chromatin remodeling（15）
negative regulation of transcription from RNA polymerase II promoter（29）
positive regulation of transcription from RNA polymerase II promoter（30）
-Log10(P-value)
g
SALL4-ΔN12
Day 0
Day 4
Day 5
Day 7
Day 10
h
Phase
SALL4-ΔN12 -iPSCs (P5)
i
SALL4--ΔN12-iPSCs
NANOG
SOX2
OCT4
Pluripotent markers
DAPI
Merge
j
picked colones/iPSCs line
SALL4-WT 17/13
SALL4-ΔN12 10/2

Supplementary Fig.2 The inhibition effect of SALL4-mutants in reprogramming

a. Schematic representation of the protein sequences showing the structure of the wildtype SALL4 and SALL4 mutants. Color codes of ZFC1, ZFC2, ZFC3 and N12 are defined as described in Figure. **b.** Western blot shows the overexpression of SALL4 mutants in MEFs. **c.** The iPSCs induction efficiency at day10 using SALL4 mutants. Wildtype SALL4 as postive control are shown. Data are mean ±SD. n = 6 well from 3 independent experiments. WT SALL4 versus SALL4 mutants, ***p = 0.0001689. **d.** Heatmaps for the level of NuRD complex-associated protein enriched by wild-type SALL4 and SALL4 mutants in reprogramming cells at day 2. **e.** Volcano plots of SALL4-WT enriched proteins of reprogramming samples at day2. IP-MS experiments were performed in triplicates and a two-sided t-test was applied. p.adjust=0.05 and fold change=2 were used as threshold. **f.** GO analysis for SALL4-WT specific enriched proteins. Statistical analysis was performed using Fisher's exact test, the -Log10(p value) for each term are shown. **g.** Morphological diagram for the iPSCs induction process using SALL4-ΔN12. Scale bars, 200μm. The experiments were repeated independently three times with similar results. **h.** The morphology of Passage 5 iPSCs colonies derived from MEFs by overexpressed SALL4-ΔN12 in iCD4. Scale bars, 200μm. The experiments were repeated independently three times with similar results. **i.** Immunofluorescence analysis of pluripotency markers in SALL4-ΔN12-iPSCs. Scale bars, 100μm. The experiments were repeated independently three times with similar results. **j.** The iPSCs colonies formation efficiency of OCT4-GFP$^+$ cells derived from SALL4-ΔN12 or SALL4-WT condition.

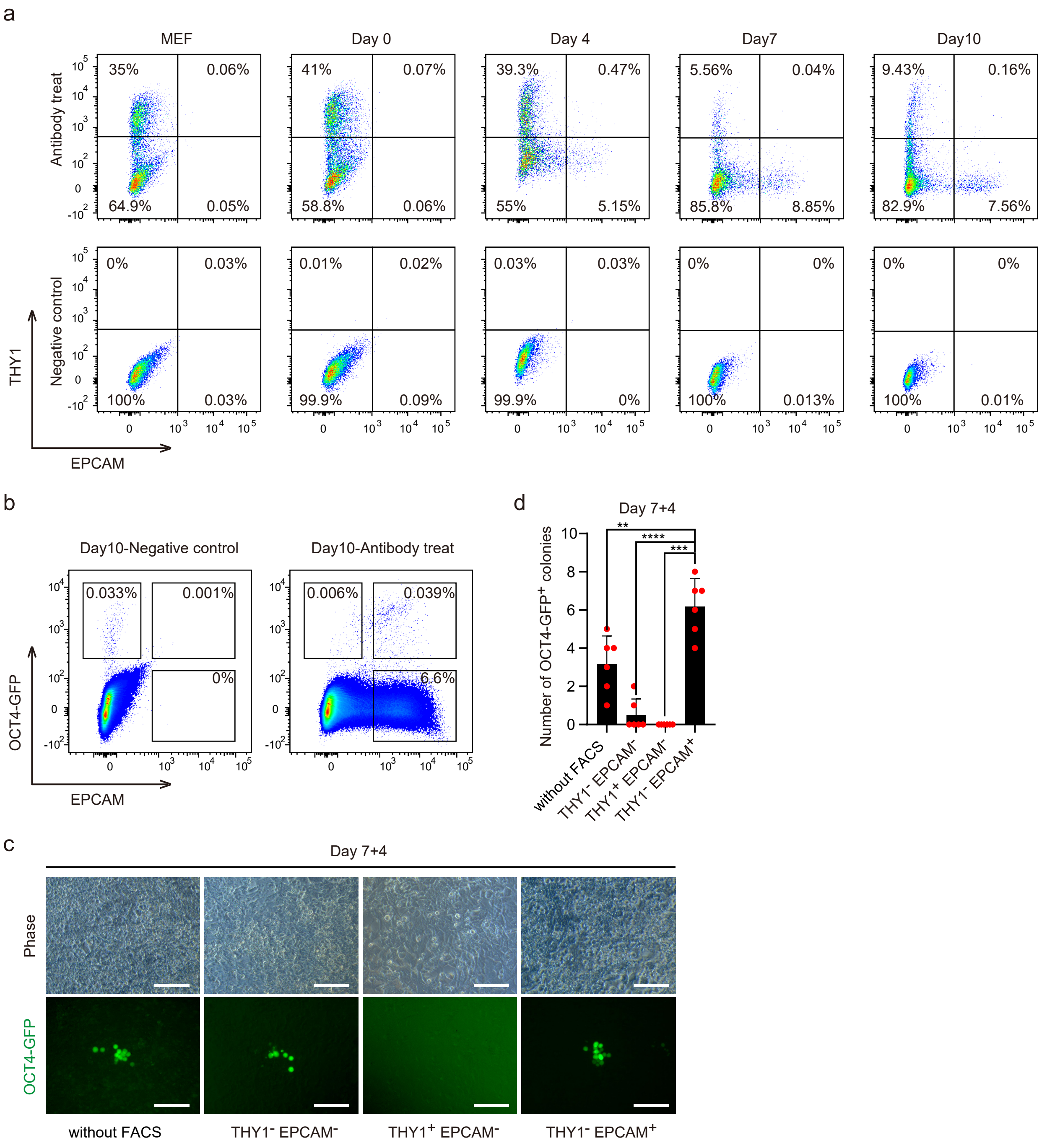
a
MEF
Day 0
Day 4
Day7
Day10
Antibody treat
Negative control
THY1
EPCAM
35%
0.06%
64.9%
0.05%
41%
0.07%
58.8%
0.06%
39.3%
0.47%
55%
5.15%
5.56%
0.04%
85.8%
8.85%
9.43%
0.16%
82.9%
7.56%
0%
0.03%
100%
0.03%
0.01%
0.02%
99.9%
0.09%
0.03%
0.03%
99.9%
0%
0%
0%
100%
0.013%
0%
0%
100%
0.01%
b
Day10-Negative control
Day10-Antibody treat
OCT4-GFP
EPCAM
0.033%
0.001%
0%
0.006%
0.039%
6.6%
d
Day 7+4
Number of OCT4-GFP+ colonies
without FACS
THY1- EPCAM-
THY1+ EPCAM-
THY1- EPCAM+
c
Day 7+4
Phase
OCT4-GFP
without FACS
THY1- EPCAM-
THY1+ EPCAM-
THY1- EPCAM+

Supplementary Fig.3 The Thy1^{-}/Epcam^{+} subgroup in SALL4 system has the potential to generate iPSCs

a. Flow cytometry was used to analyze the proportion of THY1^{-}/EPCAM^{+} subgroup in SALL4 system at day0, day4, day7 and day10, respectively. **b.** FACS analysis of OCT4-GFP^{+}/EPCAM^{+} cells at day10. **c.** Morphological diagram for the iPSCs generation at day4 induced from the day7 THY1^{-}/EPCAM^{+} subgroup in SALL4 system. Scale bars, 100μm. The experiments were repeated independently three times with similar results. **d.** The OCT4-GFP^{+} cells induction efficiency induced from subgroups classified by THY1 and EPCAM. Data are mean ± SD. Statistical analysis was performed using two-tailed, unpaired t test; n = 6 well from 3 independent experiments. THY1^{-}/EPCAM^{+} versus without FACS, **p = 0.0054460; THY1^{-}/EPCAM^{+} versus THY1^{-}/EPCAM^{-}, ****p = 0.0000386; THY1^{-}/EPCAM^{+} versus THY1^{+}/EPCAM^{-}, ***p = 0.0001510.

a

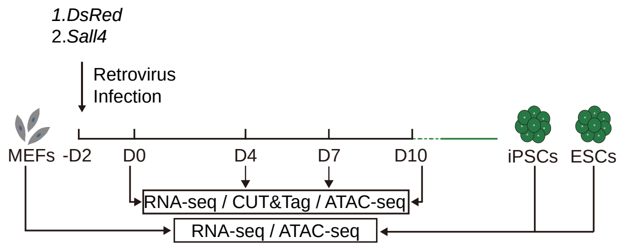

b

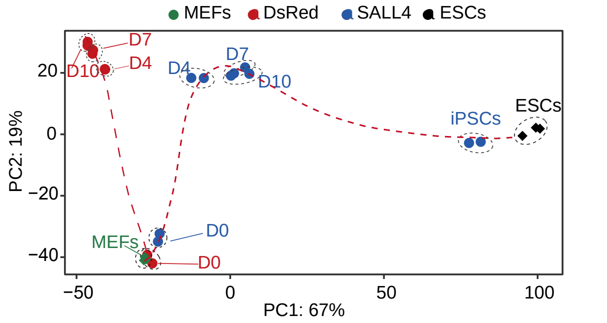

c

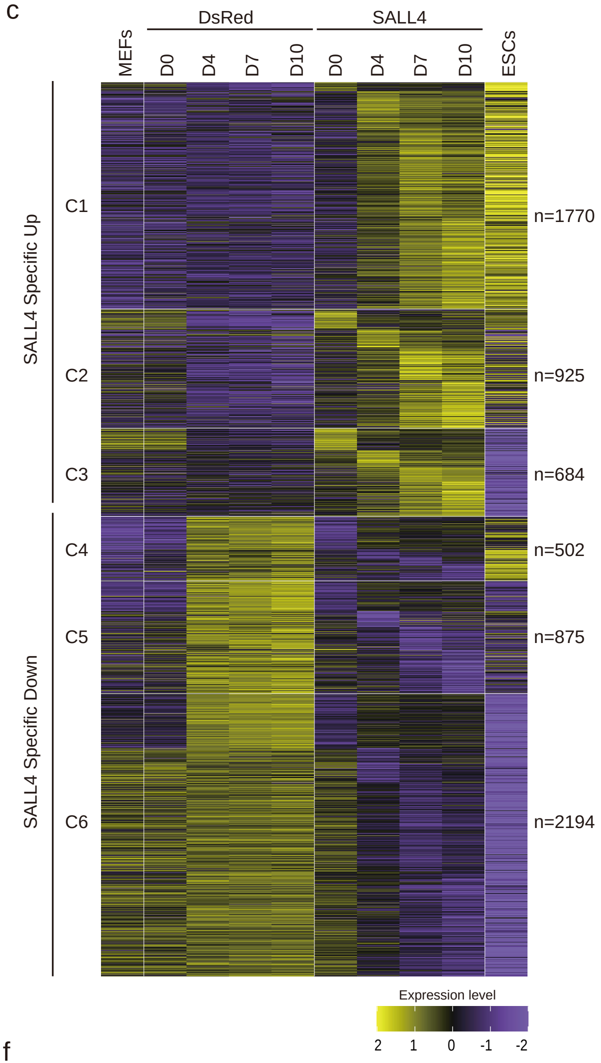

d

Cluster	GO biological process and count	P-value
SALL4 Specific Up (C1)	cell cycle(116)	4.87E-19
	cellular response to DNA damage stimulus(80)	3.81E-10
	DNA duplex unwinding(16)	4.32E-08
	meiotic cell cycle(37)	3.50E-06
	stem cell differentiation(17)	3.10E-05
	**epithelial cell morphogenesis(10)**	6.09E-05
	**stem cell population maintenance(17)**	1.08E-04
	cell proliferation(46)	1.67E-04
	regulation of transcription from RNA polymerase II promoter(155)	4.03E-04
	**spermatogenesis(60)**	5.88E-04
	oogenesis(12)	7.08E-04
	protein phosphorylation(71)	9.77E-04
	male meiosis I(9)	0.001793
	placenta development(12)	0.002252
	male meiosis(9)	0.002715
	regulation of Wnt signaling pathway(7)	0.003250
	histone modification(10)	0.004160
	**embryonic pattern specification(8)**	0.005205

e

Cluster	GO biological process and count	P-value
SALL4 Specific Down (C6)	**inflammatory response(101)**	5.62E-20
	angiogenesis(83)	7.41E-20
	**nervous system development(99)**	1.58E-15
	positive regulation of ERK1 and ERK2 cascade(63)	3.26E-13
	positive regulation of cell migration(67)	7.56E-13
	positive regulation of angiogenesis(48)	1.72E-12
	positive regulation of neuron projection development(51)	4.35E-12
	positive regulation of synapse assembly(30)	1.26E-11
	immune system process(101)	5.23E-11
	negative regulation of cell proliferation(79)	2.30E-08
	ossification(33)	3.90E-08
	learning or memory(25)	3.81E-07
	**lung development(34)**	5.19E-07
	positive regulation of apoptotic process(69)	5.33E-07
	neuron projection development(40)	1.33E-06
	axon guidance(41)	1.37E-06
	neuron migration(36)	1.47E-06
	**heart development(54)**	1.27E-05

f

Cluster	GO biological process and count	-Log10 p-value
SALL4 Specific up (C2)	**cell cycle(79)**	19.524
	cell division(56)	16.582
	positive regulation of chromosome segregation(7)	5.227
	mitotic spindle midzone assembly(6)	5.113
	G2/M transition of mitotic cell cycle(11)	4.989
	mitotic sister chromatid segregation(9)	4.678
	G1/S transition of mitotic cell cycle(12)	4.362
	mitotic spindle organization(10)	4.238
	glycolytic process(9)	4.034
	centrosome cycle(9)	3.877
	chromatin organization(28)	3.835
	mitotic cell cycle phase transition(7)	3.713
	canonical glycolysis(6)	3.530

Cluster	GO biological process and count	-Log10 p-value
SALL4 Specific up (C3)	multicellular organism development(92)	18.959
	cell differentiation(67)	8.777
	intermediate filament organization(16)	8.776
	positive regulation of gene expression(47)	8.770
	cartilage development(18)	8.680
	skeletal system development(19)	7.826
	embryonic limb morphogenesis(14)	7.469
	chondrocyte differentiation(14)	7.138
	cell-cell signaling(15)	7.130
	mesenchymal cell apoptotic process(7)	6.933
	keratinization(15)	6.469
	embryonic hindlimb morphogenesis(10)	6.275
	canonical Wnt signaling pathway(16)	6.228

g

Cluster	GO biological process and count	-Log10 p-value
SALL4 Specific down (C4)	lipid metabolic process(38)	5.723
	defense response to virus(20)	5.631
	**immune system process(31)**	5.617
	cellular response to interferon-beta(10)	5.000
	cellular detoxification of nitrogen compound(5)	4.831
	innate immune response(34)	4.604
	response to bacterium(18)	3.607
	negative regulation of viral genome replication(8)	3.582
	response to drug(19)	3.534
	response to virus(10)	3.493
	nitrobenzene metabolic process(4)	3.467
	negative regulation of NF-kappaB transcription factor activity(9)	3.444
	glutathione metabolic process(8)	3.400

Cluster	GO biological process and count	-Log10 p-value
SALL4 Specific down (C5)	lipid metabolic process(70)	11.934
	**immune system process(42)**	4.977
	transmembrane transport(37)	4.644
	positive regulation of reactive oxygen species metabolic process(9)	4.452
	response to interferon-gamma(8)	3.573
	fatty acid beta-oxidation(9)	3.309
	synaptic vesicle clustering(5)	3.285
	negative regulation of neuron projection development(12)	3.280
	lipid catabolic process(14)	3.153
	negative regulation of cell cycle(9)	3.078
	carbohydrate metabolic process(19)	2.999
	negative regulation of axon regeneration(5)	2.975

Supplementary Fig.4 Transcriptome analysis in SALL4-iPSCs reprogramming process

a. SALL4-overexpressed reprogramming samples and DsRed-overexpressed control samples were collected for RNA-seq, ATAC-seq and Cut&Tag. **b.** PCA analysis for RNA-seq data from SALL4 or DsRed-mediated reprogramming process. RNA-seq data were collected from two independent experiment. **c.** Heatmaps of differential expression gene analysis for RNA-seq data from SALL4 and DsRed systems. The six subgroups (C1-C6) were based on the fold change of gene expression between MEFs and ESCs: C1 (gene counts number (GCN) in ESCs / GCN in MEFs ≥2); C2 (0.5 < GCN in ESCs / GCN in MEFs < 2); C3 (GCN in ESCs / GCN in MEFs ≤0.5); C4 (GCN in ESCs / GCN in MEFs ≤ 2); C5 (0.5 > GCN in ESCs / GCN in MEFs > 2); and C6 (GCN in ESCs / GCN in MEFs ≥ 0.5). **d-g.** GO analysis for genes specifically up-regulated and down-regulated by SALL4 in **c**. Statistical analysis for GO was performed using Fisher's exact test, the -Log10(p value) for each term are shown.

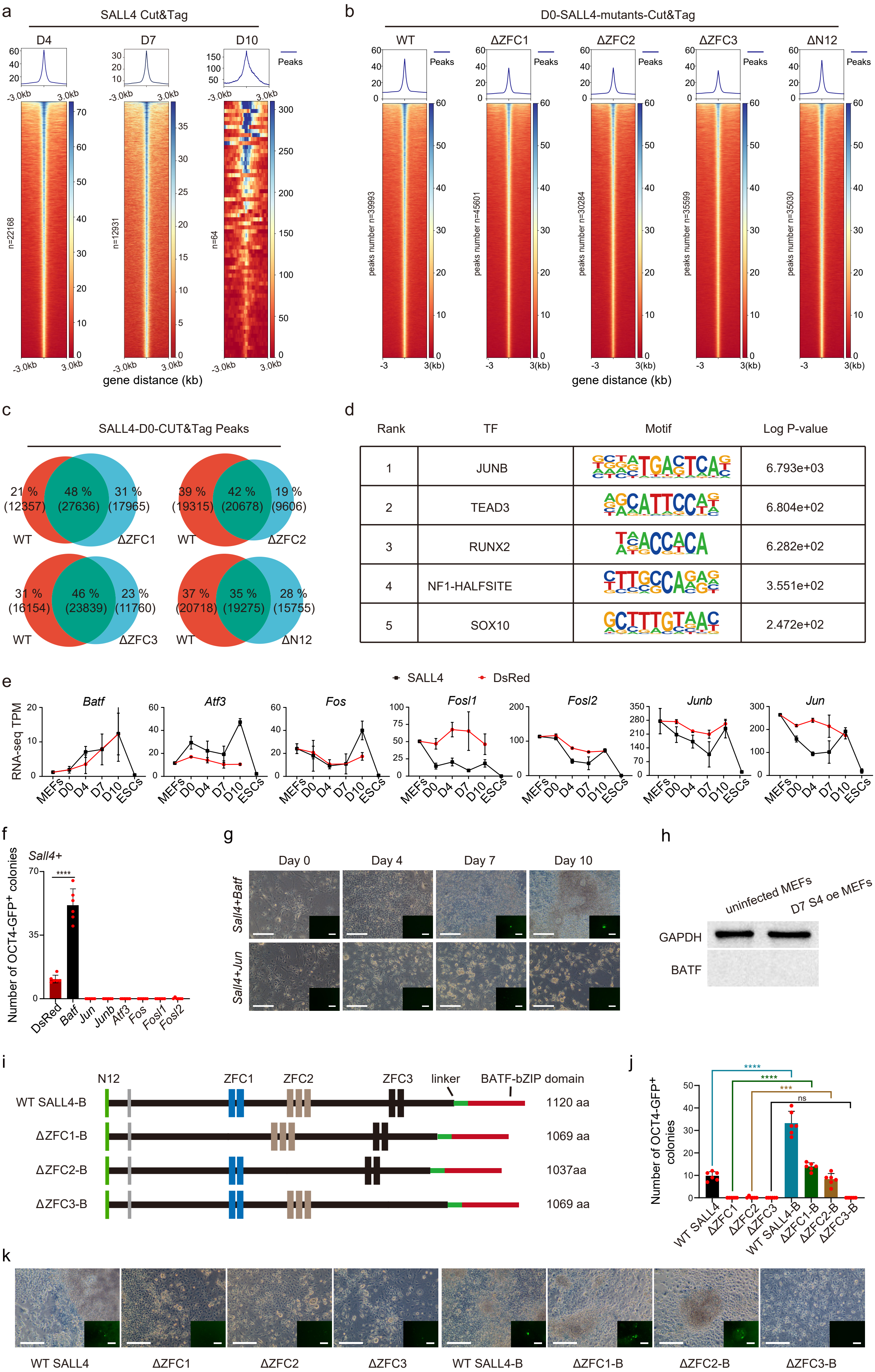
a
SALL4 Cut&Tag
D4
D7
D10
Peaks
n=22168
n=12931
n=64
gene distance (kb)
b
D0-SALL4-mutants-Cut&Tag
WT
ΔZFC1
ΔZFC2
ΔZFC3
ΔN12
peaks number n=39993
peaks number n=45601
peaks number n=30284
peaks number n=35599
peaks number n=35030
gene distance (kb)
c
SALL4-D0-CUT&Tag Peaks
21 % (12357) 48 % (27636) 31 % (17965)
WT ΔZFC1
39 % (19315) 42 % (20678) 19 % (9606)
WT ΔZFC2
31 % (16154) 46 % (23839) 23 % (11760)
WT ΔZFC3
37 % (20718) 35 % (19275) 28 % (15755)
WT ΔN12
d
Rank TF Motif Log P-value
1 JUNB 6.793e+03
2 TEAD3 6.804e+02
3 RUNX2 6.282e+02
4 NF1-HALFSITE 3.551e+02
5 SOX10 2.472e+02
e
SALL4 DsRed
Batf Atf3 Fos Fosl1 Fosl2 Junb Jun
RNA-seq TPM
MEFs D0 D4 D7 D10 ESCs
f
Sall4+
Number of OCT4-GFP+ colonies
DsRed Batf Jun Junb Atf3 Fos Fosl1 Fosl2
g
Day 0 Day 4 Day 7 Day 10
Sall4+Batf
Sall4+Jun
h
uninfected MEFs
D7 S4 oe MEFs
GAPDH
BATF
i
N12 ZFC1 ZFC2 ZFC3 linker BATF-bZIP domain
WT SALL4-B 1120 aa
ΔZFC1-B 1069 aa
ΔZFC2-B 1037aa
ΔZFC3-B 1069 aa
j
Number of OCT4-GFP+ colonies
WT SALL4 ΔZFC1 ΔZFC2 ΔZFC3 WT SALL4-B ΔZFC1-B ΔZFC2-B ΔZFC3-B
k
WT SALL4 ΔZFC1 ΔZFC2 ΔZFC3 WT SALL4-B ΔZFC1-B ΔZFC2-B ΔZFC3-B

Supplementary Fig.5 Cut&Tag analysis of SALL4 binding site during SALL4-mediated iPSCs reprogramming

a. Heatmap of Cut&Tag at D4,7,10 from SALL4, showing all binding peaks centred on the peak region within a 3 kb window around the peak. **b.** Heatmap of Cut&Tag at D0 from wide-type SALL4 (WT) and SALL4 mutants (ΔZFC1, ΔZFC2, ΔZFC3 and ΔN12), respectively. Showing all binding peaks centred on the peak region within a 3 kb window around the peak. **c.** Venn diagrams shows the overlapping numbers of day0 Cut&Tag peaks between WT-SALL4 and SALL4 mutants, respectively. **d.** De novo motif enrichment of SALL4-binding peaks. Statistical analysis was performed using Karlin/Altschul statistics. Top 5 motifs on each day ranked by -Log10(P-value) are shown, the left line (TF) of each chart shows proteins which could binding sequences most similar to the motif enriched by SALL4-binding peaks. **e.** RNA-seq shows the expression of representative genes in Fig.2e**.** DsRed, DsRed system. SALL4, SALL4 system. Data are mean ± SD**.** n = 2 samples for each time point from 2 independent experiments. **f.** The iPSCs induction efficiency using SALL4 overexpressing with representative SALL4-binding peaks enriched-motifs related genes (*Batf, Jun, Junb, Atf3, Fos, Fosl1* and *Fosl2*). Data are mean ± SD, Statistical analysis was performed using two-tailed, unpaired t test; n = 6 well from 3 independent experiments. *Sall4*+DsRed versus *Sall4*+*Batf*, ****p = 0.0000622. **g.** Morphological diagram for the iPSCs induction process using *Sall4* overexpressing with *Batf* or *Jun*, respectively. Scale bars, 200μm. The experiments were repeated independently three times with similar results. **h.** Western blot shows the absence of BATF protein in MEFs and the sample of D7 during SALL4-reprogramming. D7 S4 oe MEFs, D7 SALL4 overexpressed MEFs. **i.** Schematic representation of the protein sequences showing the structure of the wildtype SALL4 and SALL4 mutants which added the BATF-DNA binding domain. **j.** The number of OCT4-GFP$^+$ colonies on day 10 from 3 ×10^4 MEFs infected with SALL4 mutants in Supplementary Fig.5i. Data are mean ± SD**.** Statistical analysis was performed using two-tailed, unpaired t test; n = 6 well from 3 independent experiments. WT SALL4 versus WT SALL4-B, ****p = 0.0000388; ΔZFC1 versus ΔZFC1-B, ****p = 0.0000063; ΔZFC2 versus ΔZFC2-B, ***p = 0.0006027; **k.** Morphological diagram for the iPSCs induction process using the SALL4 mutants in Supplementary Fig.5j. Scale bars, 200μm. The experiments were repeated independently three times with similar results.

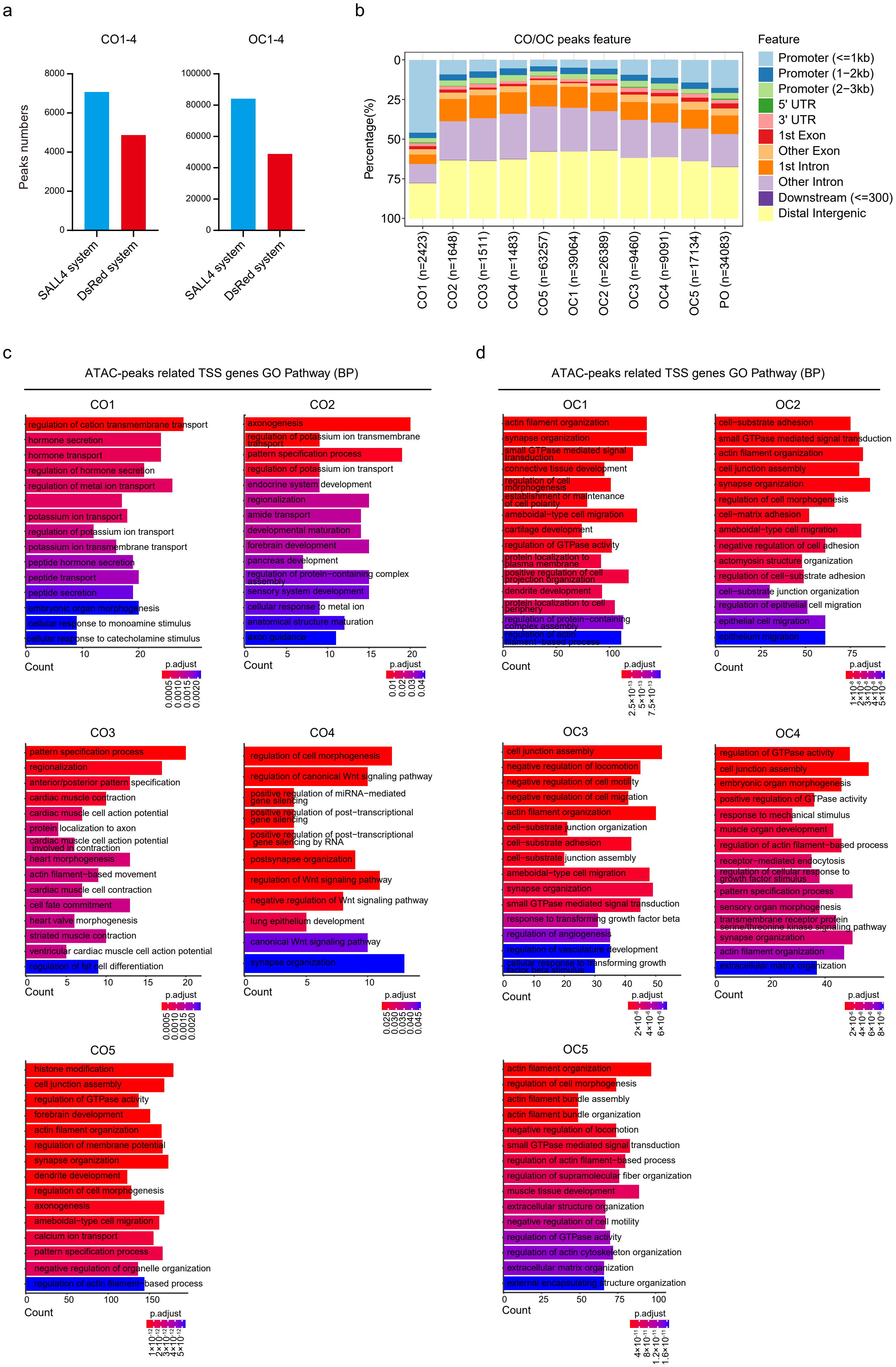
a
CO1-4
OC1-4
Peaks numbers
8000
6000
4000
2000
0
100000
80000
60000
40000
20000
0
SALL4 system
DsRed system
b
CO/OC peaks feature
Percentage(%)
0
25
50
75
100
CO1 (n=2423)
CO2 (n=1648)
CO3 (n=1511)
CO4 (n=1483)
CO5 (n=63257)
OC1 (n=39064)
OC2 (n=26389)
OC3 (n=9460)
OC4 (n=9091)
OC5 (n=17134)
PO (n=34083)
Feature
Promoter (<=1kb)
Promoter (1−2kb)
Promoter (2−3kb)
5' UTR
3' UTR
1st Exon
Other Exon
1st Intron
Other Intron
Downstream (<=300)
Distal Intergenic
c
ATAC-peaks related TSS genes GO Pathway (BP)
CO1
regulation of cation transmembrane transport
hormone secretion
hormone transport
regulation of hormone secretion
regulation of metal ion transport
potassium ion transport
regulation of potassium ion transport
potassium ion transmembrane transport
peptide hormone secretion
peptide transport
peptide secretion
embryonic organ morphogenesis
cellular response to monoamine stimulus
cellular response to catecholamine stimulus
Count
p.adjust
CO2
axonogenesis
regulation of potassium ion transmembrane transport
pattern specification process
regulation of potassium ion transport
endocrine system development
regionalization
amide transport
developmental maturation
forebrain development
pancreas development
regulation of protein−containing complex assembly
sensory system development
cellular response to metal ion
anatomical structure maturation
axon guidance
CO3
pattern specification process
regionalization
anterior/posterior pattern specification
cardiac muscle contraction
cardiac muscle cell action potential
protein localization to axon
cardiac muscle cell action potential involved in contraction
heart morphogenesis
actin filament−based movement
cardiac muscle cell contraction
cell fate commitment
heart valve morphogenesis
striated muscle contraction
ventricular cardiac muscle cell action potential
regulation of fat cell differentiation
CO4
regulation of cell morphogenesis
regulation of canonical Wnt signaling pathway
positive regulation of miRNA−mediated gene silencing
positive regulation of post−transcriptional gene silencing
positive regulation of post−transcriptional gene silencing by RNA
postsynapse organization
regulation of Wnt signaling pathway
negative regulation of Wnt signaling pathway
lung epithelium development
canonical Wnt signaling pathway
synapse organization
CO5
histone modification
cell junction assembly
regulation of GTPase activity
forebrain development
actin filament organization
regulation of membrane potential
synapse organization
dendrite development
regulation of cell morphogenesis
axonogenesis
ameboidal−type cell migration
calcium ion transport
pattern specification process
negative regulation of organelle organization
regulation of actin filament−based process
d
ATAC-peaks related TSS genes GO Pathway (BP)
OC1
actin filament organization
synapse organization
small GTPase mediated signal transduction
connective tissue development
regulation of cell morphogenesis
establishment or maintenance of cell polarity
ameboidal−type cell migration
cartilage development
regulation of GTPase activity
protein localization to plasma membrane
positive regulation of cell projection organization
dendrite development
protein localization to cell periphery
regulation of protein−containing complex assembly
regulation of actin filament−based process
OC2
cell−substrate adhesion
small GTPase mediated signal transduction
actin filament organization
cell junction assembly
synapse organization
regulation of cell morphogenesis
cell−matrix adhesion
ameboidal−type cell migration
negative regulation of cell adhesion
actomyosin structure organization
regulation of cell−substrate adhesion
cell−substrate junction organization
regulation of epithelial cell migration
epithelial cell migration
epithelium migration
OC3
cell junction assembly
negative regulation of locomotion
negative regulation of cell motility
negative regulation of cell migration
actin filament organization
cell−substrate junction organization
cell−substrate adhesion
cell−substrate junction assembly
ameboidal−type cell migration
synapse organization
small GTPase mediated signal transduction
response to transforming growth factor beta
regulation of angiogenesis
regulation of vasculature development
cellular response to transforming growth factor beta stimulus
OC4
regulation of GTPase activity
cell junction assembly
embryonic organ morphogenesis
positive regulation of GTPase activity
response to mechanical stimulus
muscle organ development
regulation of actin filament−based process
receptor−mediated endocytosis
regulation of cellular response to growth factor stimulus
pattern specification process
sensory organ morphogenesis
transmembrane receptor protein serine/threonine kinase signaling pathway
synapse organization
actin filament organization
extracellular matrix organization
OC5
actin filament organization
regulation of cell morphogenesis
actin filament bundle assembly
actin filament bundle organization
negative regulation of locomotion
small GTPase mediated signal transduction
regulation of actin filament−based process
regulation of supramolecular fiber organization
muscle tissue development
extracellular structure organization
negative regulation of cell motility
regulation of GTPase activity
regulation of actin cytoskeleton organization
extracellular matrix organization
external encapsulating structure organization
Count
p.adjust

Supplementary Fig.6 The distribution and GO analysis of SALL4-ATAC-peaks in genomic loci.

a. The histogram shows the overall number of the peaks for CO1-4 and OC1-4 subgroups of SALL4 system and DsRed system. **b.** Genome distribution of the location for SALL4-ATAC-seq close to open peaks(CO) and open to close peaks(OC) relative to the nearest annotated genes. **c-d.** GO analysis for genes annotated by CO and OC peaks relative to the nearest TSS loc**i.** Statistical analysis was performed using Fisher's exact test, the adjust p value for each term are shown.

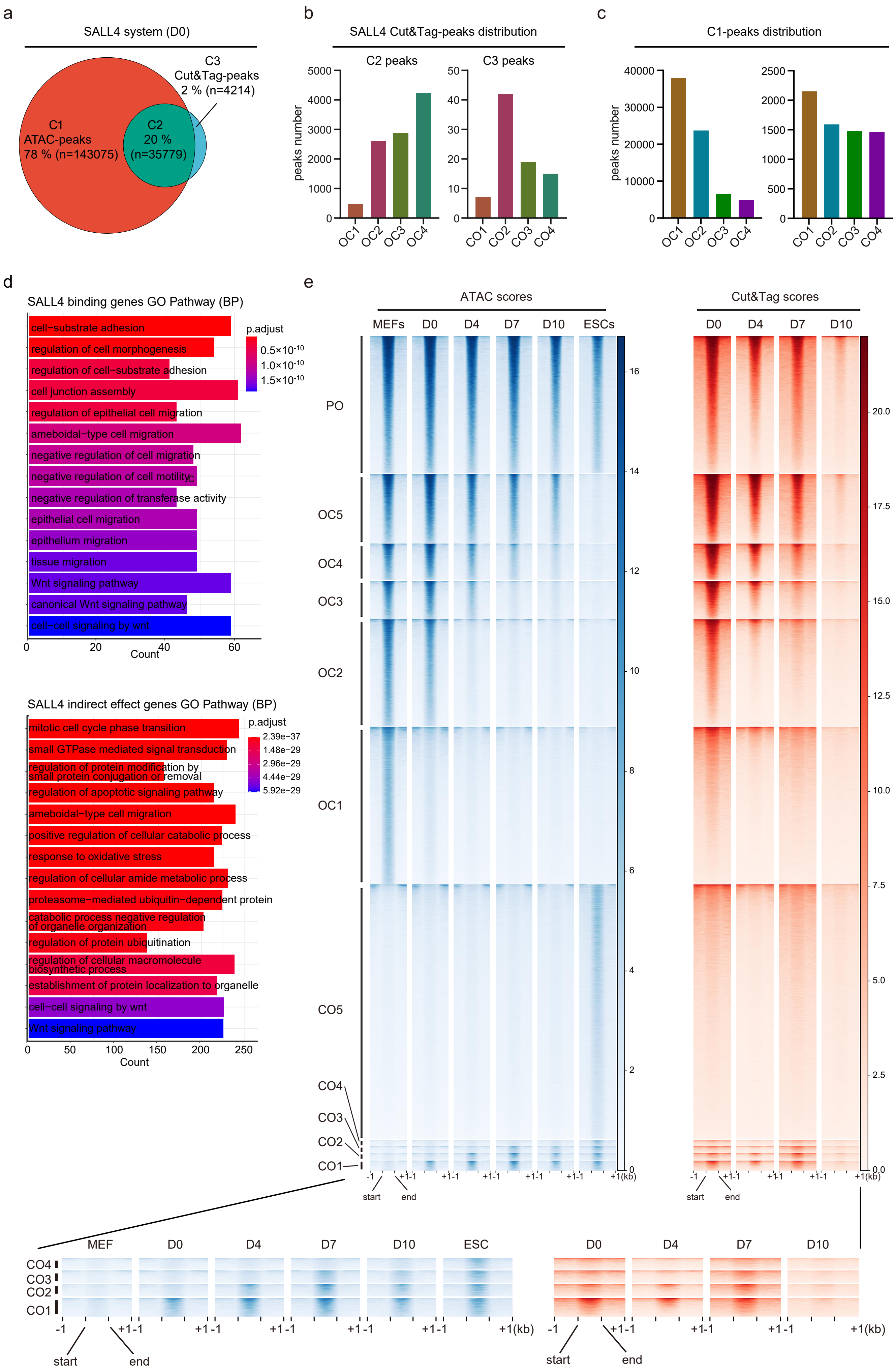

a
SALL4 system (D0)
C1
ATAC-peaks
78 % (n=143075)
C2
20 %
(n=35779)
C3
Cut&Tag-peaks
2 % (n=4214)
b
SALL4 Cut&Tag-peaks distribution
C2 peaks
C3 peaks
peaks number
OC1 OC2 OC3 OC4
c
C1-peaks distribution
CO1 CO2 CO3 CO4
d
SALL4 binding genes GO Pathway (BP)
cell−substrate adhesion
regulation of cell morphogenesis
regulation of cell−substrate adhesion
cell junction assembly
regulation of epithelial cell migration
ameboidal−type cell migration
negative regulation of cell migration
negative regulation of cell motility
negative regulation of transferase activity
epithelial cell migration
epithelium migration
tissue migration
Wnt signaling pathway
canonical Wnt signaling pathway
cell−cell signaling by wnt
p.adjust
Count
SALL4 indirect effect genes GO Pathway (BP)
mitotic cell cycle phase transition
small GTPase mediated signal transduction
regulation of protein modification by small protein conjugation or removal
regulation of apoptotic signaling pathway
ameboidal−type cell migration
positive regulation of cellular catabolic process
response to oxidative stress
regulation of cellular amide metabolic process
proteasome−mediated ubiquitin−dependent protein catabolic process
negative regulation of organelle organization
regulation of protein ubiquitination
regulation of cellular macromolecule biosynthetic process
establishment of protein localization to organelle
cell−cell signaling by wnt
Wnt signaling pathway
2.39e−37
1.48e−29
2.96e−29
4.44e−29
5.92e−29
e
ATAC scores
MEFs D0 D4 D7 D10 ESCs
Cut&Tag scores
D0 D4 D7 D10
PO
OC5
OC4
OC3
OC2
OC1
CO5
CO4
CO3
CO2
CO1
start
end
(kb)
MEF D0 D4 D7 D10 ESC

Supplementary Fig.7 The whole regulatory landscape analysis during SALL4-driven reprogramming

a. Venn diagrams shows the overlapping numbers between SALL4 Cut&Tag peaks and ATAC-seq peaks in SALL4 system at day0. **b.** SALL4 Cut&Tag peaks (C2 and C3) distribution in ATAC CO and OC subgroups. **c.** The histogram shows the Number of the peaks in CO and OC subgroups from ATAC-peaks overlap without Cut&Tag-peaks (C1). **d.** Top, GO analysis for genes annotated by Cut&Tag peaks in Supplementary Fig.7b. Bottom, GO analysis for genes annotated by ATAC peaks in Supplementary Fig.7c. Statistical analysis was performed using Fisher's exact test, the adjust p value for each term are shown. **e.** Heatmaps shows the CADs and SALL4-binding landscape during SALL4-driven reprogramming. The subgroups (CO and OC) were based on the classification of SALL4-ATAC data as described in results.

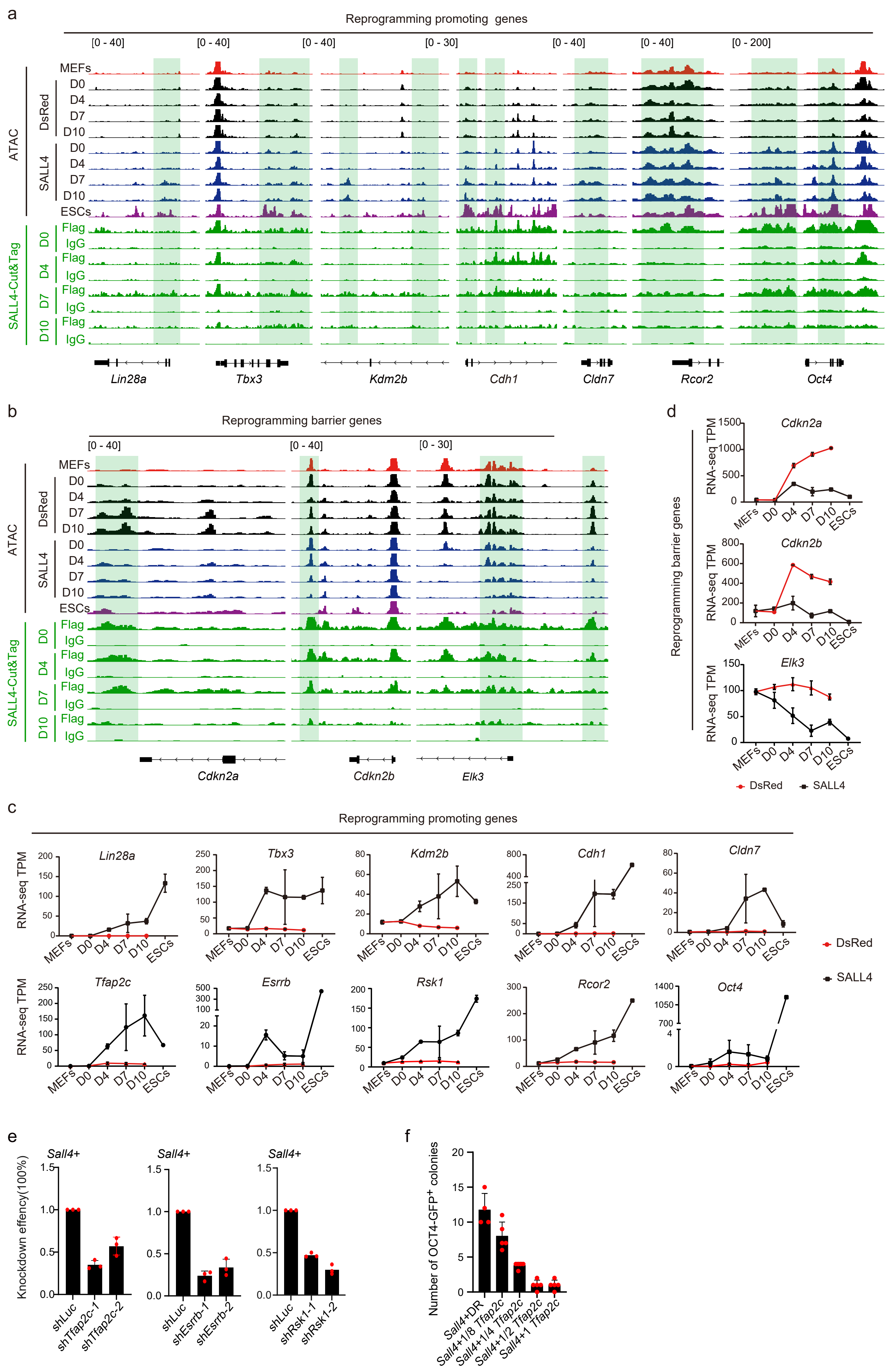

a
Reprogramming promoting genes
[0 - 40] [0 - 40] [0 - 40] [0 - 30] [0 - 40] [0 - 40] [0 - 200]
ATAC
MEFs
DsRed
SALL4
D0
D4
D7
D10
ESCs
SALL4-Cut&Tag
Flag
IgG
Lin28a
Tbx3
Kdm2b
Cdh1
Cldn7
Rcor2
Oct4
b
Reprogramming barrier genes
[0 - 40] [0 - 40] [0 - 30]
Cdkn2a
Cdkn2b
Elk3
c
Reprogramming promoting genes
RNA-seq TPM
Lin28a
Tbx3
Kdm2b
Cdh1
Cldn7
Tfap2c
Esrrb
Rsk1
Rcor2
Oct4
MEFs
ESCs
DsRed
SALL4
d
Reprogramming barrier genes
e
Sall4+
Knockdown effency(100%)
shLuc
shTfap2c-1
shTfap2c-2
shEsrrb-1
shEsrrb-2
shRsk1-1
shRsk1-2
f
Number of OCT4-GFP+ colonies
Sall4+DR
Sall4+1/8 Tfap2c
Sall4+1/4 Tfap2c
Sall4+1/2 Tfap2c
Sall4+1 Tfap2c

Supplementary Fig.8 Genes that promote iPSCs reprogramming are upregulated in SALL4-mediated iPSCs induction

a. ATAC-seq data and Cut&Tag data shows the peaks of representative reprogramming promoting genes and genomic loc**i.** DsRed, DsRed system. SALL4, SALL4 system. **b.** ATAC-seq data and Cut&Tag data shows the peaks of representative reprogramming barrier genes and genomic loc**i.** DsRed, DsRed system. SALL4, SALL4 system. **c.** RNA-seq data shows the expression of representative genes in **a**. Data are mean ± SD**.** n = 2 samples for each time point from 2 independent experiments. DsRed, DsRed system. SALL4, SALL4 system. **d.** RNA-seq data shows the expression level of representative genes in **b**. Data are mean ± SD**.** n = 2 samples for each time point from 2 independent experiments. DsRed, DsRed system. SALL4, SALL4 system. **e.** RT-qPCR showing the reduction of *Tfap2c, Esrrb* and *Rsk1* at mRNA level in the knockdown(sh*Tfap2c*) but not control(shLuc) cell lin**e.** The relative expression levels are normalized to shLuc. Data are mean ± SD; n = 3 biological replicates. **f.** The number of OCT4-GFP$^+$ colonies on day 10 from 3 ×10^4 MEFs infected with SALL4 and different volume of *Tfap2c* retroviral supernatants in iCD4.

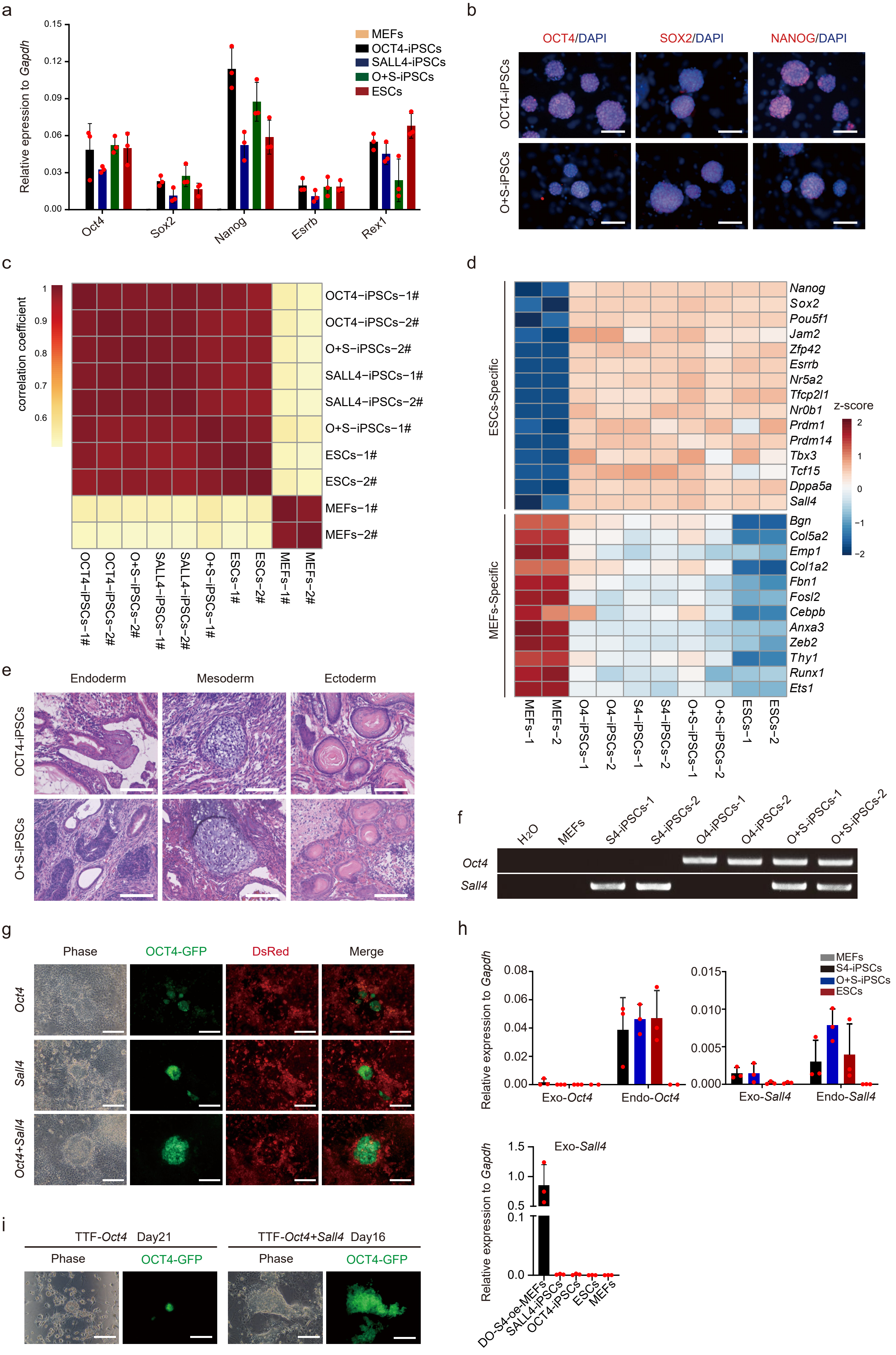
a
Relative epression to Gapdh
0.15
0.12
0.09
0.06
0.03
0.00
MEFs
OCT4-iPSCs
SALL4-iPSCs
O+S-iPSCs
ESCs
Oct4
Sox2
Nanog
Esrrb
Rex1
b
OCT4/DAPI
SOX2/DAPI
NANOG/DAPI
OCT4-iPSCs
O+S-iPSCs
c
correlation coefficient
1
0.9
0.8
0.7
0.6
OCT4−iPSCs−1#
OCT4−iPSCs−2#
O+S−iPSCs−2#
SALL4−iPSCs−1#
SALL4−iPSCs−2#
O+S−iPSCs−1#
ESCs−1#
ESCs−2#
MEFs−1#
MEFs−2#
d
ESCs-Specific
MEFs-Specific
z-score
2
1
0
−1
−2
Nanog
Sox2
Pou5f1
Jam2
Zfp42
Esrrb
Nr5a2
Tfcp2l1
Nr0b1
Prdm1
Prdm14
Tbx3
Tcf15
Dppa5a
Sall4
Bgn
Col5a2
Emp1
Col1a2
Fbn1
Fosl2
Cebpb
Anxa3
Zeb2
Thy1
Runx1
Ets1
MEFs−1
MEFs−2
O4−iPSCs−1
O4−iPSCs−2
S4−iPSCs−1
S4−iPSCs−2
O+S−iPSCs−1
O+S−iPSCs−2
ESCs−1
ESCs−2
e
Endoderm
Mesoderm
Ectoderm
OCT4-iPSCs
O+S-iPSCs
f
H2O
MEFs
S4-iPSCs-1
S4-iPSCs-2
O4-iPSCs-1
O4-iPSCs-2
O+S-iPSCs-1
O+S-iPSCs-2
Oct4
Sall4
g
Phase
OCT4-GFP
DsRed
Merge
Oct4
Sall4
Oct4+Sall4
h
Relative expression to Gapdh
0.08
0.06
0.04
0.02
0.00
Exo-Oct4
Endo-Oct4
0.015
0.010
0.005
0.000
Exo-Sall4
Endo-Sall4
MEFs
S4-iPSCs
O+S-iPSCs
ESCs
Relative expression to Gapdh
1.5
1.0
0.5
0.1
0.0
Exo-Sall4
DO-S4-oe-MEFs
SALL4-iPSCs
OCT4-iPSCs
ESCs
MEFs
i
TTF-Oct4 Day21
TTF-Oct4+Sall4 Day16
Phase
OCT4-GFP
Phase
OCT4-GFP

Supplementary Fig.9 OCT4-iPSCs and O+S-iPSCs has pluripotency

a. qRT-PCR analysis of pluripotency markers in SALL4-iPSCs, OCT4-iPSCs and O+S-iPSCs. Data are mean ± SD; n = 3 biological replicates. O+S, OCT4+SALL4. **b.** Immunofluorescence analysis of pluripotency markers in SALL4-iPSCs, OCT4-iPSCs and O+S-iPSCs. Scale bars, 200μm. O+S, OCT4+SALL4. The experiments were repeated independently three times with similar results. **c.** Correlation analysis for RNA-seq from SALL4-iPSCs OCT4-iPSCs, O+S-iPSCs, MEFs and ESCs. n = 2 biological replicates. O+S, OCT4+SALL4. **d.** Heatmaps for the expression of Naive marker and MEFs specific genes in SALL4-iPSCs, OCT4-iPSCs, O+S-iPSCs, ESCs and MEFs in RNA-seq data. O4, OCT4. S4, SALL4. O+S, OCT4+SALL4. **e.** The three germ layers of a teratoma from OCT4-iPSCs and O+S-iPSCs. Endoderm, simple cuboidal epithelium. Mesoderm, chondrocyte. Ectoderm, skin tissue. Scale bars, 100μm. O+S, OCT4+SALL4. The experiments were repeated independently three times with similar results. **f.** Genomic insertion identification confirms the derivation of O4, S4 and O+S iPSCs colonies. The presence of the retroviral transgene was examined by PCR. n = 2 biological replicates. O4, OCT4. S4, SALL4. O+S, OCT4+SALL4. **g.** Exogenous gene silencing in OCT4-GFP^+ colonies. MEFs overexpressed *Sall4*, *Oct4* or *Sall4*+*Oct4* were co-infected with DsRed, respectively. Scale bars, 200μm. The experiments were repeated independently three times with similar results. **h.** qRT-PCR analysis of exogenous gene expression in iPSCs, MEFs and ESCs. Data are mean ± SD; n = 3 biological replicates. D0-S4-oe-MEFs, D0-*Sall4*-overexpression-MEFs. S4, SALL4. O+S, OCT4+SALL4. **i.** The morphology of OCT4-GFP^+cells induced by OCT4 or SALL4+OCT4 from TTFs. Scale bars, 200μm. The experiments were repeated independently three times with similar results.

a

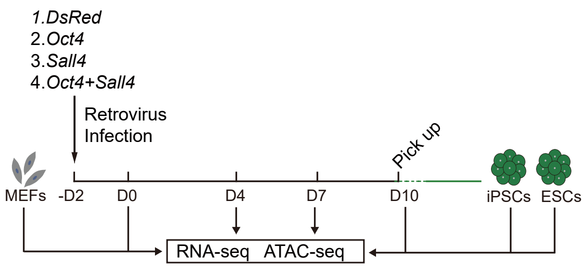

b

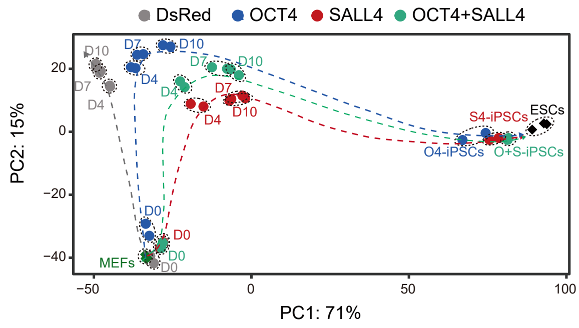

c

Cluster	GO biological process and count	-Log10(P-value)
S4/O+S C-up (UC7)	multicellular organism development(58)	4.5053
	cell differentiation(52)	3.6474
	epithelial cell differentiation(10)	2.4544
	negative regulation of cell migration(12)	2.2046
	**epithelial cell morphogenesis(5)**	2.1683
	regulation of transcription from RNA polymerase II promoter(65)	1.9969
	**spermatogenesis(26)**	1.9059
	cell proliferation(19)	1.7864
	**stem cell proliferation(6)**	1.3394
O4/O+S C-up (UC10)	**neuron differentiation(14)**	5.3184
	multicellular organism development(29)	4.3474
	cell differentiation(27)	4.0346
	neuron development(7)	3.8738
	**nervous system development(16)**	3.8627
	phosphorylation(18)	3.1307
	intracellular signal transduction(12)	1.9073
	neuron migration(6)	1.5148
	regulation of transcription from RNA polymerase II promoter(27)	1.4114
O+S Sp-up (UC19)	**DNA methylation involved in gamete generation(6)**	5.6312
	piRNA metabolic process(6)	5.5171
	**spermatogenesis(18)**	4.0447
	meiotic cell cycle(11)	3.7386
	male meiosis I(5)	3.3572
	**stem cell population maintenance(6)**	2.7770
	germ cell development(6)	2.5410
	glutathione metabolic process(5)	2.2806
	regulation of transcription from RNA polymerase II promoter(30)	2.0844

d

Cluster	GO biological process and count	-Log10(P-value)
S4/O+S C-D (DC9)	cell adhesion(73)	17.796
	homophilic cell adhesion via plasma membrane adhesion molecules(35)	14.858
	positive regulation of synapse assembly(19)	9.7254
	regulation of presynapse assembly(14)	9.2070
	**positive regulation of neuron projection development(25)**	7.0699
	memory(18)	5.6926
	modulation of synaptic transmission(18)	5.0196
	**nervous system development(36)**	4.5833
O4/O+S C-D (DC12)	extracellular matrix organization(13)	7.1391
	**negative regulation of neuron apoptotic process(10)**	4.3000
	positive regulation of cell migration(11)	3.9112
	positive regulation of endothelial cell migration(6)	3.6676
	positive regulation of angiogenesis(8)	3.3046
	positive regulation of fibroblast proliferation(6)	3.2353
	**inflammatory response(11)**	2.6225
	intracellular signal transduction(11)	2.2453
O+S Sp-D (DC21)	membrane raft assembly(3)	2.9179
	**endocytosis(7)**	2.2852
	prepulse inhibition(3)	2.0334
	**integrin-mediated signaling pathway(5)**	2.0232
	cell-substrate adhesion(3)	1.8720
	hydrogen ion transmembrane transport(4)	1.8412
	actin cytoskeleton reorganization(4)	1.8237
	modulation of inhibitory postsynaptic potential(2)	1.8137

e

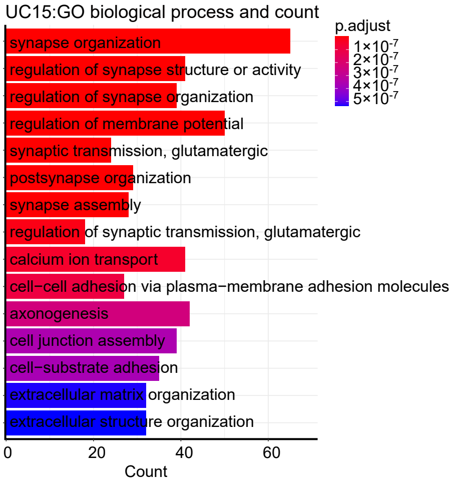

Supplementary Fig.10 Transcriptome analysis in SALL4, OCT4 and O+S- mediated iPSCs reprogramming process

a. Diagram for RNA-seq and ATAC-seq data collecting during SALL4, OCT4 and OCT4+SALL4 reprogramming process. **b.** PCA analysis for RNA-seq data from DsRed, SALL4, OCT4 and O+S system. RNA-seq data were collected from two independent experiment. DR, DsRed system. O4, OCT4 system. S4, SALL4 system. O+S, OCT4+SALL4 system. **c.** GO analysis for the clusters of genes in Fig.4e, which contains specific-upregulated in O+S and O+S co-upregulated with SALL4 or OCT4, respectively. Statistical analysis was performed using Fisher's exact test, the -Log10(p value) for each term are shown. O4, OCT4 system. S4, SALL4 system. O+S, OCT4+SALL4 system. C-up, common upregulated. sp-up, specific upregulated. **d.** GO analysis for the clusters of genes in Fig.4f, which contains specific-downregulated in O+S and O+S co-downregulated with SALL4 or OCT4, respectively. Statistical analysis was performed using Fisher's exact test, the -Log10(p value) for each term are shown. O4, OCT4 system. S4, SALL4 system. O+S, OCT4+SALL4 system. C-D, common downregulated. sp-D, specific downregulated. **e.** GO analysis for the clusters of genes in Fig.4e, which contains specific-upregulated in OCT4 system. Statistical analysis was performed using Fisher's exact test, the adjust p value for each term are shown.

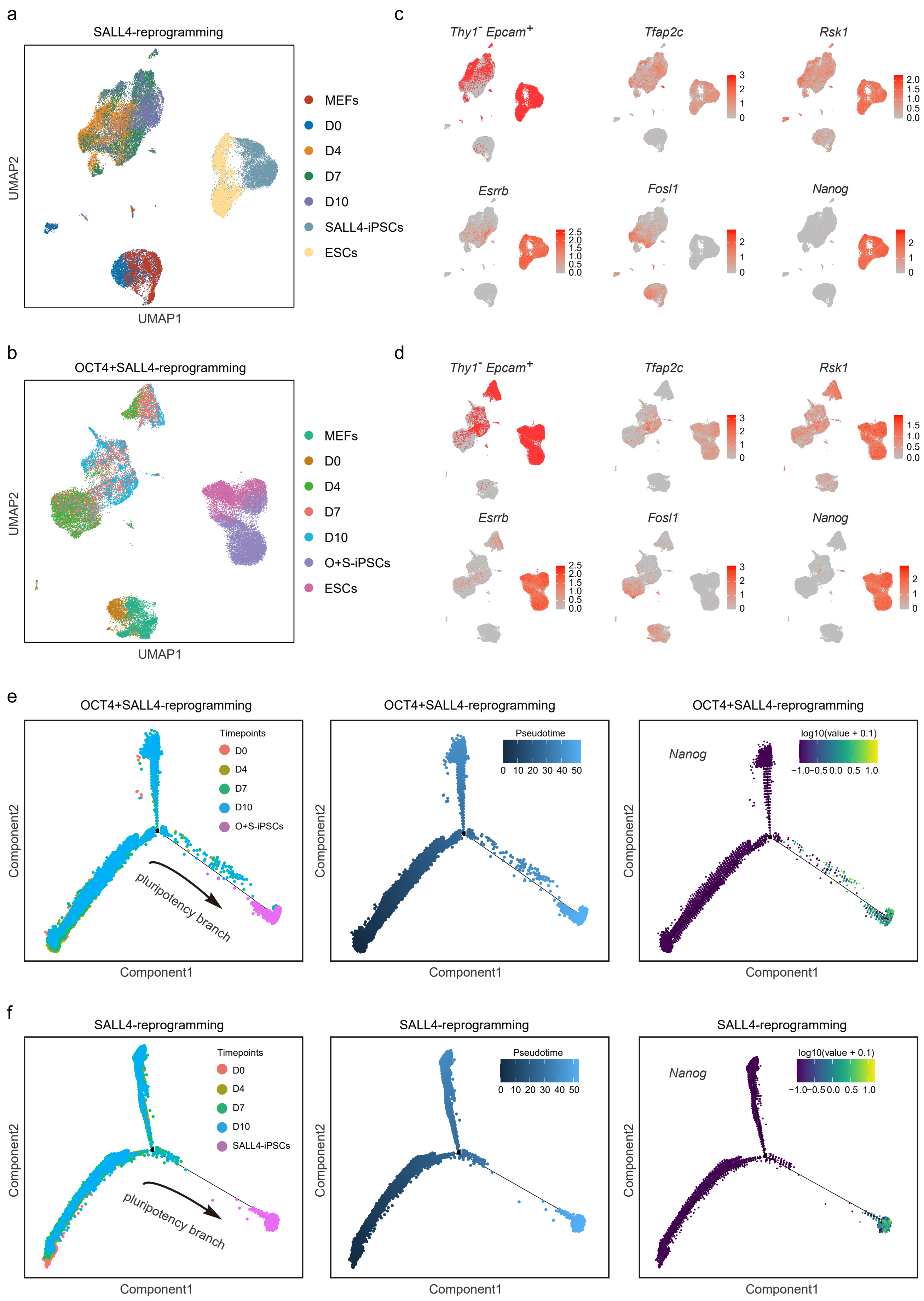
a
SALL4-reprogramming
UMAP2
UMAP1
MEFs
D0
D4
D7
D10
SALL4-iPSCs
ESCs
b
OCT4+SALL4-reprogramming
UMAP2
UMAP1
MEFs
D0
D4
D7
D10
O+S-iPSCs
ESCs
c
Thy1- Epcam+
Tfap2c
Rsk1
Esrrb
Fosl1
Nanog
d
Thy1- Epcam+
Tfap2c
Rsk1
Esrrb
Fosl1
Nanog
e
OCT4+SALL4-reprogramming
Timepoints
D0
D4
D7
D10
O+S-iPSCs
pluripotency branch
Pseudotime
0 10 20 30 40 50
Nanog
log10(value + 0.1)
−1.0 −0.5 0.0 0.5 1.0
Component2
Component1
f
SALL4-reprogramming
Timepoints
D0
D4
D7
D10
SALL4-iPSCs
pluripotency branch
Pseudotime
0 10 20 30 40 50
Nanog
log10(value + 0.1)
−1.0 −0.5 0.0 0.5 1.0
Component2
Component1

Supplementary Fig.11 Single-cell RNA sequencing of SALL4 or OCT4+SALL4-driven reprogramming process

a-b. UMAP plot for Single-cell RNA-seq data from SALL4-mediated reprogramming (**a**) and OCT4+SALL4 reprogramming process (**b**). Each dot represents one cell. The sampling time points are shown with color code. **c-d.** The expression of representative markers in SALL4-mediated reprogramming (**c**) and OCT4+SALL4 reprogramming process (**d**). **e-f.** Monocle trajectories of reprogramming samples mediated by OCT4+SALL4 (**e**) and SALL4 alone (**f**) are depicted with color-coding representing reprogramming timepoints (left), pseudotime (middle), and Nanog expression levels (right). Each data point represents an individual cell, with the ordering of cells inferred based on the expression patterns of the most variable genes across reprogramming samples.

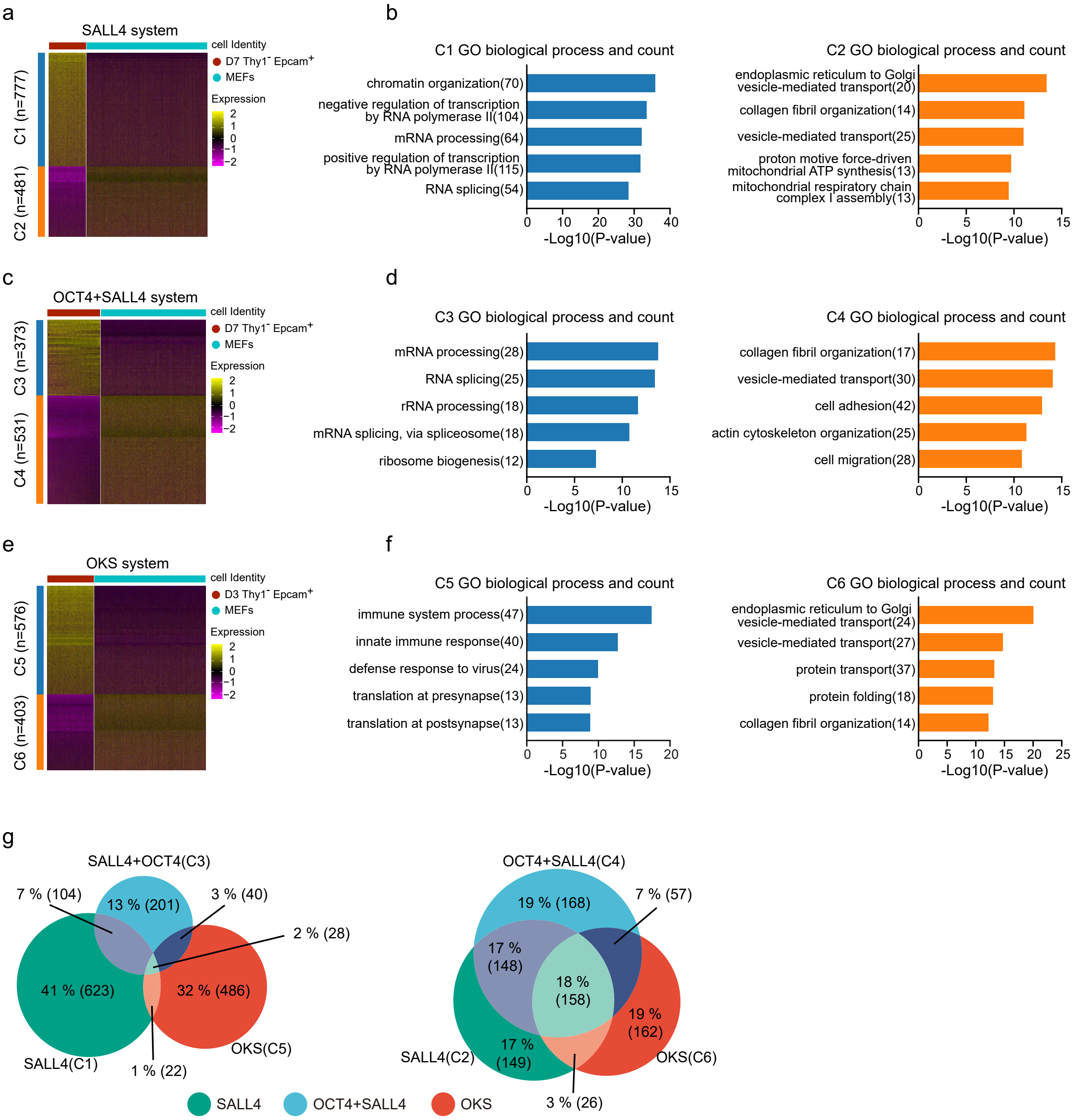
a
SALL4 system
cell Identity
D7 Thy1- Epcam+
MEFs
Expression
C1 (n=777)
C2 (n=481)
b
C1 GO biological process and count
chromatin organization(70)
negative regulation of transcription by RNA polymerase II(104)
mRNA processing(64)
positive regulation of transcription by RNA polymerase II(115)
RNA splicing(54)
-Log10(P-value)
C2 GO biological process and count
endoplasmic reticulum to Golgi vesicle-mediated transport(20)
collagen fibril organization(14)
vesicle-mediated transport(25)
proton motive force-driven mitochondrial ATP synthesis(13)
mitochondrial respiratory chain complex I assembly(13)
-Log10(P-value)
c
OCT4+SALL4 system
cell Identity
D7 Thy1- Epcam+
MEFs
Expression
C3 (n=373)
C4 (n=531)
d
C3 GO biological process and count
mRNA processing(28)
RNA splicing(25)
rRNA processing(18)
mRNA splicing, via spliceosome(18)
ribosome biogenesis(12)
-Log10(P-value)
C4 GO biological process and count
collagen fibril organization(17)
vesicle-mediated transport(30)
cell adhesion(42)
actin cytoskeleton organization(25)
cell migration(28)
-Log10(P-value)
e
OKS system
cell Identity
D3 Thy1- Epcam+
MEFs
Expression
C5 (n=576)
C6 (n=403)
f
C5 GO biological process and count
immune system process(47)
innate immune response(40)
defense response to virus(24)
translation at presynapse(13)
translation at postsynapse(13)
-Log10(P-value)
C6 GO biological process and count
endoplasmic reticulum to Golgi vesicle-mediated transport(24)
vesicle-mediated transport(27)
protein transport(37)
protein folding(18)
collagen fibril organization(14)
-Log10(P-value)
g
SALL4+OCT4(C3)
7 % (104)
13 % (201)
3 % (40)
2 % (28)
41 % (623)
32 % (486)
SALL4(C1)
1 % (22)
OKS(C5)
OCT4+SALL4(C4)
19 % (168)
7 % (57)
17 % (148)
18 % (158)
19 % (162)
17 % (149)
SALL4(C2)
OKS(C6)
3 % (26)
SALL4
OCT4+SALL4
OKS

Supplementary Fig.12 Diverse Characteristics of Thy1^{-}/Epcam^{+} Intermediates in distinct Reprogramming processes

a-f. Heatmaps of differential expression gene analysis for scRNA-seq data from SALL4 system (**a**), OCT4+SALL4 system (**c**) and OKS systems (**e**). The 6 subgroups (C1-C6) of differental expression genes were based on gene expression between MEFs and THY1^{-}/EPCAM^{+} cells. GO analysis for each subgroup are shown (**b**, **d** and **f**). Statistical analysis for GO was performed using Fisher's exact test, the -Log10(p value) for each term are shown. **g.** Venn diagrams shows the number of differental expression genes of THY1^{-}/EPCAM^{+} cells between SALL4 system, OCT4+SALL4 system and OKS system.

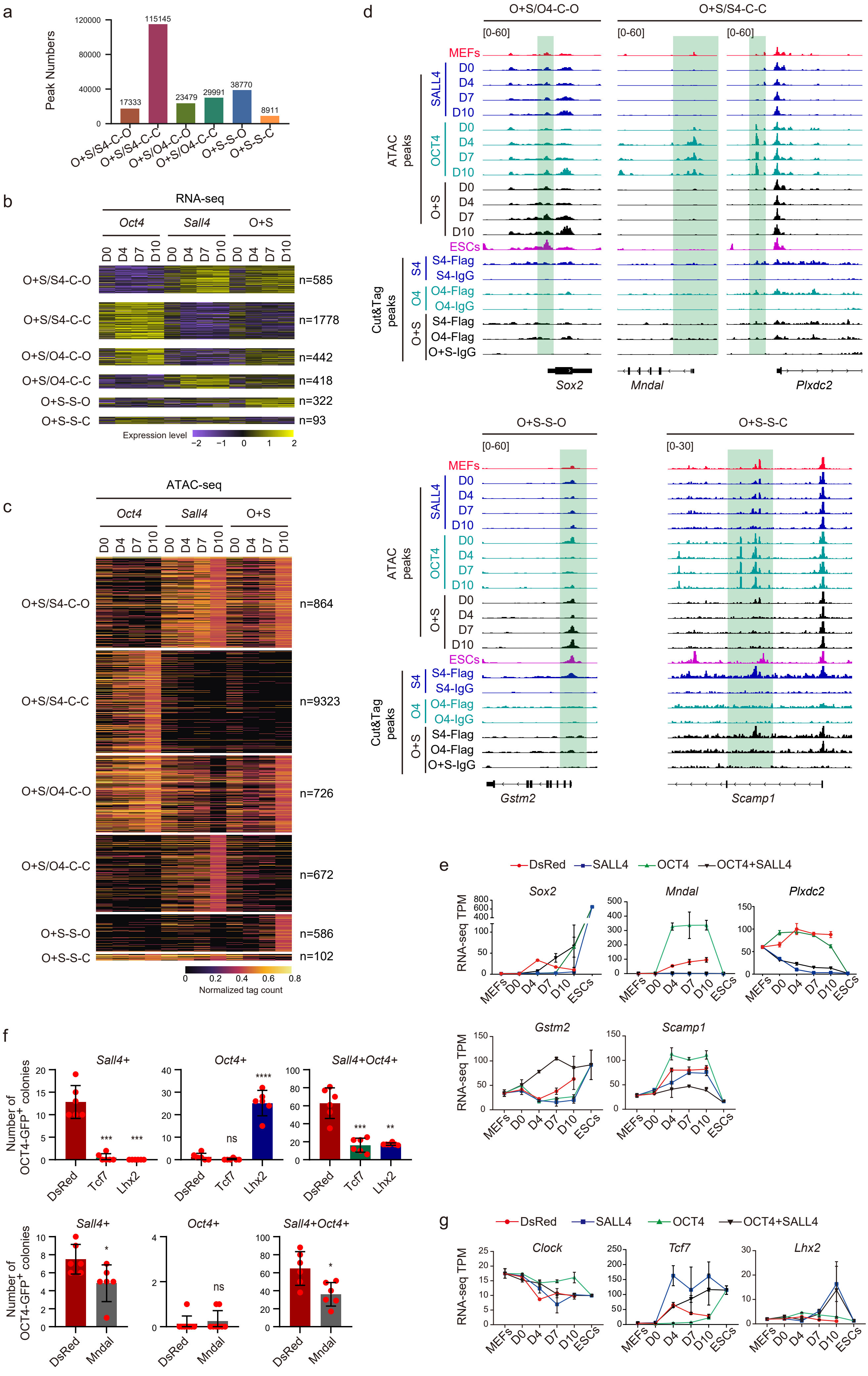
a
Peak Numbers
120000
80000
40000
0
17333
115145
23479
29991
38770
8911
O+S/S4-C-O
O+S/S4-C-C
O+S/O4-C-O
O+S/O4-C-C
O+S-S-O
O+S-S-C
b
RNA-seq
Oct4
Sall4
O+S
D0 D4 D7 D10
n=585
n=1778
n=442
n=418
n=322
n=93
Expression level
−2 −1 0 1 2
c
ATAC-seq
n=864
n=9323
n=726
n=672
n=586
n=102
0 0.2 0.4 0.6 0.8
Normalized tag count
d
[0-60]
[0-30]
MEFs
SALL4
OCT4
ESCs
ATAC peaks
Cut&Tag peaks
S4-Flag
S4-IgG
O4-Flag
O4-IgG
O+S-IgG
Sox2
Mndal
Plxdc2
Gstm2
Scamp1
e
DsRed
SALL4
OCT4
OCT4+SALL4
RNA-seq TPM
MEFs D0 D4 D7 D10 ESCs
f
Sall4+
Oct4+
Sall4+Oct4+
Number of OCT4-GFP+ colonies
DsRed
Tcf7
Lhx2
Mndal
ns
g
Clock
Tcf7
Lhx2

Supplementary Fig.13 SALL4 and OCT4 can regulate reprogramming-relative genes in O+S-mediated iPSCs induction

a. The histogram shows the number of the peaks for CADs patterns. O+S/O4-C-O, OCT4+SALL4/OCT4-Common-open; O+S/O4-C-C, OCT4+SALL4/OCT4-Common-close; O+S/S4-C-O, OCT4+SALL4/ SALL4-Common-open; O+S/S4-C-C, OCT4+SALL4/SALL4-Common-close; O+S-S-O, OCT4+SALL4 Specific-open; O+S-S-C, OCT4+SALL4 Specific-close. **b.** Heatmaps of differential expression genes related to CADs patterns for RNA-seq data from O+S system, SALL4 system and OCT4 system. **c.** Heatmaps of CADs patterns corresponding to differential expression genes in Supplementary Fig.13b. The differential transition peaks for each systems are shown. **d.** Representative Genomic loci and genes for O+S-S-O peaks, O+S/O4-C-O peaks, O+S-S-C peaks, O+S/S4-C-C peaks and Cut&Tag-binding peaks. O4, OCT4. S4, SALL4. O+S, OCT4+SALL4; O+S/O4-C-O, OCT4+SALL4/ OCT4-Common-open; O+S/S4-C-C, OCT4+SALL4/SALL4-Common-close; O+S-S-O, OCT4+SALL4 Specific-open; O+S-S-C, OCT4+SALL4 Specific-close. **e.** RNA-seq data shows the expression of representative genes in Supplementary Fig.13d. Data are mean ± SD. n = 2 samples for each time point from 2 independent experiments. DsRed, DsRed system. OCT4, OCT4 system. SALL4, SALL4 system. OCT4+SALL4, OCT4+SALL4 system. **f.** The iPSCs induction efficiency using *Sall4*, *Oct4* or *Oct4*+*Sall4* overepressing with *Tcf7*, *Lhx2* or *Mndal*. Data are mean ± SD**.** Statistical analysis was performed using two-tailed, unpaired t test; n = 6 well from 3 independent experiments. *Sall4*+DsRed versus *Sall4*+Tcf7, ***p = 0.0002964; *Sall4*+DsRed versus *Sall4*+*Lhx2*, ***p = 0.0003510; *Sall4*+DsRed versus *Sall4*+*Mndal*, *p = 0.0328333; *Oct4*+DsRed versus *Oct4*+*Tcf7*, n.s. p = 0.1189675; *Oct4*+DsRed versus *Oct4*+*Lhx2*, ****p = 0.0000815; *Oct4*+DsRed versus *Oct4*+*Mndal*, n.s. p = 0.5497377; *Sall4*+*Oct4*+DsRed versus *Sall4*+*Oct4*+*Tcf7*, ***p = 0.0004947; *Sall4*+*Oct4*+DsRed versus *Sall4*+*Oct4*+*Lhx2*, **p = 0.0012187; *Sall4*+*Oct4*+DsRed versus *Sall4*+*Oct4*+*Mndal*, *p = 0.0134630. **g.** The expression levels for the representative motif-related genes in Fig.5b. The TPM value of genes from RNA-seq data are shown. Data are mean ± SD. n = 2 samples for each time point from 2 independent experiments. DsRed, DsRed system. OCT4, OCT4 system. SALL4, SALL4 system. OCT4+SALL4, OCT4+SALL4 system.

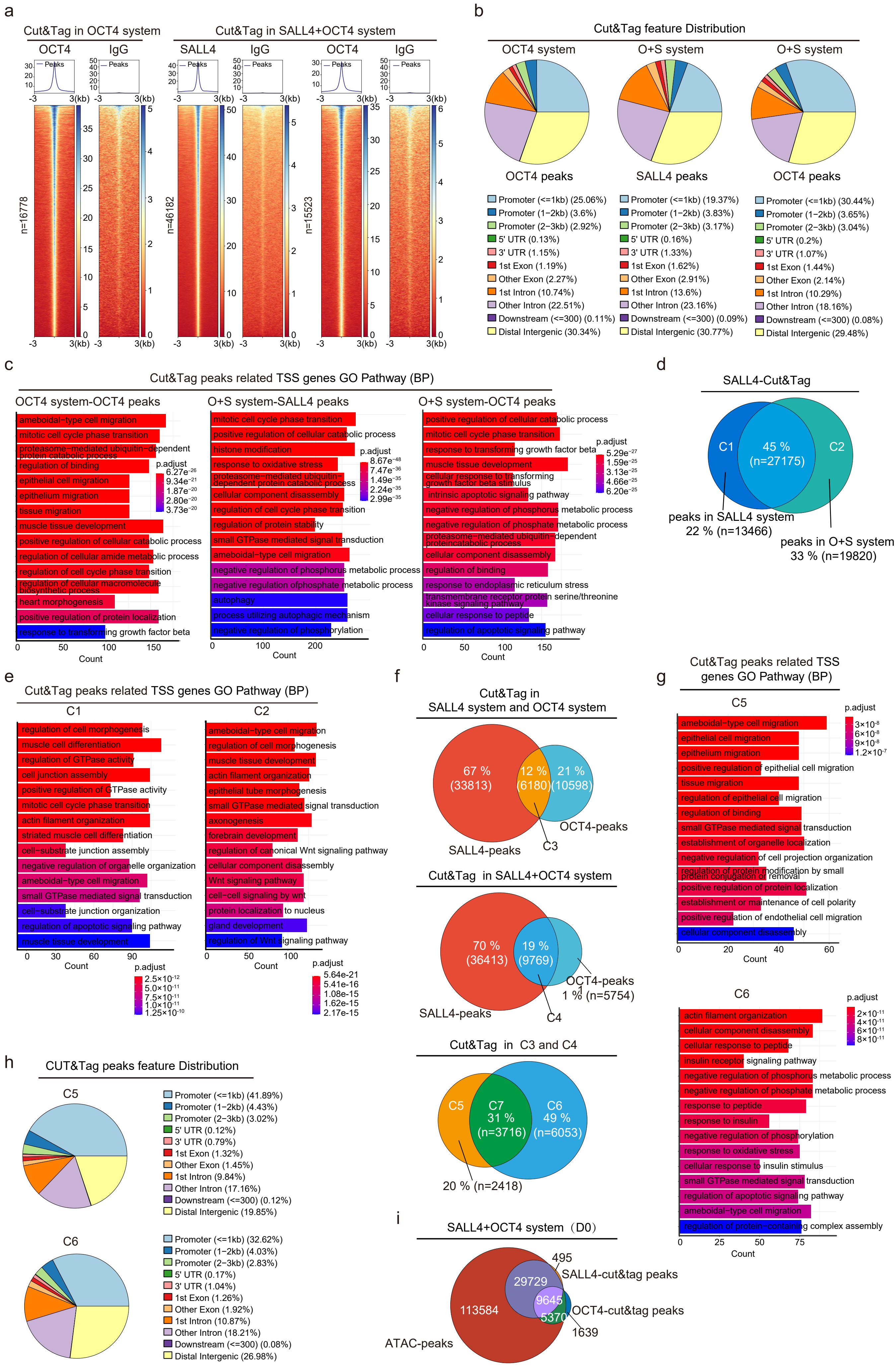
a
Cut&Tag in OCT4 system
Cut&Tag in SALL4+OCT4 system
OCT4
IgG
SALL4
IgG
OCT4
IgG
Peaks
n=16778
n=46182
n=15523
-3
3(kb)
b
Cut&Tag feature Distribution
OCT4 system
O+S system
O+S system
OCT4 peaks
SALL4 peaks
OCT4 peaks
Promoter (<=1kb) (25.06%)
Promoter (1−2kb) (3.6%)
Promoter (2−3kb) (2.92%)
5' UTR (0.13%)
3' UTR (1.15%)
1st Exon (1.19%)
Other Exon (2.27%)
1st Intron (10.74%)
Other Intron (22.51%)
Downstream (<=300) (0.11%)
Distal Intergenic (30.34%)
Promoter (<=1kb) (19.37%)
Promoter (1−2kb) (3.83%)
Promoter (2−3kb) (3.17%)
5' UTR (0.16%)
3' UTR (1.33%)
1st Exon (1.62%)
Other Exon (2.91%)
1st Intron (13.6%)
Other Intron (23.16%)
Downstream (<=300) (0.09%)
Distal Intergenic (30.77%)
Promoter (<=1kb) (30.44%)
Promoter (1−2kb) (3.65%)
Promoter (2−3kb) (3.04%)
5' UTR (0.2%)
3' UTR (1.07%)
1st Exon (1.44%)
Other Exon (2.14%)
1st Intron (10.29%)
Other Intron (18.16%)
Downstream (<=300) (0.08%)
Distal Intergenic (29.48%)
c
Cut&Tag peaks related TSS genes GO Pathway (BP)
OCT4 system-OCT4 peaks
ameboidal−type cell migration
mitotic cell cycle phase transition
proteasome−mediated ubiquitin−dependent protein catabolic process
regulation of binding
epithelial cell migration
epithelium migration
tissue migration
muscle tissue development
positive regulation of cellular catabolic process
regulation of cellular amide metabolic process
regulation of cell cycle phase transition
regulation of cellular macromolecule biosynthetic process
heart morphogenesis
positive regulation of protein localization
response to transforming growth factor beta
p.adjust
Count
O+S system-SALL4 peaks
mitotic cell cycle phase transition
positive regulation of cellular catabolic process
histone modification
response to oxidative stress
proteasome−mediated ubiquitin−dependent protein catabolic process
cellular component disassembly
regulation of cell cycle phase transition
regulation of protein stability
small GTPase mediated signal transduction
ameboidal−type cell migration
negative regulation of phosphorus metabolic process
negative regulation ofphosphate metabolic process
autophagy
process utilizing autophagic mechanism
negative regulation of phosphorylation
O+S system-OCT4 peaks
positive regulation of cellular catabolic process
mitotic cell cycle phase transition
response to transforming growth factor beta
muscle tissue development
cellular response to transforming growth factor beta stimulus
intrinsic apoptotic signaling pathway
negative regulation of phosphorus metabolic process
negative regulation of phosphate metabolic process
proteasome−mediated ubiquitin−dependent proteincatabolic process
cellular component disassembly
regulation of binding
response to endoplasmic reticulum stress
transmembrane receptor protein serine/threonine kinase signaling pathway
cellular response to peptide
regulation of apoptotic signaling pathway
d
SALL4-Cut&Tag
C1
C2
45 %
(n=27175)
peaks in SALL4 system
22 % (n=13466)
peaks in O+S system
33 % (n=19820)
e
Cut&Tag peaks related TSS genes GO Pathway (BP)
C1
regulation of cell morphogenesis
muscle cell differentiation
regulation of GTPase activity
cell junction assembly
positive regulation of GTPase activity
mitotic cell cycle phase transition
actin filament organization
striated muscle cell differentiation
cell−substrate junction assembly
negative regulation of organelle organization
ameboidal−type cell migration
small GTPase mediated signal transduction
cell−substrate junction organization
regulation of apoptotic signaling pathway
muscle tissue development
C2
ameboidal−type cell migration
regulation of cell morphogenesis
muscle tissue development
actin filament organization
epithelial tube morphogenesis
small GTPase mediated signal transduction
axonogenesis
forebrain development
regulation of canonical Wnt signaling pathway
cellular component disassembly
Wnt signaling pathway
cell−cell signaling by wnt
protein localization to nucleus
gland development
regulation of Wnt signaling pathway
f
Cut&Tag in SALL4 system and OCT4 system
67 % (33813)
12 % (6180)
21 % (10598)
SALL4-peaks
OCT4-peaks
C3
Cut&Tag in SALL4+OCT4 system
70 % (36413)
19 % (9769)
OCT4-peaks
1 % (n=5754)
SALL4-peaks
C4
Cut&Tag in C3 and C4
C5
C7
31 %
(n=3716)
C6
49 %
(n=6053)
20 % (n=2418)
g
Cut&Tag peaks related TSS genes GO Pathway (BP)
C5
ameboidal−type cell migration
epithelial cell migration
epithelium migration
positive regulation of epithelial cell migration
tissue migration
regulation of epithelial cell migration
regulation of binding
small GTPase mediated signal transduction
establishment of organelle localization
negative regulation of cell projection organization
regulation of protein modification by small protein conjugation or removal
positive regulation of protein localization
establishment or maintenance of cell polarity
positive regulation of endothelial cell migration
cellular component disassembly
C6
actin filament organization
cellular component disassembly
cellular response to peptide
insulin receptor signaling pathway
negative regulation of phosphorus metabolic process
negative regulation of phosphate metabolic process
response to peptide
response to insulin
negative regulation of phosphorylation
response to oxidative stress
cellular response to insulin stimulus
small GTPase mediated signal transduction
regulation of apoptotic signaling pathway
ameboidal−type cell migration
regulation of protein−containing complex assembly
h
CUT&Tag peaks feature Distribution
C5
Promoter (<=1kb) (41.89%)
Promoter (1−2kb) (4.43%)
Promoter (2−3kb) (3.02%)
5' UTR (0.12%)
3' UTR (0.79%)
1st Exon (1.32%)
Other Exon (1.45%)
1st Intron (9.84%)
Other Intron (17.16%)
Downstream (<=300) (0.12%)
Distal Intergenic (19.85%)
C6
Promoter (<=1kb) (32.62%)
Promoter (1−2kb) (4.03%)
Promoter (2−3kb) (2.83%)
5' UTR (0.17%)
3' UTR (1.04%)
1st Exon (1.26%)
Other Exon (1.92%)
1st Intron (10.87%)
Other Intron (18.21%)
Downstream (<=300) (0.08%)
Distal Intergenic (26.98%)
i
SALL4+OCT4 system（D0）
495
SALL4-cut&tag peaks
29729
9645
113584
5370
OCT4-cut&tag peaks
1639
ATAC-peaks

Supplementary Fig.14 Cut&Tag analysis of SALL4/OCT4 binding site during O+S-mediated iPSCs reprogramming

a. Heatmap of Cut&Tag data at D0 from IgG, OCT4 and SALL4, respectively, showing all binding peaks centred on the peak region within a 3 kb window around the peak. **b.** Genome distribution of the location for SALL4 or OCT4-occupied peaks relative to the nearest annotated gene. O+S, OCT4+SALL4. **c.** GO biological processes analysis for genes near the OCT4-TSS loci binding peaks in OCT4 system (left). GO biological processes analysis for genes near the SALL4 (middle) or OCT4 (right)-TSS loci binding peaks in OCT4+SALL4 system. Statistical analysis for GO was performed using Fisher's exact test, the adjust p value for each term are shown. O+S, OCT4+SALL4. **d.** Venn diagrams shows the overlapping numbers of SALL4 Cut&Tag peaks between SALL4 system and O+S system at day0. O+S, OCT4+SALL4. **e.** Left, GO analysis for genes annotated by Cut&Tag peaks (C1) in Supplementary Fig.14d. Right, GO analysis for genes annotated by Cut&Tag peaks (C2) in Supplementary Fig.14d. Statistical analysis for GO was performed using Fisher's exact test, the adjust p value for each term are shown. **f.** Top, Venn diagrams shows the overlapping numbers between SALL4 binding peaks and OCT4 binding peaks in SALL4 system and OCT4 system. Middle, Venn diagrams shows the overlapping numbers between SALL4 binding peaks and OCT4 binding peaks in O+S system. Bottom, Venn diagrams shows the changes of common binding peaks between the predicted OCT4 and SALL4 common binding peaks (C3 cluster) and the real OCT4 and SALL4 common binding peaks (C4 cluster). **g.** Top, GO analysis for genes annotated by Cut&Tag peaks (C5) in Supplementary Fig.14f. Bottom, GO analysis for genes annotated by Cut&Tag peaks (C6) in Supplementary Fig.14f. Statistical analysis for GO was performed using Fisher's exact test, the adjust p value for each term are shown. **h.** Top, Genome distribution of the location for SALL4 and OCT4-common occupied peaks (C5) in Supplementary Fig.14f relative to the nearest annotated gene. Bottom, Genome distribution of the location for SALL4 and OCT4-common occupied peaks (C6) in Supplementary Fig.14f relative to the nearest annotated gen**e. i.** Venn diagrams shows the overlapping numbers between SALL4 Cut&Tag peaks, OCT4 Cut&Tag peaks and ATAC-seq peaks in O+S system at day0.

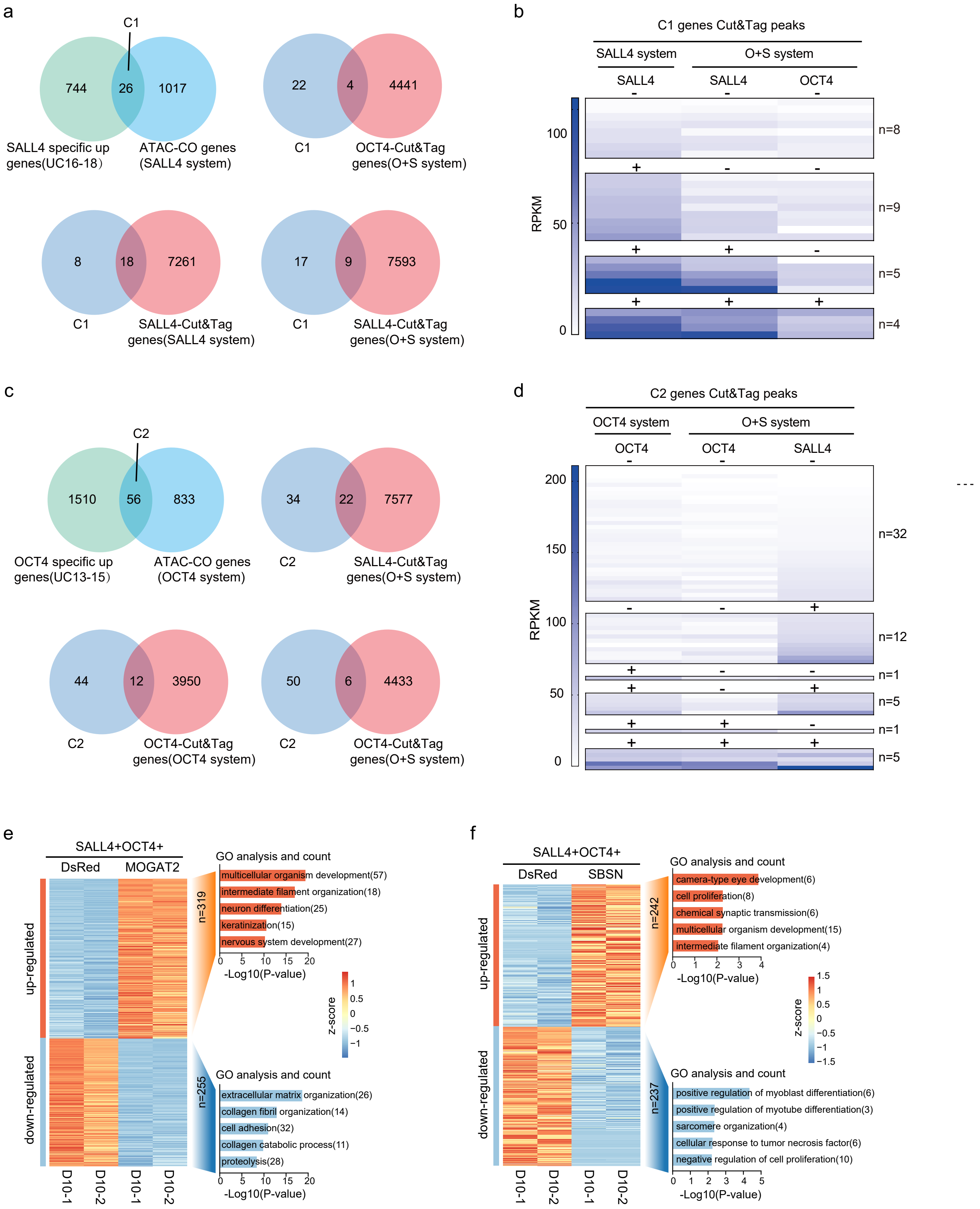
a
C1
744
26
1017
SALL4 specific up genes(UC16-18）
ATAC-CO genes (SALL4 system)
22
4
4441
C1
OCT4-Cut&Tag genes(O+S system)
8
18
7261
C1
SALL4-Cut&Tag genes(SALL4 system)
17
9
7593
C1
SALL4-Cut&Tag genes(O+S system)
b
C1 genes Cut&Tag peaks
SALL4 system
O+S system
SALL4
SALL4
OCT4
RPKM
n=8
n=9
n=5
n=4
c
C2
1510
56
833
OCT4 specific up genes(UC13-15）
ATAC-CO genes (OCT4 system)
34
22
7577
C2
SALL4-Cut&Tag genes(O+S system)
44
12
3950
C2
OCT4-Cut&Tag genes(OCT4 system)
50
6
4433
C2
OCT4-Cut&Tag genes(O+S system)
d
C2 genes Cut&Tag peaks
OCT4 system
O+S system
OCT4
OCT4
SALL4
RPKM
n=32
n=12
n=1
n=5
n=1
n=5
e
SALL4+OCT4+
DsRed
MOGAT2
up-regulated
down-regulated
D10-1
D10-2
D10-1
D10-2
n=319
n=255
GO analysis and count
multicellular organism development(57)
intermediate filament organization(18)
neuron differentiation(25)
keratinization(15)
nervous system development(27)
-Log10(P-value)
z-score
GO analysis and count
extracellular matrix organization(26)
collagen fibril organization(14)
cell adhesion(32)
collagen catabolic process(11)
proteolysis(28)
-Log10(P-value)
f
SALL4+OCT4+
DsRed
SBSN
up-regulated
down-regulated
D10-1
D10-2
D10-1
D10-2
n=242
n=237
GO analysis and count
camera-type eye development(6)
cell proliferation(8)
chemical synaptic transmission(6)
multicellular organism development(15)
intermediate filament organization(4)
-Log10(P-value)
z-score
GO analysis and count
positive regulation of myoblast differentiation(6)
positive regulation of myotube differentiation(3)
sarcomere organization(4)
cellular response to tumor necrosis factor(6)
negative regulation of cell proliferation(10)
-Log10(P-value)

Supplementary Fig.15 The functions of SALL4-downstream genes in reprogramming

a. Venn diagrams shows the overlapping numbers(C1 subgroup) between SALL4 specific up genes(Fig.4e, UC16-18) and ATAC-CO genes in SALL4 system. The overlapping numbers from the comparison of C1 subgroup genes with the SALL4 or OCT4-binding peaks related genes in SALL4 and O+S system are shown in figur**e.** O+S, OCT4+SALL4. **b.** Heatmap of SALL4 or OCT4-binding peaks enrichment on C1 genes (Supplementary Fig.15a) in SALL4 system and O+S system, respectively. O+S, OCT4+SALL4. +, genes with binding peaks. -, genes without binding peaks. **c.** Venn diagrams shows the overlapping numbers (C2 subgroup) between OCT4 specific up genes (Fig.4e, UC13-15) and ATAC-CO genes in OCT4 system. The overlapping numbers from the comparison of C2 subgroup genes with the SALL4 or OCT4-binding peaks related genes in OCT4 and O+S system are shown in figur**e.** O+S, OCT4+SALL4. **d.** Heatmap of SALL4 or OCT4-binding peaks enrichment on C2 genes (Supplementary Fig.15c) in OCT4 system and O+S system, respectively. O+S, OCT4+SALL4. +, genes with binding peaks. -, genes without binding peaks. **e-f.** Heatmap of differential expression gene analysis for day10 RNA-seq data from SALL4+OCT4+DsRed, SALL4+OCT4+MOGAT2 and SALL4+OCT4+SBSN systems. The 4 subgroups were based on the fold change of gene expression between DsRed and MOGAT2/SBSN. GO analysis for each subgroup are shown. Statistical analysis for GO was performed using Fisher's exact test, the -Log10(p value) for each term are shown.

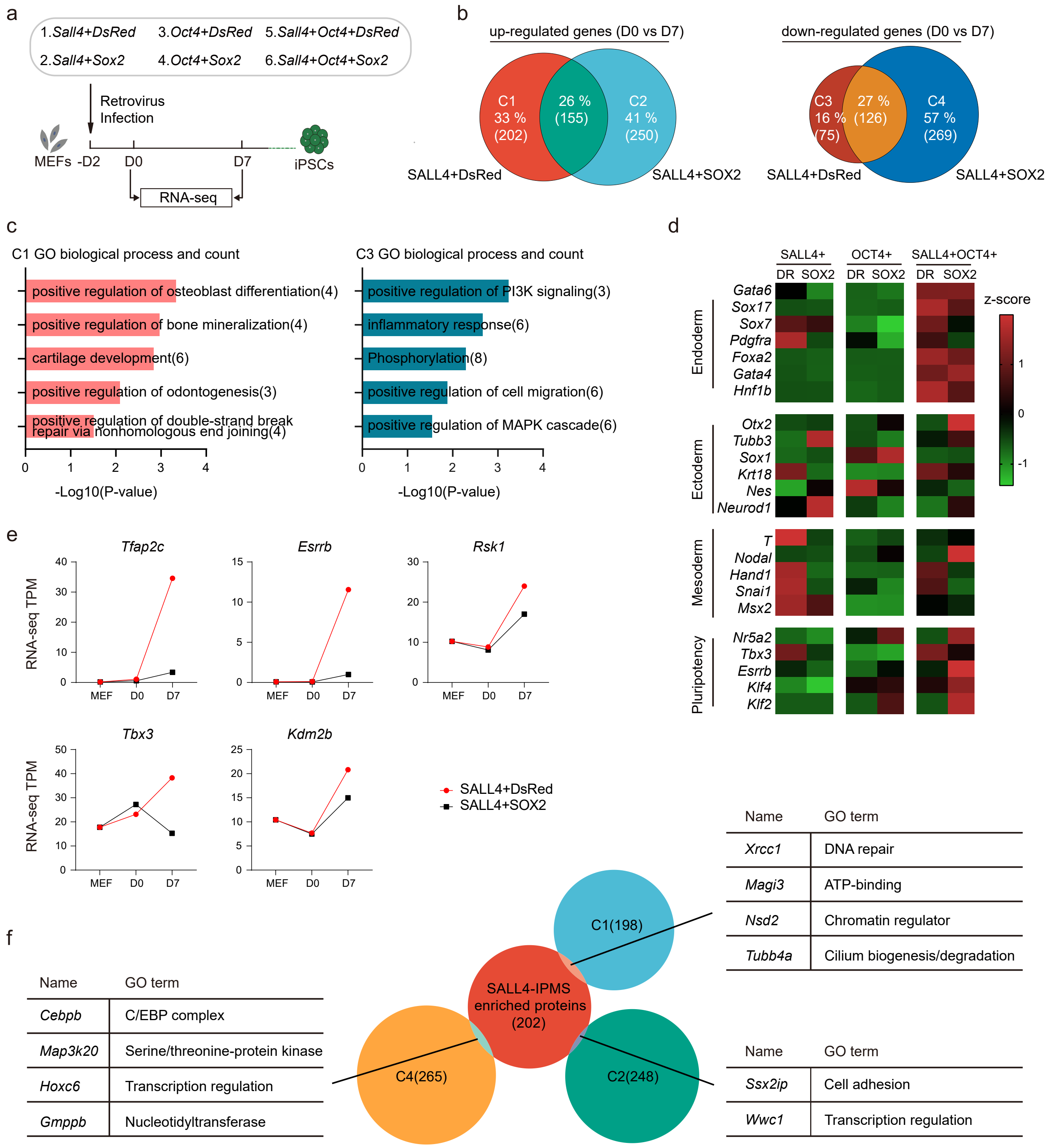
a
1.Sall4+DsRed
3.Oct4+DsRed
5.Sall4+Oct4+DsRed
2.Sall4+Sox2
4.Oct4+Sox2
6.Sall4+Oct4+Sox2
Retrovirus Infection
MEFs -D2
D0
D7
iPSCs
RNA-seq
b
up-regulated genes (D0 vs D7)
C1 33 % (202)
26 % (155)
C2 41 % (250)
SALL4+DsRed
SALL4+SOX2
down-regulated genes (D0 vs D7)
C3 16 % (75)
27 % (126)
C4 57 % (269)
SALL4+DsRed
SALL4+SOX2
c
C1 GO biological process and count
positive regulation of osteoblast differentiation(4)
positive regulation of bone mineralization(4)
cartilage development(6)
positive regulation of odontogenesis(3)
positive regulation of double-strand break repair via nonhomologous end joining(4)
0 1 2 3 4
-Log10(P-value)
C3 GO biological process and count
positive regulation of PI3K signaling(3)
inflammatory response(6)
Phosphorylation(8)
positive regulation of cell migration(6)
positive regulation of MAPK cascade(6)
0 1 2 3 4
-Log10(P-value)
d
SALL4+
OCT4+
SALL4+OCT4+
DR SOX2
DR SOX2
DR SOX2
Endoderm
Gata6
Sox17
Sox7
Pdgfra
Foxa2
Gata4
Hnf1b
Ectoderm
Otx2
Tubb3
Sox1
Krt18
Nes
Neurod1
Mesoderm
T
Nodal
Hand1
Snai1
Msx2
Pluripotency
Nr5a2
Tbx3
Esrrb
Klf4
Klf2
z-score
1
0
-1
e
Tfap2c
Esrrb
Rsk1
Tbx3
Kdm2b
RNA-seq TPM
MEF D0 D7
SALL4+DsRed
SALL4+SOX2
f
C1(198)
SALL4-IPMS enriched proteins (202)
C4(265)
C2(248)
Name GO term
Xrcc1 DNA repair
Magi3 ATP-binding
Nsd2 Chromatin regulator
Tubb4a Cilium biogenesis/degradation
Name GO term
Cebpb C/EBP complex
Map3k20 Serine/threonine-protein kinase
Hoxc6 Transcription regulation
Gmppb Nucleotidyltransferase
Name GO term
Ssx2ip Cell adhesion
Wwc1 Transcription regulation

Supplementary Fig.16 SOX2 have a opposite effect in SALL4-reprogramming and OCT4-reprogramming

a. Diagram for RNA-seq data collecting during *Sox2* related reprogramming process. **b.** Venn diagrams shows the number of differential expression genes in *Sox2* related reprogramming process. **c.** Left, GO analysis for genes specific-upregulated in SALL4+DsRed group (C1) in Supplementary Fig.16b**.** Right, GO analysis for genes specific-downregulated in SALL4+DsRed group (C3) in Supplementary Fig.16b. Statistical analysis for GO was performed using Fisher's exact test, the -Log10(p value) for each term are shown. **d.** Heatmap showing expression of master regulator genes for each of the three primary germ layers at day7. DR, DsRed. **e.** RNA-seq data shows the expression of representative reprogramming promoting genes in SALL4+DsRed and SALL4+SOX2 condition. n = 1 samples for each time point from 1 independent experiments. **f.** Venn diagrams shows the overlapping genes between SALL4 enriched proteins and SOX2-regulated differential expression genes in Supplementary Fig.16b.

Supplementary Table 1. List of primer sequences for PCR, qRT-PCR and shRNA.

qPCR Primer (5'-3')	
Endo-Oct4-F	TAGGTGAGCCGTCTTTCCAC
Endo-Oct4-R	GCTTAGCCAGGTTCGAGGAT
Endo-Sox2-F	AGGGCTGGGAGAAAGAAGAG
Endo-Sox2-R	CCGCGATTGTTGTGATTAGT
Endo-Sall4-F	CGAAGGGGGCTAAAATTTCCCA
Endo-Sall4-R	TGGCCCTCCTCCCAGTTGAT
Endo-Nanog-F	AAATCCCTTCCCTCGCCATC
Endo-Nanog-R	GCGTTCCCAGAATTCGATGC
Endo-Rex1-F	CCCTCGACAGACTGACCCTAA
Endo-Rex1-R	TCGGGGCTAATCTCACTTTCAT
Endo-Esrrb-F	GGGATGCTGAAGGAAGGTGT
Endo-Esrrb-R	TTAGCAGGTGGGGAAATCG
Endo-Nr5a2-F	TCTGAGCCATGTAGCCTTGC
Endo-Nr5a2-R	GGAAAGTGACCATAGGGTTGGTA
Exo-PF	GGGTGGACCATCCTCTAGAC
Exo-Sall4-R	GCAGTTCCCAGGGGAGTTCAC
Exo-Oct4-R	AGCTGATTGGCGATGTGAGT
PCR Primer (5'-3')	
Sall4-PF	GGGTGGACCATCCTCTAGAC
Sall4-PR	GATGGCTGGGTAGCCTCTGTC
Oct4-PF	TTAAGGATCCATGGCTGGACACCTG
Oct4-PR	TGGTACGCGTGTTTGAATGCATGGG
shRNA (5'-3')	
shTfap2c-1	GCACTTGCTCCTACACGATCA
shTfap2c-2	GCGGTGGCTGACTATTTAACG
shEsrrb-1	GATTCGATGTACATTGAGA
shEsrrb-2	GGATGGAGATTCTCATCTTGG
shRsk1-1	GCTCTATCTTATTCTGGACTT
shRsk1-2	CTACATACTCTGCTCTCAATA
