# Supplementary material for: Reconstitution of pluripotency from mouse fibroblast through Sall4 overexpression
Source: Nat Commun. 2024 Dec 30;15:10787. doi: 10.1038/s41467-024-54924-5 (PMC11686038; doi:10.1038/s41467-024-54924-5)
Supplement: Supplementary file 3 — Reporting Summary [file 41467_2024_54924_MOESM3_ESM.pdf]

nature portfolio

Corresponding author(s): NCOMMS-23-27401D

Last updated by author(s): Nov 7, 2024

Reporting Summary

Nature Portfolio wishes to improve the reproducibility of the work that we publish. This form provides structure for consistency and transparency in reporting. For further information on Nature Portfolio policies, see our Editorial Policies and the Editorial Policy Checklist.

Statistics

For all statistical analyses, confirm that the following items are present in the figure legend, table legend, main text, or Methods section.

n/a	Confirmed	
☐	☒	The exact sample size (*n*) for each experimental group/condition, given as a discrete number and unit of measurement
☐	☒	A statement on whether measurements were taken from distinct samples or whether the same sample was measured repeatedly
☐	☒	The statistical test(s) used AND whether they are one- or two-sided<br>*Only common tests should be described solely by name; describe more complex techniques in the Methods section.*
☒	☐	A description of all covariates tested
☐	☒	A description of any assumptions or corrections, such as tests of normality and adjustment for multiple comparisons
☐	☒	A full description of the statistical parameters including central tendency (e.g. means) or other basic estimates (e.g. regression coefficient) AND variation (e.g. standard deviation) or associated estimates of uncertainty (e.g. confidence intervals)
☐	☒	For null hypothesis testing, the test statistic (e.g. *F*, *t*, *r*) with confidence intervals, effect sizes, degrees of freedom and *P* value noted<br>*Give P values as exact values whenever suitable.*
☒	☐	For Bayesian analysis, information on the choice of priors and Markov chain Monte Carlo settings
☒	☐	For hierarchical and complex designs, identification of the appropriate level for tests and full reporting of outcomes
☒	☐	Estimates of effect sizes (e.g. Cohen's *d*, Pearson's *r*), indicating how they were calculated

Our web collection on statistics for biologists contains articles on many of the points above.

Software and code

Policy information about availability of computer code

Data collection	trim_galore(v0.6.7), hisat2(v2.2.1), samtools(1.6), featureCounts(v2.0.1), DEseq2(1.38.1), bowtie2(2.5.0), sambamba(v0.8.2), bedtools(v2.30.0), deeptoos(v3.5.1), macs2(2.2.7.1),DEP2, Monocle (v.2.12)
Data analysis	ATAC-seq,RNA-seq, scRNA-seq, Cut&Tag and IP-MS data were analyzed following the softeare documentations, no special changes. Microsoft Excel and GraphPad Prism 8 was used to analyze statistical data and draw graphs. ImageJ 1.53 was used to count Oct4-GFP+colonies.

For manuscripts utilizing custom algorithms or software that are central to the research but not yet described in published literature, software must be made available to editors and reviewers. We strongly encourage code deposition in a community repository (e.g. GitHub). See the Nature Portfolio guidelines for submitting code & software for further information.

Data

Policy information about availability of data

All manuscripts must include a data availability statement. This statement should provide the following information, where applicable:

- Accession codes, unique identifiers, or web links for publicly available datasets
- A description of any restrictions on data availability
- For clinical datasets or third party data, please ensure that the statement adheres to our policy

ATAC-seq, RNA-seq ,scRNA-seq and Cut&Tag datas support the findings of this study are available under Genome Sequence Archive (https://ngdc.cncb.ac.cn/?

lang=en) with Bioproject ID PRJCA017942. The mass spectrometry proteomics data have been deposited to the ProteomeXchange Consortium (https://proteomecentral.proteomexchange.org) via the iProX partner repository with the dataset identifier PXD057516. Source data are provided with this paper.

Research involving human participants, their data, or biological material

Policy information about studies with human participants or human data. See also policy information about sex, gender (identity/presentation), and sexual orientation and race, ethnicity and racism.

Reporting on sex and gender	This study did not involve that.
Reporting on race, ethnicity, or other socially relevant groupings	This study did not involve that.
Population characteristics	This study did not involve that.
Recruitment	This study did not involve that.
Ethics oversight	This study did not involve that.

Note that full information on the approval of the study protocol must also be provided in the manuscript.

Field-specific reporting

Please select the one below that is the best fit for your research. If you are not sure, read the appropriate sections before making your selection.

☒ Life sciences ☐ Behavioural & social sciences ☐ Ecological, evolutionary & environmental sciences

For a reference copy of the document with all sections, see nature.com/documents/nr-reporting-summary-flat.pdf

Life sciences study design

All studies must disclose on these points even when the disclosure is negative.

Sample size	No sample size calculation was performed. Sample size was chosen based on our experience and experiments from published literature.
Data exclusions	No data were exclude from the analysis.
Replication	For all the main figures from which the significance were assessed, three biological replication experiments were performed. The biological or technical replicates were present in relevant figure legends. The experimental findings were reliably reproduced.
Randomization	For chimera experiments, mice and embryos were selected at random for blastocyst injection.
Blinding	The investigates were blinded to group allocation during data collection and analysis.

Reporting for specific materials, systems and methods

We require information from authors about some types of materials, experimental systems and methods used in many studies. Here, indicate whether each material, system or method listed is relevant to your study. If you are not sure if a list item applies to your research, read the appropriate section before selecting a response.

Materials & experimental systems

n/a	Involved in the study	
☐	☒	Antibodies
☐	☒	Eukaryotic cell lines
☒	☐	Palaeontology and archaeology
☐	☒	Animals and other organisms
☒	☐	Clinical data
☒	☐	Dual use research of concern
☒	☐	Plants

Methods

n/a	Involved in the study	
☐	☒	ChIP-seq
☐	☒	Flow cytometry
☒	☐	MRI-based neuroimaging

Antibodies

Antibodies used	Rabbit anti-Oct4(Abcam, ab19857, 1:1000 for IF); Rabbit anti-Sox2(Cell Signaling Ttchnology, 3579, 1:200 for IF); Rabbit anti-

Antibodies used	Nanog(Cell Signaling Ttchnology, 8822S, 1:1000 for IF); Mouse anti-Flag tag(Sigma, F1804, 5ug for Cut&Tag); Anti-mouse IgG(Abcam, ab18413, 5ug for Cut&Tag);Mouse anti-BATF(sc-100974, Santa Cruz, 1:500 for western blot);Mouse anti-GAPDH (60004-1-lg, ProteinTech, 1:1000 for western blot)
Validation	Antibodies were validated according to manufacturer's instruction, and were used for similar experiments by other researchers.

Eukaryotic cell lines

Policy information about cell lines and Sex and Gender in Research

Cell line source(s)	MEF and ESC derived from mouse embryos were generated from 129S4/SvJaeJ female mice which were crossed with male OCT4-GFP transgenic allele-carrying mice(CBA/CaJ 3 C57BL/6J).TTF were generated from 6-8 week-old male OCT4-GFP transgenic allele-carrying mice(CBA/CaJ 3 C57BL/6J).
Authentication	cell lines were not authenticated.
Mycoplasma contamination	All the cell lines have been confirmed as Mycoplasma contamination free with the kit from lonza(LT07-318).
Commonly misidentified lines (See ICLAC register)	No commonly misidentified cell lines were used.

Animals and other research organisms

Policy information about studies involving animals; ARRIVE guidelines recommended for reporting animal research, and Sex and Gender in Research

Laboratory animals	The mice used in this study were 8-week-old male Oct4-GFP transgenetic allele carrying mice(CBA/CaJ 3 C57BL/6J), female 129S4/SvJaeJ mice, NCG and ICR mice
Wild animals	This study did not involve wild animals.
Reporting on sex	This information in this study has not been collected.
Field-collected samples	This study did not involve samples collected from the field.
Ethics oversight	All experiments related to animal were performed on the basis of the Animal Protection Guidelines of Guangzhou Institutes of Biomedicine and Health(GIBH), and approved by the Committee on the Ethics of Animal Experiments at GIBH.

Note that full information on the approval of the study protocol must also be provided in the manuscript.

Plants

Seed stocks	This study did not involve that.
Novel plant genotypes	This study did not involve that.
Authentication	This study did not involve that.

ChIP-seq

Data deposition

☒ Confirm that both raw and final processed data have been deposited in a public database such as GEO.

☒ Confirm that you have deposited or provided access to graph files (e.g. BED files) for the called peaks.

Data access links *May remain private before publication.*	https://ngdc.cncb.ac.cn/bioproject/browse/PRJCA017942
Files in database submission	D0-S4-Cut&Tag; D4-S4-Cut&Tag; D7-S4-Cut&Tag; D10-S4-Cut&Tag; D0-IgG-Cut&Tag; D4-IgG-Cut&Tag; D7-IgG-Cut&Tag; D10-IgG-Cut&Tag;D0-O4-Cut&Tag; D0-O4 in OS-Cut&Tag; D0-S4 in OS-Cut&Tag; D0-S4-ZFC1-Cut&Tag; D0-S4-ZFC2-Cut&Tag; D0-S4-ZFC3-Cut&Tag; D0-IgG in OS-Cut&Tag; D0-S4-N12-Cut&Tag; D0-S4-ZFC1-IgG-Cut&Tag; D0-S4-ZFC2-IgG-Cut&Tag; D0-S4-ZFC3-IgG-Cut&Tag; D0-S4-N12-IgG-Cut&Tag.

Genome browser session
(e.g. UCSC)

No public available genome browser session

Methodology

Replicates	There were no biological replicates. The ChIP-seq experiment was performed once.
Sequencing depth	About 30M raw reads for each sample
Antibodies	Mouse anti-Flag tag(Sigma, F1804, 5ug for Cut&Tag); Anti-mouse IgG(Abcam, ab18413, 5ug for Cut&Tag)
Peak calling parameters	Peaks calling parameters:Peaks were called by MACS2 with parameters with"-g mm -f BAMPE -q 0.1 --keep-dup all"
Data quality	For Sall4 Cut&tag data, we detect significant 39993, 22168, 12931, 64peaks at day 0,4,7,10, respectively. For Oct4 Cut&tag data,we detect significant 16778 peaks at day 0. For Oct4 in OS Cut&tag data,we detect significant 15523 peaks at day 0. For Sall4 in OS Cut&tag data,we detect significant 46182 peaks at day 0. For Sall4-ZFC1/2/3 Cut&tag data, we detect significant 59497, 45683, 66830 peaks at day 0,respectively. For Sall4-N12 Cut&tag data, we detect significant 62633 peaks at day 0.
Software	Sequencing data was mapped to the mm10 mouse genome assembly using bowtie2 with the following options: --end-to-end --very-sensitive --no-mixed --no-discordant. Duplicated PCR reads were removed using sambamba with the option --overflow-list-size 600000. BigWig files were generated from bam files using bedtools and bamCoverage, with normalization based on RPKM (Reads Per Kilobase per Million mapped reads). Peaks were called using MACS2.

Flow Cytometry

Plots

Confirm that:

☒ The axis labels state the marker and fluorochrome used (e.g. CD4-FITC).

☒ The axis scales are clearly visible. Include numbers along axes only for bottom left plot of group (a 'group' is an analysis of identical markers).

☒ All plots are contour plots with outliers or pseudocolor plots.

☒ A numerical value for number of cells or percentage (with statistics) is provided.

Methodology

Sample preparation	Cells were dissociated into single cell using 0.25% trypsin and collected by centrifugation. The cell pellet was resuspended with PBS. Added the Thy1.2 antibody(#48-0902-82, invitrogen, 1:200) and Epcam antibody(#12-5791-81,invitrogen, 1:200) in cell suspension and incubated for 30 min. Washed twice with PBS followed by filtration using a cell strainer (BD Biosciences) to remove large clumps of cells.
Instrument	BD FACSAria?
Software	Data analysis was done using FlowJo (V10)software.
Cell population abundance	A minimum of 10000 cells were counted per sample analyzed. Notably, a total of 1000 cells expressing GFP were isolated from the SALL4-D10 samples.
Gating strategy	We set the preliminary FSC/SSC gates based on cell size and complexity to remove debris and other events of non-interest, then we set the gate for the thy1 negative /epcam positive populations based on negative conteol(samples with out antibody staining).

☒ Tick this box to confirm that a figure exemplifying the gating strategy is provided in the Supplementary Information.
