# Supplementary material for: Reconstitution of pluripotency from mouse fibroblast through Sall4 overexpression
Source: Nat Commun. 2024 Dec 30;15:10787. doi: 10.1038/s41467-024-54924-5 (PMC11686038; doi:10.1038/s41467-024-54924-5)
Supplement: Supplementary file 4 — Source Data [file 41467_2024_54924_MOESM4_ESM.zip › source data/main figures/figure2/e/D0_S4.rmdup.sort.bed.motif/homerResults.html]

rmdup\_vs\_igg\_motifs/D0\_S4.rmdup.sort.bed.motif/ - Homer de novo Motif Results


# Homer *de novo* Motif Results (rmdup\_vs\_igg\_motifs/D0\_S4.rmdup.sort.bed.motif/)

Known Motif Enrichment Results  
Gene Ontology Enrichment Results  
If Homer is having trouble matching a motif to a known motif, try copy/pasting the matrix file into
STAMP  
More information on motif finding results: HOMER
| Description of Results
| Tips
  
Total target sequences = 39986  
Total background sequences = 39953  
\* - possible false positive  

|  |  |  |  |  |  |  |  |  |
| --- | --- | --- | --- | --- | --- | --- | --- | --- |
| Rank | Motif | P-value | log P-pvalue | % of Targets | % of Background | STD(Bg STD) | Best Match/Details | Motif File |
| 1 | C A T G T A G C C A G T T C G A C G A T A C T G C G T A A T G C G C A T T G A C G C T A A C T G | 1e-2950 | -6.793e+03 | 22.43% | 5.27% | 54.4bp (60.7bp) | JunB(bZIP)/DendriticCells-Junb-ChIP-Seq(GSE36099)/Homer(0.987) More Information | Similar Motifs Found | motif file (matrix) |
| 2 | T C G A C T A G T G A C C G T A G C A T C G A T A T G C G A T C C G T A C A T G | 1e-295 | -6.804e+02 | 11.55% | 6.55% | 55.7bp (57.7bp) | TEAD3/MA0808.1/Jaspar(0.973) More Information | Similar Motifs Found | motif file (matrix) |
| 3 | C G A T T G C A C T G A A G T C A T G C C G T A T G A C C G T A | 1e-272 | -6.282e+02 | 20.25% | 13.80% | 56.1bp (59.2bp) | RUNX2(Runt)/PCa-RUNX2-ChIP-Seq(GSE33889)/Homer(0.938) More Information | Similar Motifs Found | motif file (matrix) |
| 4 | A G T C A G C T A G C T T A C G A T G C A G T C C G T A T C A G C T G A T C A G | 1e-154 | -3.551e+02 | 31.20% | 25.31% | 55.5bp (58.0bp) | NF1-halfsite(CTF)/LNCaP-NF1-ChIP-Seq(Unpublished)/Homer(0.870) More Information | Similar Motifs Found | motif file (matrix) |
| 5 | C T A G T A G C G A C T A C G T C G A T A T C G G A C T T C G A G C T A A G T C | 1e-107 | -2.472e+02 | 12.79% | 9.41% | 55.1bp (59.8bp) | SOX10/MA0442.2/Jaspar(0.755) More Information | Similar Motifs Found | motif file (matrix) |
| 6 | T C A G A T C G T C G A C T G A T G C A G T A C G T C A T A C G A G T C A G T C | 1e-106 | -2.459e+02 | 26.21% | 21.58% | 55.5bp (57.3bp) | NFATC1/MA0624.1/Jaspar(0.841) More Information | Similar Motifs Found | motif file (matrix) |
| 7 | T C G A A C G T C A T G T C G A A T G C C T A G G A C T T G C A | 1e-95 | -2.207e+02 | 10.00% | 7.17% | 56.5bp (60.1bp) | Atf1/MA0604.1/Jaspar(0.978) More Information | Similar Motifs Found | motif file (matrix) |
| 8 | C T A G A G T C G T A C G A T C G T A C C T A G A T G C G A T C A G T C G A C T | 1e-90 | -2.094e+02 | 20.64% | 16.75% | 55.2bp (56.9bp) | KLF1(Zf)/HUDEP2-KLF1-CutnRun(GSE136251)/Homer(0.858) More Information | Similar Motifs Found | motif file (matrix) |
| 9 | T C A G G T C A G C T A T A C G G C T A T C G A G A C T T C A G G A C T A G T C | 1e-79 | -1.821e+02 | 13.37% | 10.38% | 55.7bp (58.9bp) | SOX14/MA1562.1/Jaspar(0.740) More Information | Similar Motifs Found | motif file (matrix) |
| 10 | A T G C C G A T A T C G C A T G C T A G C G A T T A C G C G T A | 1e-75 | -1.740e+02 | 18.54% | 15.14% | 56.5bp (59.0bp) | Znf281/MA1630.1/Jaspar(0.696) More Information | Similar Motifs Found | motif file (matrix) |
| 11 | A G C T C G A T C A T G C T A G G A T C G C A T A T G C T C A G | 1e-68 | -1.570e+02 | 15.16% | 12.20% | 55.8bp (59.9bp) | NFIX/MA0671.1/Jaspar(0.851) More Information | Similar Motifs Found | motif file (matrix) |
| 12 | G A T C C T A G A C T G A T G C G A C T T G C A G C T A C A G T A G C T T G C A | 1e-67 | -1.549e+02 | 20.96% | 17.58% | 55.3bp (59.7bp) | Lhx2(Homeobox)/HFSC-Lhx2-ChIP-Seq(GSE48068)/Homer(0.877) More Information | Similar Motifs Found | motif file (matrix) |
| 13 | A G T C A G C T A T C G C A G T C A G T C G A T G T C A T A G C | 1e-61 | -1.418e+02 | 13.66% | 10.98% | 55.8bp (59.1bp) | Foxf1/MA1606.1/Jaspar(0.941) More Information | Similar Motifs Found | motif file (matrix) |
| 14 | T C G A T C G A C G T A A G T C G C T A A G C T A C G T A G C T A G T C G A T C G C A T A C T G | 1e-55 | -1.276e+02 | 2.76% | 1.66% | 56.3bp (58.3bp) | EWS:ERG-fusion(ETS)/CADO\_ES1-EWS:ERG-ChIP-Seq(SRA014231)/Homer(0.872) More Information | Similar Motifs Found | motif file (matrix) |
| 15 | A C T G A C G T A C T G A C T G A T G C A C G T A C G T A C G T | 1e-55 | -1.269e+02 | 4.39% | 2.97% | 58.8bp (60.4bp) | RUNX1(Runt)/Jurkat-RUNX1-ChIP-Seq(GSE29180)/Homer(0.711) More Information | Similar Motifs Found | motif file (matrix) |
| 16 | C A G T C G A T A C T G G T A C G A C T A C T G C G T A C T G A C G A T A C T G G C T A C G T A | 1e-48 | -1.112e+02 | 0.10% | 0.00% | 48.6bp (47.9bp) | PB0028.1\_Hbp1\_1/Jaspar(0.714) More Information | Similar Motifs Found | motif file (matrix) |
| 17 | T G C A T C G A G A T C T G A C C A T G G T C A T G C A C T G A T C A G C A T G A G C T C G A T | 1e-43 | -1.003e+02 | 0.09% | 0.00% | 56.4bp (0.0bp) | IRF2(IRF)/Erythroblas-IRF2-ChIP-Seq(GSE36985)/Homer(0.691) More Information | Similar Motifs Found | motif file (matrix) |
| 18 | A T G C G A T C C G A T A G T C G A T C G T A C G C A T A G T C G C A T T A C G C T A G T A C G | 1e-40 | -9.270e+01 | 3.52% | 2.42% | 53.7bp (54.8bp) | ZNF263/MA0528.2/Jaspar(0.663) More Information | Similar Motifs Found | motif file (matrix) |
| 19 | G A C T C A G T C T A G T A C G T A G C G C T A T A G C G C T A T A C G C G T A T C A G T A G C | 1e-36 | -8.476e+01 | 3.50% | 2.45% | 54.0bp (59.0bp) | NFIA/MA0670.1/Jaspar(0.680) More Information | Similar Motifs Found | motif file (matrix) |
| 20 | G T C A G A C T G A C T G C T A G T A C G T C A G C T A T G A C C G T A A C G T T A G C G A T C | 1e-35 | -8.098e+01 | 0.50% | 0.18% | 54.4bp (56.2bp) | Chop(bZIP)/MEF-Chop-ChIP-Seq(GSE35681)/Homer(0.775) More Information | Similar Motifs Found | motif file (matrix) |
| 21 | A T G C C A T G A T C G A C G T A C G T A C T G A C T G C G T A C G A T C G T A C G T A A C G T | 1e-31 | -7.266e+01 | 0.09% | 0.01% | 56.3bp (52.2bp) | HLF/MA0043.3/Jaspar(0.752) More Information | Similar Motifs Found | motif file (matrix) |
| 22 | A C T G A C T G C T A G T A G C C T A G A T C G T C G A A C T G C G T A C T G A A C G T C A G T | 1e-31 | -7.218e+01 | 0.07% | 0.00% | 58.4bp (0.0bp) | Sp5(Zf)/mES-Sp5.Flag-ChIP-Seq(GSE72989)/Homer(0.715) More Information | Similar Motifs Found | motif file (matrix) |
| 23 | A G T C A C G T C G T A C G T A A G T C A G T C A C T G C G T A G T A C C G T A | 1e-27 | -6.408e+01 | 0.08% | 0.01% | 61.1bp (8.5bp) | MF0009.1\_TRP(MYB)\_class/Jaspar(0.798) More Information | Similar Motifs Found | motif file (matrix) |
| 24 | G A T C A G C T G T C A G T A C T C A G G C T A A G T C G A C T A T G C G T C A A C G T G T A C | 1e-24 | -5.655e+01 | 0.09% | 0.01% | 51.5bp (19.1bp) | NFE2/MA0841.1/Jaspar(0.715) More Information | Similar Motifs Found | motif file (matrix) |
| 25 | A C G T C A T G A G C T A G T C G T A C C G T A A C G T A G T C A C T G A C T G | 1e-24 | -5.641e+01 | 0.10% | 0.01% | 47.4bp (78.2bp) | PB0189.1\_Tcfap2a\_2/Jaspar(0.661) More Information | Similar Motifs Found | motif file (matrix) |
| 26 | G A C T G A T C T G A C A T G C G C A T A G C T T G A C A C T G | 1e-23 | -5.413e+01 | 2.70% | 1.96% | 55.6bp (60.4bp) | Ebf2/MA1604.1/Jaspar(0.668) More Information | Similar Motifs Found | motif file (matrix) |
| 27 | A G T C A C G T A G T C A C G T A C G T A C T G A C G T C G T A C G T A C G T A | 1e-21 | -5.037e+01 | 0.07% | 0.01% | 56.7bp (32.0bp) | HOXC13/MA0907.1/Jaspar(0.733) More Information | Similar Motifs Found | motif file (matrix) |
| 28 | A G T C A C T G A C T G A C G T A G T C C G T A A C G T A C T G A C T G A C T G | 1e-21 | -5.037e+01 | 0.07% | 0.01% | 56.6bp (48.4bp) | NR2F1/MA0017.2/Jaspar(0.678) More Information | Similar Motifs Found | motif file (matrix) |
| 29 | A G T C A C T G A C T G A C G T A G T C A G T C A C G T A C T G C G T A A C G T A C G T A C T G | 1e-19 | -4.513e+01 | 0.07% | 0.01% | 48.8bp (17.3bp) | Dux/MA0611.1/Jaspar(0.694) More Information | Similar Motifs Found | motif file (matrix) |
| 30 | A C T G A C G T A G T C C G T A C G T A A C G T A C G T A C G T C G T A A C G T | 1e-17 | -3.998e+01 | 0.07% | 0.01% | 47.7bp (29.9bp) | Hnf6b(Homeobox)/LNCaP-Hnf6b-ChIP-Seq(GSE106305)/Homer(0.678) More Information | Similar Motifs Found | motif file (matrix) |
| 31 | A G T C A T G C C T A G G C A T C G T A C T A G C G T A A C G T C G A T A C T G | 1e-16 | -3.894e+01 | 0.08% | 0.01% | 54.5bp (18.7bp) | Dux/MA0611.1/Jaspar(0.649) More Information | Similar Motifs Found | motif file (matrix) |
| 32 | A C T G A C T G A G T C A G T C A C G T A G T C A C G T A C G T C G T A A G T C | 1e-12 | -2.917e+01 | 0.06% | 0.01% | 57.1bp (16.6bp) | Zac1(Zf)/Neuro2A-Plagl1-ChIP-Seq(GSE75942)/Homer(0.738) More Information | Similar Motifs Found | motif file (matrix) |
