# Supplementary material for: Reconstitution of pluripotency from mouse fibroblast through Sall4 overexpression
Source: Nat Commun. 2024 Dec 30;15:10787. doi: 10.1038/s41467-024-54924-5 (PMC11686038; doi:10.1038/s41467-024-54924-5)
Supplement: Supplementary file 4 — Source Data [file 41467_2024_54924_MOESM4_ESM.zip › source data/main figures/figure2/e/D0_S4.rmdup.sort.bed.motif/homerResults/motif1.similar.html]

motif1

## Information for motif1

C
A
T
G
T
A
G
C
C
A
G
T
T
C
G
A
C
G
A
T
A
C
T
G
C
G
T
A
A
T
G
C
G
C
A
T
T
G
A
C
G
C
T
A
A
C
T
G
  
Reverse Opposite:  

T
G
A
C
C
G
A
T
A
C
T
G
C
G
T
A
T
A
C
G
G
C
A
T
T
G
A
C
G
C
T
A
A
G
C
T
G
T
C
A
A
T
C
G
G
T
A
C
  

|  |  |
| --- | --- |
| p-value: | 1e-2950 |
| log p-value: | -6.793e+03 |
| Information Content per bp: | 1.532 |
| Number of Target Sequences with motif | 8970.0 |
| Percentage of Target Sequences with motif | 22.43% |
| Number of Background Sequences with motif | 2105.4 |
| Percentage of Background Sequences with motif | 5.27% |
| Average Position of motif in Targets | 99.9 +/- 54.4bp |
| Average Position of motif in Background | 100.2 +/- 60.7bp |
| Strand Bias (log2 ratio + to - strand density) | 0.0 |
| Multiplicity (# of sites on avg that occur together) | 1.15 |
| Motif File: | file (matrix) reverse opposite |

### Similar de novo motifs found

|  |  |  |  |  |  |  |  |
| --- | --- | --- | --- | --- | --- | --- | --- |
| Rank | Match Score | Redundant Motif | P-value | log P-value | % of Targets | % of Background | Motif file |
| 1 | 0.983 | A T G C C A T G T C G A G A C T C A T G C G T A A T C G G C A T G T A C C T G A | 1e-2883 | -6639.802170 | 21.76% | 5.06% | motif file (matrix) |
| 2 | 0.969 | T C G A A G C T A C T G C G T A T A C G A C G T G T A C C T G A | 1e-2778 | -6398.087531 | 22.17% | 5.44% | motif file (matrix) |
| 3 | 0.939 | A G C T C T A G G C T A T A C G A C G T T G A C C T G A A G C T | 1e-2225 | -5125.086686 | 31.55% | 12.27% | motif file (matrix) |
| 4 | 0.923 | C G A T T C G A A C G T A C T G C G T A A G C T C G A T G T A C C T G A A G C T | 1e-2171 | -4999.231493 | 22.40% | 6.80% | motif file (matrix) |
| 5 | 0.798 | T G A C G A C T T C A G C G T A A G T C G C A T A G T C C T A G | 1e-1823 | -4198.157546 | 23.84% | 8.56% | motif file (matrix) |
| 6 | 0.659 | T C G A T C A G G A C T G T A C C G T A A G T C A C T G T G A C | 1e-422 | -971.939277 | 30.91% | 21.46% | motif file (matrix) |
| 7 | 0.802 | A G C T A C G T C G T A A T C G A C G T A G T C C G T A A G C T G T A C G C A T | 1e-392 | -904.024283 | 7.03% | 2.86% | motif file (matrix) |
| 8 | 0.611 | A T C G T C G A G A C T A C G T C G T A T G A C C A T G A G T C G T C A G T C A | 1e-308 | -710.846724 | 19.63% | 12.92% | motif file (matrix) |
| 9 | 0.633 | A C T G A C G T A C T G C G T A A G T C A C G T C G T A C G T A A C T G A G T C C G T A C G T A | 1e-224 | -515.995900 | 20.46% | 14.53% | motif file (matrix) |
| 10 | 0.669 | T A G C A G C T T C A G G C T A G C A T A C G T G C T A T C G A | 1e-190 | -439.363970 | 33.84% | 27.13% | motif file (matrix) |
| 11 | 0.744 | G C T A T A C G C G A T A T C G C G A T T A C G C G T A C G A T G A C T T A G C G C T A A T G C | 1e-168 | -388.207536 | 1.56% | 0.40% | motif file (matrix) |
| 12 | 0.684 | T A G C G T C A A G C T A C G T C G T A T A G C G C A T G T A C G T C A T A C G T G A C T C G A | 1e-158 | -365.693151 | 2.94% | 1.20% | motif file (matrix) |
| 13 | 0.638 | T C A G C G T A A T C G G C A T T A G C G C T A A G C T A C G T A G T C G T A C G T C A T C G A | 1e-141 | -326.555125 | 8.32% | 5.26% | motif file (matrix) |
| 14 | 0.647 | G C A T C T G A G C A T A G T C G C T A C G A T T A C G G T C A C G A T C A G T G T A C G T C A | 1e-88 | -203.537244 | 0.23% | 0.01% | motif file (matrix) |
