# Supplementary material for: Reconstitution of pluripotency from mouse fibroblast through Sall4 overexpression
Source: Nat Commun. 2024 Dec 30;15:10787. doi: 10.1038/s41467-024-54924-5 (PMC11686038; doi:10.1038/s41467-024-54924-5)
Supplement: Supplementary file 4 — Source Data [file 41467_2024_54924_MOESM4_ESM.zip › source data/main figures/figure2/e/D0_S4.rmdup.sort.bed.motif/homerResults/motif11.similar.html]

motif11

## Information for motif11

A
G
C
T
C
G
A
T
C
A
T
G
C
T
A
G
G
A
T
C
G
C
A
T
A
T
G
C
T
C
A
G
  
Reverse Opposite:  

A
G
T
C
T
A
C
G
C
G
T
A
C
T
A
G
G
A
T
C
G
T
A
C
G
C
T
A
T
C
G
A
  

|  |  |
| --- | --- |
| p-value: | 1e-68 |
| log p-value: | -1.570e+02 |
| Information Content per bp: | 1.609 |
| Number of Target Sequences with motif | 6061.0 |
| Percentage of Target Sequences with motif | 15.16% |
| Number of Background Sequences with motif | 4869.2 |
| Percentage of Background Sequences with motif | 12.20% |
| Average Position of motif in Targets | 99.1 +/- 55.8bp |
| Average Position of motif in Background | 99.0 +/- 59.9bp |
| Strand Bias (log2 ratio + to - strand density) | 0.0 |
| Multiplicity (# of sites on avg that occur together) | 1.09 |
| Motif File: | file (matrix) reverse opposite |

### Similar de novo motifs found

|  |  |  |  |  |  |  |  |
| --- | --- | --- | --- | --- | --- | --- | --- |
| Rank | Match Score | Redundant Motif | P-value | log P-value | % of Targets | % of Background | Motif file |
| 1 | 0.685 | A G C T A G C T C A G T C A T G A C T G A G T C G A C T A C G T C A T G A C T G A T G C G A T C | 1e-47 | -110.010017 | 5.50% | 4.00% | motif file (matrix) |
| 2 | 0.672 | A C T G A C G T A C G T A T C G C G T A A G T C A C G T A G T C A C T G A C G T | 1e-26 | -61.045231 | 0.10% | 0.01% | motif file (matrix) |
| 3 | 0.605 | A C G T A C G T A C T G A C T G A C T G A C G T A G T C C G T A A C T G A C G T | 1e-11 | -27.146989 | 0.06% | 0.01% | motif file (matrix) |
