# Supplementary material for: Reconstitution of pluripotency from mouse fibroblast through Sall4 overexpression
Source: Nat Commun. 2024 Dec 30;15:10787. doi: 10.1038/s41467-024-54924-5 (PMC11686038; doi:10.1038/s41467-024-54924-5)
Supplement: Supplementary file 4 — Source Data [file 41467_2024_54924_MOESM4_ESM.zip › source data/main figures/figure2/e/D0_S4.rmdup.sort.bed.motif/homerResults/motif14.info.html]

Motif 14

## Information for 13-AAACATTTCCTG (Motif 14)

T
C
G
A
T
C
G
A
C
G
T
A
A
G
T
C
G
C
T
A
A
G
C
T
A
C
G
T
A
G
C
T
A
G
T
C
G
A
T
C
G
C
A
T
A
C
T
G
  
Reverse Opposite:  
