# Supplementary material for: Reconstitution of pluripotency from mouse fibroblast through Sall4 overexpression
Source: Nat Commun. 2024 Dec 30;15:10787. doi: 10.1038/s41467-024-54924-5 (PMC11686038; doi:10.1038/s41467-024-54924-5)
Supplement: Supplementary file 4 — Source Data [file 41467_2024_54924_MOESM4_ESM.zip › source data/main figures/figure2/e/D0_S4.rmdup.sort.bed.motif/homerResults/motif2.similar.html]

motif2

## Information for motif2

T
C
G
A
C
T
A
G
T
G
A
C
C
G
T
A
G
C
A
T
C
G
A
T
A
T
G
C
G
A
T
C
C
G
T
A
C
A
T
G
  
Reverse Opposite:  

G
T
A
C
G
C
A
T
C
T
A
G
T
A
C
G
G
C
T
A
C
G
T
A
G
C
A
T
A
C
T
G
A
G
T
C
A
G
C
T
  

|  |  |
| --- | --- |
| p-value: | 1e-295 |
| log p-value: | -6.804e+02 |
| Information Content per bp: | 1.629 |
| Number of Target Sequences with motif | 4620.0 |
| Percentage of Target Sequences with motif | 11.55% |
| Number of Background Sequences with motif | 2614.1 |
| Percentage of Background Sequences with motif | 6.55% |
| Average Position of motif in Targets | 100.3 +/- 55.7bp |
| Average Position of motif in Background | 99.2 +/- 57.7bp |
| Strand Bias (log2 ratio + to - strand density) | -0.0 |
| Multiplicity (# of sites on avg that occur together) | 1.08 |
| Motif File: | file (matrix) reverse opposite |

### Similar de novo motifs found

|  |  |  |  |  |  |  |  |
| --- | --- | --- | --- | --- | --- | --- | --- |
| Rank | Match Score | Redundant Motif | P-value | log P-value | % of Targets | % of Background | Motif file |
| 1 | 0.964 | G T A C T C G A T G A C G T C A A C G T A G C T A G T C A G T C C G A T C A T G C G T A A T G C | 1e-286 | -660.153852 | 10.77% | 6.02% | motif file (matrix) |
| 2 | 0.917 | G T C A C T G A A T G C C G T A A C G T C G A T T A G C A G T C | 1e-268 | -617.188149 | 8.51% | 4.47% | motif file (matrix) |
| 3 | 0.871 | C A T G C T A G A C T G G T C A C G T A A C G T A C T G A G T C | 1e-240 | -553.769927 | 14.15% | 9.05% | motif file (matrix) |
| 4 | 0.839 | C T G A G T C A C T G A A C G T C G T A A T G C A G T C C G T A | 1e-181 | -419.064284 | 10.71% | 6.80% | motif file (matrix) |
| 5 | 0.647 | C T G A T A C G A C T G T G C A C T G A C G T A A G T C T A G C | 1e-136 | -313.485947 | 33.67% | 27.98% | motif file (matrix) |
| 6 | 0.716 | C G T A G C T A T G C A T G A C G C T A G C A T C G A T G A T C G A T C G T A C | 1e-95 | -219.646883 | 16.00% | 12.44% | motif file (matrix) |
| 7 | 0.653 | G A T C G T A C A C G T A C T G A G T C A G T C C T G A C T A G | 1e-87 | -200.326999 | 15.81% | 12.42% | motif file (matrix) |
| 8 | 0.668 | A C T G G T C A C G T A A C G T A C T G A C T G C G T A C G T A A C G T A G T C | 1e-8 | -20.719218 | 0.11% | 0.04% | motif file (matrix) |
