# Supplementary material for: Reconstitution of pluripotency from mouse fibroblast through Sall4 overexpression
Source: Nat Commun. 2024 Dec 30;15:10787. doi: 10.1038/s41467-024-54924-5 (PMC11686038; doi:10.1038/s41467-024-54924-5)
Supplement: Supplementary file 4 — Source Data [file 41467_2024_54924_MOESM4_ESM.zip › source data/main figures/figure2/e/D0_S4.rmdup.sort.bed.motif/homerResults/motif21.similar.html]

motif21

## Information for motif21

A
T
G
C
C
A
T
G
A
T
C
G
A
C
G
T
A
C
G
T
A
C
T
G
A
C
T
G
C
G
T
A
C
G
A
T
C
G
T
A
C
G
T
A
A
C
G
T
  
Reverse Opposite:  

C
G
T
A
A
C
G
T
A
C
G
T
C
G
T
A
C
G
A
T
A
G
T
C
A
G
T
C
C
G
T
A
C
G
T
A
A
T
G
C
G
T
A
C
T
A
C
G
  

|  |  |
| --- | --- |
| p-value: | 1e-31 |
| log p-value: | -7.266e+01 |
| Information Content per bp: | 1.835 |
| Number of Target Sequences with motif | 36.0 |
| Percentage of Target Sequences with motif | 0.09% |
| Number of Background Sequences with motif | 3.0 |
| Percentage of Background Sequences with motif | 0.01% |
| Average Position of motif in Targets | 107.6 +/- 56.3bp |
| Average Position of motif in Background | 89.6 +/- 52.2bp |
| Strand Bias (log2 ratio + to - strand density) | -0.2 |
| Multiplicity (# of sites on avg that occur together) | 1.00 |
| Motif File: | file (matrix) reverse opposite |

### Similar de novo motifs found

|  |  |  |  |  |  |  |  |
| --- | --- | --- | --- | --- | --- | --- | --- |
| Rank | Match Score | Redundant Motif | P-value | log P-value | % of Targets | % of Background | Motif file |
