# Supplementary material for: Reconstitution of pluripotency from mouse fibroblast through Sall4 overexpression
Source: Nat Commun. 2024 Dec 30;15:10787. doi: 10.1038/s41467-024-54924-5 (PMC11686038; doi:10.1038/s41467-024-54924-5)
Supplement: Supplementary file 4 — Source Data [file 41467_2024_54924_MOESM4_ESM.zip › source data/main figures/figure2/e/D0_S4.rmdup.sort.bed.motif/homerResults/motif3.similar.html]

motif3

## Information for motif3

C
G
A
T
T
G
C
A
C
T
G
A
A
G
T
C
A
T
G
C
C
G
T
A
T
G
A
C
C
G
T
A
  
Reverse Opposite:  

A
C
G
T
A
C
T
G
G
C
A
T
A
T
C
G
A
C
T
G
A
G
C
T
A
C
G
T
C
G
T
A
  

|  |  |
| --- | --- |
| p-value: | 1e-272 |
| log p-value: | -6.282e+02 |
| Information Content per bp: | 1.739 |
| Number of Target Sequences with motif | 8097.0 |
| Percentage of Target Sequences with motif | 20.25% |
| Number of Background Sequences with motif | 5510.9 |
| Percentage of Background Sequences with motif | 13.80% |
| Average Position of motif in Targets | 99.9 +/- 56.1bp |
| Average Position of motif in Background | 99.4 +/- 59.2bp |
| Strand Bias (log2 ratio + to - strand density) | 0.0 |
| Multiplicity (# of sites on avg that occur together) | 1.15 |
| Motif File: | file (matrix) reverse opposite |

### Similar de novo motifs found

|  |  |  |  |  |  |  |  |
| --- | --- | --- | --- | --- | --- | --- | --- |
| Rank | Match Score | Redundant Motif | P-value | log P-value | % of Targets | % of Background | Motif file |
| 1 | 0.937 | A T G C G A C T A T C G G C A T A T C G C A T G A G C T G A C T G C A T T C A G | 1e-251 | -578.422035 | 13.17% | 8.16% | motif file (matrix) |
| 2 | 0.635 | C T A G T C G A C G A T A C T G G C T A A T G C T C A G C G A T T G A C C G T A | 1e-230 | -529.764395 | 9.12% | 5.17% | motif file (matrix) |
| 3 | 0.620 | T C A G C T A G T C A G A C G T A C T G C G T A A T G C T A C G A C G T A G T C C G T A A G C T | 1e-212 | -489.889196 | 4.55% | 2.01% | motif file (matrix) |
| 4 | 0.826 | A T C G A C G T A C T G A G C T A C T G A C T G A C G T A G T C | 1e-198 | -456.575479 | 10.49% | 6.48% | motif file (matrix) |
| 5 | 0.871 | A C G T C G A T A T C G A C G T C T A G A C T G A G C T A C G T C G T A A C T G G C A T A G C T | 1e-144 | -331.780815 | 4.22% | 2.13% | motif file (matrix) |
| 6 | 0.775 | G C T A T C G A T C G A T G A C G T A C G T C A T A G C C G T A C T A G T A C G T G A C G T A C | 1e-139 | -320.821030 | 12.57% | 8.80% | motif file (matrix) |
| 7 | 0.613 | A G C T A C T G C G T A A G T C C T G A A C G T A G T C C T G A | 1e-123 | -283.381496 | 4.10% | 2.17% | motif file (matrix) |
| 8 | 0.617 | G C T A C A G T A C T G T G A C A G T C G C A T A C T G C G A T A C T G A T C G | 1e-85 | -195.881143 | 7.21% | 4.95% | motif file (matrix) |
