# Supplementary material for: Reconstitution of pluripotency from mouse fibroblast through Sall4 overexpression
Source: Nat Commun. 2024 Dec 30;15:10787. doi: 10.1038/s41467-024-54924-5 (PMC11686038; doi:10.1038/s41467-024-54924-5)
Supplement: Supplementary file 4 — Source Data [file 41467_2024_54924_MOESM4_ESM.zip › source data/main figures/figure2/e/D0_S4.rmdup.sort.bed.motif/homerResults/motif4.similar.html]

motif4

## Information for motif4

A
G
T
C
A
G
C
T
A
G
C
T
T
A
C
G
A
T
G
C
A
G
T
C
C
G
T
A
T
C
A
G
C
T
G
A
T
C
A
G
  
Reverse Opposite:  

A
G
T
C
A
G
C
T
A
G
T
C
A
C
G
T
A
C
T
G
A
T
C
G
A
T
G
C
T
C
G
A
C
T
G
A
T
C
A
G
  

|  |  |
| --- | --- |
| p-value: | 1e-154 |
| log p-value: | -3.551e+02 |
| Information Content per bp: | 1.654 |
| Number of Target Sequences with motif | 12479.0 |
| Percentage of Target Sequences with motif | 31.20% |
| Number of Background Sequences with motif | 10103.6 |
| Percentage of Background Sequences with motif | 25.31% |
| Average Position of motif in Targets | 100.2 +/- 55.5bp |
| Average Position of motif in Background | 99.2 +/- 58.0bp |
| Strand Bias (log2 ratio + to - strand density) | 0.0 |
| Multiplicity (# of sites on avg that occur together) | 1.17 |
| Motif File: | file (matrix) reverse opposite |

### Similar de novo motifs found

|  |  |  |  |  |  |  |  |
| --- | --- | --- | --- | --- | --- | --- | --- |
| Rank | Match Score | Redundant Motif | P-value | log P-value | % of Targets | % of Background | Motif file |
| 1 | 0.820 | A G C T A G C T C A G T C A T G T A C G T G A C G T C A C T G A | 1e-136 | -314.123739 | 47.45% | 41.28% | motif file (matrix) |
| 2 | 0.731 | A G C T A G C T A G T C G A T C G A T C G T A C C G T A C T A G C T A G T C A G C T A G T A C G | 1e-95 | -219.002668 | 11.72% | 8.66% | motif file (matrix) |
| 3 | 0.607 | T C A G T A G C G C T A A T C G T A G C A G T C C G A T A C T G T A G C G T A C G T C A T C G A | 1e-55 | -127.595267 | 5.69% | 4.04% | motif file (matrix) |
| 4 | 0.738 | C T G A T G C A G C T A A T G C G A C T C A G T C A T G A T C G T A G C G T C A C T G A C T G A | 1e-36 | -82.910306 | 0.42% | 0.13% | motif file (matrix) |
| 5 | 0.622 | G T C A A G T C A C G T A G T C A C G T A C T G A C T G A T C G | 1e-32 | -74.438140 | 5.21% | 3.99% | motif file (matrix) |
