# Supplementary material for: Reconstitution of pluripotency from mouse fibroblast through Sall4 overexpression
Source: Nat Commun. 2024 Dec 30;15:10787. doi: 10.1038/s41467-024-54924-5 (PMC11686038; doi:10.1038/s41467-024-54924-5)
Supplement: Supplementary file 4 — Source Data [file 41467_2024_54924_MOESM4_ESM.zip › source data/main figures/figure2/e/D0_S4.rmdup.sort.bed.motif/homerResults/motif8.similar.html]

motif8

## Information for motif8

C
T
A
G
A
G
T
C
G
T
A
C
G
A
T
C
G
T
A
C
C
T
A
G
A
T
G
C
G
A
T
C
A
G
T
C
G
A
C
T
  
Reverse Opposite:  

C
T
G
A
T
C
A
G
C
T
A
G
T
A
C
G
G
A
T
C
C
A
T
G
C
T
A
G
C
A
T
G
T
C
A
G
G
A
T
C
  

|  |  |
| --- | --- |
| p-value: | 1e-90 |
| log p-value: | -2.094e+02 |
| Information Content per bp: | 1.531 |
| Number of Target Sequences with motif | 8255.0 |
| Percentage of Target Sequences with motif | 20.64% |
| Number of Background Sequences with motif | 6685.5 |
| Percentage of Background Sequences with motif | 16.75% |
| Average Position of motif in Targets | 99.3 +/- 55.2bp |
| Average Position of motif in Background | 96.2 +/- 56.9bp |
| Strand Bias (log2 ratio + to - strand density) | 0.0 |
| Multiplicity (# of sites on avg that occur together) | 1.39 |
| Motif File: | file (matrix) reverse opposite |

### Similar de novo motifs found

|  |  |  |  |  |  |  |  |
| --- | --- | --- | --- | --- | --- | --- | --- |
| Rank | Match Score | Redundant Motif | P-value | log P-value | % of Targets | % of Background | Motif file |
| 1 | 0.861 | T A C G C T A G C T A G A C T G A C T G A G T C A C T G A C T G C T A G T C A G A G T C A C G T | 1e-73 | -168.893458 | 7.88% | 5.66% | motif file (matrix) |
| 2 | 0.774 | G A C T A T G C G A T C C T A G T G A C A T G C G T A C G C T A | 1e-58 | -134.369320 | 9.29% | 7.12% | motif file (matrix) |
