# Supplementary material for: Reconstitution of pluripotency from mouse fibroblast through Sall4 overexpression
Source: Nat Commun. 2024 Dec 30;15:10787. doi: 10.1038/s41467-024-54924-5 (PMC11686038; doi:10.1038/s41467-024-54924-5)
Supplement: Supplementary file 4 — Source Data [file 41467_2024_54924_MOESM4_ESM.zip › source data/main figures/figure2/e/D0_S4.rmdup.sort.bed.motif/knownResults.html]

rmdup\_vs\_igg\_motifs/D0\_S4.rmdup.sort.bed.motif - Homer Known Motif Enrichment Results


# Homer Known Motif Enrichment Results (rmdup\_vs\_igg\_motifs/D0\_S4.rmdup.sort.bed.motif)

Homer *de novo* Motif Results  
Gene Ontology Enrichment Results  
Known Motif Enrichment Results (txt file)  
Total Target Sequences = 39991, Total Background Sequences = 39935

|  |  |  |  |  |  |  |  |  |  |  |  |
| --- | --- | --- | --- | --- | --- | --- | --- | --- | --- | --- | --- |
| Rank | Motif | Name | P-value | log P-pvalue | q-value (Benjamini) | # Target Sequences with Motif | % of Targets Sequences with Motif | # Background Sequences with Motif | % of Background Sequences with Motif | Motif File | SVG |
| 1 | A C T G C A T G T C G A A C G T A C T G C G T A A T C G A C G T G T A C C G T A G A C T A G T C | Fos(bZIP)/TSC-Fos-ChIP-Seq(GSE110950)/Homer | 1e-3059 | -7.045e+03 | 0.0000 | 8950.0 | 22.38% | 2024.7 | 5.07% | motif file (matrix) | svg |
| 2 | A C T G C T A G T C G A C G A T C A T G G C T A A T C G C G A T G T A C G C T A A G C T G T A C | Fra1(bZIP)/BT549-Fra1-ChIP-Seq(GSE46166)/Homer | 1e-3021 | -6.957e+03 | 0.0000 | 8670.0 | 21.68% | 1918.3 | 4.81% | motif file (matrix) | svg |
| 3 | C T A G T C G A G C A T C A T G G C T A T A G C C G A T G T A C C T G A A G C T | JunB(bZIP)/DendriticCells-Junb-ChIP-Seq(GSE36099)/Homer | 1e-2973 | -6.846e+03 | 0.0000 | 8652.0 | 21.63% | 1940.0 | 4.86% | motif file (matrix) | svg |
| 4 | C T A G T C G A A C G T A C T G C G T A A T G C A C G T G T A C C G T A A G C T G A T C G T A C | Atf3(bZIP)/GBM-ATF3-ChIP-Seq(GSE33912)/Homer | 1e-2948 | -6.790e+03 | 0.0000 | 9542.0 | 23.86% | 2386.4 | 5.98% | motif file (matrix) | svg |
| 5 | C A T G C T A G T C G A A C G T A C T G C G T A T A G C C G A T T G A C C G T A A G C T G A T C | Fra2(bZIP)/Striatum-Fra2-ChIP-Seq(GSE43429)/Homer | 1e-2878 | -6.628e+03 | 0.0000 | 7950.0 | 19.88% | 1679.6 | 4.21% | motif file (matrix) | svg |
| 6 | C A G T T G C A A C G T A C T G C G T A A T C G C G A T T G A C C G T A A C G T | BATF(bZIP)/Th17-BATF-ChIP-Seq(GSE39756)/Homer | 1e-2838 | -6.535e+03 | 0.0000 | 9403.0 | 23.51% | 2397.8 | 6.01% | motif file (matrix) | svg |
| 7 | T C G A A C G T C A T G G C T A T A G C C G A T G T A C G C T A A C G T A T G C | AP-1(bZIP)/ThioMac-PU.1-ChIP-Seq(GSE21512)/Homer | 1e-2755 | -6.346e+03 | 0.0000 | 9927.0 | 24.82% | 2733.1 | 6.85% | motif file (matrix) | svg |
| 8 | C T A G T C G A C G A T A C T G C G T A T A C G A G C T T G A C G C T A A C G T G A T C T A G C | Fosl2(bZIP)/3T3L1-Fosl2-ChIP-Seq(GSE56872)/Homer | 1e-2754 | -6.342e+03 | 0.0000 | 6510.0 | 16.28% | 1143.2 | 2.86% | motif file (matrix) | svg |
| 9 | C T A G T C G A A C G T A C T G C G T A T A G C C G A T G T A C C G T A A G C T G A T C G T A C | Jun-AP1(bZIP)/K562-cJun-ChIP-Seq(GSE31477)/Homer | 1e-2450 | -5.643e+03 | 0.0000 | 5265.0 | 13.16% | 813.2 | 2.04% | motif file (matrix) | svg |
| 10 | C G A T T A C G T G A C G A C T C A T G C G T A T A C G A C G T G T A C C T G A | Bach2(bZIP)/OCILy7-Bach2-ChIP-Seq(GSE44420)/Homer | 1e-1168 | -2.690e+03 | 0.0000 | 3343.0 | 8.36% | 696.8 | 1.75% | motif file (matrix) | svg |
| 11 | T A C G T C G A C A G T A C T G G C T A A T G C C G A T G T A C C G T A A C T G T A G C C G T A | NF-E2(bZIP)/K562-NFE2-ChIP-Seq(GSE31477)/Homer | 1e-403 | -9.282e+02 | 0.0000 | 1123.0 | 2.81% | 223.2 | 0.56% | motif file (matrix) | svg |
| 12 | C G T A C G T A C G T A G C A T G C A T T A C G G T A C G A C T A C T G C G T A A T C G A C G T G T A C C G T A A G C T | Bach1(bZIP)/K562-Bach1-ChIP-Seq(GSE31477)/Homer | 1e-366 | -8.431e+02 | 0.0000 | 1014.0 | 2.54% | 200.0 | 0.50% | motif file (matrix) | svg |
| 13 | G T C A G C A T A C T G G T A C G A C T A C T G G C T A A T C G C A G T G T A C C G T A A G C T | Nrf2(bZIP)/Lymphoblast-Nrf2-ChIP-Seq(GSE37589)/Homer | 1e-336 | -7.739e+02 | 0.0000 | 937.0 | 2.34% | 186.6 | 0.47% | motif file (matrix) | svg |
| 14 | A G C T C T A G T G A C C G T A A C G T C G A T A G T C A G T C C T G A C A T G | TEAD3(TEA)/HepG2-TEAD3-ChIP-Seq(Encode)/Homer | 1e-269 | -6.217e+02 | 0.0000 | 6184.0 | 15.46% | 3929.4 | 9.84% | motif file (matrix) | svg |
| 15 | T G A C A G T C C T G A T G A C C G T A A C G T A C G T A G T C A G T C C G T A | TEAD1(TEAD)/HepG2-TEAD1-ChIP-Seq(Encode)/Homer | 1e-265 | -6.104e+02 | 0.0000 | 5464.0 | 13.66% | 3366.5 | 8.43% | motif file (matrix) | svg |
| 16 | T A G C T A G C G C A T C A T G A C T G G C T A C G T A A C G T A C T G G A T C | TEAD4(TEA)/Tropoblast-Tead4-ChIP-Seq(GSE37350)/Homer | 1e-254 | -5.863e+02 | 0.0000 | 4890.0 | 12.23% | 2946.7 | 7.38% | motif file (matrix) | svg |
| 17 | G A T C G T A C C G A T A C T G A C T G C G T A C G T A A C G T A C T G G A T C | TEAD(TEA)/Fibroblast-PU.1-ChIP-Seq(Unpublished)/Homer | 1e-243 | -5.612e+02 | 0.0000 | 3855.0 | 9.64% | 2180.8 | 5.46% | motif file (matrix) | svg |
| 18 | T A G C C T A G T C G A G A C T A C T G C T G A A G T C T C A G G C A T T G A C C T G A A G C T | Atf7(bZIP)/3T3L1-Atf7-ChIP-Seq(GSE56872)/Homer | 1e-232 | -5.364e+02 | 0.0000 | 3069.0 | 7.67% | 1625.5 | 4.07% | motif file (matrix) | svg |
| 19 | T C G A G C A T A C T G C T G A A G T C T C A G G A C T G T A C C G T A A G C T A G T C G A T C | c-Jun-CRE(bZIP)/K562-cJun-ChIP-Seq(GSE31477)/Homer | 1e-225 | -5.199e+02 | 0.0000 | 2183.0 | 5.46% | 1018.4 | 2.55% | motif file (matrix) | svg |
| 20 | T G C A T C G A T C G A A C G T C A T G C G T A A G T C T C A G A C G T G T A C C G T A A G C T | CREB5(bZIP)/LNCaP-CREB5.V5-ChIP-Seq(GSE137775)/Homer | 1e-225 | -5.195e+02 | 0.0000 | 2415.0 | 6.04% | 1177.3 | 2.95% | motif file (matrix) | svg |
| 21 | A T G C T C A G T C G A G C A T A C T G C G T A A G T C T C A G G A C T T G A C C G T A A G C T | Atf2(bZIP)/3T3L1-Atf2-ChIP-Seq(GSE56872)/Homer | 1e-225 | -5.188e+02 | 0.0000 | 2346.0 | 5.87% | 1130.6 | 2.83% | motif file (matrix) | svg |
| 22 | T C A G T A G C G A C T C A T G C T G A A T C G G C A T G T A C C G T A A C T G T A G C T G C A | MafK(bZIP)/C2C12-MafK-ChIP-Seq(GSE36030)/Homer | 1e-223 | -5.158e+02 | 0.0000 | 2250.0 | 5.63% | 1067.9 | 2.67% | motif file (matrix) | svg |
| 23 | A C T G G A T C G A C T A C T G A C G T C A T G A C T G A C G T A G C T C G A T | RUNX-AML(Runt)/CD4+-PolII-ChIP-Seq(Barski\_et\_al.)/Homer | 1e-204 | -4.698e+02 | 0.0000 | 4468.0 | 11.17% | 2783.6 | 6.97% | motif file (matrix) | svg |
| 24 | C G T A C G T A C G T A G C A T G C A T A C T G G T A C G A C T C T A G G C T A T A C G G A C T T G A C C G T A A G C T | NFE2L2(bZIP)/HepG2-NFE2L2-ChIP-Seq(Encode)/Homer | 1e-183 | -4.234e+02 | 0.0000 | 857.0 | 2.14% | 264.7 | 0.66% | motif file (matrix) | svg |
| 25 | T A C G T C G A G A C T A C T G C T G A A G T C T C A G G A C T T G A C C T G A | Atf1(bZIP)/K562-ATF1-ChIP-Seq(GSE31477)/Homer | 1e-173 | -3.990e+02 | 0.0000 | 3725.0 | 9.31% | 2301.4 | 5.76% | motif file (matrix) | svg |
| 26 | A G T C G A C T C A G T A C T G C T A G T G A C G C T A A T G C G C A T A T C G C G A T A C T G G A T C G T A C G T C A C T G A | NF1(CTF)/LNCAP-NF1-ChIP-Seq(Unpublished)/Homer | 1e-162 | -3.748e+02 | 0.0000 | 2445.0 | 6.11% | 1351.0 | 3.38% | motif file (matrix) | svg |
| 27 | T G A C G C T A T C G A T G C A A G T C A G T C C G T A A G T C C G T A C T G A G C T A G T A C | RUNX2(Runt)/PCa-RUNX2-ChIP-Seq(GSE33889)/Homer | 1e-156 | -3.604e+02 | 0.0000 | 5108.0 | 12.77% | 3500.0 | 8.77% | motif file (matrix) | svg |
| 28 | T A G C G C T A T C G A C T G A A G T C A G T C C T G A A G T C C G T A C T A G | RUNX(Runt)/HPC7-Runx1-ChIP-Seq(GSE22178)/Homer | 1e-151 | -3.494e+02 | 0.0000 | 4426.0 | 11.07% | 2953.0 | 7.40% | motif file (matrix) | svg |
| 29 | A T G C G A T C C G A T C T A G A C T G G C T A C G T A A G C T A C T G A G C T | TEAD2(TEA)/Py2T-Tead2-ChIP-Seq(GSE55709)/Homer | 1e-150 | -3.457e+02 | 0.0000 | 3036.0 | 7.59% | 1839.3 | 4.61% | motif file (matrix) | svg |
| 30 | G C T A C T G A T C G A A G T C A G T C C T G A A G T C G T C A C T G A T G C A | RUNX1(Runt)/Jurkat-RUNX1-ChIP-Seq(GSE29180)/Homer | 1e-131 | -3.039e+02 | 0.0000 | 5901.0 | 14.76% | 4300.1 | 10.77% | motif file (matrix) | svg |
| 31 | T C G A A C G T A C T G C T G A A G T C T C A G A G C T G T A C C G T A A G C T G A T C T C G A | JunD(bZIP)/K562-JunD-ChIP-Seq/Homer | 1e-125 | -2.895e+02 | 0.0000 | 829.0 | 2.07% | 318.4 | 0.80% | motif file (matrix) | svg |
| 32 | G A C T C T A G G A T C C A G T A C T G C T G A A T G C G C A T A T G C C T G A | MafA(bZIP)/Islet-MafA-ChIP-Seq(GSE30298)/Homer | 1e-105 | -2.436e+02 | 0.0000 | 4852.0 | 12.13% | 3538.9 | 8.86% | motif file (matrix) | svg |
| 33 | T C G A A C G T A C G T C T G A G A T C T C A G G A C T G T C A C G T A A G C T G T C A C T A G A G C T A C G T T C G A | NFIL3(bZIP)/HepG2-NFIL3-ChIP-Seq(Encode)/Homer | 1e-98 | -2.268e+02 | 0.0000 | 2754.0 | 6.89% | 1808.8 | 4.53% | motif file (matrix) | svg |
| 34 | T C G A T A G C G T C A A C T G A C T G C G T A C G T A C T A G A G C T T C A G | ERG(ETS)/VCaP-ERG-ChIP-Seq(GSE14097)/Homer | 1e-84 | -1.946e+02 | 0.0000 | 8027.0 | 20.07% | 6529.3 | 16.36% | motif file (matrix) | svg |
| 35 | A G C T A C G T A C T G A T G C A G T C C G T A C T G A T A C G | NF1-halfsite(CTF)/LNCaP-NF1-ChIP-Seq(Unpublished)/Homer | 1e-80 | -1.857e+02 | 0.0000 | 8483.0 | 21.21% | 6983.5 | 17.49% | motif file (matrix) | svg |
| 36 | A G T C C T G A A G T C C G A T C A G T G A T C A T G C A C T G A T C G G A C T | Fli1(ETS)/CD8-FLI-ChIP-Seq(GSE20898)/Homer | 1e-65 | -1.497e+02 | 0.0000 | 5768.0 | 14.42% | 4629.9 | 11.60% | motif file (matrix) | svg |
| 37 | C G A T C T A G A C G T G T C A C G T A C G T A A G T C C G T A | Foxo3(Forkhead)/U2OS-Foxo3-ChIP-Seq(E-MTAB-2701)/Homer | 1e-62 | -1.446e+02 | 0.0000 | 3322.0 | 8.31% | 2469.8 | 6.19% | motif file (matrix) | svg |
| 38 | G A C T T C A G C T A G A G T C A G T C G T A C A G T C C T G A A G T C A G T C A G T C G A C T A G T C A C T G A T G C | KLF3(Zf)/MEF-Klf3-ChIP-Seq(GSE44748)/Homer | 1e-60 | -1.384e+02 | 0.0000 | 3050.0 | 7.63% | 2250.2 | 5.64% | motif file (matrix) | svg |
| 39 | T C G A T A G C T G C A A C T G A C T G C G T A C G T A C T A G G A C T T A C G | ETS1(ETS)/Jurkat-ETS1-ChIP-Seq(GSE17954)/Homer | 1e-59 | -1.375e+02 | 0.0000 | 5387.0 | 13.47% | 4328.9 | 10.84% | motif file (matrix) | svg |
| 40 | T C A G C T A G A T G C G A C T T G C A C G T A A G C T A C G T C T G A T A C G | En1(Homeobox)/SUM149-EN1-ChIP-Seq(GSE120957)/Homer | 1e-59 | -1.370e+02 | 0.0000 | 7750.0 | 19.38% | 6504.0 | 16.29% | motif file (matrix) | svg |
| 41 | G A C T G T A C T G C A A C G T G A T C G C T A T C G A A C G T A G T C C G T A | Pdx1(Homeobox)/Islet-Pdx1-ChIP-Seq(SRA008281)/Homer | 1e-58 | -1.347e+02 | 0.0000 | 4061.0 | 10.15% | 3148.0 | 7.89% | motif file (matrix) | svg |
| 42 | T G C A A G C T A C G T C T A G G A T C C T A G G A T C G T C A C T G A A G T C | CEBP(bZIP)/ThioMac-CEBPb-ChIP-Seq(GSE21512)/Homer | 1e-58 | -1.342e+02 | 0.0000 | 2694.0 | 6.74% | 1955.8 | 4.90% | motif file (matrix) | svg |
| 43 | T C A G C T A G C T A G A C T G A C T G G A T C A C T G A C T G C T A G C T A G A G T C G A T C | KLF1(Zf)/HUDEP2-KLF1-CutnRun(GSE136251)/Homer | 1e-58 | -1.340e+02 | 0.0000 | 5065.0 | 12.66% | 4049.8 | 10.14% | motif file (matrix) | svg |
| 44 | A T G C A G T C C T G A A G T C C G A T A C G T A G T C A G T C A C G T A T C G G A C T A C G T | Etv2(ETS)/ES-ER71-ChIP-Seq(GSE59402)/Homer | 1e-57 | -1.314e+02 | 0.0000 | 4772.0 | 11.93% | 3794.6 | 9.51% | motif file (matrix) | svg |
| 45 | C G T A C T G A A C T G A G C T A G T C G A T C G A T C G C A T C T G A C T A G C T A G T A C G T C G A T G C A G C A T | EBF2(EBF)/BrownAdipose-EBF2-ChIP-Seq(GSE97114)/Homer | 1e-56 | -1.292e+02 | 0.0000 | 4443.0 | 11.11% | 3506.2 | 8.78% | motif file (matrix) | svg |
| 46 | A G T C C T A G C T A G A G T C G A T C G T A C A G T C C T A G A G T C A G T C A G T C G T A C | Sp2(Zf)/HEK293-Sp2.eGFP-ChIP-Seq(Encode)/Homer | 1e-54 | -1.264e+02 | 0.0000 | 7641.0 | 19.11% | 6449.8 | 16.16% | motif file (matrix) | svg |
| 47 | T C G A T C G A T A G C G T A C T C A G T A C G C G T A C G T A T C A G A G C T | GABPA(ETS)/Jurkat-GABPa-ChIP-Seq(GSE17954)/Homer | 1e-53 | -1.221e+02 | 0.0000 | 4590.0 | 11.48% | 3664.0 | 9.18% | motif file (matrix) | svg |
| 48 | C G T A G A C T C G A T A T C G G T A C G C A T C A T G C G T A T A C G G C A T G T A C C G T A C A T G A T G C G C T A C T A G G C A T G C A T G C A T G A C T | MafB(bZIP)/BMM-Mafb-ChIP-Seq(GSE75722)/Homer | 1e-52 | -1.211e+02 | 0.0000 | 2494.0 | 6.24% | 1817.2 | 4.55% | motif file (matrix) | svg |
| 49 | T A C G C T A G A T G C G A T C G T A C A G T C C T A G A G T C A G T C A G T C G T A C A G T C | Sp1(Zf)/Promoter/Homer | 1e-52 | -1.208e+02 | 0.0000 | 1844.0 | 4.61% | 1269.2 | 3.18% | motif file (matrix) | svg |
| 50 | A G T C A C G T A C T G A G C T A C G T A C G T G T C A A G T C | Foxo1(Forkhead)/RAW-Foxo1-ChIP-Seq(Fan\_et\_al.)/Homer | 1e-52 | -1.200e+02 | 0.0000 | 8605.0 | 21.52% | 7383.4 | 18.50% | motif file (matrix) | svg |
| 51 | C T G A T C A G C A G T C T A G A C T G C T A G G A T C A T C G A C T G C T G A T C A G G A T C | Sp5(Zf)/mES-Sp5.Flag-ChIP-Seq(GSE72989)/Homer | 1e-51 | -1.189e+02 | 0.0000 | 5418.0 | 13.55% | 4427.8 | 11.09% | motif file (matrix) | svg |
| 52 | C G A T G A C T C G A T T C A G G A C T A C G T C A G T C T G A G A C T G A C T A G C T C G A T A C T G A T C G G T A C G C T A | NF1:FOXA1(CTF,Forkhead)/LNCAP-FOXA1-ChIP-Seq(GSE27824)/Homer | 1e-49 | -1.144e+02 | 0.0000 | 342.0 | 0.86% | 135.1 | 0.34% | motif file (matrix) | svg |
| 53 | A T G C A T C G T A C G A G C T A T C G C T G A A G T C C T A G A G C T A T G C C T G A A T G C | CRE(bZIP)/Promoter/Homer | 1e-46 | -1.078e+02 | 0.0000 | 1247.0 | 3.12% | 807.6 | 2.02% | motif file (matrix) | svg |
| 54 | T C G A C T G A T A G C T G A C T C A G T C A G C G T A C G T A T C A G A G C T | ETV1(ETS)/GIST48-ETV1-ChIP-Seq(GSE22441)/Homer | 1e-46 | -1.062e+02 | 0.0000 | 6844.0 | 17.11% | 5800.3 | 14.53% | motif file (matrix) | svg |
| 55 | A G C T T G A C C G A T C G A T C T A G A C G T C A G T C A G T G C T A A G T C | FOXK1(Forkhead)/HEK293-FOXK1-ChIP-Seq(GSE51673)/Homer | 1e-46 | -1.060e+02 | 0.0000 | 4371.0 | 10.93% | 3525.7 | 8.83% | motif file (matrix) | svg |
| 56 | C T A G T C G A C G A T C T A G G C A T C A G T C T A G G A T C C G T A G T C A | CEBP:AP1(bZIP)/ThioMac-CEBPb-ChIP-Seq(GSE21512)/Homer | 1e-45 | -1.055e+02 | 0.0000 | 3093.0 | 7.73% | 2383.9 | 5.97% | motif file (matrix) | svg |
| 57 | T G C A A G C T C A T G C G T A A G C T A C T G G A T C G T C A C G T A A G C T | Atf4(bZIP)/MEF-Atf4-ChIP-Seq(GSE35681)/Homer | 1e-45 | -1.039e+02 | 0.0000 | 1191.0 | 2.98% | 769.8 | 1.93% | motif file (matrix) | svg |
| 58 | T C G A A G C T A C G T A C G T A G T C A G T C A C G T A T C G G A C T A T C G | EWS:ERG-fusion(ETS)/CADO\_ES1-EWS:ERG-ChIP-Seq(SRA014231)/Homer | 1e-44 | -1.027e+02 | 0.0000 | 3476.0 | 8.69% | 2732.5 | 6.84% | motif file (matrix) | svg |
| 59 | A G T C C G A T A C T G A T C G T G A C G C T A C A T G A T C G T G A C C G A T A C T G T A G C G T A C G T C A | Tlx?(NR)/NPC-H3K4me1-ChIP-Seq(GSE16256)/Homer | 1e-43 | -1.010e+02 | 0.0000 | 2312.0 | 5.78% | 1714.5 | 4.29% | motif file (matrix) | svg |
| 60 | C T G A T A G C T G A C T C A G C T A G G T C A C G T A T C A G A G C T T C A G | ETV4(ETS)/HepG2-ETV4-ChIP-Seq(ENCODE)/Homer | 1e-43 | -1.003e+02 | 0.0000 | 5691.0 | 14.23% | 4757.1 | 11.92% | motif file (matrix) | svg |
| 61 | G A C T C G A T C T G A G T C A G A C T C G A T T C G A C G T A G C T A G C T A T G A C G T A C C G T A A C T G T G C A C G A T A C T G A C G T | Pitx1:Ebox(Homeobox,bHLH)/Hindlimb-Pitx1-ChIP-Seq(GSE41591)/Homer | 1e-43 | -9.989e+01 | 0.0000 | 845.0 | 2.11% | 504.7 | 1.26% | motif file (matrix) | svg |
| 62 | C T A G G T A C A G T C T G C A A G T C C T G A A G T C A G T C A G T C G C T A | Klf4(Zf)/mES-Klf4-ChIP-Seq(GSE11431)/Homer | 1e-43 | -9.984e+01 | 0.0000 | 2182.0 | 5.46% | 1605.5 | 4.02% | motif file (matrix) | svg |
| 63 | A G T C T A G C G A C T A C G T C T A G A C G T A C G T A C G T C T G A A G T C G C T A G A C T C G T A C T A G A C T G | Foxa3(Forkhead)/Liver-Foxa3-ChIP-Seq(GSE77670)/Homer | 1e-41 | -9.510e+01 | 0.0000 | 1395.0 | 3.49% | 952.6 | 2.39% | motif file (matrix) | svg |
| 64 | C G T A C T A G A C T G A C T G G A C T C T A G C A G T C T A G C A T G G A T C | KLF5(Zf)/LoVo-KLF5-ChIP-Seq(GSE49402)/Homer | 1e-41 | -9.469e+01 | 0.0000 | 6775.0 | 16.94% | 5792.3 | 14.51% | motif file (matrix) | svg |
| 65 | C T G A C G A T C T A G C G T A A G C T C G A T C A G T C T G A G A C T C T A G C T A G A T G C | PBX2(Homeobox)/K562-PBX2-ChIP-Seq(Encode)/Homer | 1e-39 | -9.174e+01 | 0.0000 | 3474.0 | 8.69% | 2769.4 | 6.94% | motif file (matrix) | svg |
| 66 | A T G C G A C T A C G T C T A G A C G T A C G T A C G T C T G A G A T C G C T A A G C T C G T A | Foxa2(Forkhead)/Liver-Foxa2-ChIP-Seq(GSE25694)/Homer | 1e-39 | -9.138e+01 | 0.0000 | 3389.0 | 8.47% | 2694.9 | 6.75% | motif file (matrix) | svg |
| 67 | A T G C T A C G A G T C G A C T T G C A C T G A G A C T A C G T C T G A T C A G | LHX9(Homeobox)/Hct116-LHX9.V5-ChIP-Seq(GSE116822)/Homer | 1e-39 | -9.025e+01 | 0.0000 | 5881.0 | 14.71% | 4980.1 | 12.48% | motif file (matrix) | svg |
| 68 | T G C A C T G A A T G C G T C A A C T G A C T G C G T A C G T A C T A G A G C T | Ets1-distal(ETS)/CD4+-PolII-ChIP-Seq(Barski\_et\_al.)/Homer | 1e-38 | -8.980e+01 | 0.0000 | 1656.0 | 4.14% | 1182.7 | 2.96% | motif file (matrix) | svg |
| 69 | T C G A G C A T A C G T C T A G G T A C T C G A G C A T T G A C T C G A A C G T | Chop(bZIP)/MEF-Chop-ChIP-Seq(GSE35681)/Homer | 1e-38 | -8.950e+01 | 0.0000 | 932.0 | 2.33% | 588.5 | 1.47% | motif file (matrix) | svg |
| 70 | G A T C C T G A A G T C C G A T C G A T G A T C A G T C A C T G A T C G A G C T | Elk4(ETS)/Hela-Elk4-ChIP-Seq(GSE31477)/Homer | 1e-38 | -8.885e+01 | 0.0000 | 2921.0 | 7.30% | 2285.8 | 5.73% | motif file (matrix) | svg |
| 71 | C A T G A G T C A G C T C G T A C G A T C G A T G C A T G C A T C G A T C T G A C A T G T G A C | Mef2d(MADS)/Retina-Mef2d-ChIP-Seq(GSE61391)/Homer | 1e-38 | -8.784e+01 | 0.0000 | 682.0 | 1.71% | 397.4 | 1.00% | motif file (matrix) | svg |
| 72 | C T A G A T C G G A C T A G T C A G T C A G T C G A C T C T G A A C T G C T A G A C T G C T G A | EBF(EBF)/proBcell-EBF-ChIP-Seq(GSE21978)/Homer | 1e-37 | -8.723e+01 | 0.0000 | 1108.0 | 2.77% | 733.4 | 1.84% | motif file (matrix) | svg |
| 73 | T C A G G A C T C A G T C T G A A G C T C T A G G A C T T G C A C T G A A G T C | HLF(bZIP)/HSC-HLF.Flag-ChIP-Seq(GSE69817)/Homer | 1e-37 | -8.666e+01 | 0.0000 | 3149.0 | 7.87% | 2496.8 | 6.25% | motif file (matrix) | svg |
| 74 | G C A T G C A T C T G A A C G T C T G A A C G T C G T A C G T A C G T A A G T C G T C A G T C A | Foxf1(Forkhead)/Lung-Foxf1-ChIP-Seq(GSE77951)/Homer | 1e-36 | -8.374e+01 | 0.0000 | 3728.0 | 9.32% | 3029.5 | 7.59% | motif file (matrix) | svg |
| 75 | G A T C T C G A A G T C C G A T C G A T A G T C A T G C A C T G A T C G G A C T | Elk1(ETS)/Hela-Elk1-ChIP-Seq(GSE31477)/Homer | 1e-35 | -8.217e+01 | 0.0000 | 2904.0 | 7.26% | 2293.4 | 5.74% | motif file (matrix) | svg |
| 76 | A G C T A G T C A G T C A C G T C T A G A C G T A C G T A C G T C G T A A G T C G A T C C G T A | FOXP1(Forkhead)/H9-FOXP1-ChIP-Seq(GSE31006)/Homer | 1e-34 | -7.945e+01 | 0.0000 | 1899.0 | 4.75% | 1417.5 | 3.55% | motif file (matrix) | svg |
| 77 | A G T C C G A T C T G A C G T A A C G T C A G T T C A G T G A C | Isl1(Homeobox)/Neuron-Isl1-ChIP-Seq(GSE31456)/Homer | 1e-34 | -7.939e+01 | 0.0000 | 8157.0 | 20.40% | 7181.9 | 17.99% | motif file (matrix) | svg |
| 78 | T A C G T A G C G C T A C G A T C T A G A C G T C A G T C A G T G C T A A G T C G T C A G C A T | FOXK2(Forkhead)/U2OS-FOXK2-ChIP-Seq(E-MTAB-2204)/Homer | 1e-34 | -7.833e+01 | 0.0000 | 2633.0 | 6.58% | 2066.0 | 5.18% | motif file (matrix) | svg |
| 79 | A T G C C T G A A T C G T A C G A G T C C G A T T C A G C G A T C T A G A G C T G T C A G T C A C G T A A G T C C G T A T A C G C T G A | Fox:Ebox(Forkhead,bHLH)/Panc1-Foxa2-ChIP-Seq(GSE47459)/Homer | 1e-33 | -7.716e+01 | 0.0000 | 4598.0 | 11.50% | 3854.5 | 9.66% | motif file (matrix) | svg |
| 80 | T C A G A G C T A T G C C G T A A G C T T C A G C A G T A C T G C T G A A G T C | MITF(bHLH)/MastCells-MITF-ChIP-Seq(GSE48085)/Homer | 1e-33 | -7.700e+01 | 0.0000 | 4416.0 | 11.04% | 3687.8 | 9.24% | motif file (matrix) | svg |
| 81 | G T A C C A G T A C T G A C T G A C T G G A T C A C T G A C G T A C T G A C T G A G T C G A T C | KLF6(Zf)/PDAC-KLF6-ChIP-Seq(GSE64557)/Homer | 1e-33 | -7.652e+01 | 0.0000 | 5324.0 | 13.31% | 4530.6 | 11.35% | motif file (matrix) | svg |
| 82 | C T A G A G C T G A C T C A T G A G T C A G T C G T C A C A G T C T A G T C A G G T A C C T G A T C G A G A T C T G A C | Rfx2(HTH)/LoVo-RFX2-ChIP-Seq(GSE49402)/Homer | 1e-32 | -7.549e+01 | 0.0000 | 617.0 | 1.54% | 365.1 | 0.91% | motif file (matrix) | svg |
| 83 | C G T A T A G C T A G C T G C A A C T G C T A G C G T A C G T A T C A G G A C T | EHF(ETS)/LoVo-EHF-ChIP-Seq(GSE49402)/Homer | 1e-32 | -7.502e+01 | 0.0000 | 6006.0 | 15.02% | 5175.7 | 12.97% | motif file (matrix) | svg |
| 84 | T C A G A G C T A C G T A C G T G T A C G A T C C G T A C T A G C A T G G T C A C G T A T C G A | STAT4(Stat)/CD4-Stat4-ChIP-Seq(GSE22104)/Homer | 1e-32 | -7.493e+01 | 0.0000 | 3967.0 | 9.92% | 3284.0 | 8.23% | motif file (matrix) | svg |
| 85 | T A C G T C A G G A T C G T A C T C G A G A C T G C T A G C T A G C T A C G T A G A T C G T C A | CDX4(Homeobox)/ZebrafishEmbryos-Cdx4.Myc-ChIP-Seq(GSE48254)/Homer | 1e-31 | -7.325e+01 | 0.0000 | 3356.0 | 8.39% | 2734.8 | 6.85% | motif file (matrix) | svg |
| 86 | A G T C A T C G C T A G A G C T G A C T C T A G A G T C A G T C G C T A C A G T T C A G T C A G G A T C C T G A T C G A G A T C | RFX(HTH)/K562-RFX3-ChIP-Seq(SRA012198)/Homer | 1e-31 | -7.300e+01 | 0.0000 | 557.0 | 1.39% | 323.0 | 0.81% | motif file (matrix) | svg |
| 87 | C A T G A C G T A G T C G A T C G A T C G A T C G C T A C T A G C T A G C T A G T C A G T C G A | EBF1(EBF)/Near-E2A-ChIP-Seq(GSE21512)/Homer | 1e-31 | -7.231e+01 | 0.0000 | 4777.0 | 11.94% | 4043.1 | 10.13% | motif file (matrix) | svg |
| 88 | C T G A A G T C C G A T A G C T A T G C G T A C A C G T A T C G C A G T G C A T | Elf4(ETS)/BMDM-Elf4-ChIP-Seq(GSE88699)/Homer | 1e-30 | -7.094e+01 | 0.0000 | 5058.0 | 12.65% | 4311.1 | 10.80% | motif file (matrix) | svg |
| 89 | C G T A G C T A C G A T C T A G A C G T G T C A C G T A C G T A A G T C C G T A T G C A T A C G | FoxL2(Forkhead)/Ovary-FoxL2-ChIP-Seq(GSE60858)/Homer | 1e-30 | -6.955e+01 | 0.0000 | 3383.0 | 8.46% | 2774.4 | 6.95% | motif file (matrix) | svg |
| 90 | C G T A C G T A A G C T G A C T T G C A G T C A A C G T A G C T C T G A T C A G | Lhx3(Homeobox)/Neuron-Lhx3-ChIP-Seq(GSE31456)/Homer | 1e-30 | -6.945e+01 | 0.0000 | 6611.0 | 16.53% | 5776.8 | 14.47% | motif file (matrix) | svg |
| 91 | C T A G T A C G G A T C G T A C C T G A A G C T T G C A G C T A C G T A G C A T G A T C G C T A | Hoxc9(Homeobox)/Ainv15-Hoxc9-ChIP-Seq(GSE21812)/Homer | 1e-30 | -6.935e+01 | 0.0000 | 1970.0 | 4.93% | 1509.7 | 3.78% | motif file (matrix) | svg |
| 92 | G C T A T C G A C G T A C T A G A G C T G T C A G T C A C G T A A G T C C G T A | FOXA1(Forkhead)/LNCAP-FOXA1-ChIP-Seq(GSE27824)/Homer | 1e-29 | -6.902e+01 | 0.0000 | 5014.0 | 12.54% | 4280.5 | 10.72% | motif file (matrix) | svg |
| 93 | C T G A A T G C C G T A A C G T A G T C A G T C A C G T A C T G A T C G G C A T | SPDEF(ETS)/VCaP-SPDEF-ChIP-Seq(SRA014231)/Homer | 1e-29 | -6.855e+01 | 0.0000 | 4919.0 | 12.30% | 4194.8 | 10.51% | motif file (matrix) | svg |
| 94 | C G A T C T A G A C G T A C G T A C G T C G T A A G C T C G A T A G C T C G T A C T A G T A G C | FoxD3(forkhead)/ZebrafishEmbryo-Foxd3.biotin-ChIP-seq(GSE106676)/Homer | 1e-29 | -6.802e+01 | 0.0000 | 3679.0 | 9.20% | 3051.4 | 7.64% | motif file (matrix) | svg |
| 95 | T G C A T C G A T A G C G T A C T C A G C T A G G T C A G C T A T C A G G A C T | ETS(ETS)/Promoter/Homer | 1e-29 | -6.739e+01 | 0.0000 | 1795.0 | 4.49% | 1362.2 | 3.41% | motif file (matrix) | svg |
| 96 | C G A T T G C A T G C A G A T C C G T A A C T G T G A C G A C T C A T G A C T G | Tcf21(bHLH)/ArterySmoothMuscle-Tcf21-ChIP-Seq(GSE61369)/Homer | 1e-28 | -6.670e+01 | 0.0000 | 4503.0 | 11.26% | 3817.7 | 9.56% | motif file (matrix) | svg |
| 97 | T G A C C G A T A C T G A C T G A C T G G A C T A C T G A C G T A C T G A C T G G A T C G A T C | EKLF(Zf)/Erythrocyte-Klf1-ChIP-Seq(GSE20478)/Homer | 1e-27 | -6.417e+01 | 0.0000 | 1125.0 | 2.81% | 796.1 | 1.99% | motif file (matrix) | svg |
| 98 | T A C G C T G A T C G A C G A T C T A G C T A G T C G A C T G A T C G A T C G A C G T A T C G A G C A T C A T G C G T A T A C G G C A T T G A C C G T A A G C T | NFAT:AP1(RHD,bZIP)/Jurkat-NFATC1-ChIP-Seq(Jolma\_et\_al.)/Homer | 1e-27 | -6.381e+01 | 0.0000 | 872.0 | 2.18% | 587.4 | 1.47% | motif file (matrix) | svg |
| 99 | C A T G G T A C G A C T G C T A C G T A C G T A C G T A G C T A G A C T C T G A T C A G G T A C | Mef2c(MADS)/GM12878-Mef2c-ChIP-Seq(GSE32465)/Homer | 1e-27 | -6.328e+01 | 0.0000 | 1449.0 | 3.62% | 1074.3 | 2.69% | motif file (matrix) | svg |
| 100 | A G T C G A C T C A G T G T A C A G T C A T C G T C A G A C T G G T C A C G T A | Stat3(Stat)/mES-Stat3-ChIP-Seq(GSE11431)/Homer | 1e-27 | -6.275e+01 | 0.0000 | 2277.0 | 5.69% | 1803.9 | 4.52% | motif file (matrix) | svg |
| 101 | G C T A T C G A C G T A C T A G A G C T G T C A G T C A C G T A A G T C C G T A | FOXA1(Forkhead)/MCF7-FOXA1-ChIP-Seq(GSE26831)/Homer | 1e-27 | -6.246e+01 | 0.0000 | 4060.0 | 10.15% | 3428.8 | 8.59% | motif file (matrix) | svg |
| 102 | C T G A T G C A T A G C T G A C T A C G T C A G C T G A G C T A T C A G G A C T | ELF1(ETS)/Jurkat-ELF1-ChIP-Seq(SRA014231)/Homer | 1e-26 | -6.166e+01 | 0.0000 | 2670.0 | 6.68% | 2160.5 | 5.41% | motif file (matrix) | svg |
| 103 | T G C A C T G A A G T C G T C A A C T G A C T G C G T A C G T A C T G A A G C T | EWS:FLI1-fusion(ETS)/SK\_N\_MC-EWS:FLI1-ChIP-Seq(SRA014231)/Homer | 1e-26 | -6.125e+01 | 0.0000 | 2965.0 | 7.41% | 2429.3 | 6.09% | motif file (matrix) | svg |
| 104 | A T G C A G T C G C A T A G C T A C G T T C A G C G A T A G C T G A T C A T C G | Sox10(HMG)/SciaticNerve-Sox3-ChIP-Seq(GSE35132)/Homer | 1e-26 | -6.062e+01 | 0.0000 | 7038.0 | 17.60% | 6236.6 | 15.62% | motif file (matrix) | svg |
| 105 | A T C G A T C G A T G C G A C T T C G A C G T A G C A T A G C T C T G A T A C G | DLX2(Homeobox)/BasalGanglia-Dlx2-ChIP-seq(GSE124936)/Homer | 1e-26 | -6.030e+01 | 0.0000 | 5918.0 | 14.80% | 5177.4 | 12.97% | motif file (matrix) | svg |
| 106 | A G C T G T C A C G T A A C G T A C G T C T G A T C A G A T G C | Lhx2(Homeobox)/HFSC-Lhx2-ChIP-Seq(GSE48068)/Homer | 1e-25 | -5.945e+01 | 0.0000 | 4214.0 | 10.54% | 3586.3 | 8.98% | motif file (matrix) | svg |
| 107 | A C T G C A T G G C T A T C G A G C T A A G C T A G C T G T A C A G T C T G A C | NFkB-p65-Rel(RHD)/ThioMac-LPS-Expression(GSE23622)/Homer | 1e-25 | -5.909e+01 | 0.0000 | 327.0 | 0.82% | 171.3 | 0.43% | motif file (matrix) | svg |
| 108 | A T G C T C G A A G T C A G C T A C G T G T A C A G T C G C T A C T A G C A T G G T C A C T G A T C A G A G T C | Stat3+il21(Stat)/CD4-Stat3-ChIP-Seq(GSE19198)/Homer | 1e-25 | -5.807e+01 | 0.0000 | 3065.0 | 7.66% | 2534.1 | 6.35% | motif file (matrix) | svg |
| 109 | C A T G A G T C G A C T C G T A C G A T G C A T G A C T G C A T C G A T C T A G C A T G T G A C | Mef2b(MADS)/HEK293-Mef2b.V5-ChIP-Seq(GSE67450)/Homer | 1e-24 | -5.640e+01 | 0.0000 | 2764.0 | 6.91% | 2267.9 | 5.68% | motif file (matrix) | svg |
| 110 | A C T G C A G T A C G T C G T A C G T A A C G T A C T G C T G A | Nkx6.1(Homeobox)/Islet-Nkx6.1-ChIP-Seq(GSE40975)/Homer | 1e-24 | -5.614e+01 | 0.0000 | 10136.0 | 25.34% | 9243.0 | 23.15% | motif file (matrix) | svg |
| 111 | A C G T C T A G A G C T A C G T A C G T C T G A A G T C G A C T A G C T C G T A | FOXM1(Forkhead)/MCF7-FOXM1-ChIP-Seq(GSE72977)/Homer | 1e-23 | -5.486e+01 | 0.0000 | 4294.0 | 10.74% | 3685.9 | 9.23% | motif file (matrix) | svg |
| 112 | G A C T C A G T A G C T C G A T A G T C G A T C A G T C C G T A A T G C T C A G | Rbpj1(?)/Panc1-Rbpj1-ChIP-Seq(GSE47459)/Homer | 1e-23 | -5.385e+01 | 0.0000 | 6640.0 | 16.60% | 5903.2 | 14.79% | motif file (matrix) | svg |
| 113 | C A T G C T A G A G C T G A C T C A T G A G T C G A T C G C T A C G A T C T A G T C A G G T A C C T G A T C G A | X-box(HTH)/NPC-H3K4me1-ChIP-Seq(GSE16256)/Homer | 1e-23 | -5.308e+01 | 0.0000 | 638.0 | 1.60% | 418.9 | 1.05% | motif file (matrix) | svg |
| 114 | C G T A T G A C T A G C T G C A A C T G A C T G C G T A C G T A T C A G G A C T | ELF3(ETS)/PDAC-ELF3-ChIP-Seq(GSE64557)/Homer | 1e-22 | -5.202e+01 | 0.0000 | 3431.0 | 8.58% | 2899.8 | 7.26% | motif file (matrix) | svg |
| 115 | C T A G T A G C A G T C G T C A C T G A A C G T C G T A C G T A C G T A G C T A | Hoxd13(Homeobox)/ChickenMSG-Hoxd13.Flag-ChIP-Seq(GSE86088)/Homer | 1e-22 | -5.163e+01 | 0.0000 | 6157.0 | 15.40% | 5459.2 | 13.68% | motif file (matrix) | svg |
| 116 | C G A T A C G T A C G T A C G T C G T A A G C T C A G T C T A G A T C G A C T G | HOXB13(Homeobox)/ProstateTumor-HOXB13-ChIP-Seq(GSE56288)/Homer | 1e-21 | -5.046e+01 | 0.0000 | 4109.0 | 10.27% | 3537.0 | 8.86% | motif file (matrix) | svg |
| 117 | G A C T A G T C C G A T A C T G C T G A T G A C G T A C C G T A A T C G G C A T C T G A C T A G | Bcl11a(Zf)/HSPC-BCL11A-ChIP-Seq(GSE104676)/Homer | 1e-21 | -5.037e+01 | 0.0000 | 3113.0 | 7.78% | 2614.9 | 6.55% | motif file (matrix) | svg |
| 118 | A T C G A G C T C T G A C T A G A C T G A C G T G T A C G C T A A T G C A C G T C T A G C A T G T A C G C G A T A T G C C G T A | Reverb(NR),DR2/RAW-Reverba.biotin-ChIP-Seq(GSE45914)/Homer | 1e-21 | -4.842e+01 | 0.0000 | 727.0 | 1.82% | 500.9 | 1.25% | motif file (matrix) | svg |
| 119 | A G C T C A T G G C A T G A T C T G C A C T A G G A T C A C G T | Tgif2(Homeobox)/mES-Tgif2-ChIP-Seq(GSE55404)/Homer | 1e-20 | -4.773e+01 | 0.0000 | 14670.0 | 36.68% | 13743.6 | 34.43% | motif file (matrix) | svg |
| 120 | T A C G A T C G G A T C G T C A C T G A G C A T C G A T G C T A T C G A G C T A | Unknown(Homeobox)/Limb-p300-ChIP-Seq/Homer | 1e-19 | -4.576e+01 | 0.0000 | 2485.0 | 6.21% | 2060.6 | 5.16% | motif file (matrix) | svg |
| 121 | T C A G C G T A A G T C A G C T C G T A A G T C C T G A C G T A A G T C G C A T A G T C A G T C A G T C C T G A A C T G T G C A T C G A C A T G A T C G G A T C | Ronin(THAP)/ES-Thap11-ChIP-Seq(GSE51522)/Homer | 1e-19 | -4.560e+01 | 0.0000 | 194.0 | 0.49% | 92.4 | 0.23% | motif file (matrix) | svg |
| 122 | T C A G G C T A T C A G C A G T T G A C G T C A A G T C A T C G T G C A G T A C C A G T G A T C | Npas4(bHLH)/Neuron-Npas4-ChIP-Seq(GSE127793)/Homer | 1e-19 | -4.556e+01 | 0.0000 | 3103.0 | 7.76% | 2629.4 | 6.59% | motif file (matrix) | svg |
| 123 | T C A G T A C G G A T C G T A C T C G A C G A T C T G A C G T A G C T A C G T A | Hoxd11(Homeobox)/ChickenMSG-Hoxd11.Flag-ChIP-Seq(GSE86088)/Homer | 1e-19 | -4.499e+01 | 0.0000 | 9500.0 | 23.75% | 8720.2 | 21.84% | motif file (matrix) | svg |
| 124 | T C A G T G A C G T A C T G C A G T A C C T A G G T A C A T G C A G T C G T C A A G T C G A C T | Klf9(Zf)/GBM-Klf9-ChIP-Seq(GSE62211)/Homer | 1e-19 | -4.455e+01 | 0.0000 | 2242.0 | 5.61% | 1844.1 | 4.62% | motif file (matrix) | svg |
| 125 | C G T A C A T G A G T C G A C T T G C A C G T A A C G T A C G T C T G A T C A G | Lhx1(Homeobox)/EmbryoCarcinoma-Lhx1-ChIP-Seq(GSE70957)/Homer | 1e-18 | -4.315e+01 | 0.0000 | 4242.0 | 10.61% | 3705.6 | 9.28% | motif file (matrix) | svg |
| 126 | G T C A T G C A G C T A A G T C C G T A A C T G T G A C G C A T T C A G C A G T | Ap4(bHLH)/AML-Tfap4-ChIP-Seq(GSE45738)/Homer | 1e-18 | -4.274e+01 | 0.0000 | 5721.0 | 14.31% | 5107.8 | 12.80% | motif file (matrix) | svg |
| 127 | T C G A G C A T A T G C C T G A A T G C T A G C A G T C G T A C T C G A A G C T | Srebp1a(bHLH)/HepG2-Srebp1a-ChIP-Seq(GSE31477)/Homer | 1e-18 | -4.222e+01 | 0.0000 | 904.0 | 2.26% | 664.5 | 1.66% | motif file (matrix) | svg |
| 128 | C T A G T C A G C A G T T C A G A C T G A C T G G A T C C T A G A C T G C T A G T C A G A T G C | KLF14(Zf)/HEK293-KLF14.GFP-ChIP-Seq(GSE58341)/Homer | 1e-18 | -4.211e+01 | 0.0000 | 8472.0 | 21.18% | 7751.4 | 19.42% | motif file (matrix) | svg |
| 129 | C G T A T A C G T C G A A C T G A C T G C G T A C G T A T A C G A G C T T A C G | PU.1(ETS)/ThioMac-PU.1-ChIP-Seq(GSE21512)/Homer | 1e-18 | -4.208e+01 | 0.0000 | 2405.0 | 6.01% | 2004.1 | 5.02% | motif file (matrix) | svg |
| 130 | C T A G T A C G G A T C G T A C G C T A A G C T A G C T G T C A T C G A T A G C | Nanog(Homeobox)/mES-Nanog-ChIP-Seq(GSE11724)/Homer | 1e-17 | -4.049e+01 | 0.0000 | 17701.0 | 44.26% | 16814.4 | 42.12% | motif file (matrix) | svg |
| 131 | C G T A C A T G C A T G A C T G C T A G T C G A G C A T C G A T A G C T A G T C G A T C G T A C | NFkB-p65(RHD)/GM12787-p65-ChIP-Seq(GSE19485)/Homer | 1e-17 | -4.027e+01 | 0.0000 | 2032.0 | 5.08% | 1672.5 | 4.19% | motif file (matrix) | svg |
| 132 | T C A G A C G T T C G A T A G C A G T C C G T A A C T G G T A C A C G T A C T G A T C G A G T C | Atoh1(bHLH)/Cerebellum-Atoh1-ChIP-Seq(GSE22111)/Homer | 1e-16 | -3.885e+01 | 0.0000 | 4921.0 | 12.30% | 4375.5 | 10.96% | motif file (matrix) | svg |
| 133 | T C G A C A G T A C T G A G C T C G T A C G T A A C G T A C G T C T G A T A G C | Dlx3(Homeobox)/Kerainocytes-Dlx3-ChIP-Seq(GSE89884)/Homer | 1e-16 | -3.858e+01 | 0.0000 | 2247.0 | 5.62% | 1876.2 | 4.70% | motif file (matrix) | svg |
| 134 | G A C T G C A T C T A G C G A T G A T C T C G A C A T G G A T C | Tgif1(Homeobox)/mES-Tgif1-ChIP-Seq(GSE55404)/Homer | 1e-16 | -3.819e+01 | 0.0000 | 13768.0 | 34.43% | 12955.5 | 32.45% | motif file (matrix) | svg |
| 135 | C A G T T C A G A G C T G A C T A C G T A G T C G A T C G A C T C T G A A C T G G A T C C G T A C T G A A G T C G T A C | Rfx6(HTH)/Min6b1-Rfx6.HA-ChIP-Seq(GSE62844)/Homer | 1e-16 | -3.780e+01 | 0.0000 | 4892.0 | 12.23% | 4355.8 | 10.91% | motif file (matrix) | svg |
| 136 | A C T G T C A G A G C T G A C T C A T G A G T C A G T C G C T A C G A T C T A G T C A G G T A C C T G A T C G A | Rfx1(HTH)/NPC-H3K4me1-ChIP-Seq(GSE16256)/Homer | 1e-16 | -3.762e+01 | 0.0000 | 1025.0 | 2.56% | 782.8 | 1.96% | motif file (matrix) | svg |
| 137 | A G T C A G T C C G A T A C G T A C G T A C T G A C G T A G C T A G T C A G T C | Sox4(HMG)/proB-Sox4-ChIP-Seq(GSE50066)/Homer | 1e-15 | -3.548e+01 | 0.0000 | 3493.0 | 8.73% | 3050.3 | 7.64% | motif file (matrix) | svg |
| 138 | C G T A T A G C A G T C C T A G C A G T C T A G C T G A G T A C G C A T T C G A C G T A G C A T A G C T C T A G T C G A | PAX3:FKHR-fusion(Paired,Homeobox)/Rh4-PAX3:FKHR-ChIP-Seq(GSE19063)/Homer | 1e-14 | -3.303e+01 | 0.0000 | 879.0 | 2.20% | 669.6 | 1.68% | motif file (matrix) | svg |
| 139 | A T G C T A C G A G C T T G C A C G T A C G A T A C G T C T G A | DLX5(Homeobox)/BasalGanglia-Dlx5-ChIP-seq(GSE124936)/Homer | 1e-14 | -3.283e+01 | 0.0000 | 2756.0 | 6.89% | 2377.1 | 5.95% | motif file (matrix) | svg |
| 140 | C A G T C G A T G C A T G C A T G T C A A G C T C A T G C T A G A T G C G T A C | Hoxa11(Homeobox)/ChickenMSG-Hoxa11.Flag-ChIP-Seq(GSE86088)/Homer | 1e-14 | -3.251e+01 | 0.0000 | 8980.0 | 22.45% | 8334.5 | 20.88% | motif file (matrix) | svg |
| 141 | T A G C G A T C G T A C C T G A G C A T C T G A C G T A T G C A C G T A G A T C | Hoxa13(Homeobox)/ChickenMSG-Hoxa13.Flag-ChIP-Seq(GSE86088)/Homer | 1e-14 | -3.250e+01 | 0.0000 | 9650.0 | 24.13% | 8986.6 | 22.51% | motif file (matrix) | svg |
| 142 | A T G C G A T C C G A T A C G T A C G T A C T G C A G T A G C T | Sox3(HMG)/NPC-Sox3-ChIP-Seq(GSE33059)/Homer | 1e-13 | -3.156e+01 | 0.0000 | 7251.0 | 18.13% | 6668.1 | 16.70% | motif file (matrix) | svg |
| 143 | T C G A T G A C G T A C C G T A C A G T T G A C A C G T A C T G A G C T A G C T | NeuroG2(bHLH)/Fibroblast-NeuroG2-ChIP-Seq(GSE75910)/Homer | 1e-13 | -3.098e+01 | 0.0000 | 6946.0 | 17.37% | 6379.5 | 15.98% | motif file (matrix) | svg |
| 144 | A G C T G C A T A C T G A C G T A G T C A C G T C T A G T A C G | Smad3(MAD)/NPC-Smad3-ChIP-Seq(GSE36673)/Homer | 1e-13 | -3.048e+01 | 0.0000 | 13666.0 | 34.17% | 12944.6 | 32.43% | motif file (matrix) | svg |
| 145 | C G T A C T A G T C A G T C A G A G T C A T G C A G T C G C A T A G C T A C G T A T C G C G A T | Sox9(HMG)/Limb-SOX9-ChIP-Seq(GSE73225)/Homer | 1e-13 | -3.038e+01 | 0.0000 | 3761.0 | 9.40% | 3337.2 | 8.36% | motif file (matrix) | svg |
| 146 | C T A G T C G A T G A C A G T C C G T A A C T G G T A C A C G T A C T G A C T G | BHLHA15(bHLH)/NIH3T3-BHLHB8.HA-ChIP-Seq(GSE119782)/Homer | 1e-13 | -3.035e+01 | 0.0000 | 6334.0 | 15.84% | 5795.5 | 14.52% | motif file (matrix) | svg |
| 147 | T G C A T A G C G A C T T G C A T G A C T G C A C G T A A G C T A G C T A G T C A G T C G T A C | GFY(?)/Promoter/Homer | 1e-12 | -2.857e+01 | 0.0000 | 325.0 | 0.81% | 212.8 | 0.53% | motif file (matrix) | svg |
| 148 | G C T A A G T C T A C G T G C A A T C G T C A G G C T A T C G A T C A G A G C T | ELF5(ETS)/T47D-ELF5-ChIP-Seq(GSE30407)/Homer | 1e-12 | -2.851e+01 | 0.0000 | 3368.0 | 8.42% | 2979.3 | 7.46% | motif file (matrix) | svg |
| 149 | A T G C C T G A G A C T A C G T A C G T G T A C G A T C C G A T C T A G C A T G C G T A C G T A C T G A G A C T | STAT1(Stat)/HelaS3-STAT1-ChIP-Seq(GSE12782)/Homer | 1e-12 | -2.847e+01 | 0.0000 | 1188.0 | 2.97% | 959.1 | 2.40% | motif file (matrix) | svg |
| 150 | C T G A C T G A C A T G A T C G A G C T A T C G G A C T C A T G C T G A G T C A | Tbr1(T-box)/Cortex-Tbr1-ChIP-Seq(GSE71384)/Homer | 1e-12 | -2.845e+01 | 0.0000 | 5490.0 | 13.73% | 5001.1 | 12.53% | motif file (matrix) | svg |
| 151 | G T A C G A C T C G T A C T G A T C G A C G T A G C T A C A G T C T G A T A C G | Mef2a(MADS)/HL1-Mef2a.biotin-ChIP-Seq(GSE21529)/Homer | 1e-12 | -2.807e+01 | 0.0000 | 1381.0 | 3.45% | 1135.8 | 2.85% | motif file (matrix) | svg |
| 152 | C T A G C G T A G T C A C G T A A G T C G A T C A G C T C T A G C G T A A C G T G T C A G A T C | Six2(Homeobox)/NephronProgenitor-Six2-ChIP-Seq(GSE39837)/Homer | 1e-11 | -2.664e+01 | 0.0000 | 4002.0 | 10.01% | 3594.6 | 9.00% | motif file (matrix) | svg |
| 153 | C T A G T A C G G A T C G T C A T C G A A C G T T C A G C G T A C G T A C G T A | Hoxd10(Homeobox)/ChickenMSG-Hoxd10.Flag-ChIP-Seq(GSE86088)/Homer | 1e-11 | -2.651e+01 | 0.0000 | 4364.0 | 10.91% | 3940.0 | 9.87% | motif file (matrix) | svg |
| 154 | A T C G A G T C A G T C G A C T A T G C C T G A C T A G A C T G T A C G G T A C C T G A C G A T | AP-2gamma(AP2)/MCF7-TFAP2C-ChIP-Seq(GSE21234)/Homer | 1e-11 | -2.628e+01 | 0.0000 | 4497.0 | 11.24% | 4069.2 | 10.19% | motif file (matrix) | svg |
| 155 | G A C T C A G T G A T C G A T C A C G T G A T C C T G A T A C G C G T A G T C A | STAT6(Stat)/Macrophage-Stat6-ChIP-Seq(GSE38377)/Homer | 1e-10 | -2.488e+01 | 0.0000 | 2118.0 | 5.30% | 1830.3 | 4.58% | motif file (matrix) | svg |
| 156 | C T G A A C G T A C G T A C G T A G T C G A C T C G A T C T G A A C T G C G T A C G T A T C G A | STAT5(Stat)/mCD4+-Stat5-ChIP-Seq(GSE12346)/Homer | 1e-10 | -2.454e+01 | 0.0000 | 1396.0 | 3.49% | 1165.2 | 2.92% | motif file (matrix) | svg |
| 157 | C G A T C T A G T C G A A G C T C G A T C T G A C G T A A G C T A C T G C T A G A T G C G A T C | Hoxb4(Homeobox)/ES-Hoxb4-ChIP-Seq(GSE34014)/Homer | 1e-10 | -2.387e+01 | 0.0000 | 873.0 | 2.18% | 695.9 | 1.74% | motif file (matrix) | svg |
| 158 | A T C G A T G C A G T C T A G C G A C T T C G A G C T A G C A T A G C T C T G A | DLX1(Homeobox)/BasalGanglia-Dlx1-ChIP-seq(GSE124936)/Homer | 1e-10 | -2.372e+01 | 0.0000 | 4950.0 | 12.38% | 4526.7 | 11.34% | motif file (matrix) | svg |
| 159 | C T G A T C G A C G T A A T G C C G T A C G T A C G A T C T A G T C A G G A T C | Sox15(HMG)/CPA-Sox15-ChIP-Seq(GSE62909)/Homer | 1e-10 | -2.359e+01 | 0.0000 | 4278.0 | 10.70% | 3883.3 | 9.73% | motif file (matrix) | svg |
| 160 | A T G C G T A C C G T A A G C T G C A T T A C G A G C T A G C T A G T C A G C T | Sox6(HMG)/Myotubes-Sox6-ChIP-Seq(GSE32627)/Homer | 1e-10 | -2.346e+01 | 0.0000 | 6398.0 | 16.00% | 5925.5 | 14.84% | motif file (matrix) | svg |
| 161 | T C A G A G C T G T A C C G T A A C G T C G T A C G T A C G T A G C T A G A C T | Cdx2(Homeobox)/mES-Cdx2-ChIP-Seq(GSE14586)/Homer | 1e-10 | -2.314e+01 | 0.0000 | 2379.0 | 5.95% | 2085.7 | 5.22% | motif file (matrix) | svg |
| 162 | T A C G A C T G A G C T G T A C C G T A T C G A C T G A A C T G C A T G A C G T A G T C C G T A | COUP-TFII(NR)/K562-NR2F1-ChIP-Seq(Encode)/Homer | 1e-9 | -2.299e+01 | 0.0000 | 6339.0 | 15.85% | 5873.7 | 14.71% | motif file (matrix) | svg |
| 163 | G C T A C G A T C A T G A T G C A G T C A G T C G A C T T A C G T C G A C T A G A C T G T A G C | AP-2alpha(AP2)/Hela-AP2alpha-ChIP-Seq(GSE31477)/Homer | 1e-9 | -2.139e+01 | 0.0000 | 3334.0 | 8.34% | 3001.3 | 7.52% | motif file (matrix) | svg |
| 164 | T G A C G C T A T G A C C G T A T C A G G A T C C G T A C A T G C A T G C T A G C T A G C T A G | Unknown-ESC-element(?)/mES-Nanog-ChIP-Seq(GSE11724)/Homer | 1e-9 | -2.107e+01 | 0.0000 | 2556.0 | 6.39% | 2266.6 | 5.68% | motif file (matrix) | svg |
| 165 | C T A G T A C G G A T C G T C A T G C A A C G T T G C A G C T A T C G A T G C A | Hoxa9(Homeobox)/ChickenMSG-Hoxa9.Flag-ChIP-Seq(GSE86088)/Homer | 1e-8 | -2.043e+01 | 0.0000 | 10803.0 | 27.01% | 10261.0 | 25.70% | motif file (matrix) | svg |
| 166 | T C A G T C A G A C G T G T A C G C T A T C A G C T G A A C T G A C T G A G C T A G T C C G T A | EAR2(NR)/K562-NR2F6-ChIP-Seq(Encode)/Homer | 1e-8 | -2.039e+01 | 0.0000 | 5853.0 | 14.64% | 5431.8 | 13.61% | motif file (matrix) | svg |
| 167 | A G T C G A C T A G C T C G A T A T C G G C T A C G A T A T C G C G A T A C T G T A C G A C G T | Tcf7(HMG)/GM12878-TCF7-ChIP-Seq(Encode)/Homer | 1e-8 | -2.034e+01 | 0.0000 | 1455.0 | 3.64% | 1241.3 | 3.11% | motif file (matrix) | svg |
| 168 | C G T A A C T G C G T A A C G T C G T A C T G A T C A G T G C A A G C T T G A C | TRPS1(Zf)/MCF7-TRPS1-ChIP-Seq(GSE107013)/Homer | 1e-8 | -2.031e+01 | 0.0000 | 7434.0 | 18.59% | 6967.0 | 17.45% | motif file (matrix) | svg |
| 169 | T A C G T G C A A G T C C G T A A C G T T G A C A C G T A C T G A C T G G C A T | TCF4(bHLH)/SHSY5Y-TCF4-ChIP-Seq(GSE96915)/Homer | 1e-8 | -2.010e+01 | 0.0000 | 7045.0 | 17.62% | 6591.4 | 16.51% | motif file (matrix) | svg |
| 170 | C G T A A C G T A C T G G T A C C G T A A C G T C G T A C G T A A C G T A C T G A G T C C G T A A C G T C T G A G C A T | OCT:OCT-short(POU,Homeobox)/NPC-OCT6-ChIP-Seq(GSE43916)/Homer | 1e-8 | -1.973e+01 | 0.0000 | 2203.0 | 5.51% | 1943.4 | 4.87% | motif file (matrix) | svg |
| 171 | C T G A T C A G G T A C G C T A A C T G T G A C G C A T C A T G | SCL(bHLH)/HPC7-Scl-ChIP-Seq(GSE13511)/Homer | 1e-8 | -1.924e+01 | 0.0000 | 19995.0 | 50.00% | 19384.5 | 48.56% | motif file (matrix) | svg |
| 172 | T A C G A T G C G C T A A C T G C G T A A C G T C G T A C T G A T A C G T C G A | Gata4(Zf)/Heart-Gata4-ChIP-Seq(GSE35151)/Homer | 1e-7 | -1.823e+01 | 0.0000 | 3643.0 | 9.11% | 3324.2 | 8.33% | motif file (matrix) | svg |
| 173 | C T G A A G C T A C G T A C G T A G T C G A C T G A C T C T G A C T G A C T A G C G T A C G T A | STAT6(Stat)/CD4-Stat6-ChIP-Seq(GSE22104)/Homer | 1e-7 | -1.819e+01 | 0.0000 | 1997.0 | 4.99% | 1760.1 | 4.41% | motif file (matrix) | svg |
| 174 | C A G T T C A G G A T C A C T G A C G T C T A G A C T G A C T G G A C T C T A G | Egr1(Zf)/K562-Egr1-ChIP-Seq(GSE32465)/Homer | 1e-7 | -1.771e+01 | 0.0000 | 3090.0 | 7.73% | 2800.8 | 7.02% | motif file (matrix) | svg |
| 175 | C A G T A C T G C G T A G T C A C G T A A G C T G A T C A G C T A C T G C G T A A G T C G T C A A G T C A G T C A T C G | Six4(Homeobox)/MCF7-SIX4-ChIP-Seq(Encode)/Homer | 1e-7 | -1.758e+01 | 0.0000 | 221.0 | 0.55% | 149.8 | 0.38% | motif file (matrix) | svg |
| 176 | A T G C A G T C G A T C C G T A A C G T A C G T A C T G A C G T A G C T G A T C | Sox2(HMG)/mES-Sox2-ChIP-Seq(GSE11431)/Homer | 1e-7 | -1.743e+01 | 0.0000 | 3538.0 | 8.85% | 3231.8 | 8.10% | motif file (matrix) | svg |
| 177 | C T A G A C T G C T A G T C A G T C A G T A C G C T A G A C T G | Maz(Zf)/HepG2-Maz-ChIP-Seq(GSE31477)/Homer | 1e-7 | -1.736e+01 | 0.0000 | 6062.0 | 15.16% | 5669.3 | 14.20% | motif file (matrix) | svg |
| 178 | A T G C G A T C C G T A A G C T C A G T A T C G G C A T A G C T G A C T A C T G | Sox17(HMG)/Endoderm-Sox17-ChIP-Seq(GSE61475)/Homer | 1e-7 | -1.714e+01 | 0.0000 | 2852.0 | 7.13% | 2578.5 | 6.46% | motif file (matrix) | svg |
| 179 | T A G C G C A T A G T C G A T C A T G C G A C T C T A G A C T G A C T G C T G A A C T G C T A G A G T C T G A C C G A T | GLIS3(Zf)/Thyroid-Glis3.GFP-ChIP-Seq(GSE103297)/Homer | 1e-7 | -1.679e+01 | 0.0000 | 6032.0 | 15.08% | 5647.3 | 14.15% | motif file (matrix) | svg |
| 180 | A C G T T C G A T C G A A G T C G T C A T A C G A T G C A C G T A C T G A G C T | Myf5(bHLH)/GM-Myf5-ChIP-Seq(GSE24852)/Homer | 1e-7 | -1.659e+01 | 0.0000 | 3093.0 | 7.73% | 2813.6 | 7.05% | motif file (matrix) | svg |
| 181 | G A C T C G A T T C A G G A T C G A C T A G C T A G C T A G T C G A T C C G T A C T A G C T A G T C G A T C G A C T G A | Bcl6(Zf)/Liver-Bcl6-ChIP-Seq(GSE31578)/Homer | 1e-7 | -1.659e+01 | 0.0000 | 5910.0 | 14.78% | 5532.0 | 13.86% | motif file (matrix) | svg |
| 182 | A T G C G A C T A G C T A G C T A G T C G C T A C A G T C G A T G C T A A C G T A C T G G C T A T A G C G C A T T G A C | IRF:BATF(IRF:bZIP)/pDC-Irf8-ChIP-Seq(GSE66899)/Homer | 1e-7 | -1.652e+01 | 0.0000 | 464.0 | 1.16% | 359.7 | 0.90% | motif file (matrix) | svg |
| 183 | G A C T A G C T G T A C G A C T C T G A A C T G G T C A C T G A A T G C T A C G G A C T A C G T A G T C G A C T C T G A | HRE(HSF)/Striatum-HSF1-ChIP-Seq(GSE38000)/Homer | 1e-7 | -1.650e+01 | 0.0000 | 897.0 | 2.24% | 748.3 | 1.87% | motif file (matrix) | svg |
| 184 | T A G C G C T A A C T G C G T A A C G T C G T A C G T A T A C G T C A G T C G A | Gata1(Zf)/K562-GATA1-ChIP-Seq(GSE18829)/Homer | 1e-7 | -1.625e+01 | 0.0000 | 2162.0 | 5.41% | 1930.3 | 4.84% | motif file (matrix) | svg |
| 185 | T C G A G A C T A G C T T G A C A G C T G T A C T C A G G A T C A T C G T G C A A C T G C T G A | GFX(?)/Promoter/Homer | 1e-7 | -1.625e+01 | 0.0000 | 72.0 | 0.18% | 37.0 | 0.09% | motif file (matrix) | svg |
| 186 | T C A G A C G T A G T C T C G A A G T C T C A G G C A T C T A G C T A G A G C T | Usf2(bHLH)/C2C12-Usf2-ChIP-Seq(GSE36030)/Homer | 1e-7 | -1.615e+01 | 0.0000 | 1363.0 | 3.41% | 1180.5 | 2.96% | motif file (matrix) | svg |
| 187 | T G C A A C T G T A C G A T G C A G T C G A C T T C G A A T C G | ZNF711(Zf)/SHSY5Y-ZNF711-ChIP-Seq(GSE20673)/Homer | 1e-6 | -1.600e+01 | 0.0000 | 7684.0 | 19.21% | 7268.3 | 18.21% | motif file (matrix) | svg |
| 188 | C G T A C G T A C G T A A G T C A G C T C T G A A C T G C T A G A G C T A G T C C G T A T C A G | RORg(NR)/Liver-Rorc-ChIP-Seq(GSE101115)/Homer | 1e-6 | -1.580e+01 | 0.0000 | 357.0 | 0.89% | 268.2 | 0.67% | motif file (matrix) | svg |
| 189 | C T A G C A T G T G C A A C G T A G T C C G T A C A T G T C A G A C G T A C G T G C T A A G T C | Six1(Homeobox)/Myoblast-Six1-ChIP-Chip(GSE20150)/Homer | 1e-6 | -1.518e+01 | 0.0000 | 1016.0 | 2.54% | 864.8 | 2.17% | motif file (matrix) | svg |
| 190 | A G C T A G T C A T G C A G C T A C G T C G T A A C G T A G T C C G A T A T G C | Gata2(Zf)/K562-GATA2-ChIP-Seq(GSE18829)/Homer | 1e-6 | -1.503e+01 | 0.0000 | 2396.0 | 5.99% | 2162.4 | 5.42% | motif file (matrix) | svg |
| 191 | C T G A A C T G A C T G A G T C A G T C A G C T C T A G T A C G | ZFX(Zf)/mES-Zfx-ChIP-Seq(GSE11431)/Homer | 1e-6 | -1.489e+01 | 0.0000 | 5436.0 | 13.59% | 5092.3 | 12.76% | motif file (matrix) | svg |
| 192 | C A T G G A C T G C A T A C T G A G C T A C T G A C T G C G T A G C A T A G C T A T C G T A C G | Foxh1(Forkhead)/hESC-FOXH1-ChIP-Seq(GSE29422)/Homer | 1e-6 | -1.484e+01 | 0.0000 | 2307.0 | 5.77% | 2079.1 | 5.21% | motif file (matrix) | svg |
| 193 | T C G A T C G A A G T C C G T A C T A G T A G C A C G T A C T G | MyoG(bHLH)/C2C12-MyoG-ChIP-Seq(GSE36024)/Homer | 1e-6 | -1.481e+01 | 0.0000 | 4670.0 | 11.68% | 4350.6 | 10.90% | motif file (matrix) | svg |
| 194 | A T G C A G T C G C T A C G A T C G T A G C A T G C T A C G A T C T A G C A T G T G A C G T C A | CArG(MADS)/PUER-Srf-ChIP-Seq(Sullivan\_et\_al.)/Homer | 1e-6 | -1.480e+01 | 0.0000 | 1124.0 | 2.81% | 966.2 | 2.42% | motif file (matrix) | svg |
| 195 | T G C A C T G A A T G C G T C A A C G T A T G C A C G T A C T G A C T G T G C A | ZBTB18(Zf)/HEK293-ZBTB18.GFP-ChIP-Seq(GSE58341)/Homer | 1e-6 | -1.467e+01 | 0.0000 | 2255.0 | 5.64% | 2031.0 | 5.09% | motif file (matrix) | svg |
| 196 | T C G A C T A G A G T C A G T C C G T A C G T A A C G T T A G C T C A G T A C G | NFY(CCAAT)/Promoter/Homer | 1e-6 | -1.440e+01 | 0.0000 | 3474.0 | 8.69% | 3200.1 | 8.02% | motif file (matrix) | svg |
| 197 | T C G A T A G C G T C A A C T G C T A G C G T A C G A T A C T G A C G T A C T G A C T G A C G T | ETS:RUNX(ETS,Runt)/Jurkat-RUNX1-ChIP-Seq(GSE17954)/Homer | 1e-6 | -1.430e+01 | 0.0000 | 435.0 | 1.09% | 341.3 | 0.86% | motif file (matrix) | svg |
| 198 | T C G A A G T C C G T A A T C G A T G C C G A T A C T G A G T C A G C T A C T G | Tcf12(bHLH)/GM12878-Tcf12-ChIP-Seq(GSE32465)/Homer | 1e-6 | -1.397e+01 | 0.0000 | 4144.0 | 10.36% | 3851.5 | 9.65% | motif file (matrix) | svg |
| 199 | A T G C A G C T T C A G T G A C T C A G A T G C T G C A A C G T A T C G G A T C A C T G A G T C | NRF1(NRF)/MCF7-NRF1-ChIP-Seq(Unpublished)/Homer | 1e-6 | -1.396e+01 | 0.0000 | 683.0 | 1.71% | 565.7 | 1.42% | motif file (matrix) | svg |
| 200 | A T G C A G T C A G C T A G C T A C G T A T C G C G T A C G A T T A G C G A C T | LEF1(HMG)/H1-LEF1-ChIP-Seq(GSE64758)/Homer | 1e-5 | -1.377e+01 | 0.0000 | 2880.0 | 7.20% | 2637.0 | 6.61% | motif file (matrix) | svg |
| 201 | C T G A C A T G A C T G A C G T A T G C C G T A C A T G T A C G A T G C G C T A T A C G C T G A C T A G A C T G A C G T A T G C C G T A T A G C | RAR:RXR(NR),DR5/ES-RAR-ChIP-Seq(GSE56893)/Homer | 1e-5 | -1.363e+01 | 0.0000 | 135.0 | 0.34% | 87.7 | 0.22% | motif file (matrix) | svg |
| 202 | G T A C C T A G T C A G A G C T T A G C C G T A A T G C T A C G A G T C G T A C G T C A A G T C | Srebp2(bHLH)/HepG2-Srebp2-ChIP-Seq(GSE31477)/Homer | 1e-5 | -1.323e+01 | 0.0000 | 548.0 | 1.37% | 447.0 | 1.12% | motif file (matrix) | svg |
| 203 | C G T A C T G A C G T A C T A G T C G A C T A G A C T G C G T A C G T A T A C G A G C T A T C G | SpiB(ETS)/OCILY3-SPIB-ChIP-Seq(GSE56857)/Homer | 1e-5 | -1.233e+01 | 0.0000 | 965.0 | 2.41% | 833.7 | 2.09% | motif file (matrix) | svg |
| 204 | T A C G G A C T T G A C C G T A A C G T G A T C G T C A C G T A A C G T A T G C C G T A G A C T | HOXA2(Homeobox)/mES-Hoxa2-ChIP-Seq(Donaldson\_et\_al.)/Homer | 1e-5 | -1.222e+01 | 0.0000 | 413.0 | 1.03% | 329.8 | 0.83% | motif file (matrix) | svg |
| 205 | A T G C G A C T A C T G C A G T G A T C A C G T T A C G T A C G | Smad2(MAD)/ES-SMAD2-ChIP-Seq(GSE29422)/Homer | 1e-5 | -1.222e+01 | 0.0000 | 7233.0 | 18.09% | 6884.3 | 17.25% | motif file (matrix) | svg |
| 206 | T C A G A T C G G A C T A C T G G A C T C A G T C T A G C G T A G T A C C G T A C T A G A T C G | Tbx20(T-box)/Heart-Tbx20-ChIP-Seq(GSE29636)/Homer | 1e-5 | -1.213e+01 | 0.0000 | 1011.0 | 2.53% | 877.2 | 2.20% | motif file (matrix) | svg |
| 207 | C G T A C T A G C A T G A G C T A C T G C G A T A T C G C G T A G T C A G T C A | Tbet(T-box)/CD8-Tbet-ChIP-Seq(GSE33802)/Homer | 1e-5 | -1.210e+01 | 0.0000 | 4271.0 | 10.68% | 3997.6 | 10.01% | motif file (matrix) | svg |
| 208 | A G C T A T G C G A C T G C A T C G T A A G C T G T A C C G A T A T C G A G T C | Gata6(Zf)/HUG1N-GATA6-ChIP-Seq(GSE51936)/Homer | 1e-5 | -1.163e+01 | 0.0000 | 3243.0 | 8.11% | 3008.1 | 7.54% | motif file (matrix) | svg |
| 209 | T G A C A G T C C G T A A C T G G T A C A C G T A C T G A C G T G A C T G A T C | Twist2(bHLH)/Myoblast-Twist2.Ty1-ChIP-Seq(GSE127998)/Homer | 1e-4 | -1.139e+01 | 0.0000 | 7907.0 | 19.77% | 7559.9 | 18.94% | motif file (matrix) | svg |
| 210 | A C G T T A C G G A T C A C T G A C G T C T A G A C T G A C T G G A T C C T A G C A T G C T A G | Egr2(Zf)/Thymocytes-Egr2-ChIP-Seq(GSE34254)/Homer | 1e-4 | -1.135e+01 | 0.0000 | 837.0 | 2.09% | 720.3 | 1.80% | motif file (matrix) | svg |
| 211 | T A G C G T A C A G T C G T A C C G A T A G T C A G T C A G T C A G T C A G T C C G T A G A T C | Zfp281(Zf)/ES-Zfp281-ChIP-Seq(GSE81042)/Homer | 1e-4 | -1.109e+01 | 0.0000 | 1148.0 | 2.87% | 1012.4 | 2.54% | motif file (matrix) | svg |
| 212 | C T G A T C A G A G T C C G T A A T C G A T G C C G A T A C T G A G T C G A C T A T C G A G T C | MyoD(bHLH)/Myotube-MyoD-ChIP-Seq(GSE21614)/Homer | 1e-4 | -1.101e+01 | 0.0000 | 3371.0 | 8.43% | 3139.4 | 7.86% | motif file (matrix) | svg |
| 213 | C T A G A C G T A G T C C G T A A C T G A G T C G C A T A C T G G C A T A G T C G A C T G A T C G C A T A G T C A G C T | ZNF317(Zf)/HEK293-ZNF317.GFP-ChIP-Seq(GSE58341)/Homer | 1e-4 | -1.090e+01 | 0.0000 | 376.0 | 0.94% | 301.9 | 0.76% | motif file (matrix) | svg |
| 214 | C G A T C T A G C T G A A T G C C T G A T C G A C G T A C T G A T C G A T A G C A G T C C G T A A C T G T C G A A T G C | Hand2(bHLH)/Mesoderm-Hand2-ChIP-Seq(GSE61475)/Homer | 1e-4 | -1.068e+01 | 0.0000 | 2038.0 | 5.10% | 1860.1 | 4.66% | motif file (matrix) | svg |
| 215 | C G A T C T A G C G T A G A C T C A G T C T A G C G T A A G C T C A T G C T A G | HOXA1(Homeobox)/mES-Hoxa1-ChIP-Seq(SRP084292)/Homer | 1e-4 | -1.060e+01 | 0.0001 | 978.0 | 2.45% | 856.7 | 2.15% | motif file (matrix) | svg |
| 216 | G T A C G A T C C A G T A G T C A G T C A G T C T G C A G A T C C T G A A T G C G T C A A C G T | WT1(Zf)/Kidney-WT1-ChIP-Seq(GSE90016)/Homer | 1e-4 | -1.053e+01 | 0.0001 | 2620.0 | 6.55% | 2420.1 | 6.06% | motif file (matrix) | svg |
| 217 | T A C G C T G A C A T G G A T C G T A C G C A T T C A G T A C G A G C T G T C A G A T C G C A T T A C G C G T A C T A G G A T C G A T C C G A T A C T G T C A G | ZNF322(Zf)/HEK293-ZNF322.GFP-ChIP-Seq(GSE58341)/Homer | 1e-4 | -1.028e+01 | 0.0001 | 1268.0 | 3.17% | 1131.3 | 2.83% | motif file (matrix) | svg |
| 218 | C G T A A T G C C G A T A C G T A G T C C G T A C G T A C G T A C T A G A T C G | TCFL2(HMG)/K562-TCF7L2-ChIP-Seq(GSE29196)/Homer | 1e-4 | -1.028e+01 | 0.0001 | 400.0 | 1.00% | 325.8 | 0.82% | motif file (matrix) | svg |
| 219 | A C T G A C G T C A T G A T C G A T C G T G A C A C T G A T C G A T C G T G C A C T G A C G T A | E2F3(E2F)/MEF-E2F3-ChIP-Seq(GSE71376)/Homer | 1e-4 | -1.012e+01 | 0.0001 | 2194.0 | 5.49% | 2015.7 | 5.05% | motif file (matrix) | svg |
| 220 | T C G A T C A G T C G A A C T G C A T G A C G T A G T C C T G A | COUP-TFII(NR)/Artia-Nr2f2-ChIP-Seq(GSE46497)/Homer | 1e-4 | -9.811e+00 | 0.0001 | 7367.0 | 18.42% | 7057.6 | 17.68% | motif file (matrix) | svg |
| 221 | A C T G G A T C C T G A A T C G A G T C T A G C C T G A C G T A T A C G A G T C C T A G C A G T T C A G T C G A T G A C G A T C | PAX5(Paired,Homeobox)/GM12878-PAX5-ChIP-Seq(GSE32465)/Homer | 1e-4 | -9.714e+00 | 0.0001 | 1555.0 | 3.89% | 1408.8 | 3.53% | motif file (matrix) | svg |
| 222 | T G A C C T G A C T A G C T G A C G T A A G T C C T G A A C G T G C A T T A G C G C A T A T C G G A C T G A C T G A T C | GRE(NR),IR3/RAW264.7-GRE-ChIP-Seq(Unpublished)/Homer | 1e-4 | -9.684e+00 | 0.0001 | 917.0 | 2.29% | 805.9 | 2.02% | motif file (matrix) | svg |
| 223 | A C T G G A C T A G T C C T G A G A T C T C A G A T G C G A C T A G T C A T G C T A G C A G C T A T C G T G C A | PAX5(Paired,Homeobox),condensed/GM12878-PAX5-ChIP-Seq(GSE32465)/Homer | 1e-4 | -9.512e+00 | 0.0001 | 491.0 | 1.23% | 411.3 | 1.03% | motif file (matrix) | svg |
| 224 | C T G A C T G A C T A G T C G A C G T A A T G C C G T A A C T G C G T A A C G T C T G A C G A T A G C T C G T A A C G T A G T C C G A T T A C G G T C A G C A T | GATA(Zf),IR3/iTreg-Gata3-ChIP-Seq(GSE20898)/Homer | 1e-4 | -9.505e+00 | 0.0001 | 527.0 | 1.32% | 444.2 | 1.11% | motif file (matrix) | svg |
| 225 | C G T A A C G T A C G T A C G T A C G T A G T C A G T C C T G A A G C T A G C T | NFAT(RHD)/Jurkat-NFATC1-ChIP-Seq(Jolma\_et\_al.)/Homer | 1e-4 | -9.452e+00 | 0.0002 | 3300.0 | 8.25% | 3090.7 | 7.74% | motif file (matrix) | svg |
| 226 | C G A T C T G A C G T A C A G T A G T C G A T C G A T C A C T G | Pitx1(Homeobox)/Chicken-Pitx1-ChIP-Seq(GSE38910)/Homer | 1e-4 | -9.397e+00 | 0.0002 | 17128.0 | 42.83% | 16725.5 | 41.90% | motif file (matrix) | svg |
| 227 | A C G T A G T C A G T C C G A T A C G T A C G T A C T G A C G T A T G C G A C T A C T G T A C G | Sox21(HMG)/ESC-SOX21-ChIP-Seq(GSE110505)/Homer | 1e-4 | -9.388e+00 | 0.0002 | 7362.0 | 18.41% | 7060.2 | 17.69% | motif file (matrix) | svg |
| 228 | A T G C T A G C A G C T A G C T T G A C G A C T T C A G T A C G G T C A C T G A A T C G T A G C G A C T C A G T A G T C A G C T T C G A A T C G T G C A T G C A | HRE(HSF)/HepG2-HSF1-ChIP-Seq(GSE31477)/Homer | 1e-4 | -9.385e+00 | 0.0002 | 637.0 | 1.59% | 546.8 | 1.37% | motif file (matrix) | svg |
| 229 | G C T A A T C G G C T A G A C T G C T A T C G A T A G C T C G A | GATA3(Zf)/iTreg-Gata3-ChIP-Seq(GSE20898)/Homer | 1e-4 | -9.325e+00 | 0.0002 | 5378.0 | 13.45% | 5117.0 | 12.82% | motif file (matrix) | svg |
| 230 | A G T C G A T C G C T A C G A T A C G T T A C G G C A T C T G A G A C T A C T G A G T C G C T A C T G A T C G A C A G T | Oct4:Sox17(POU,Homeobox,HMG)/F9-Sox17-ChIP-Seq(GSE44553)/Homer | 1e-4 | -9.324e+00 | 0.0002 | 433.0 | 1.08% | 359.0 | 0.90% | motif file (matrix) | svg |
| 231 | C T G A A T G C G C T A C G A T A T G C C G T A C G T A C G T A C T A G T A C G | Tcf3(HMG)/mES-Tcf3-ChIP-Seq(GSE11724)/Homer | 1e-3 | -9.130e+00 | 0.0002 | 1140.0 | 2.85% | 1019.7 | 2.55% | motif file (matrix) | svg |
| 232 | T G A C T A G C T C A G T C G A T C G A C G T A A G T C C G T A C G T A C G A T C T A G T A C G | Sox7(HMG)/ESC-Sox7-ChIP-Seq(GSE133899)/Homer | 1e-3 | -9.099e+00 | 0.0002 | 1163.0 | 2.91% | 1041.0 | 2.61% | motif file (matrix) | svg |
| 233 | T C G A C T G A C T G A A T G C G T A C G T C A A G C T A G C T C G T A T C A G | Barx1(Homeobox)/Stomach-Barx1.3xFlag-ChIP-Seq(GSE69483)/Homer | 1e-3 | -8.753e+00 | 0.0003 | 1664.0 | 4.16% | 1521.4 | 3.81% | motif file (matrix) | svg |
| 234 | A G C T C T G A C T A G C T A G A C T G T A G C T G C A T C G A C T G A C T A G C A T G A C G T A T G C T C G A | RXR(NR),DR1/3T3L1-RXR-ChIP-Seq(GSE13511)/Homer | 1e-3 | -8.436e+00 | 0.0004 | 4571.0 | 11.43% | 4342.7 | 10.88% | motif file (matrix) | svg |
| 235 | T A C G T C A G T G C A A G C T T G A C A G C T A G T C A C T G G A T C A C T G T C G A A C T G C T G A C T G A A T G C | ZBTB33(Zf)/GM12878-ZBTB33-ChIP-Seq(GSE32465)/Homer | 1e-3 | -8.365e+00 | 0.0004 | 162.0 | 0.41% | 121.4 | 0.30% | motif file (matrix) | svg |
| 236 | T A C G A T C G T A G C G A T C A C T G A C G T A G T C A C G T C T A G A T C G | Smad4(MAD)/ESC-SMAD4-ChIP-Seq(GSE29422)/Homer | 1e-3 | -8.201e+00 | 0.0005 | 7384.0 | 18.46% | 7105.3 | 17.80% | motif file (matrix) | svg |
| 237 | C A T G T A C G T A G C G A T C G A T C A T G C G T A C G A C T T C A G A T G C C G A T A T C G C A G T A C T G G T A C | Zic3(Zf)/mES-Zic3-ChIP-Seq(GSE37889)/Homer | 1e-3 | -7.824e+00 | 0.0007 | 2449.0 | 6.12% | 2287.2 | 5.73% | motif file (matrix) | svg |
| 238 | A T C G T G A C A T G C C T G A T C A G G A C T A G T C C G A T T C A G T C G A C A T G C T A G C T A G C G T A C T A G C T A G C T G A C T A G C T A G A T G C | ZSCAN22(Zf)/HEK293-ZSCAN22.GFP-ChIP-Seq(GSE58341)/Homer | 1e-3 | -7.804e+00 | 0.0008 | 291.0 | 0.73% | 238.0 | 0.60% | motif file (matrix) | svg |
| 239 | A T G C A T G C A T C G T A C G A G C T A G T C G C T A A G T C T C A G G A C T A C T G T C G A | E-box(bHLH)/Promoter/Homer | 1e-3 | -7.650e+00 | 0.0009 | 308.0 | 0.77% | 253.3 | 0.63% | motif file (matrix) | svg |
| 240 | G A C T C T A G C T A G A G T C T G C A A C T G A C G T A C G T C T A G T C A G | AMYB(HTH)/Testes-AMYB-ChIP-Seq(GSE44588)/Homer | 1e-3 | -7.471e+00 | 0.0010 | 6650.0 | 16.63% | 6398.7 | 16.03% | motif file (matrix) | svg |
| 241 | T C A G T G A C G T A C C G T A A C G T T G A C A C G T T C A G A G C T G A C T | NeuroD1(bHLH)/Islet-NeuroD1-ChIP-Seq(GSE30298)/Homer | 1e-3 | -7.304e+00 | 0.0012 | 3470.0 | 8.68% | 3286.9 | 8.23% | motif file (matrix) | svg |
| 242 | G C A T C T A G G T A C A G T C C G A T A C T G C T A G C T A G G T A C G C T A | ZNF416(Zf)/HEK293-ZNF416.GFP-ChIP-Seq(GSE58341)/Homer | 1e-3 | -7.199e+00 | 0.0014 | 6095.0 | 15.24% | 5858.8 | 14.68% | motif file (matrix) | svg |
| 243 | T C A G A G C T A T G C C G T A A G T C T C A G A C G T A T C G T C G A A G T C G A T C T G A C | TFE3(bHLH)/MEF-TFE3-ChIP-Seq(GSE75757)/Homer | 1e-3 | -6.938e+00 | 0.0018 | 381.0 | 0.95% | 323.1 | 0.81% | motif file (matrix) | svg |
| 244 | A T G C G A T C G A C T A G C T C G A T C G A T G T C A C G A T T C G A A T C G T A G C T A G C | TATA-Box(TBP)/Promoter/Homer | 1e-3 | -6.933e+00 | 0.0018 | 4817.0 | 12.04% | 4609.4 | 11.55% | motif file (matrix) | svg |
| 245 | C T A G A T G C A T G C C G A T A C T G G A C T A T G C G C T A T G A C A G C T T A G C G C T A | PBX1(Homeobox)/MCF7-PBX1-ChIP-Seq(GSE28007)/Homer | 1e-2 | -6.787e+00 | 0.0020 | 391.0 | 0.98% | 333.9 | 0.84% | motif file (matrix) | svg |
| 246 | A C T G A G C T A G T C G T C A A G C T T C A G A T G C G A T C G C A T A T C G T C G A T A G C C G A T C A T G T A G C | Pax8(Paired,Homeobox)/Thyroid-Pax8-ChIP-Seq(GSE26938)/Homer | 1e-2 | -6.614e+00 | 0.0024 | 1394.0 | 3.49% | 1284.1 | 3.22% | motif file (matrix) | svg |
| 247 | C G T A C G A T G A C T G A C T T G A C C T G A A T G C C T G A T A G C A G T C A C G T T C G A C A T G T A C G G A C T A T C G G A C T A C G T C T G A T C G A C G T A | Brachyury(T-box)/Mesoendoderm-Brachyury-ChIP-exo(GSE54963)/Homer | 1e-2 | -6.544e+00 | 0.0026 | 1081.0 | 2.70% | 985.5 | 2.47% | motif file (matrix) | svg |
| 248 | G C T A C G T A A G T C A C G T T C G A T A C G A C T G A G C T A G T C T C G A | RORgt(NR)/EL4-RORgt.Flag-ChIP-Seq(GSE56019)/Homer | 1e-2 | -6.478e+00 | 0.0027 | 499.0 | 1.25% | 435.9 | 1.09% | motif file (matrix) | svg |
| 249 | G C T A C G T A A G T C A C G T T C G A T A C G A C T G A G C T A G T C T C G A | RORgt(NR)/EL4-RORgt.Flag-ChIP-Seq(GSE56019)/Homer | 1e-2 | -6.478e+00 | 0.0027 | 499.0 | 1.25% | 435.9 | 1.09% | motif file (matrix) | svg |
| 250 | A C G T C T A G C G T A A G T C G T A C A C G T A C G T A C G T G T C A G T A C T G A C G A C T | Nur77(NR)/K562-NR4A1-ChIP-Seq(GSE31363)/Homer | 1e-2 | -6.406e+00 | 0.0029 | 669.0 | 1.67% | 595.9 | 1.49% | motif file (matrix) | svg |
| 251 | A G T C G T A C A G C T C T A G A G T C C G A T A C T G C G T A A C T G G T C A | Zic(Zf)/Cerebellum-ZIC1.2-ChIP-Seq(GSE60731)/Homer | 1e-2 | -6.329e+00 | 0.0031 | 3684.0 | 9.21% | 3511.2 | 8.80% | motif file (matrix) | svg |
| 252 | T C G A A C T G A C T G C G T A C G T A T C G A A G T C C T G A A T C G G T A C G C A T C A T G | ETS:E-box(ETS,bHLH)/HPC7-Scl-ChIP-Seq(GSE22178)/Homer | 1e-2 | -6.169e+00 | 0.0037 | 356.0 | 0.89% | 304.5 | 0.76% | motif file (matrix) | svg |
| 253 | C T A G C T G A A G T C G C T A C G A T A C T G G A C T G A T C G A T C C T G A C T A G C T G A T G A C G C T A C G A T T C A G G A C T G A T C G A T C T G A C | p53(p53)/Saos-p53-ChIP-Seq(GSE15780)/Homer | 1e-2 | -5.936e+00 | 0.0046 | 401.0 | 1.00% | 347.8 | 0.87% | motif file (matrix) | svg |
| 254 | C T A G C T G A A G T C G C T A C G A T A C T G G A C T G A T C G A T C C T G A C T A G C T G A T G A C G C T A C G A T T C A G G A C T G A T C G A T C T G A C | p53(p53)/Saos-p53-ChIP-Seq/Homer | 1e-2 | -5.936e+00 | 0.0046 | 401.0 | 1.00% | 347.8 | 0.87% | motif file (matrix) | svg |
| 255 | C T A G C A T G C A T G T A C G A G T C G C A T A G C T C T A G A C G T A G T C G A C T A C T G A C T G A C T G T C G A | Zfp809(Zf)/ES-Zfp809-ChIP-Seq(GSE70799)/Homer | 1e-2 | -5.834e+00 | 0.0051 | 792.0 | 1.98% | 716.6 | 1.80% | motif file (matrix) | svg |
| 256 | T G A C C T A G T C A G G T C A C G T A T C A G C G A T T C A G T C G A T G C A C T G A T A G C | PU.1-IRF(ETS:IRF)/Bcell-PU.1-ChIP-Seq(GSE21512)/Homer | 1e-2 | -5.811e+00 | 0.0051 | 5468.0 | 13.67% | 5271.2 | 13.20% | motif file (matrix) | svg |
| 257 | A G T C G C A T C G T A C G T A G T A C A C G T A C T G G A T C G A T C T C G A | BMYB(HTH)/Hela-BMYB-ChIP-Seq(GSE27030)/Homer | 1e-2 | -5.702e+00 | 0.0057 | 6338.0 | 15.85% | 6130.5 | 15.36% | motif file (matrix) | svg |
| 258 | C G T A C G T A C G T A A G T C A G C T C T G A A C T G A C T G A G C T A G T C C G T A C T A G C T A G C T A G T G C A | RORa(NR)/Liver-Rora-ChIP-Seq(GSE101115)/Homer | 1e-2 | -5.681e+00 | 0.0058 | 502.0 | 1.26% | 443.3 | 1.11% | motif file (matrix) | svg |
| 259 | C T A G T C G A C T G A C G T A T A C G G A C T T C A G T C G A G T C A T G C A T A C G A G C T | IRF2(IRF)/Erythroblas-IRF2-ChIP-Seq(GSE36985)/Homer | 1e-2 | -5.559e+00 | 0.0065 | 390.0 | 0.98% | 339.3 | 0.85% | motif file (matrix) | svg |
| 260 | C G A T T A C G T G C A G T A C G A T C G A C T A G C T A C G T A T C G G T A C G A T C G T A C G A T C G T C A | PPARE(NR),DR1/3T3L1-Pparg-ChIP-Seq(GSE13511)/Homer | 1e-2 | -5.519e+00 | 0.0068 | 3771.0 | 9.43% | 3611.5 | 9.05% | motif file (matrix) | svg |
| 261 | C T A G G A T C T C G A A T G C T C G A A G C T C T G A A C T G A C G T C T G A A C T G A C T G A C G T A G T C G A T C | ZKSCAN1(Zf)/HepG2-ZKSCAN1-ChIP-Seq(Encode)/Homer | 1e-2 | -5.252e+00 | 0.0088 | 109.0 | 0.27% | 84.1 | 0.21% | motif file (matrix) | svg |
| 262 | T C A G C T G A C G T A C G T A T A C G G C A T C T A G C T G A C G T A C G T A T A C G G A C T | IRF1(IRF)/PBMC-IRF1-ChIP-Seq(GSE43036)/Homer | 1e-2 | -5.183e+00 | 0.0094 | 493.0 | 1.23% | 438.0 | 1.10% | motif file (matrix) | svg |
| 263 | T A C G T A C G G T A C A T C G A C T G T A C G T C G A C T G A T C G A A T C G | E2F6(E2F)/Hela-E2F6-ChIP-Seq(GSE31477)/Homer | 1e-2 | -5.110e+00 | 0.0101 | 1848.0 | 4.62% | 1741.8 | 4.36% | motif file (matrix) | svg |
| 264 | T A G C C G A T A C G T A G C T A G C T A G T C A T G C A G T C A C T G A T G C A T G C G C T A | E2F7(E2F)/Hela-E2F7-ChIP-Seq(GSE32673)/Homer | 1e-2 | -5.085e+00 | 0.0103 | 389.0 | 0.97% | 341.5 | 0.86% | motif file (matrix) | svg |
| 265 | G A C T C T A G A T G C A G T C G T C A T A C G A T G C A T C G | HIC1(Zf)/Treg-ZBTB29-ChIP-Seq(GSE99889)/Homer | 1e-2 | -5.075e+00 | 0.0104 | 8914.0 | 22.29% | 8691.1 | 21.77% | motif file (matrix) | svg |
| 266 | T G C A C G T A G T C A A G C T A G T C G C T A T A G C C G A T C T A G G A T C | Gfi1b(Zf)/HPC7-Gfi1b-ChIP-Seq(GSE22178)/Homer | 1e-2 | -5.035e+00 | 0.0108 | 2620.0 | 6.55% | 2494.1 | 6.25% | motif file (matrix) | svg |
| 267 | T A C G C T G A C T A G T C G A C G T A A G T C C T G A A T C G G C A T T A G C G A C T A C T G A C G T A G C T A G T C G A C T | GRE(NR),IR3/A549-GR-ChIP-Seq(GSE32465)/Homer | 1e-2 | -5.021e+00 | 0.0109 | 496.0 | 1.24% | 442.8 | 1.11% | motif file (matrix) | svg |
| 268 | C T A G T A C G G A C T T G C A T G C A C G A T T A C G C T G A T C G A C T G A | Hoxa10(Homeobox)/ChickenMSG-Hoxa10.Flag-ChIP-Seq(GSE86088)/Homer | 1e-2 | -4.941e+00 | 0.0117 | 2008.0 | 5.02% | 1899.3 | 4.76% | motif file (matrix) | svg |
| 269 | C A T G A G T C C T G A T G A C A T C G G A C T G T C A A G T C T A G C G A T C | HIF2a(bHLH)/785\_O-HIF2a-ChIP-Seq(GSE34871)/Homer | 1e-2 | -4.903e+00 | 0.0121 | 1268.0 | 3.17% | 1182.8 | 2.96% | motif file (matrix) | svg |
| 270 | C A G T G A C T C G T A G C T A G T A C G A T C G T A C G A C T A G C T A C G T T G A C C G T A C A G T A C G T A T G C | ZNF652/HepG2-ZNF652.Flag-ChIP-Seq(Encode)/Homer | 1e-2 | -4.848e+00 | 0.0128 | 836.0 | 2.09% | 767.9 | 1.92% | motif file (matrix) | svg |
| 271 | G A T C G C A T A C T G C T A G C T G A A G C T G C T A G C T A C T G A T C A G G C A T T G C A A C G T A C G T G A T C G A C T G C A T C T A G T A C G G A C T C T A G C A T G C T A G G T A C T C G A | ZNF136(Zf)/HEK293-ZNF136.GFP-ChIP-Seq(GSE58341)/Homer | 1e-2 | -4.829e+00 | 0.0130 | 266.0 | 0.67% | 228.7 | 0.57% | motif file (matrix) | svg |
