# Supplementary material for: Reconstitution of pluripotency from mouse fibroblast through Sall4 overexpression
Source: Nat Commun. 2024 Dec 30;15:10787. doi: 10.1038/s41467-024-54924-5 (PMC11686038; doi:10.1038/s41467-024-54924-5)
Supplement: Supplementary file 4 — Source Data [file 41467_2024_54924_MOESM4_ESM.zip › source data/main figures/figure3/d/all the motif for the SALL4 system.pdf]

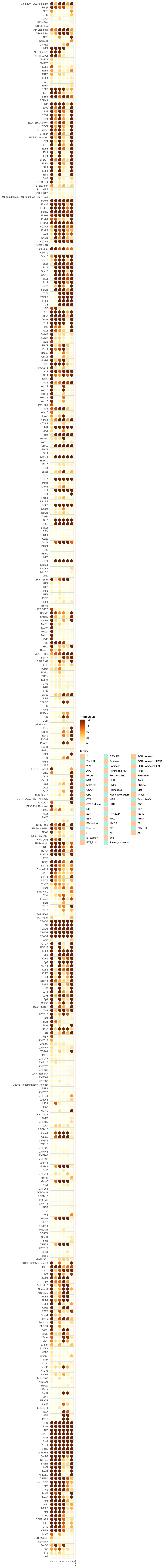
−logpvalue
100
75
50
25
0
family
?
?,bHLH
?,Zf
AP2
bHLH
bZIP
bZIP,IRF
CCAAT
CP2
CTF
CTF,Forkhead
DM
E2F
EBF
EBV−virus
Encode
ETS
ETS,bHLH
ETS,Runt
ETS:IRF
forkhead
Forkhead
Forkhead,bHLH
Forkhead,NR
HLH
HMG
Homeobox
Homeobox,bHLH
HSF
HTH
IRF
IRF:bZIP
MAD
MADS
NR
NRF
p53
Paired,Homeobox
POU,Homeobox
POU,Homeobox,HMG
POU,Homeobox,IR1
RHD
RHD,bZIP
Runt
SNAPc
Stat
T−box
T−box,MAD
TBP
TEA
TEAD
THAP
Zf
Zf,bHLH
Zf?
