# Supplementary material for: Reconstitution of pluripotency from mouse fibroblast through Sall4 overexpression
Source: Nat Commun. 2024 Dec 30;15:10787. doi: 10.1038/s41467-024-54924-5 (PMC11686038; doi:10.1038/s41467-024-54924-5)
Supplement: Supplementary file 4 — Source Data [file 41467_2024_54924_MOESM4_ESM.zip › source data/Supplementary figures/Supplementary figure10/c,d,e/UC15_res/UC15_GO_res.pdf]

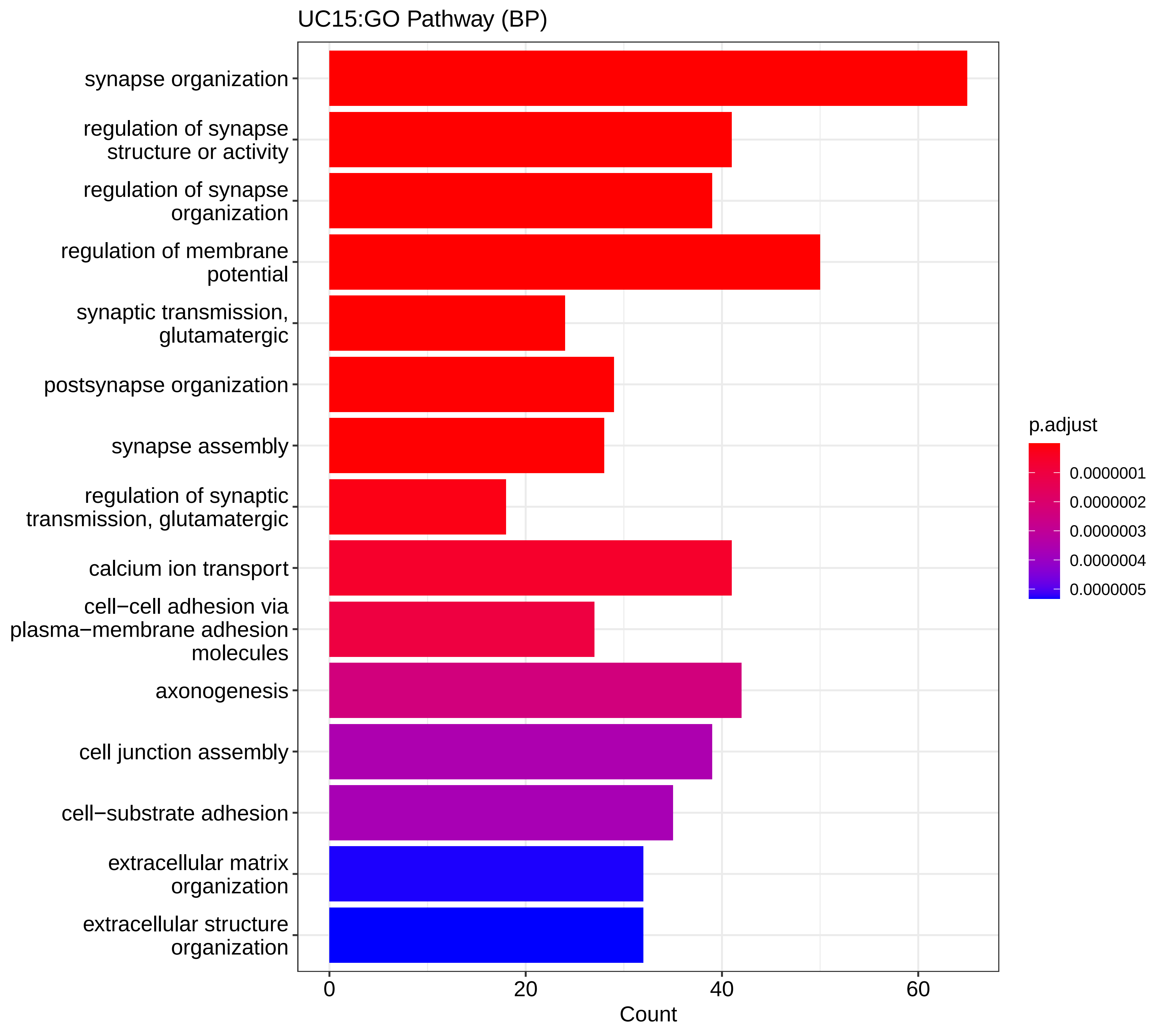
UC15:GO Pathway (BP)
synapse organization
regulation of synapse structure or activity
regulation of synapse organization
regulation of membrane potential
synaptic transmission, glutamatergic
postsynapse organization
synapse assembly
regulation of synaptic transmission, glutamatergic
calcium ion transport
cell−cell adhesion via plasma−membrane adhesion molecules
axonogenesis
cell junction assembly
cell−substrate adhesion
extracellular matrix organization
extracellular structure organization
0
20
40
60
Count
p.adjust
0.0000001
0.0000002
0.0000003
0.0000004
0.0000005
