# Supplementary material for: Reconstitution of pluripotency from mouse fibroblast through Sall4 overexpression
Source: Nat Commun. 2024 Dec 30;15:10787. doi: 10.1038/s41467-024-54924-5 (PMC11686038; doi:10.1038/s41467-024-54924-5)
Supplement: Supplementary file 4 — Source Data [file 41467_2024_54924_MOESM4_ESM.zip › source data/Supplementary figures/Supplementary figure14/b,c/ct_D0_OCT4 in oct4 system_res/TSS_GObp_res.pdf]

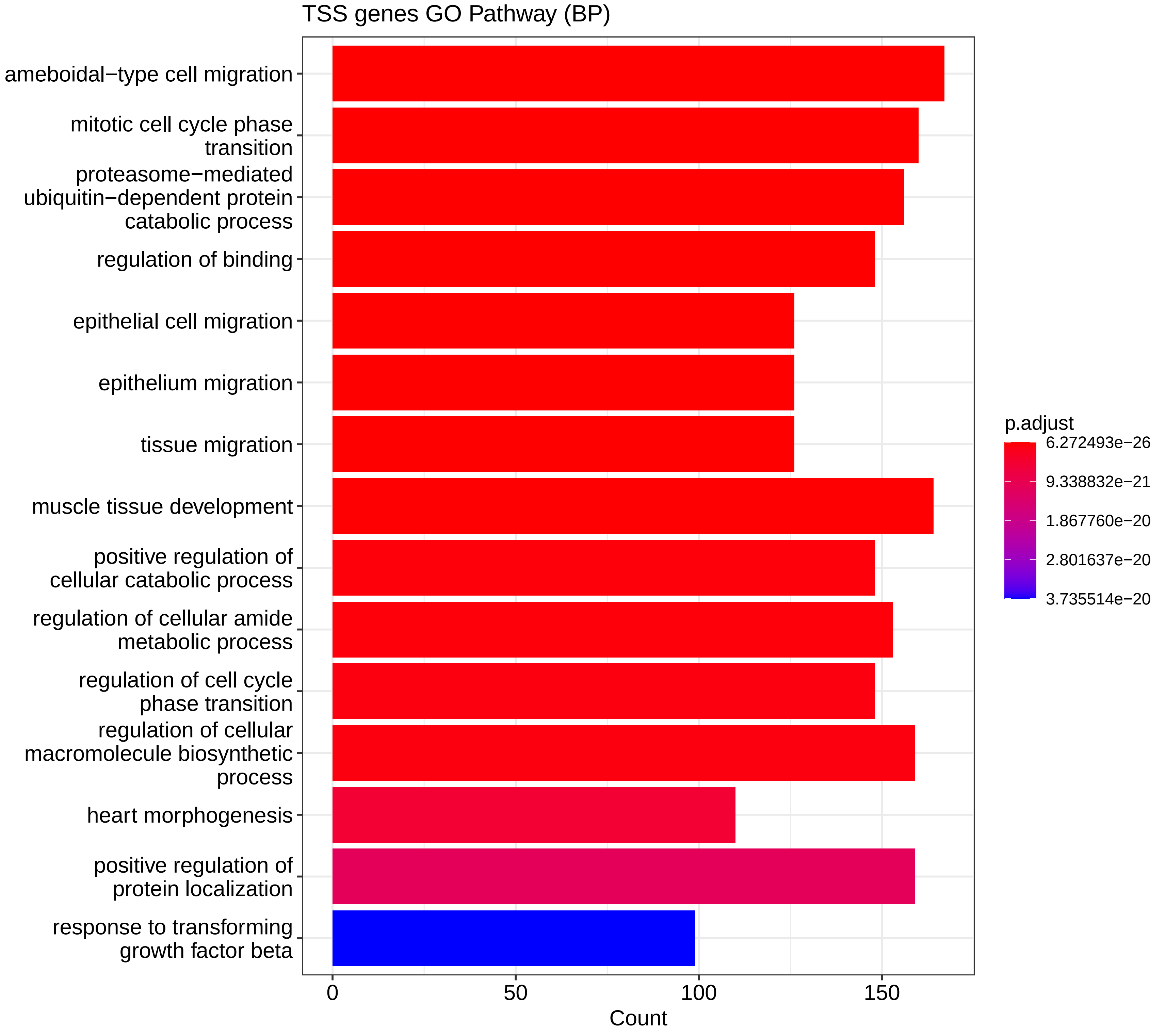
TSS genes GO Pathway (BP)
ameboidal−type cell migration
mitotic cell cycle phase transition
proteasome−mediated ubiquitin−dependent protein catabolic process
regulation of binding
epithelial cell migration
epithelium migration
tissue migration
muscle tissue development
positive regulation of cellular catabolic process
regulation of cellular amide metabolic process
regulation of cell cycle phase transition
regulation of cellular macromolecule biosynthetic process
heart morphogenesis
positive regulation of protein localization
response to transforming growth factor beta
0
50
100
150
Count
p.adjust
6.272493e−26
9.338832e−21
1.867760e−20
2.801637e−20
3.735514e−20
