# Supplementary material for: Reconstitution of pluripotency from mouse fibroblast through Sall4 overexpression
Source: Nat Commun. 2024 Dec 30;15:10787. doi: 10.1038/s41467-024-54924-5 (PMC11686038; doi:10.1038/s41467-024-54924-5)
Supplement: Supplementary file 4 — Source Data [file 41467_2024_54924_MOESM4_ESM.zip › source data/Supplementary figures/Supplementary figure14/b,c/ct_D0_OCT4 in OS system_res/all_peaks_annopie.pdf]

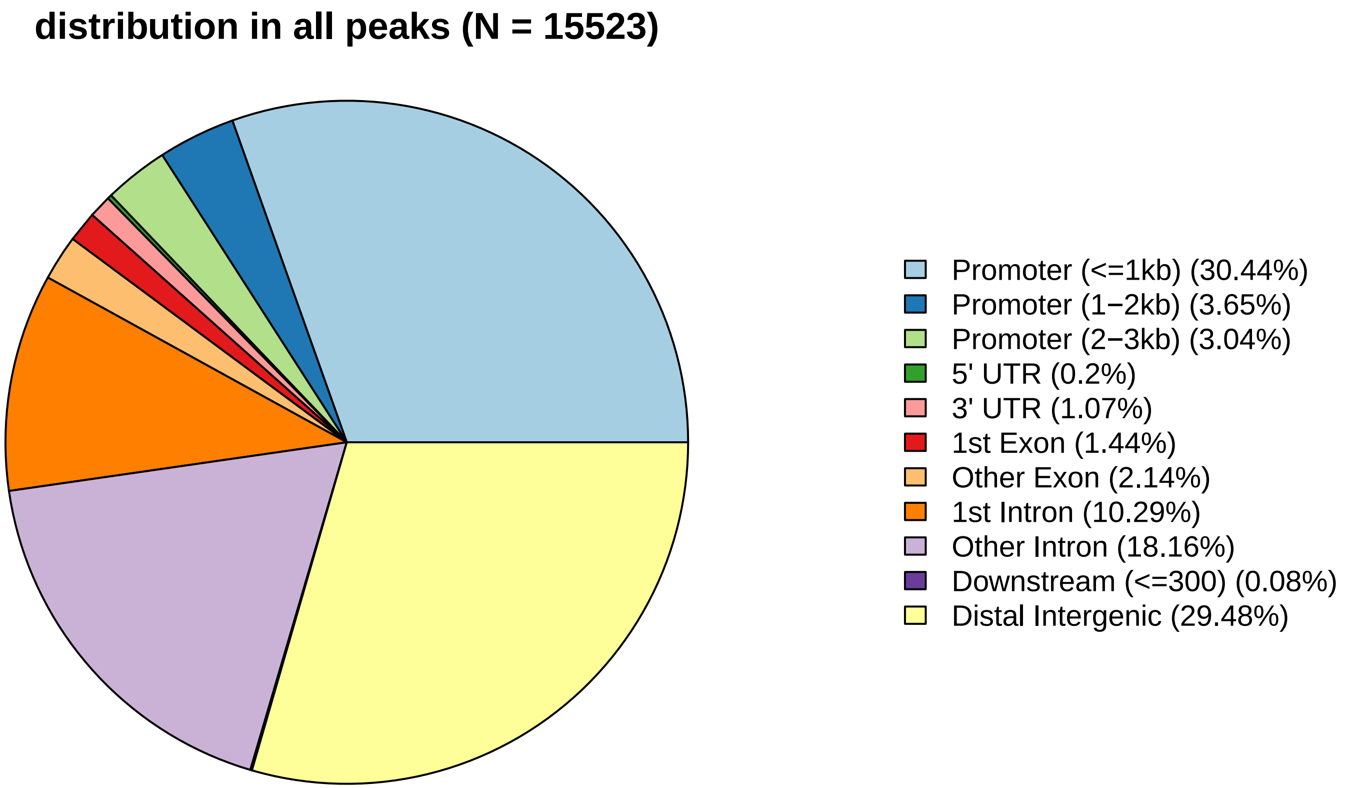
distribution in all peaks (N = 15523)
Promoter (<=1kb) (30.44%)
Promoter (1−2kb) (3.65%)
Promoter (2−3kb) (3.04%)
5' UTR (0.2%)
3' UTR (1.07%)
1st Exon (1.44%)
Other Exon (2.14%)
1st Intron (10.29%)
Other Intron (18.16%)
Downstream (<=300) (0.08%)
Distal Intergenic (29.48%)
