# Supplementary material for: Reconstitution of pluripotency from mouse fibroblast through Sall4 overexpression
Source: Nat Commun. 2024 Dec 30;15:10787. doi: 10.1038/s41467-024-54924-5 (PMC11686038; doi:10.1038/s41467-024-54924-5)
Supplement: Supplementary file 4 — Source Data [file 41467_2024_54924_MOESM4_ESM.zip › source data/Supplementary figures/Supplementary figure14/b,c/ct_D0_OCT4 in OS system_res/TSS_GObp_res.pdf]

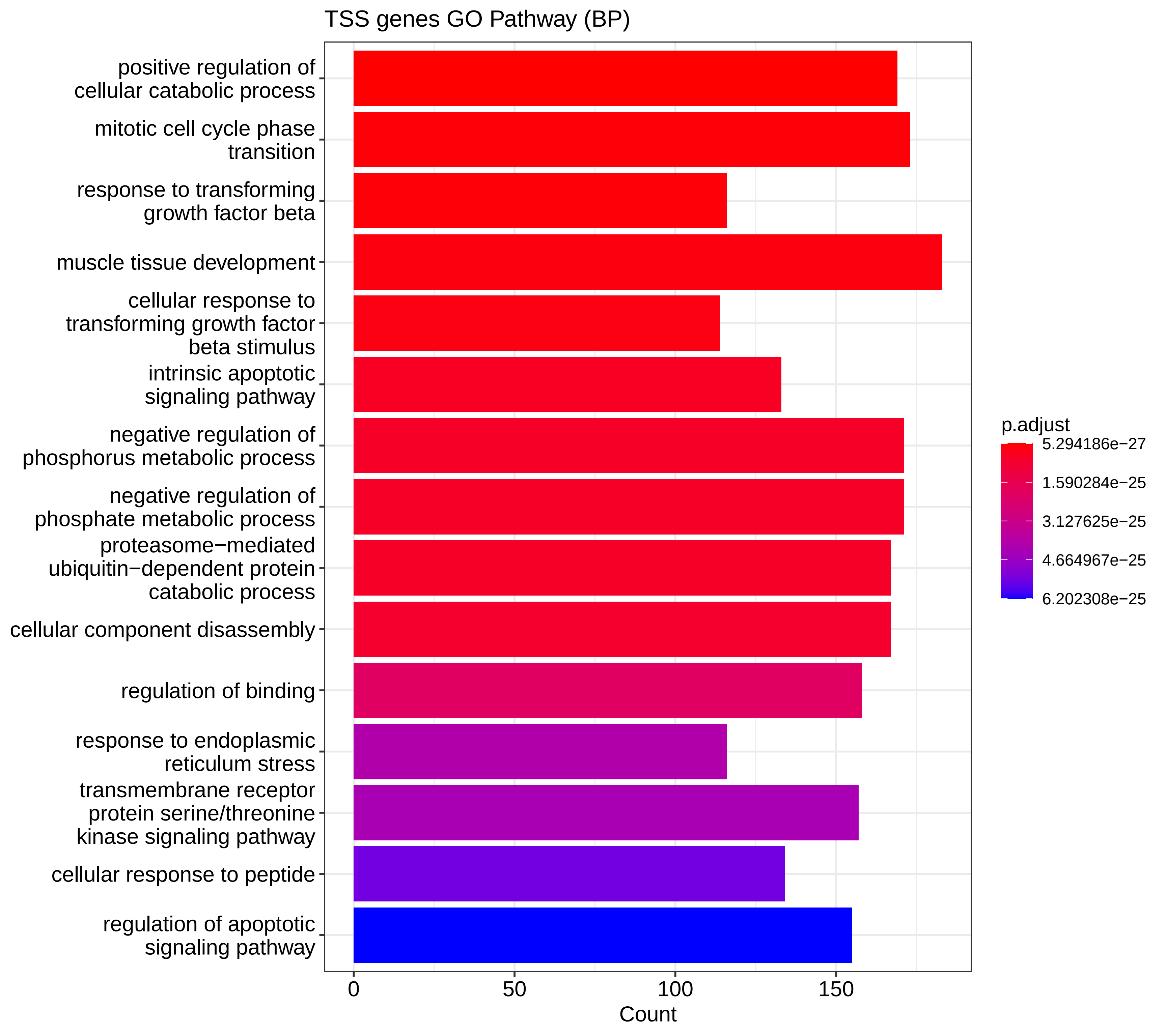
TSS genes GO Pathway (BP)
positive regulation of cellular catabolic process
mitotic cell cycle phase transition
response to transforming growth factor beta
muscle tissue development
cellular response to transforming growth factor beta stimulus
intrinsic apoptotic signaling pathway
negative regulation of phosphorus metabolic process
negative regulation of phosphate metabolic process
proteasome−mediated ubiquitin−dependent protein catabolic process
cellular component disassembly
regulation of binding
response to endoplasmic reticulum stress
transmembrane receptor protein serine/threonine kinase signaling pathway
cellular response to peptide
regulation of apoptotic signaling pathway
0
50
100
150
Count
p.adjust
5.294186e−27
1.590284e−25
3.127625e−25
4.664967e−25
6.202308e−25
