# Supplementary material for: Reconstitution of pluripotency from mouse fibroblast through Sall4 overexpression
Source: Nat Commun. 2024 Dec 30;15:10787. doi: 10.1038/s41467-024-54924-5 (PMC11686038; doi:10.1038/s41467-024-54924-5)
Supplement: Supplementary file 4 — Source Data [file 41467_2024_54924_MOESM4_ESM.zip › source data/Supplementary figures/Supplementary figure14/b,c/ct_D0_SALL4 in OS system_res/TSS_GObp_res.pdf]

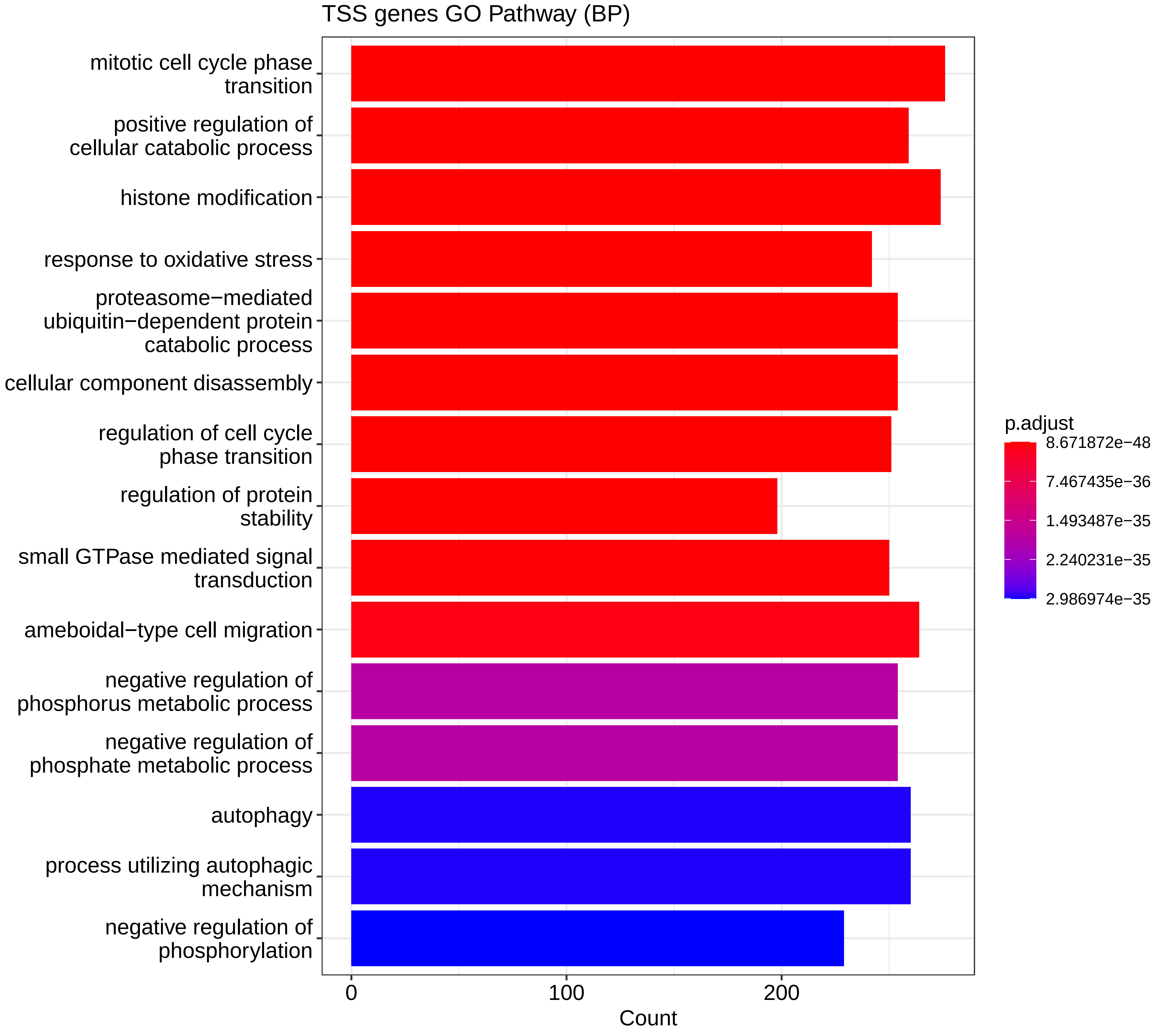

TSS genes GO Pathway (BP)
mitotic cell cycle phase transition
positive regulation of cellular catabolic process
histone modification
response to oxidative stress
proteasome−mediated ubiquitin−dependent protein catabolic process
cellular component disassembly
regulation of cell cycle phase transition
regulation of protein stability
small GTPase mediated signal transduction
ameboidal−type cell migration
negative regulation of phosphorus metabolic process
negative regulation of phosphate metabolic process
autophagy
process utilizing autophagic mechanism
negative regulation of phosphorylation
0
100
200
Count
p.adjust
8.671872e−48
7.467435e−36
1.493487e−35
2.240231e−35
2.986974e−35
