# Supplementary material for: Reconstitution of pluripotency from mouse fibroblast through Sall4 overexpression
Source: Nat Commun. 2024 Dec 30;15:10787. doi: 10.1038/s41467-024-54924-5 (PMC11686038; doi:10.1038/s41467-024-54924-5)
Supplement: Supplementary file 4 — Source Data [file 41467_2024_54924_MOESM4_ESM.zip › source data/Supplementary figures/Supplementary figure14/e/S4_SF_changes/all_S4_D0_cuttag_sites in Sall4 system_res/all_peaks_annopie.pdf]

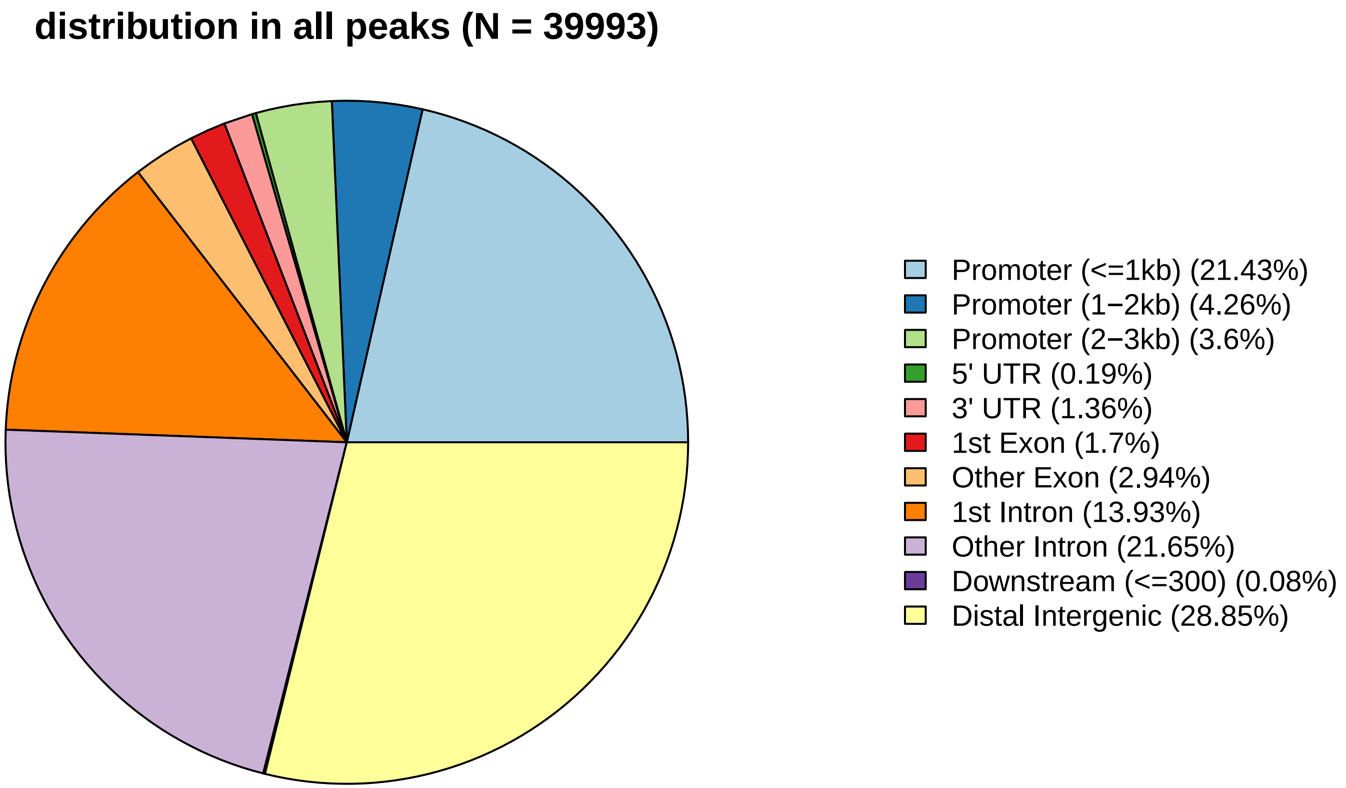

distribution in all peaks (N = 39993)
Promoter (<=1kb) (21.43%)
Promoter (1−2kb) (4.26%)
Promoter (2−3kb) (3.6%)
5' UTR (0.19%)
3' UTR (1.36%)
1st Exon (1.7%)
Other Exon (2.94%)
1st Intron (13.93%)
Other Intron (21.65%)
Downstream (<=300) (0.08%)
Distal Intergenic (28.85%)
