# Supplementary material for: Reconstitution of pluripotency from mouse fibroblast through Sall4 overexpression
Source: Nat Commun. 2024 Dec 30;15:10787. doi: 10.1038/s41467-024-54924-5 (PMC11686038; doi:10.1038/s41467-024-54924-5)
Supplement: Supplementary file 4 — Source Data [file 41467_2024_54924_MOESM4_ESM.zip › source data/Supplementary figures/Supplementary figure14/e/S4_SF_changes/all_S4_D0_cuttag_sites in Sall4 system_res/TSS_GObp_res.pdf]

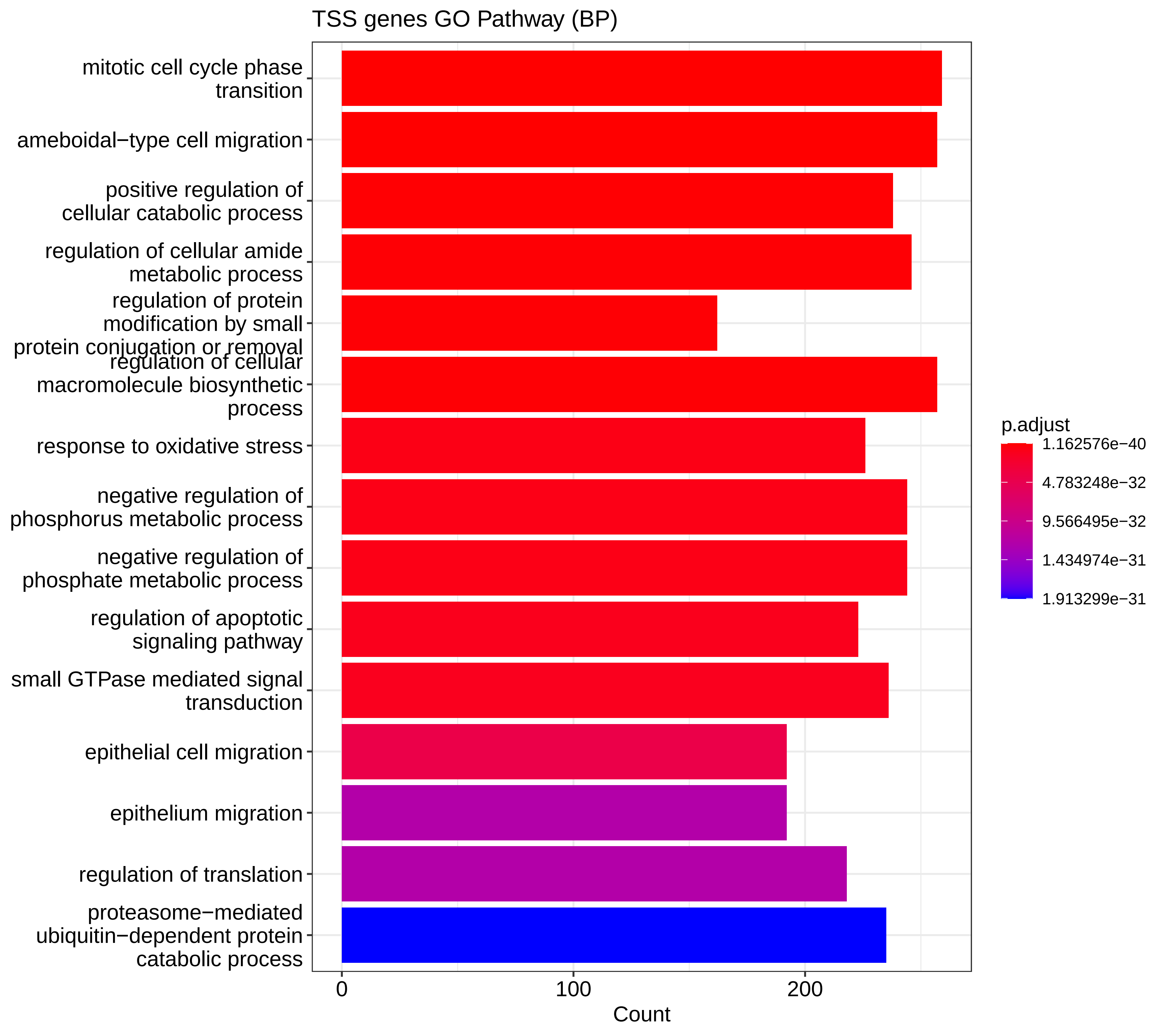
TSS genes GO Pathway (BP)
mitotic cell cycle phase transition
ameboidal−type cell migration
positive regulation of cellular catabolic process
regulation of cellular amide metabolic process
regulation of protein modification by small protein conjugation or removal
regulation of cellular macromolecule biosynthetic process
response to oxidative stress
negative regulation of phosphorus metabolic process
negative regulation of phosphate metabolic process
regulation of apoptotic signaling pathway
small GTPase mediated signal transduction
epithelial cell migration
epithelium migration
regulation of translation
proteasome−mediated ubiquitin−dependent protein catabolic process
0
100
200
Count
p.adjust
1.162576e−40
4.783248e−32
9.566495e−32
1.434974e−31
1.913299e−31
