# Supplementary material for: Reconstitution of pluripotency from mouse fibroblast through Sall4 overexpression
Source: Nat Commun. 2024 Dec 30;15:10787. doi: 10.1038/s41467-024-54924-5 (PMC11686038; doi:10.1038/s41467-024-54924-5)
Supplement: Supplementary file 4 — Source Data [file 41467_2024_54924_MOESM4_ESM.zip › source data/Supplementary figures/Supplementary figure14/e/S4_SF_changes/C1/TSS_GObp_res.pdf]

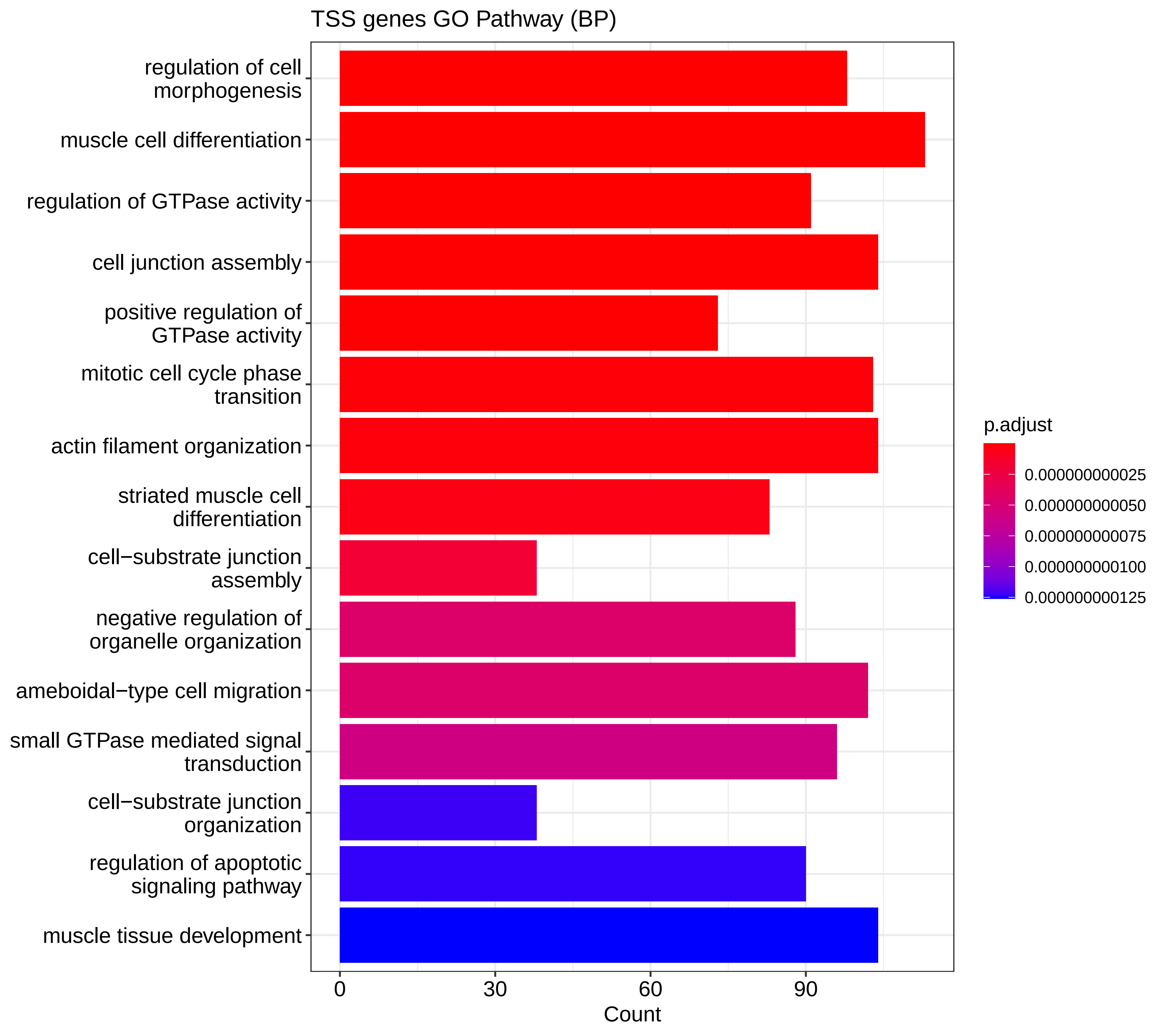
TSS genes GO Pathway (BP)
regulation of cell morphogenesis
muscle cell differentiation
regulation of GTPase activity
cell junction assembly
positive regulation of GTPase activity
mitotic cell cycle phase transition
actin filament organization
striated muscle cell differentiation
cell−substrate junction assembly
negative regulation of organelle organization
ameboidal−type cell migration
small GTPase mediated signal transduction
cell−substrate junction organization
regulation of apoptotic signaling pathway
muscle tissue development
0
30
60
90
Count
p.adjust
0.000000000025
0.000000000050
0.000000000075
0.000000000100
0.000000000125
