# Supplementary material for: Reconstitution of pluripotency from mouse fibroblast through Sall4 overexpression
Source: Nat Commun. 2024 Dec 30;15:10787. doi: 10.1038/s41467-024-54924-5 (PMC11686038; doi:10.1038/s41467-024-54924-5)
Supplement: Supplementary file 4 — Source Data [file 41467_2024_54924_MOESM4_ESM.zip › source data/Supplementary figures/Supplementary figure14/e/S4_SF_changes/C2/TSS_GObp_res.pdf]

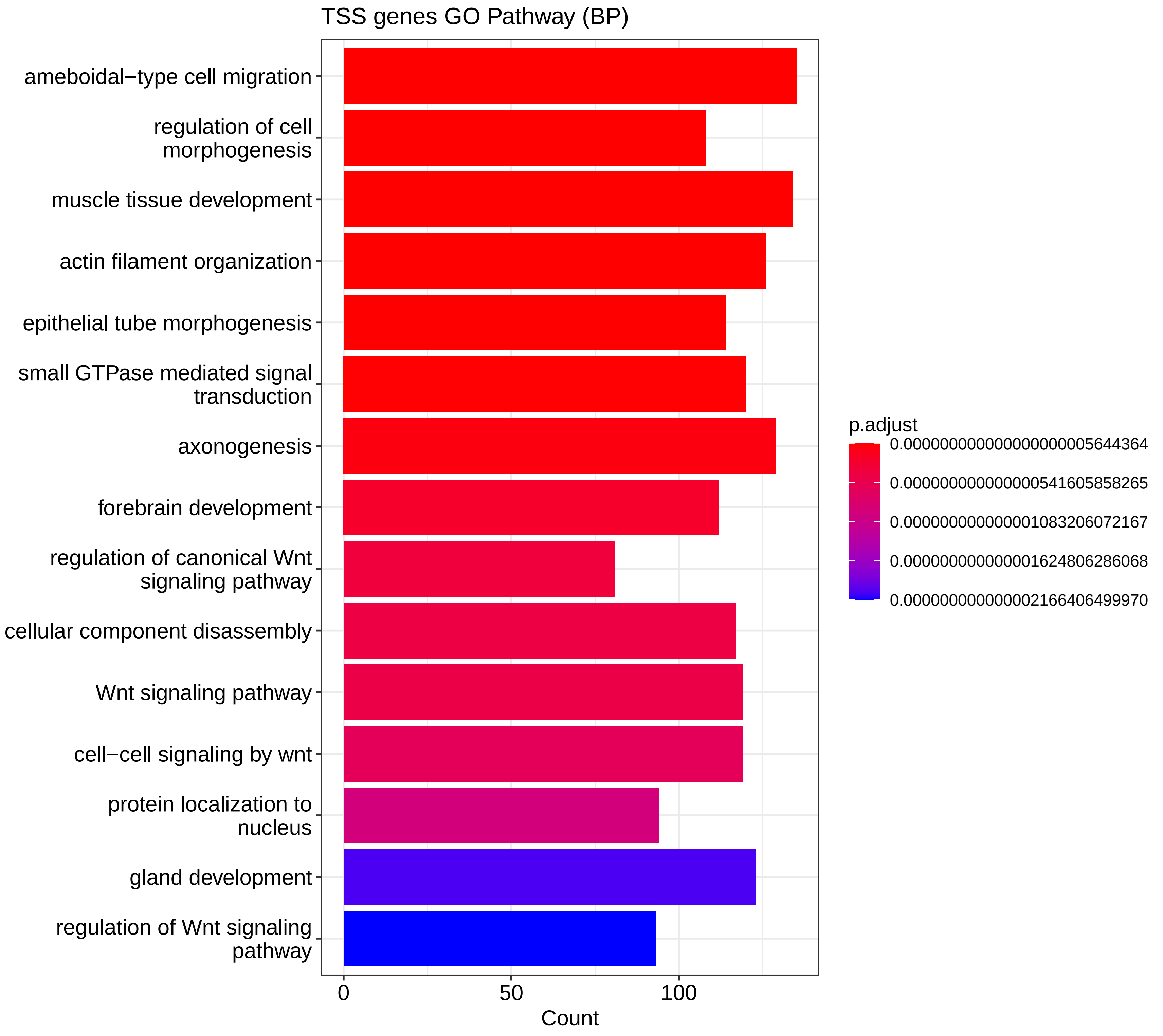
TSS genes GO Pathway (BP)
ameboidal−type cell migration
regulation of cell morphogenesis
muscle tissue development
actin filament organization
epithelial tube morphogenesis
small GTPase mediated signal transduction
axonogenesis
forebrain development
regulation of canonical Wnt signaling pathway
cellular component disassembly
Wnt signaling pathway
cell−cell signaling by wnt
protein localization to nucleus
gland development
regulation of Wnt signaling pathway
0
50
100
Count
p.adjust
0.00000000000000000005644364
0.00000000000000541605858265
0.00000000000001083206072167
0.00000000000001624806286068
0.00000000000002166406499970
