# Supplementary material for: Reconstitution of pluripotency from mouse fibroblast through Sall4 overexpression
Source: Nat Commun. 2024 Dec 30;15:10787. doi: 10.1038/s41467-024-54924-5 (PMC11686038; doi:10.1038/s41467-024-54924-5)
Supplement: Supplementary file 4 — Source Data [file 41467_2024_54924_MOESM4_ESM.zip › source data/Supplementary figures/Supplementary figure14/g,h/C3/all_peaks_annopie.pdf]

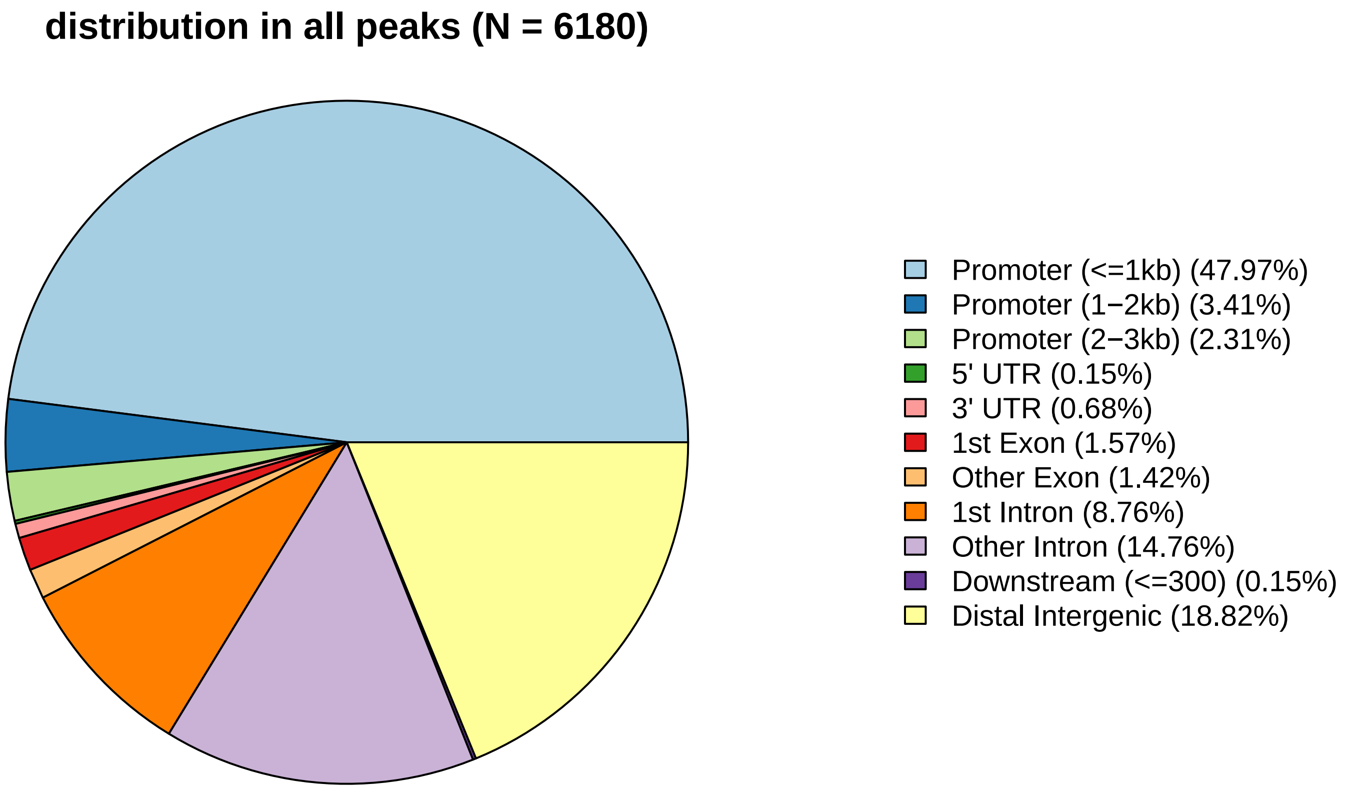
distribution in all peaks (N = 6180)
Promoter (<=1kb) (47.97%)
Promoter (1−2kb) (3.41%)
Promoter (2−3kb) (2.31%)
5' UTR (0.15%)
3' UTR (0.68%)
1st Exon (1.57%)
Other Exon (1.42%)
1st Intron (8.76%)
Other Intron (14.76%)
Downstream (<=300) (0.15%)
Distal Intergenic (18.82%)
