# Supplementary material for: Reconstitution of pluripotency from mouse fibroblast through Sall4 overexpression
Source: Nat Commun. 2024 Dec 30;15:10787. doi: 10.1038/s41467-024-54924-5 (PMC11686038; doi:10.1038/s41467-024-54924-5)
Supplement: Supplementary file 4 — Source Data [file 41467_2024_54924_MOESM4_ESM.zip › source data/Supplementary figures/Supplementary figure14/g,h/C3/TSS_GObp_res.pdf]

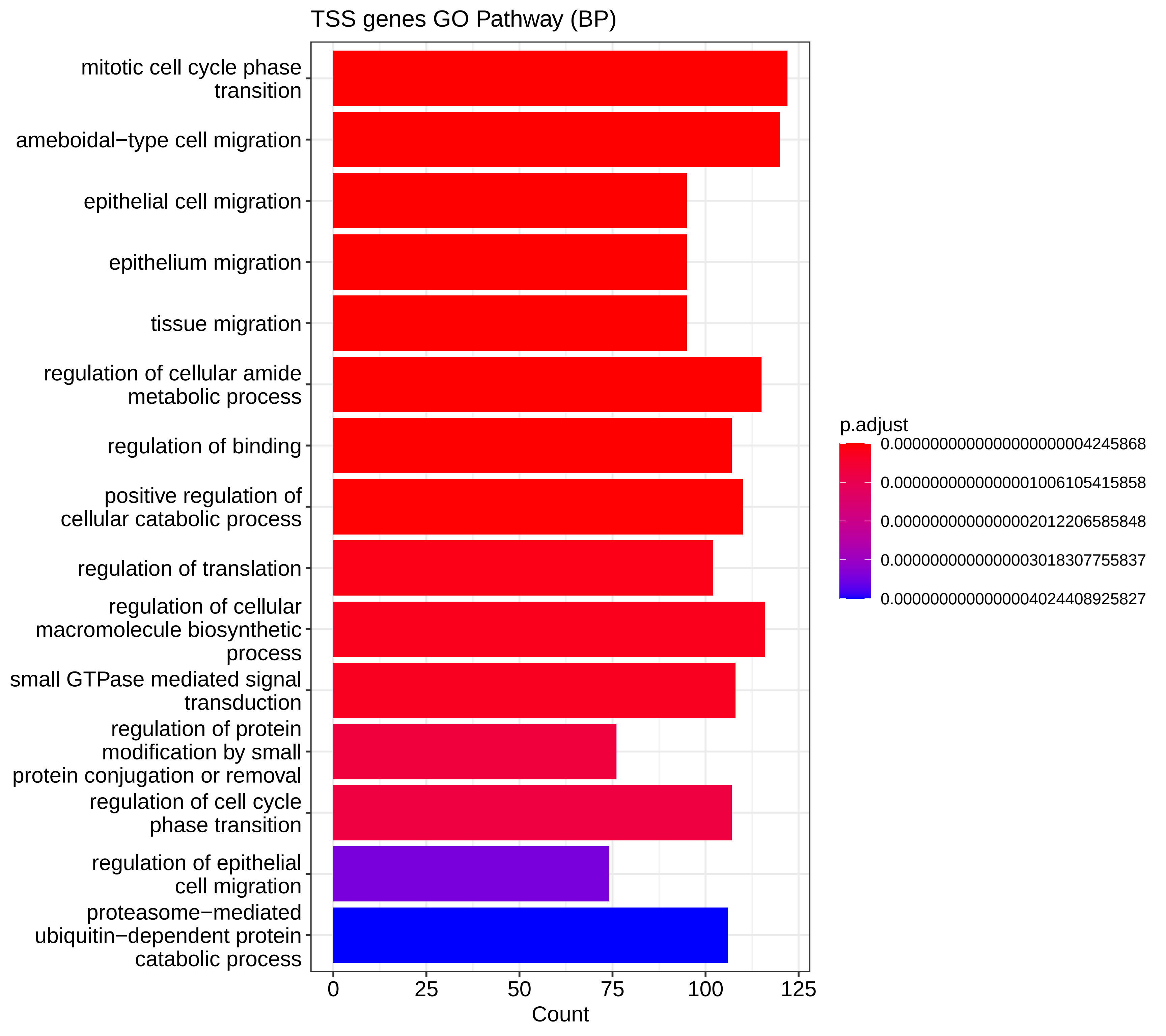
TSS genes GO Pathway (BP)
mitotic cell cycle phase transition
ameboidal−type cell migration
epithelial cell migration
epithelium migration
tissue migration
regulation of cellular amide metabolic process
regulation of binding
positive regulation of cellular catabolic process
regulation of translation
regulation of cellular macromolecule biosynthetic process
small GTPase mediated signal transduction
regulation of protein modification by small protein conjugation or removal
regulation of cell cycle phase transition
regulation of epithelial cell migration
proteasome−mediated ubiquitin−dependent protein catabolic process
0
25
50
75
100
125
Count
p.adjust
0.000000000000000000004245868
0.000000000000001006105415858
0.000000000000002012206585848
0.000000000000003018307755837
0.000000000000004024408925827
