# Supplementary material for: Reconstitution of pluripotency from mouse fibroblast through Sall4 overexpression
Source: Nat Commun. 2024 Dec 30;15:10787. doi: 10.1038/s41467-024-54924-5 (PMC11686038; doi:10.1038/s41467-024-54924-5)
Supplement: Supplementary file 4 — Source Data [file 41467_2024_54924_MOESM4_ESM.zip › source data/Supplementary figures/Supplementary figure14/g,h/C4/TSS_GObp_res.pdf]

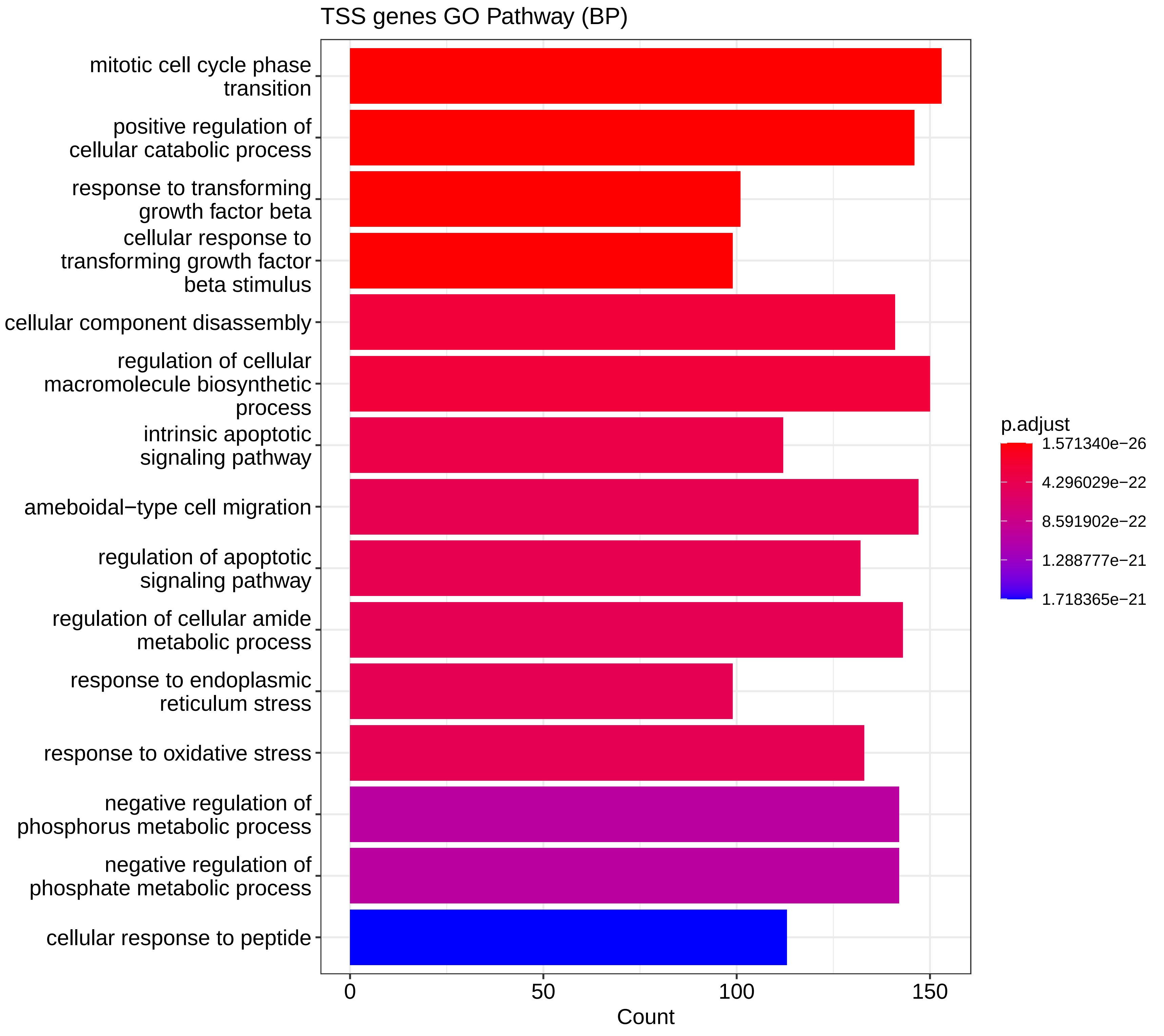

TSS genes GO Pathway (BP)
mitotic cell cycle phase transition
positive regulation of cellular catabolic process
response to transforming growth factor beta
cellular response to transforming growth factor beta stimulus
cellular component disassembly
regulation of cellular macromolecule biosynthetic process
intrinsic apoptotic signaling pathway
ameboidal−type cell migration
regulation of apoptotic signaling pathway
regulation of cellular amide metabolic process
response to endoplasmic reticulum stress
response to oxidative stress
negative regulation of phosphorus metabolic process
negative regulation of phosphate metabolic process
cellular response to peptide
0
50
100
150
Count
p.adjust
1.571340e−26
4.296029e−22
8.591902e−22
1.288777e−21
1.718365e−21
