# Supplementary material for: Reconstitution of pluripotency from mouse fibroblast through Sall4 overexpression
Source: Nat Commun. 2024 Dec 30;15:10787. doi: 10.1038/s41467-024-54924-5 (PMC11686038; doi:10.1038/s41467-024-54924-5)
Supplement: Supplementary file 4 — Source Data [file 41467_2024_54924_MOESM4_ESM.zip › source data/Supplementary figures/Supplementary figure14/g,h/C5/TSS_GObp_res.pdf]

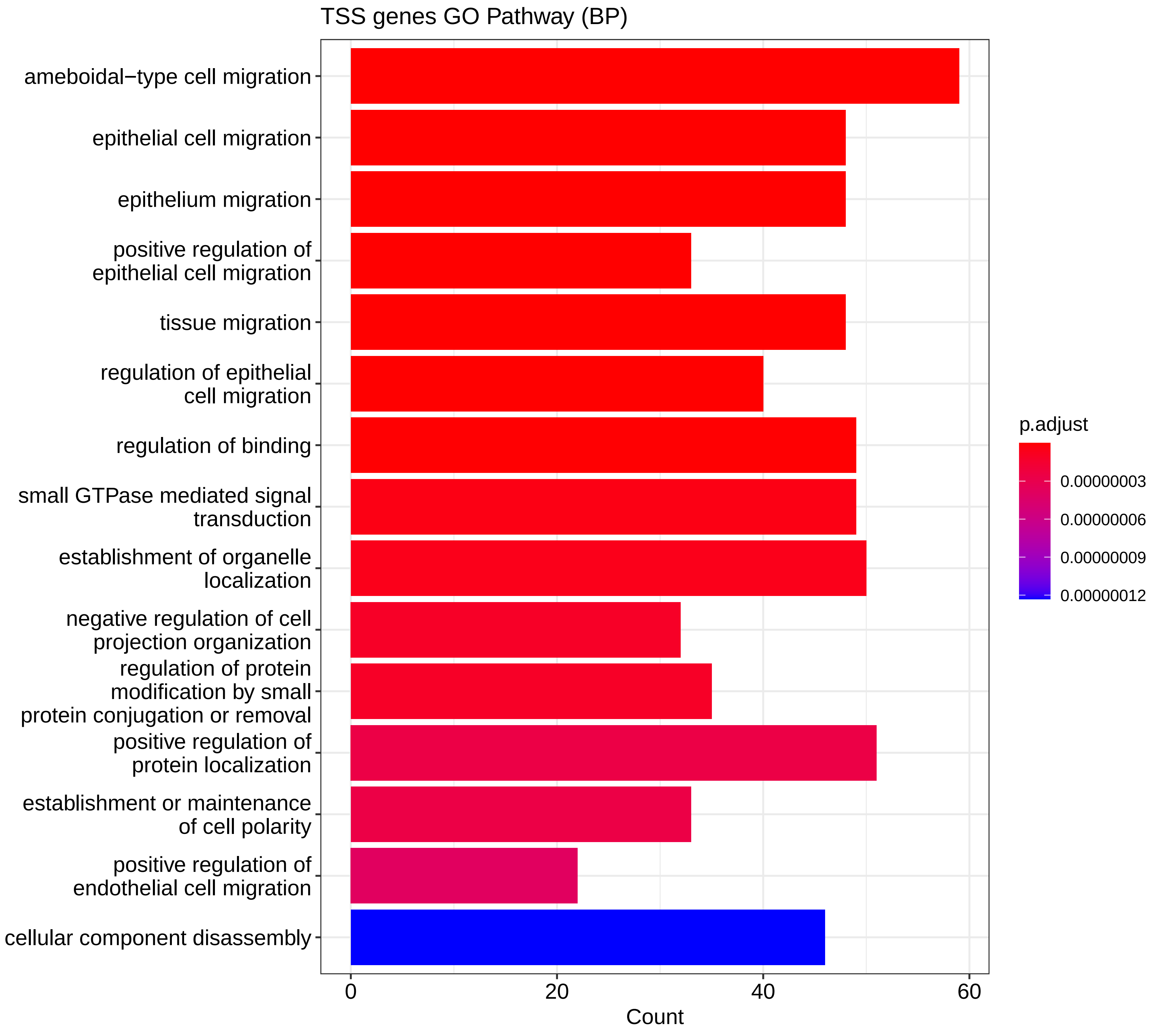
TSS genes GO Pathway (BP)
ameboidal−type cell migration
epithelial cell migration
epithelium migration
positive regulation of epithelial cell migration
tissue migration
regulation of epithelial cell migration
regulation of binding
small GTPase mediated signal transduction
establishment of organelle localization
negative regulation of cell projection organization
regulation of protein modification by small protein conjugation or removal
positive regulation of protein localization
establishment or maintenance of cell polarity
positive regulation of endothelial cell migration
cellular component disassembly
0
20
40
60
Count
p.adjust
0.00000003
0.00000006
0.00000009
0.00000012
