# Supplementary material for: Reconstitution of pluripotency from mouse fibroblast through Sall4 overexpression
Source: Nat Commun. 2024 Dec 30;15:10787. doi: 10.1038/s41467-024-54924-5 (PMC11686038; doi:10.1038/s41467-024-54924-5)
Supplement: Supplementary file 4 — Source Data [file 41467_2024_54924_MOESM4_ESM.zip › source data/Supplementary figures/Supplementary figure14/g,h/C6/TSS_GObp_res.pdf]

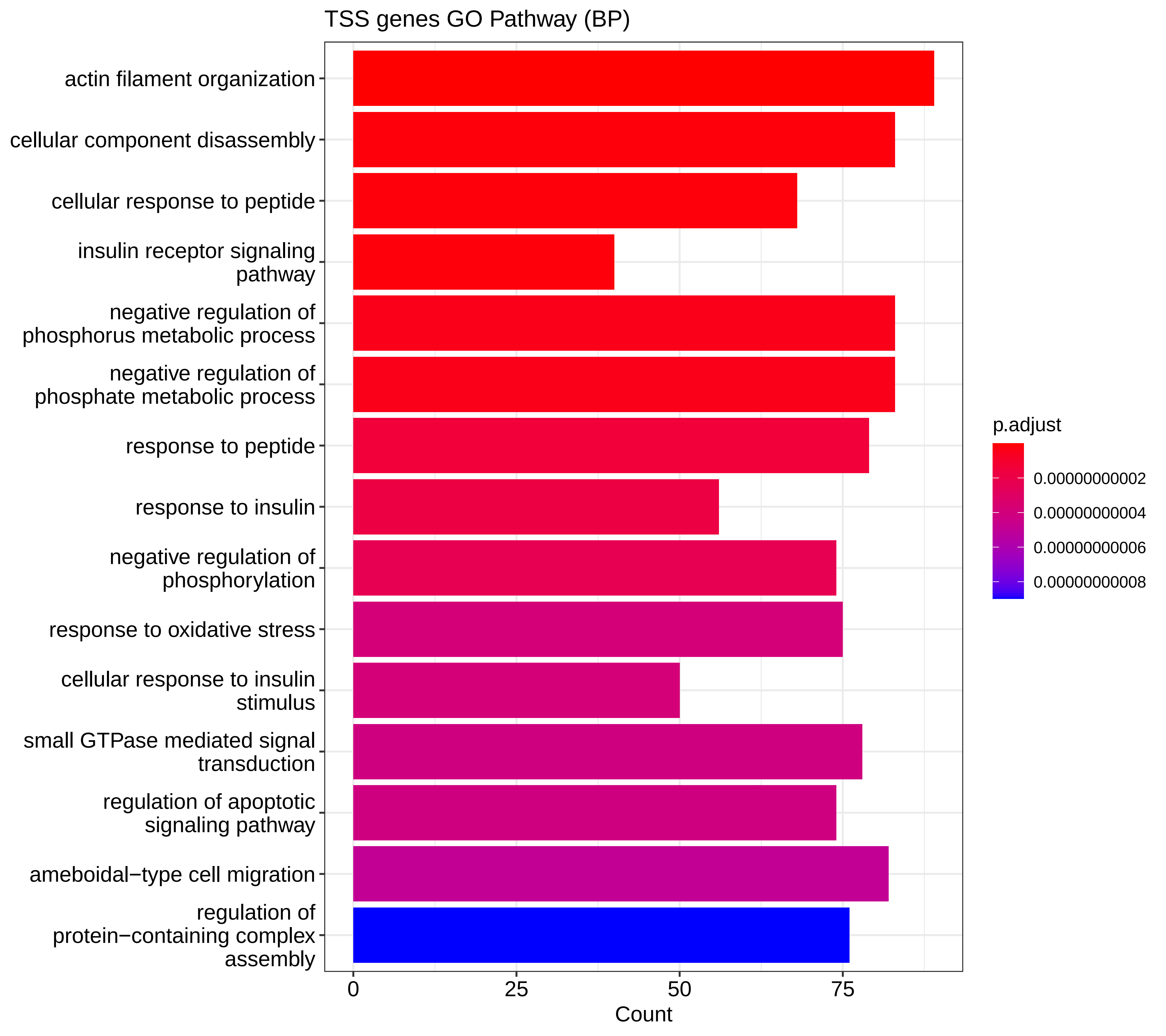
TSS genes GO Pathway (BP)
actin filament organization
cellular component disassembly
cellular response to peptide
insulin receptor signaling pathway
negative regulation of phosphorus metabolic process
negative regulation of phosphate metabolic process
response to peptide
response to insulin
negative regulation of phosphorylation
response to oxidative stress
cellular response to insulin stimulus
small GTPase mediated signal transduction
regulation of apoptotic signaling pathway
ameboidal−type cell migration
regulation of protein−containing complex assembly
0
25
50
75
Count
p.adjust
0.00000000002
0.00000000004
0.00000000006
0.00000000008
