# Supplementary material for: Reconstitution of pluripotency from mouse fibroblast through Sall4 overexpression
Source: Nat Commun. 2024 Dec 30;15:10787. doi: 10.1038/s41467-024-54924-5 (PMC11686038; doi:10.1038/s41467-024-54924-5)
Supplement: Supplementary file 4 — Source Data [file 41467_2024_54924_MOESM4_ESM.zip › source data/Supplementary figures/Supplementary figure2/d,e,f/IP-MS DATA/heatmap.pdf]

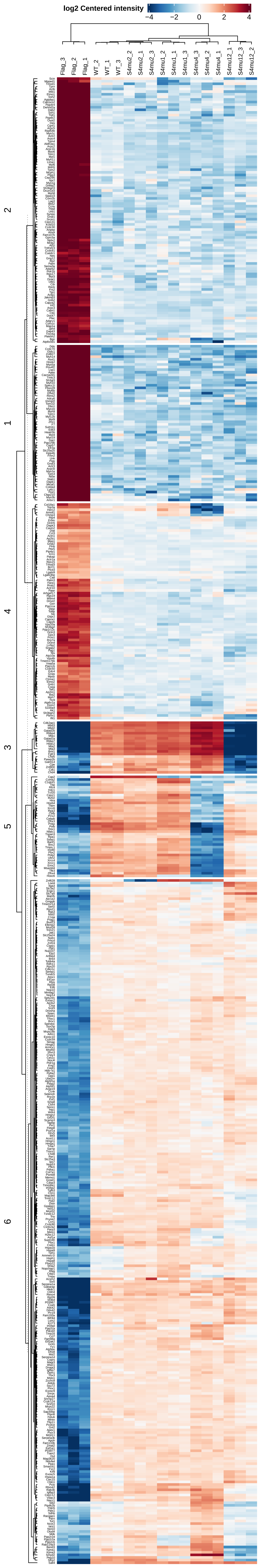
log2 Centered intensity
−4
−2
0
2
4
Flag_3
Flag_2
Flag_1
WT_2
WT_1
WT_3
S4mu2_2
S4mu2_1
S4mu2_3
S4mu1_2
S4mu1_1
S4mu1_3
S4mu4_3
S4mu4_2
S4mu4_1
S4mu12_1
S4mu12_3
S4mu12_2
2
1
4
3
5
6
