# Supplementary material for: Reconstitution of pluripotency from mouse fibroblast through Sall4 overexpression
Source: Nat Commun. 2024 Dec 30;15:10787. doi: 10.1038/s41467-024-54924-5 (PMC11686038; doi:10.1038/s41467-024-54924-5)
Supplement: Supplementary file 4 — Source Data [file 41467_2024_54924_MOESM4_ESM.zip › source data/Supplementary figures/Supplementary figure6/b/CO_OC_peaks_feature.pdf]

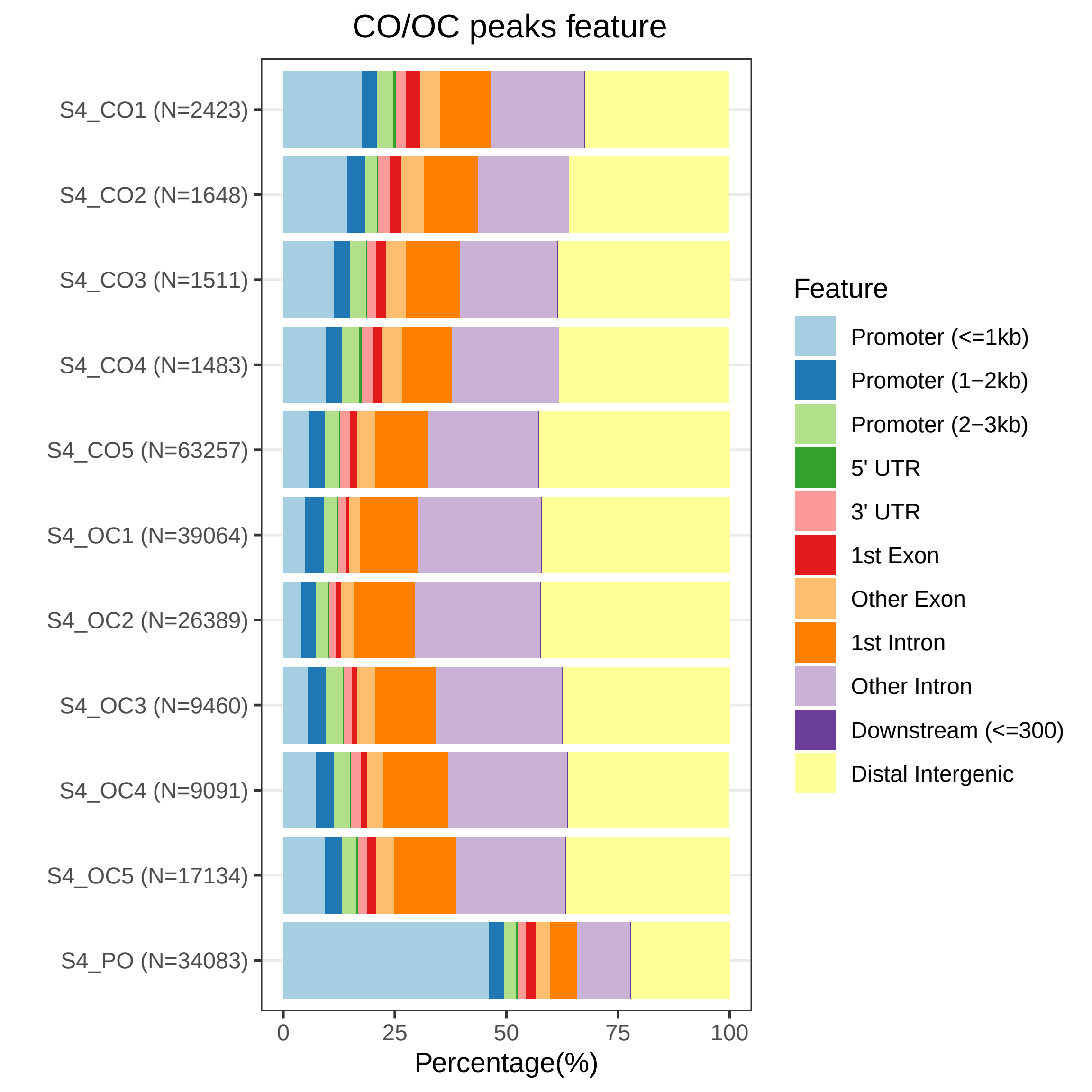

CO/OC peaks feature
S4_CO1 (N=2423)
S4_CO2 (N=1648)
S4_CO3 (N=1511)
S4_CO4 (N=1483)
S4_CO5 (N=63257)
S4_OC1 (N=39064)
S4_OC2 (N=26389)
S4_OC3 (N=9460)
S4_OC4 (N=9091)
S4_OC5 (N=17134)
S4_PO (N=34083)
0
25
50
75
100
Percentage(%)
Feature
Promoter (<=1kb)
Promoter (1−2kb)
Promoter (2−3kb)
5' UTR
3' UTR
1st Exon
Other Exon
1st Intron
Other Intron
Downstream (<=300)
Distal Intergenic
