# Supplementary material for: Reconstitution of pluripotency from mouse fibroblast through Sall4 overexpression
Source: Nat Commun. 2024 Dec 30;15:10787. doi: 10.1038/s41467-024-54924-5 (PMC11686038; doi:10.1038/s41467-024-54924-5)
Supplement: Supplementary file 4 — Source Data [file 41467_2024_54924_MOESM4_ESM.zip › source data/Supplementary figures/Supplementary figure6/c,d/S4_CO1_res_res/TSS_GObp_res.pdf]

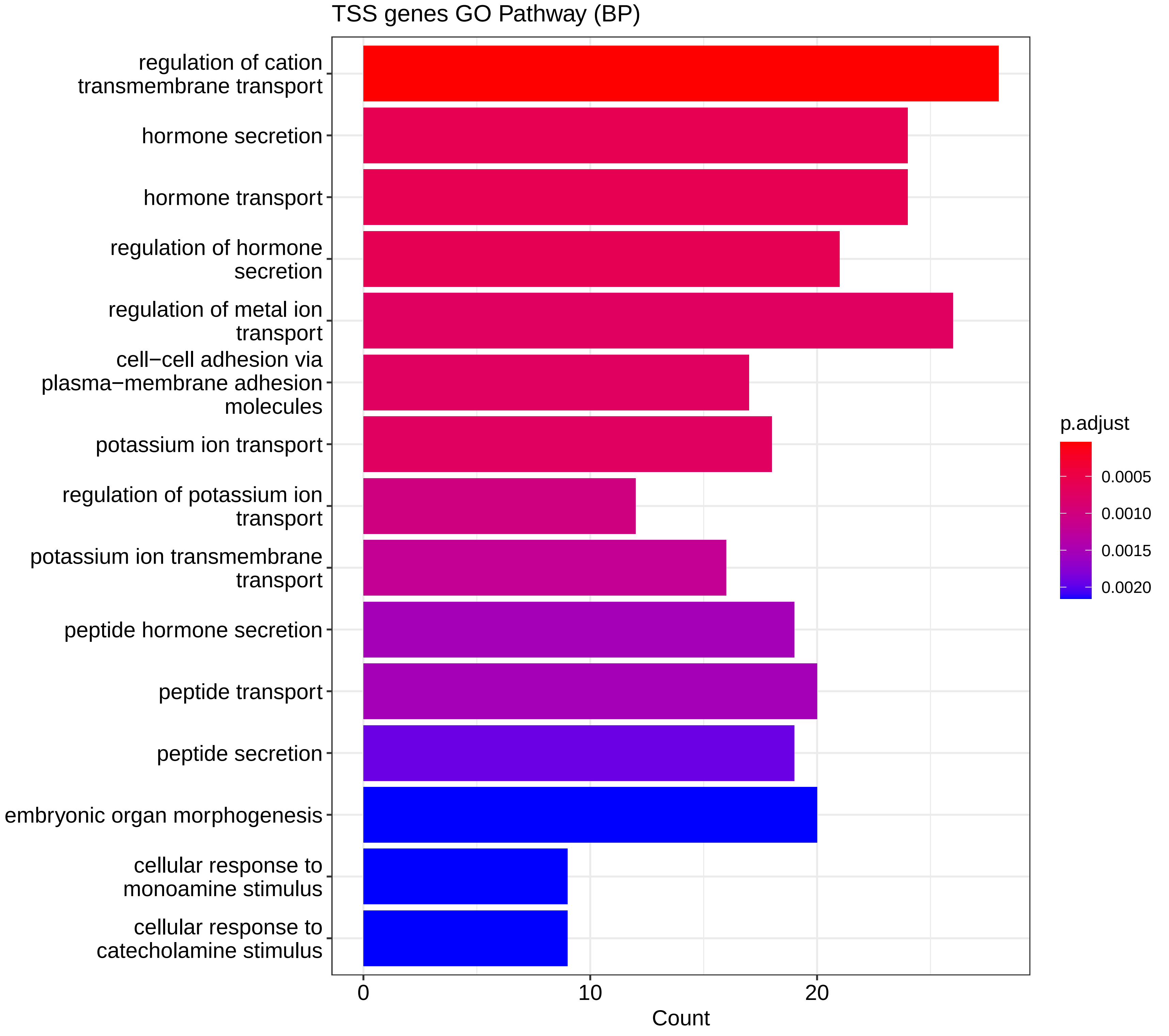
TSS genes GO Pathway (BP)
regulation of cation transmembrane transport
hormone secretion
hormone transport
regulation of hormone secretion
regulation of metal ion transport
cell−cell adhesion via plasma−membrane adhesion molecules
potassium ion transport
regulation of potassium ion transport
potassium ion transmembrane transport
peptide hormone secretion
peptide transport
peptide secretion
embryonic organ morphogenesis
cellular response to monoamine stimulus
cellular response to catecholamine stimulus
0
10
20
Count
p.adjust
0.0005
0.0010
0.0015
0.0020
