# Supplementary material for: Reconstitution of pluripotency from mouse fibroblast through Sall4 overexpression
Source: Nat Commun. 2024 Dec 30;15:10787. doi: 10.1038/s41467-024-54924-5 (PMC11686038; doi:10.1038/s41467-024-54924-5)
Supplement: Supplementary file 4 — Source Data [file 41467_2024_54924_MOESM4_ESM.zip › source data/Supplementary figures/Supplementary figure6/c,d/S4_CO2_res_res/TSS_GObp_res.pdf]

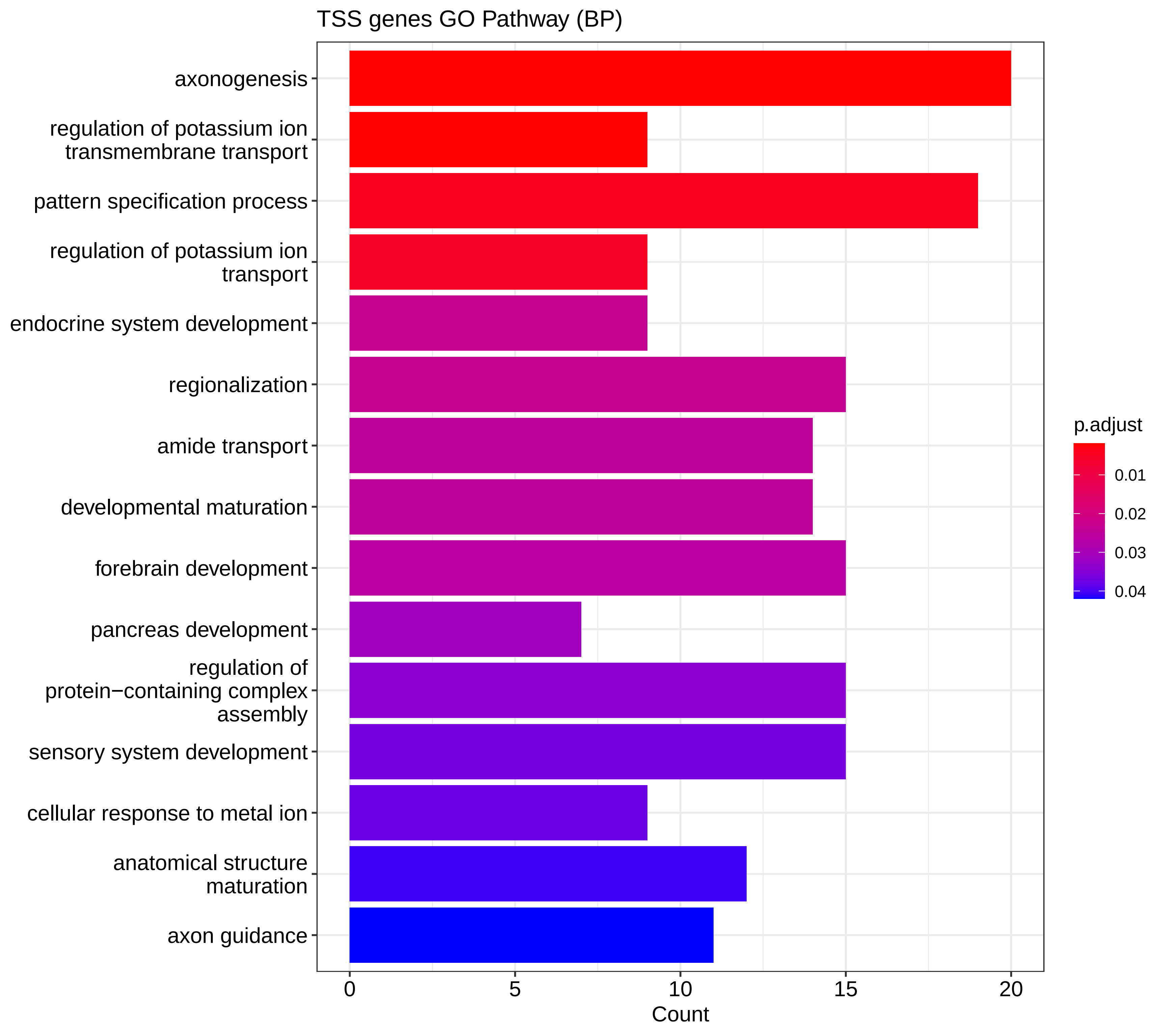
TSS genes GO Pathway (BP)
axonogenesis
regulation of potassium ion transmembrane transport
pattern specification process
regulation of potassium ion transport
endocrine system development
regionalization
amide transport
developmental maturation
forebrain development
pancreas development
regulation of protein−containing complex assembly
sensory system development
cellular response to metal ion
anatomical structure maturation
axon guidance
0
5
10
15
20
Count
p.adjust
0.01
0.02
0.03
0.04
