# Supplementary material for: Reconstitution of pluripotency from mouse fibroblast through Sall4 overexpression
Source: Nat Commun. 2024 Dec 30;15:10787. doi: 10.1038/s41467-024-54924-5 (PMC11686038; doi:10.1038/s41467-024-54924-5)
Supplement: Supplementary file 4 — Source Data [file 41467_2024_54924_MOESM4_ESM.zip › source data/Supplementary figures/Supplementary figure6/c,d/S4_CO3_res_res/all_peaks_annopie.pdf]

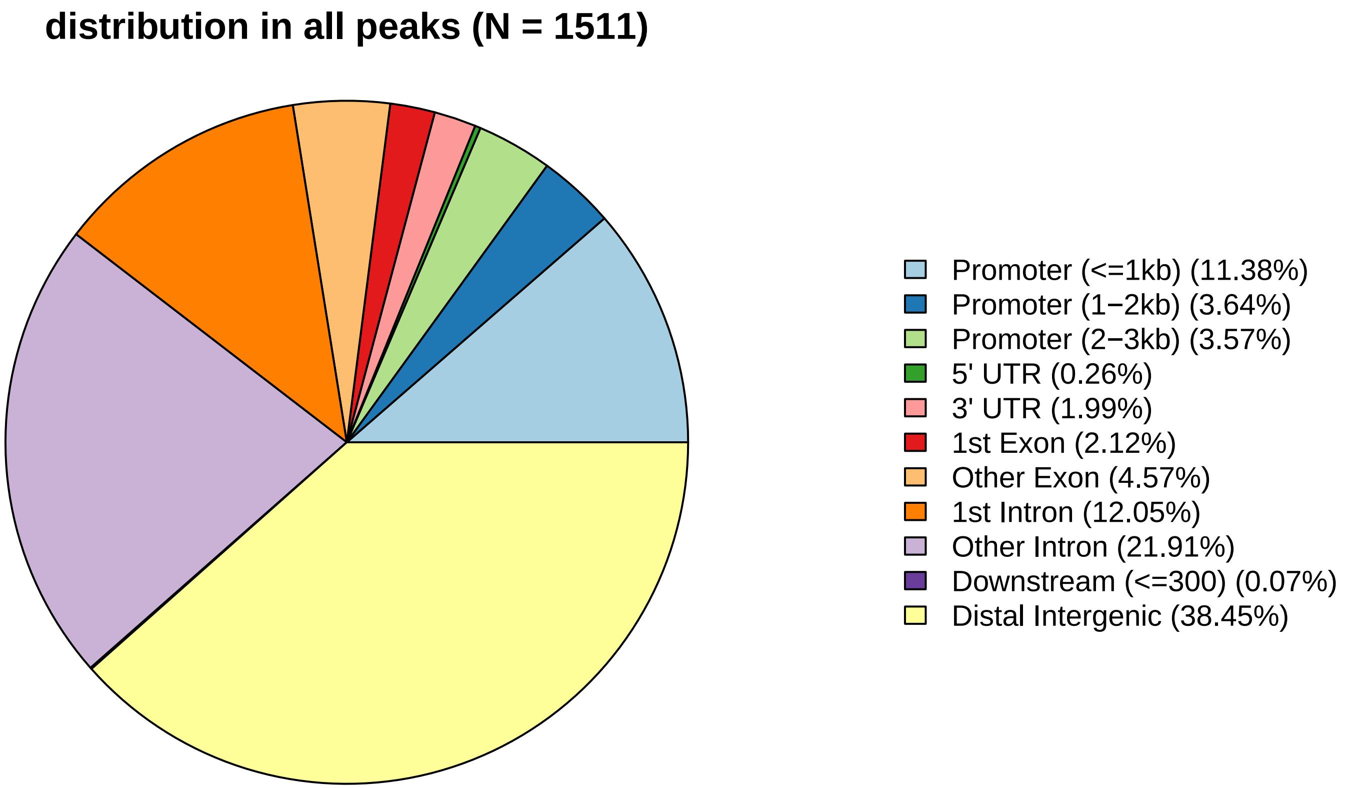
distribution in all peaks (N = 1511)
Promoter (<=1kb) (11.38%)
Promoter (1−2kb) (3.64%)
Promoter (2−3kb) (3.57%)
5' UTR (0.26%)
3' UTR (1.99%)
1st Exon (2.12%)
Other Exon (4.57%)
1st Intron (12.05%)
Other Intron (21.91%)
Downstream (<=300) (0.07%)
Distal Intergenic (38.45%)
