# Supplementary material for: Reconstitution of pluripotency from mouse fibroblast through Sall4 overexpression
Source: Nat Commun. 2024 Dec 30;15:10787. doi: 10.1038/s41467-024-54924-5 (PMC11686038; doi:10.1038/s41467-024-54924-5)
Supplement: Supplementary file 4 — Source Data [file 41467_2024_54924_MOESM4_ESM.zip › source data/Supplementary figures/Supplementary figure6/c,d/S4_CO3_res_res/TSS_GObp_res.pdf]

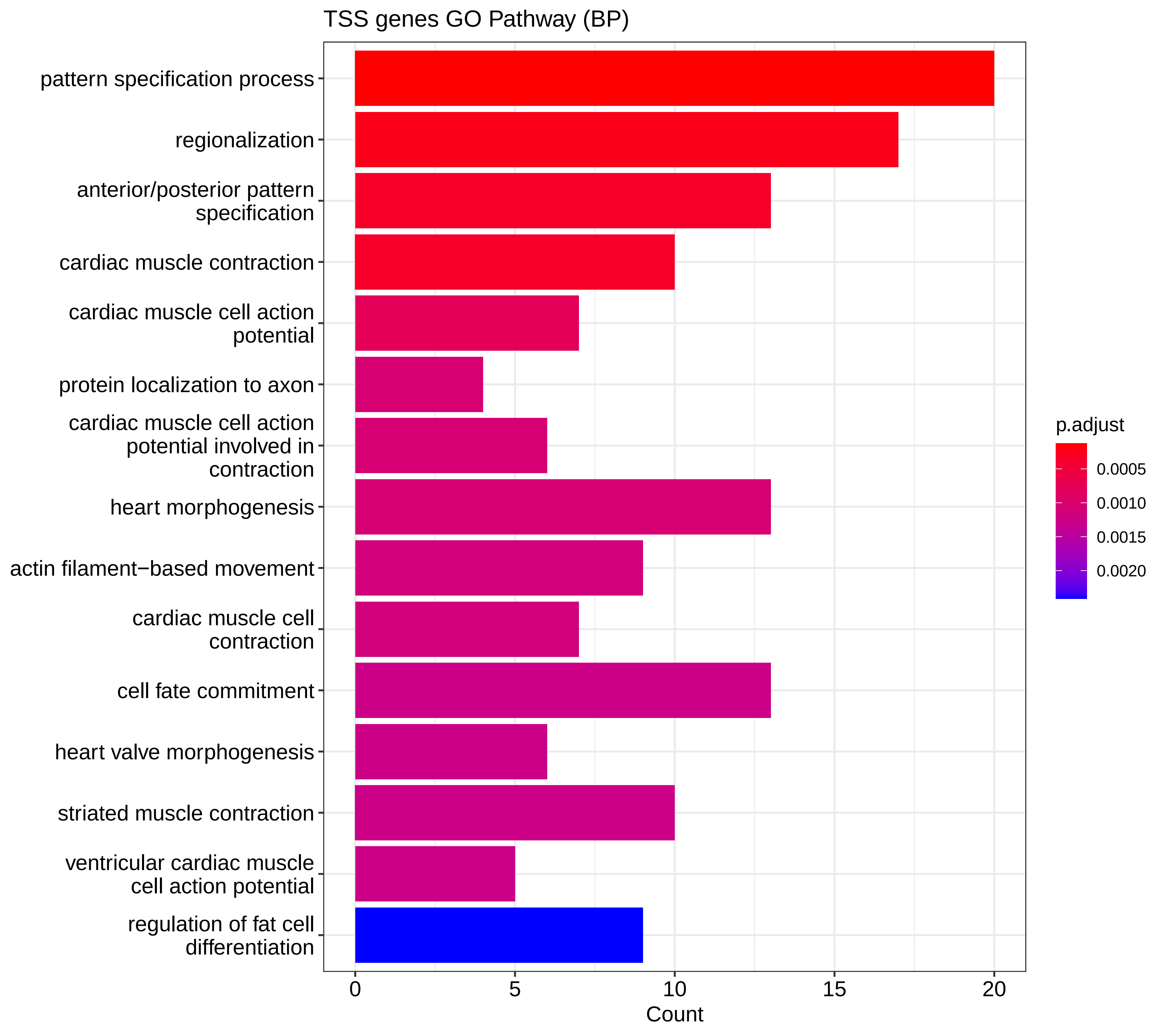

TSS genes GO Pathway (BP)
pattern specification process
regionalization
anterior/posterior pattern specification
cardiac muscle contraction
cardiac muscle cell action potential
protein localization to axon
cardiac muscle cell action potential involved in contraction
heart morphogenesis
actin filament−based movement
cardiac muscle cell contraction
cell fate commitment
heart valve morphogenesis
striated muscle contraction
ventricular cardiac muscle cell action potential
regulation of fat cell differentiation
0
5
10
15
20
Count
p.adjust
0.0005
0.0010
0.0015
0.0020
