# Supplementary material for: Reconstitution of pluripotency from mouse fibroblast through Sall4 overexpression
Source: Nat Commun. 2024 Dec 30;15:10787. doi: 10.1038/s41467-024-54924-5 (PMC11686038; doi:10.1038/s41467-024-54924-5)
Supplement: Supplementary file 4 — Source Data [file 41467_2024_54924_MOESM4_ESM.zip › source data/Supplementary figures/Supplementary figure6/c,d/S4_CO4_res_res/all_peaks_annopie.pdf]

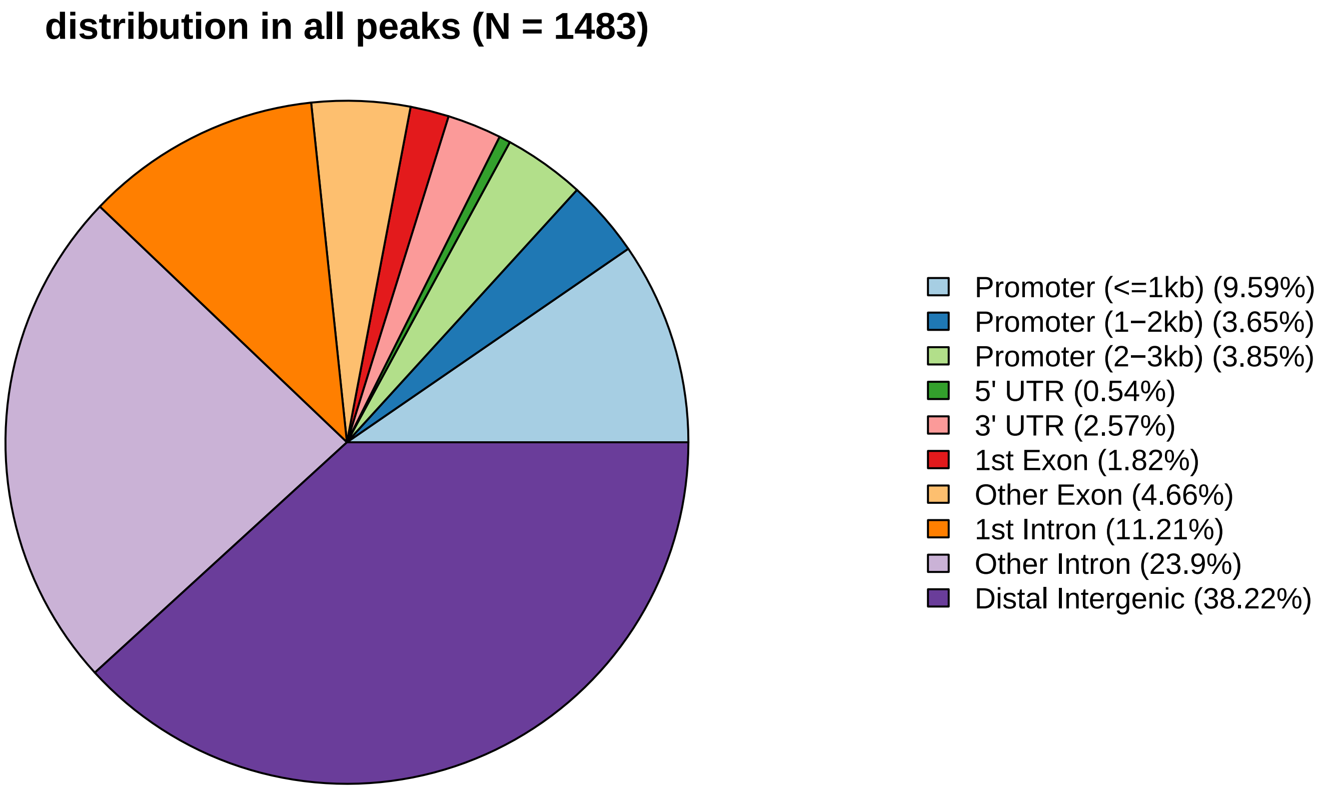
distribution in all peaks (N = 1483)
Promoter (<=1kb) (9.59%)
Promoter (1−2kb) (3.65%)
Promoter (2−3kb) (3.85%)
5' UTR (0.54%)
3' UTR (2.57%)
1st Exon (1.82%)
Other Exon (4.66%)
1st Intron (11.21%)
Other Intron (23.9%)
Distal Intergenic (38.22%)
