# Supplementary material for: Reconstitution of pluripotency from mouse fibroblast through Sall4 overexpression
Source: Nat Commun. 2024 Dec 30;15:10787. doi: 10.1038/s41467-024-54924-5 (PMC11686038; doi:10.1038/s41467-024-54924-5)
Supplement: Supplementary file 4 — Source Data [file 41467_2024_54924_MOESM4_ESM.zip › source data/Supplementary figures/Supplementary figure6/c,d/S4_CO4_res_res/TSS_GObp_res.pdf]

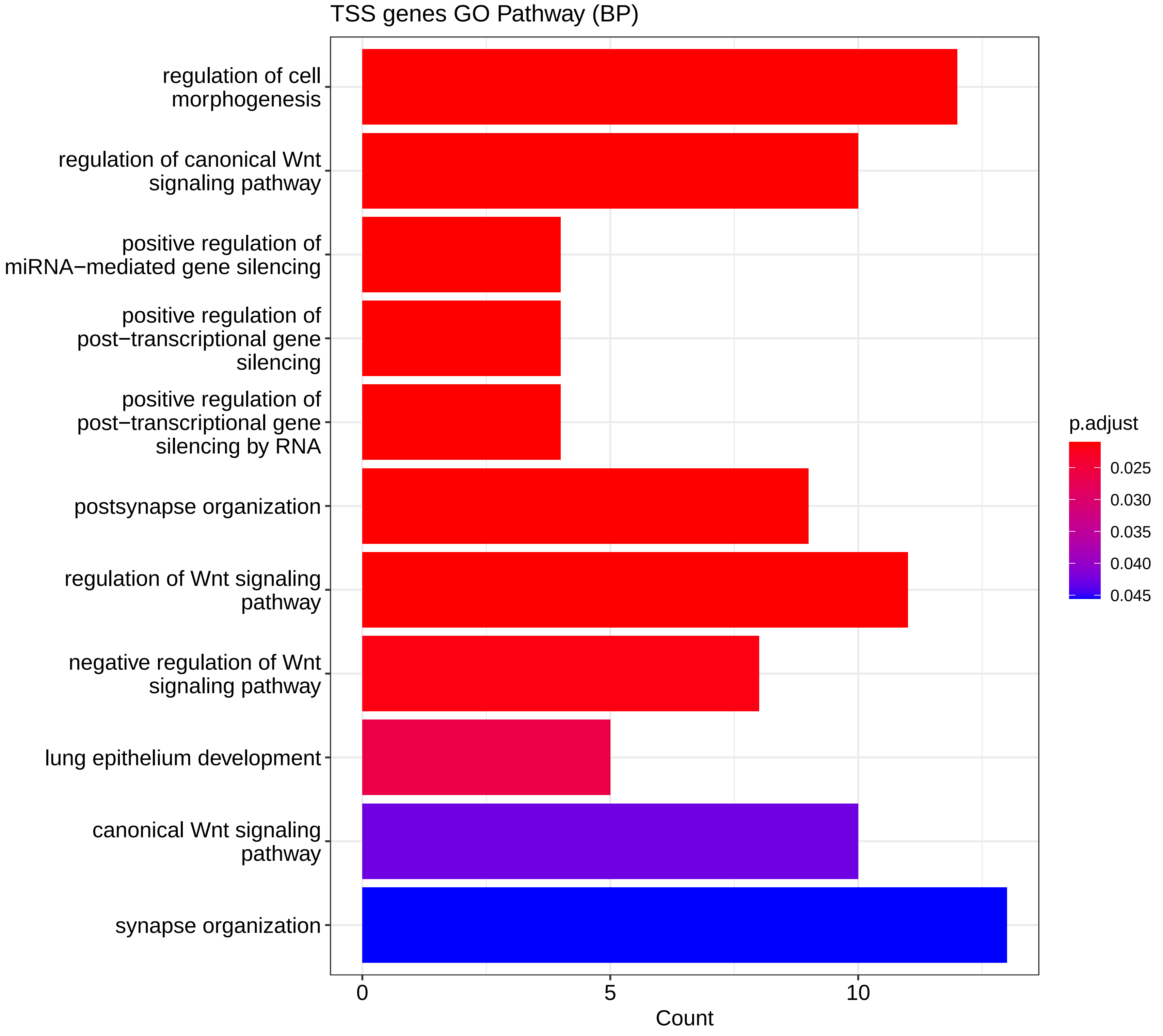

TSS genes GO Pathway (BP)
regulation of cell morphogenesis
regulation of canonical Wnt signaling pathway
positive regulation of miRNA−mediated gene silencing
positive regulation of post−transcriptional gene silencing
positive regulation of post−transcriptional gene silencing by RNA
postsynapse organization
regulation of Wnt signaling pathway
negative regulation of Wnt signaling pathway
lung epithelium development
canonical Wnt signaling pathway
synapse organization
0
5
10
Count
p.adjust
0.025
0.030
0.035
0.040
0.045
