# Supplementary material for: Reconstitution of pluripotency from mouse fibroblast through Sall4 overexpression
Source: Nat Commun. 2024 Dec 30;15:10787. doi: 10.1038/s41467-024-54924-5 (PMC11686038; doi:10.1038/s41467-024-54924-5)
Supplement: Supplementary file 4 — Source Data [file 41467_2024_54924_MOESM4_ESM.zip › source data/Supplementary figures/Supplementary figure6/c,d/S4_CO5_res_res/all_peaks_annopie.pdf]

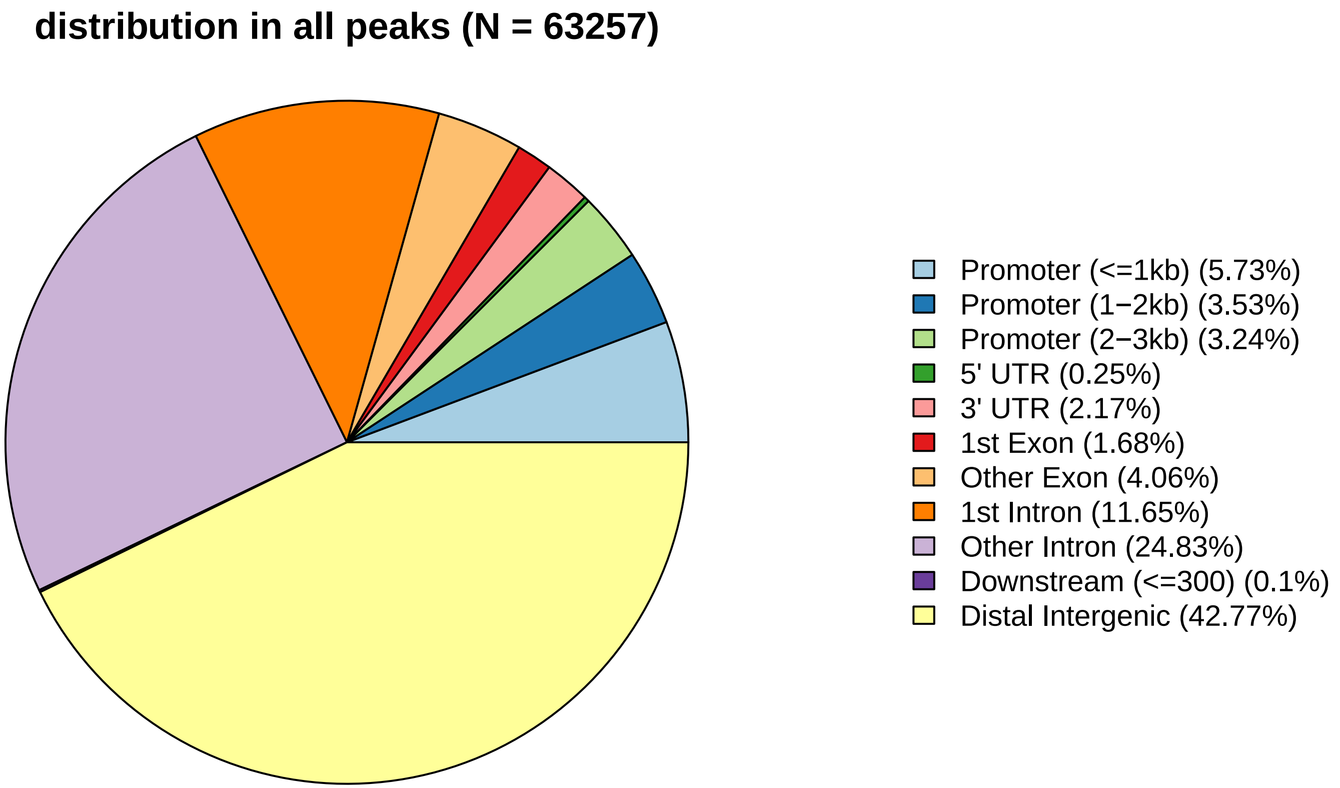
distribution in all peaks (N = 63257)
Promoter (<=1kb) (5.73%)
Promoter (1−2kb) (3.53%)
Promoter (2−3kb) (3.24%)
5' UTR (0.25%)
3' UTR (2.17%)
1st Exon (1.68%)
Other Exon (4.06%)
1st Intron (11.65%)
Other Intron (24.83%)
Downstream (<=300) (0.1%)
Distal Intergenic (42.77%)
