# Supplementary material for: Reconstitution of pluripotency from mouse fibroblast through Sall4 overexpression
Source: Nat Commun. 2024 Dec 30;15:10787. doi: 10.1038/s41467-024-54924-5 (PMC11686038; doi:10.1038/s41467-024-54924-5)
Supplement: Supplementary file 4 — Source Data [file 41467_2024_54924_MOESM4_ESM.zip › source data/Supplementary figures/Supplementary figure6/c,d/S4_CO5_res_res/TSS_GObp_res.pdf]

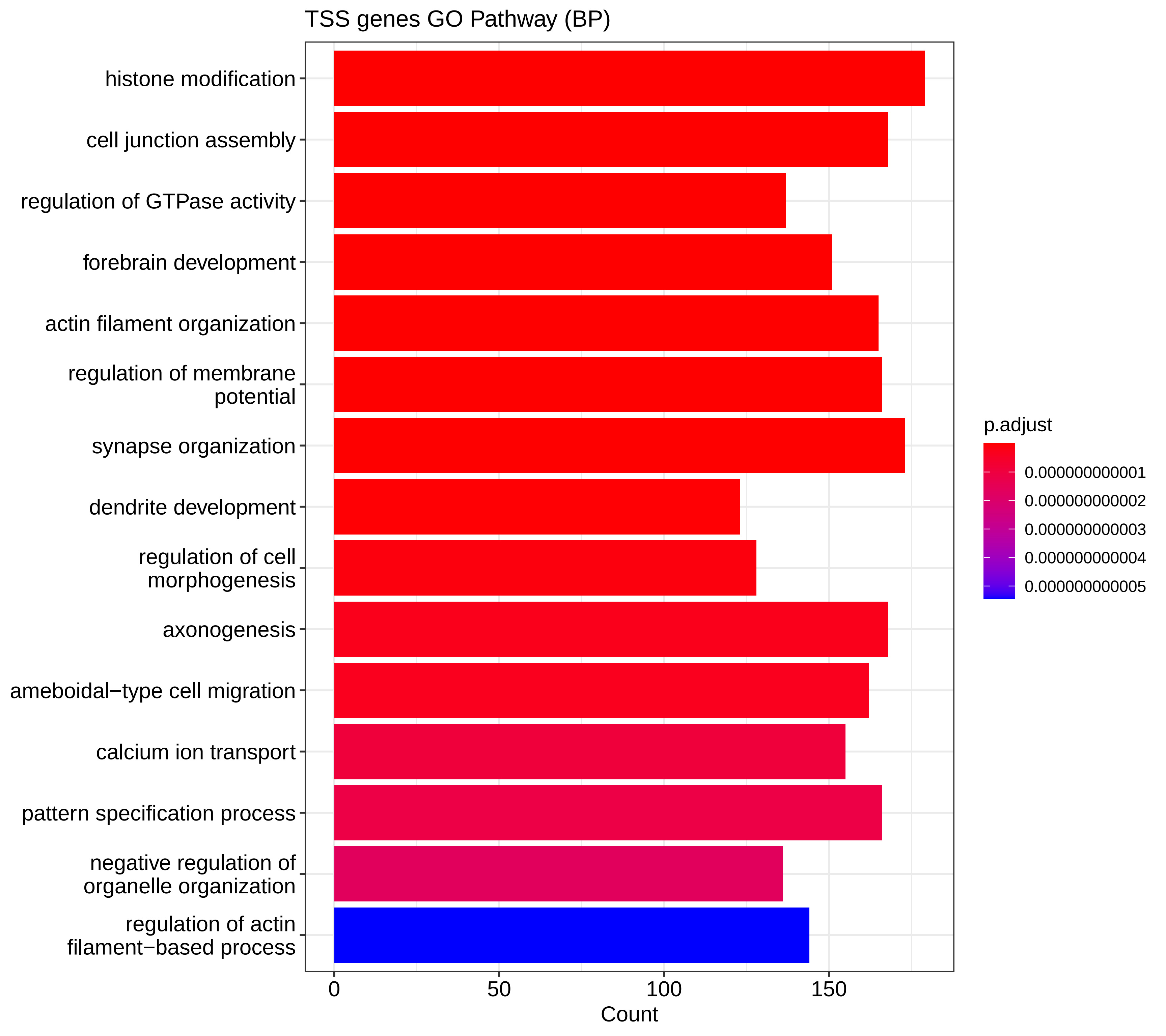

TSS genes GO Pathway (BP)
histone modification
cell junction assembly
regulation of GTPase activity
forebrain development
actin filament organization
regulation of membrane potential
synapse organization
dendrite development
regulation of cell morphogenesis
axonogenesis
ameboidal−type cell migration
calcium ion transport
pattern specification process
negative regulation of organelle organization
regulation of actin filament−based process
0
50
100
150
Count
p.adjust
0.000000000001
0.000000000002
0.000000000003
0.000000000004
0.000000000005
