# Supplementary material for: Reconstitution of pluripotency from mouse fibroblast through Sall4 overexpression
Source: Nat Commun. 2024 Dec 30;15:10787. doi: 10.1038/s41467-024-54924-5 (PMC11686038; doi:10.1038/s41467-024-54924-5)
Supplement: Supplementary file 4 — Source Data [file 41467_2024_54924_MOESM4_ESM.zip › source data/Supplementary figures/Supplementary figure6/c,d/S4_OC1_res_res/TSS_GObp_res.pdf]

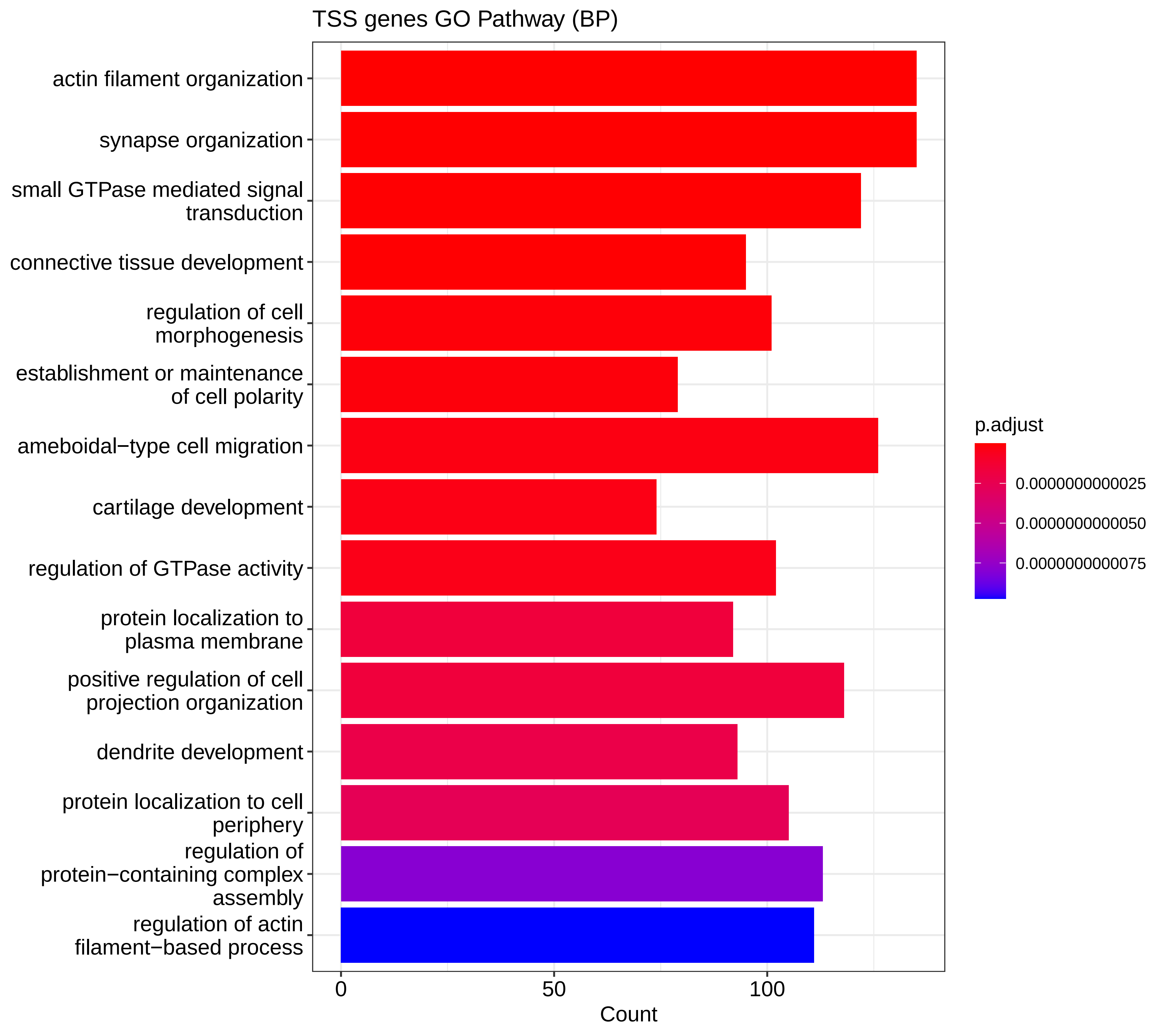

TSS genes GO Pathway (BP)
actin filament organization
synapse organization
small GTPase mediated signal transduction
connective tissue development
regulation of cell morphogenesis
establishment or maintenance of cell polarity
ameboidal−type cell migration
cartilage development
regulation of GTPase activity
protein localization to plasma membrane
positive regulation of cell projection organization
dendrite development
protein localization to cell periphery
regulation of protein−containing complex assembly
regulation of actin filament−based process
0
50
100
Count
p.adjust
0.0000000000025
0.0000000000050
0.0000000000075
