# Supplementary material for: Reconstitution of pluripotency from mouse fibroblast through Sall4 overexpression
Source: Nat Commun. 2024 Dec 30;15:10787. doi: 10.1038/s41467-024-54924-5 (PMC11686038; doi:10.1038/s41467-024-54924-5)
Supplement: Supplementary file 4 — Source Data [file 41467_2024_54924_MOESM4_ESM.zip › source data/Supplementary figures/Supplementary figure6/c,d/S4_OC2_res_res/TSS_GObp_res.pdf]

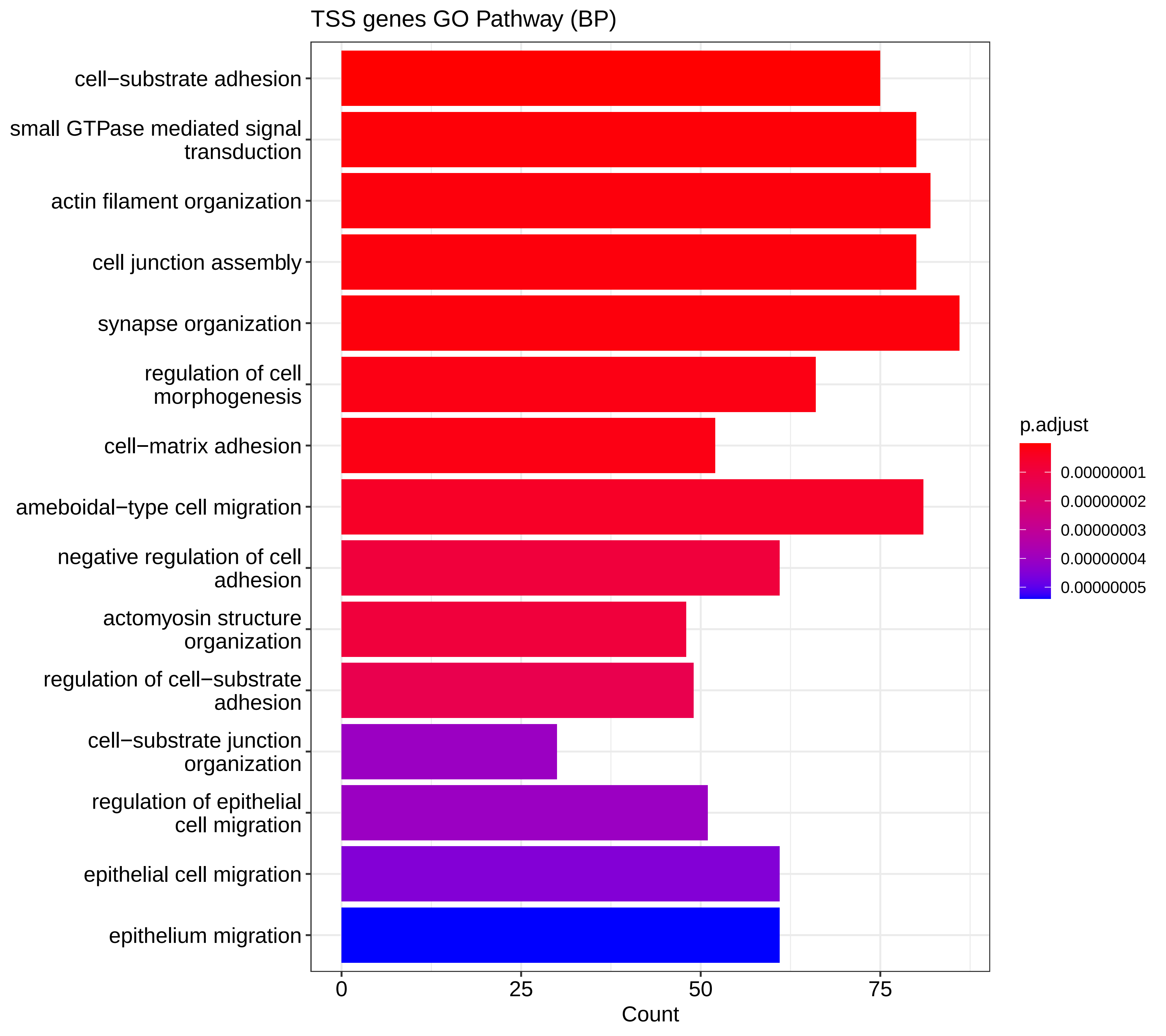

TSS genes GO Pathway (BP)
cell−substrate adhesion
small GTPase mediated signal transduction
actin filament organization
cell junction assembly
synapse organization
regulation of cell morphogenesis
cell−matrix adhesion
ameboidal−type cell migration
negative regulation of cell adhesion
actomyosin structure organization
regulation of cell−substrate adhesion
cell−substrate junction organization
regulation of epithelial cell migration
epithelial cell migration
epithelium migration
0
25
50
75
Count
p.adjust
0.00000001
0.00000002
0.00000003
0.00000004
0.00000005
