# Supplementary material for: Reconstitution of pluripotency from mouse fibroblast through Sall4 overexpression
Source: Nat Commun. 2024 Dec 30;15:10787. doi: 10.1038/s41467-024-54924-5 (PMC11686038; doi:10.1038/s41467-024-54924-5)
Supplement: Supplementary file 4 — Source Data [file 41467_2024_54924_MOESM4_ESM.zip › source data/Supplementary figures/Supplementary figure6/c,d/S4_OC3_res_res/TSS_GObp_res.pdf]

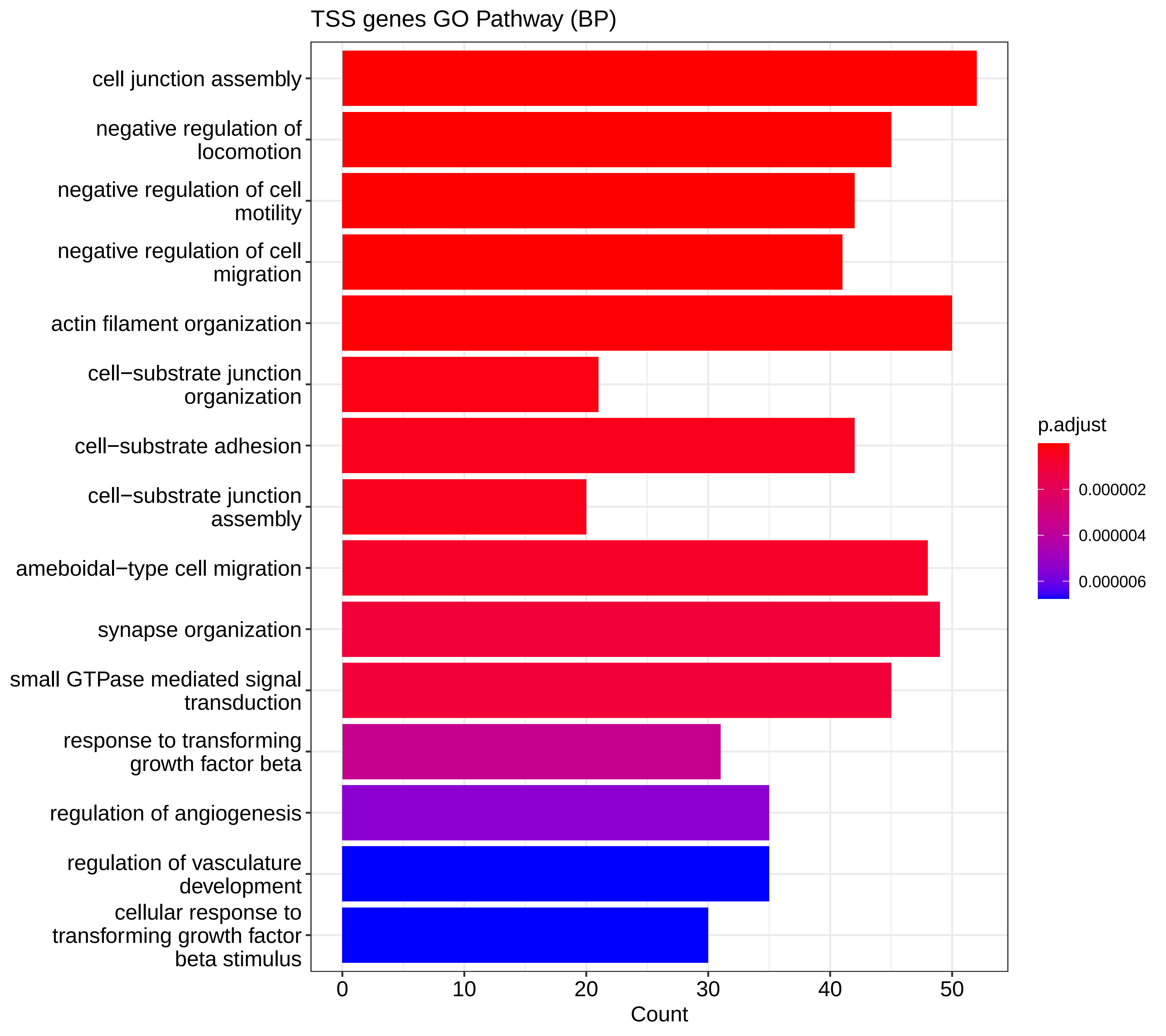

TSS genes GO Pathway (BP)
cell junction assembly
negative regulation of locomotion
negative regulation of cell motility
negative regulation of cell migration
actin filament organization
cell−substrate junction organization
cell−substrate adhesion
cell−substrate junction assembly
ameboidal−type cell migration
synapse organization
small GTPase mediated signal transduction
response to transforming growth factor beta
regulation of angiogenesis
regulation of vasculature development
cellular response to transforming growth factor beta stimulus
0
10
20
30
40
50
Count
p.adjust
0.000002
0.000004
0.000006
