# Supplementary material for: Reconstitution of pluripotency from mouse fibroblast through Sall4 overexpression
Source: Nat Commun. 2024 Dec 30;15:10787. doi: 10.1038/s41467-024-54924-5 (PMC11686038; doi:10.1038/s41467-024-54924-5)
Supplement: Supplementary file 4 — Source Data [file 41467_2024_54924_MOESM4_ESM.zip › source data/Supplementary figures/Supplementary figure6/c,d/S4_OC4_res_res/TSS_GObp_res.pdf]

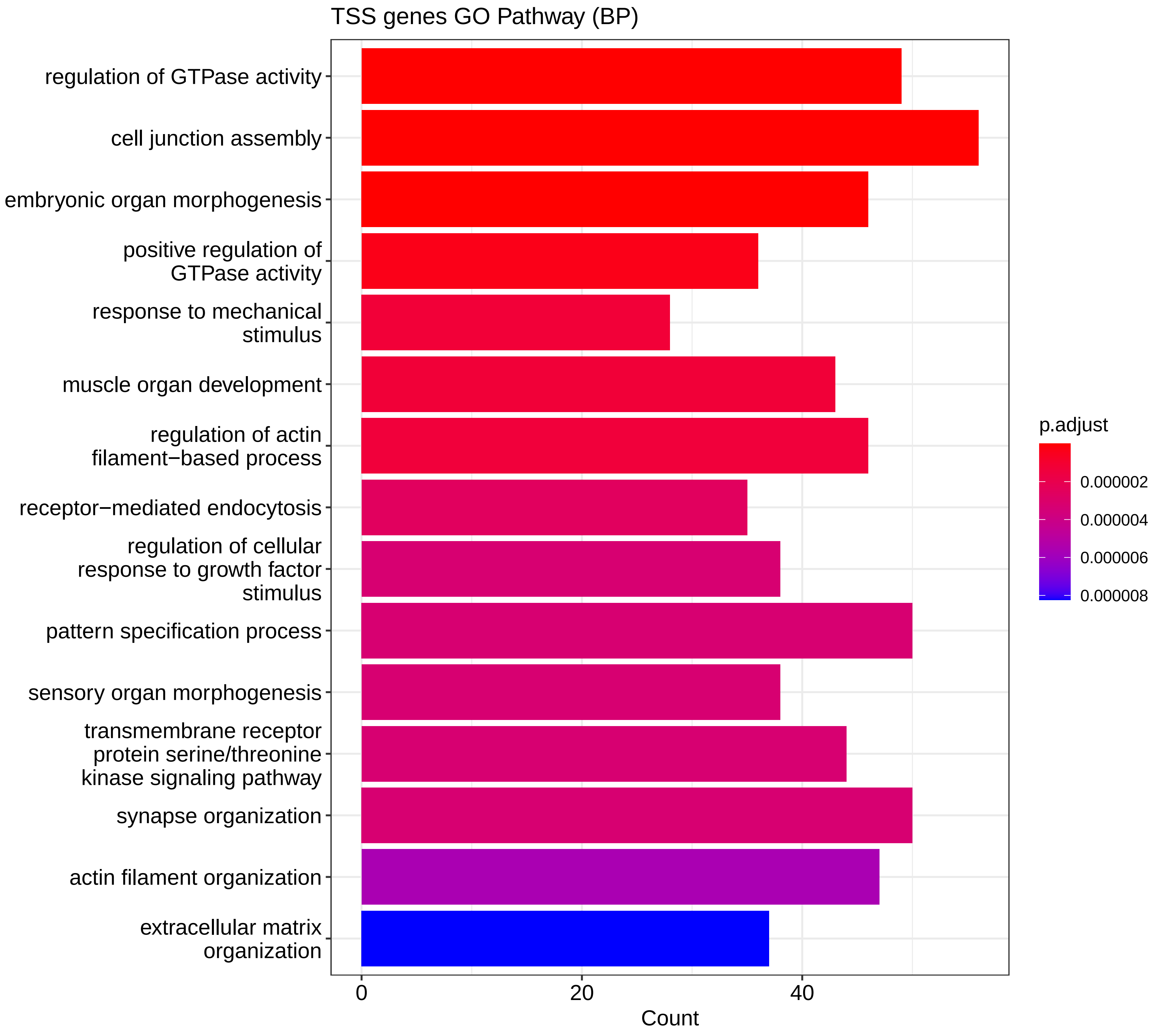
TSS genes GO Pathway (BP)
regulation of GTPase activity
cell junction assembly
embryonic organ morphogenesis
positive regulation of GTPase activity
response to mechanical stimulus
muscle organ development
regulation of actin filament−based process
receptor−mediated endocytosis
regulation of cellular response to growth factor stimulus
pattern specification process
sensory organ morphogenesis
transmembrane receptor protein serine/threonine kinase signaling pathway
synapse organization
actin filament organization
extracellular matrix organization
0
20
40
Count
p.adjust
0.000002
0.000004
0.000006
0.000008
