# Supplementary material for: Reconstitution of pluripotency from mouse fibroblast through Sall4 overexpression
Source: Nat Commun. 2024 Dec 30;15:10787. doi: 10.1038/s41467-024-54924-5 (PMC11686038; doi:10.1038/s41467-024-54924-5)
Supplement: Supplementary file 4 — Source Data [file 41467_2024_54924_MOESM4_ESM.zip › source data/Supplementary figures/Supplementary figure6/c,d/S4_OC5_res_res/all_peaks_annopie.pdf]

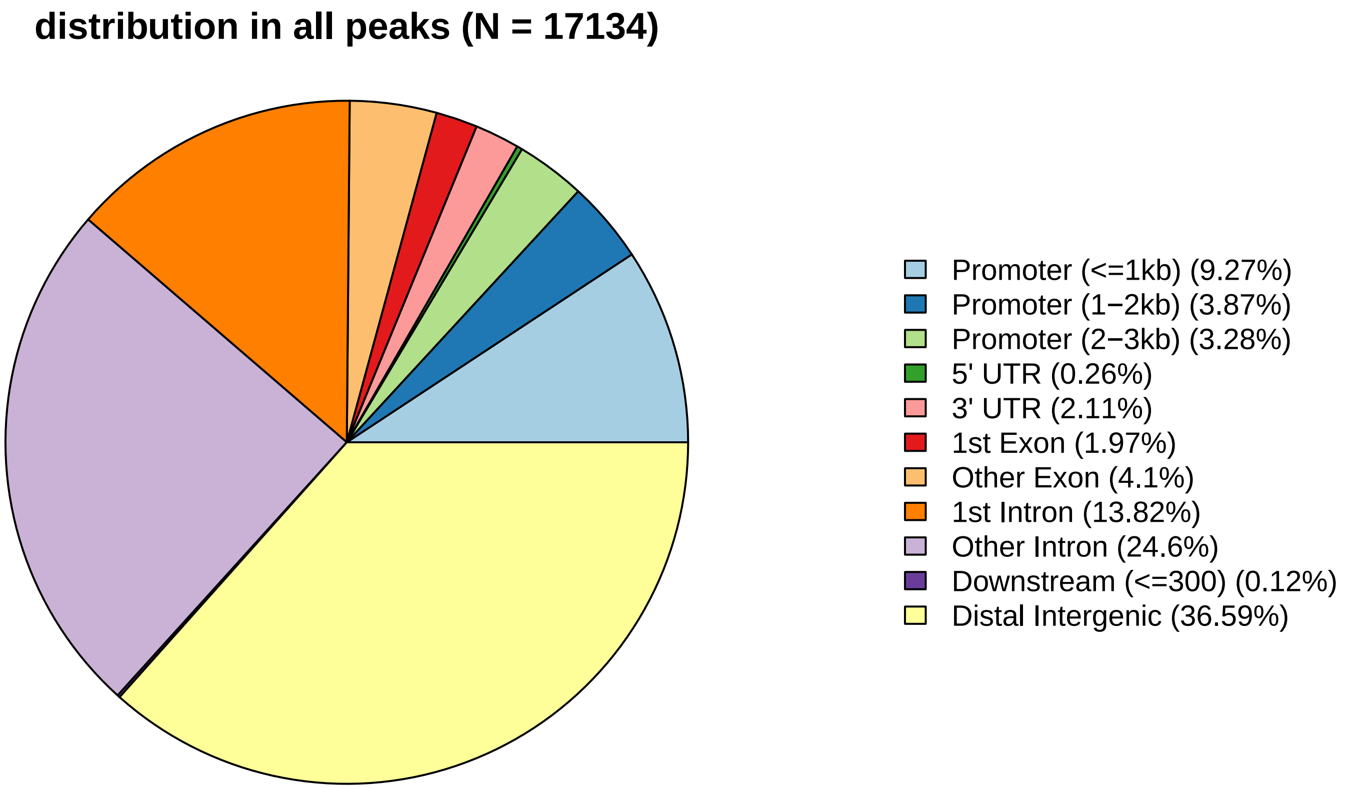
distribution in all peaks (N = 17134)
Promoter (<=1kb) (9.27%)
Promoter (1−2kb) (3.87%)
Promoter (2−3kb) (3.28%)
5' UTR (0.26%)
3' UTR (2.11%)
1st Exon (1.97%)
Other Exon (4.1%)
1st Intron (13.82%)
Other Intron (24.6%)
Downstream (<=300) (0.12%)
Distal Intergenic (36.59%)
