# Supplementary material for: Reconstitution of pluripotency from mouse fibroblast through Sall4 overexpression
Source: Nat Commun. 2024 Dec 30;15:10787. doi: 10.1038/s41467-024-54924-5 (PMC11686038; doi:10.1038/s41467-024-54924-5)
Supplement: Supplementary file 4 — Source Data [file 41467_2024_54924_MOESM4_ESM.zip › source data/Supplementary figures/Supplementary figure6/c,d/S4_OC5_res_res/TSS_GObp_res.pdf]

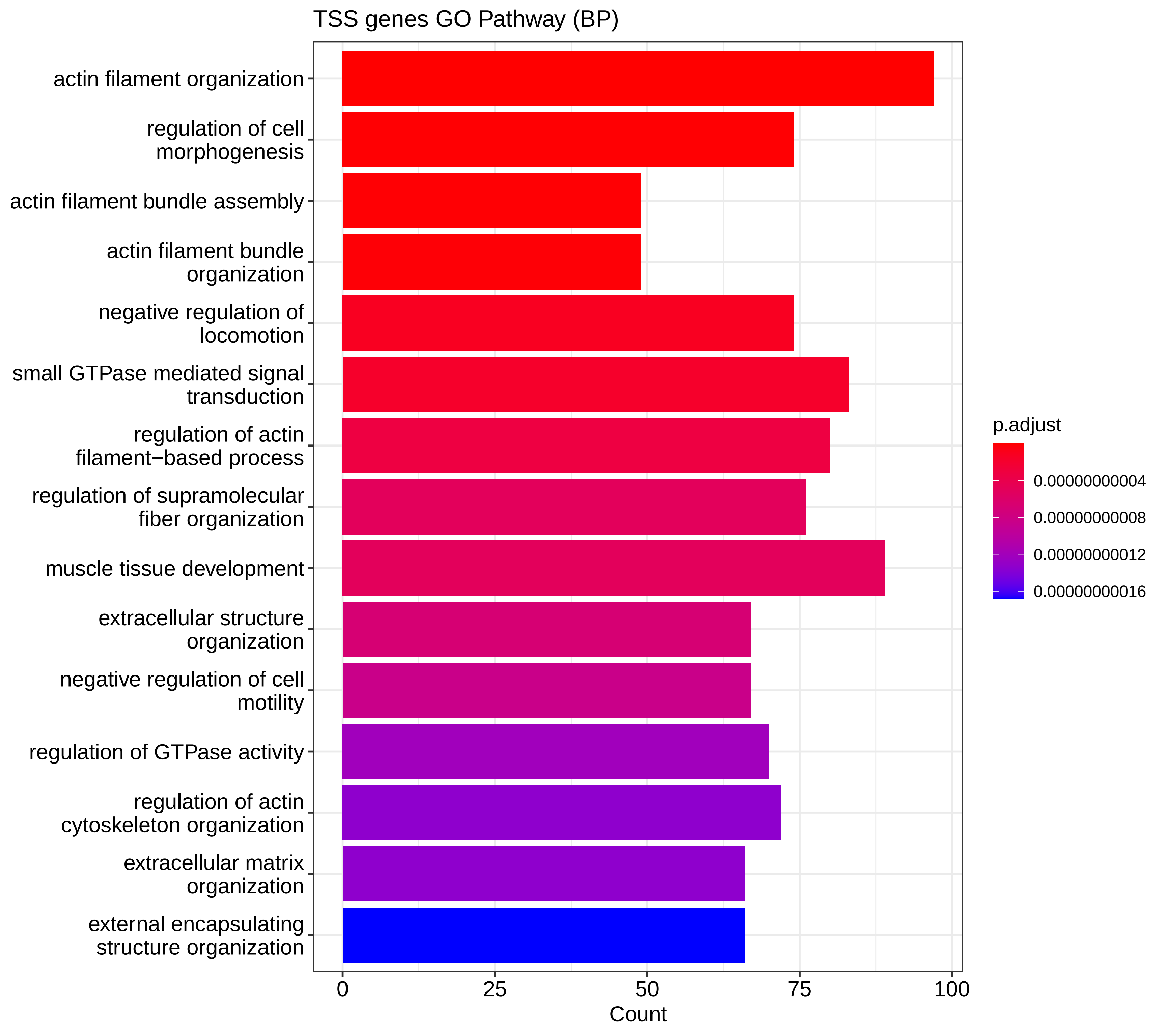

TSS genes GO Pathway (BP)
actin filament organization
regulation of cell morphogenesis
actin filament bundle assembly
actin filament bundle organization
negative regulation of locomotion
small GTPase mediated signal transduction
regulation of actin filament−based process
regulation of supramolecular fiber organization
muscle tissue development
extracellular structure organization
negative regulation of cell motility
regulation of GTPase activity
regulation of actin cytoskeleton organization
extracellular matrix organization
external encapsulating structure organization
0
25
50
75
100
Count
p.adjust
0.00000000004
0.00000000008
0.00000000012
0.00000000016
