# Supplementary material for: Reconstitution of pluripotency from mouse fibroblast through Sall4 overexpression
Source: Nat Commun. 2024 Dec 30;15:10787. doi: 10.1038/s41467-024-54924-5 (PMC11686038; doi:10.1038/s41467-024-54924-5)
Supplement: Supplementary file 4 — Source Data [file 41467_2024_54924_MOESM4_ESM.zip › source data/Supplementary figures/Supplementary figure6/c,d/S4_PO_res_res/TSS_GObp_res.pdf]

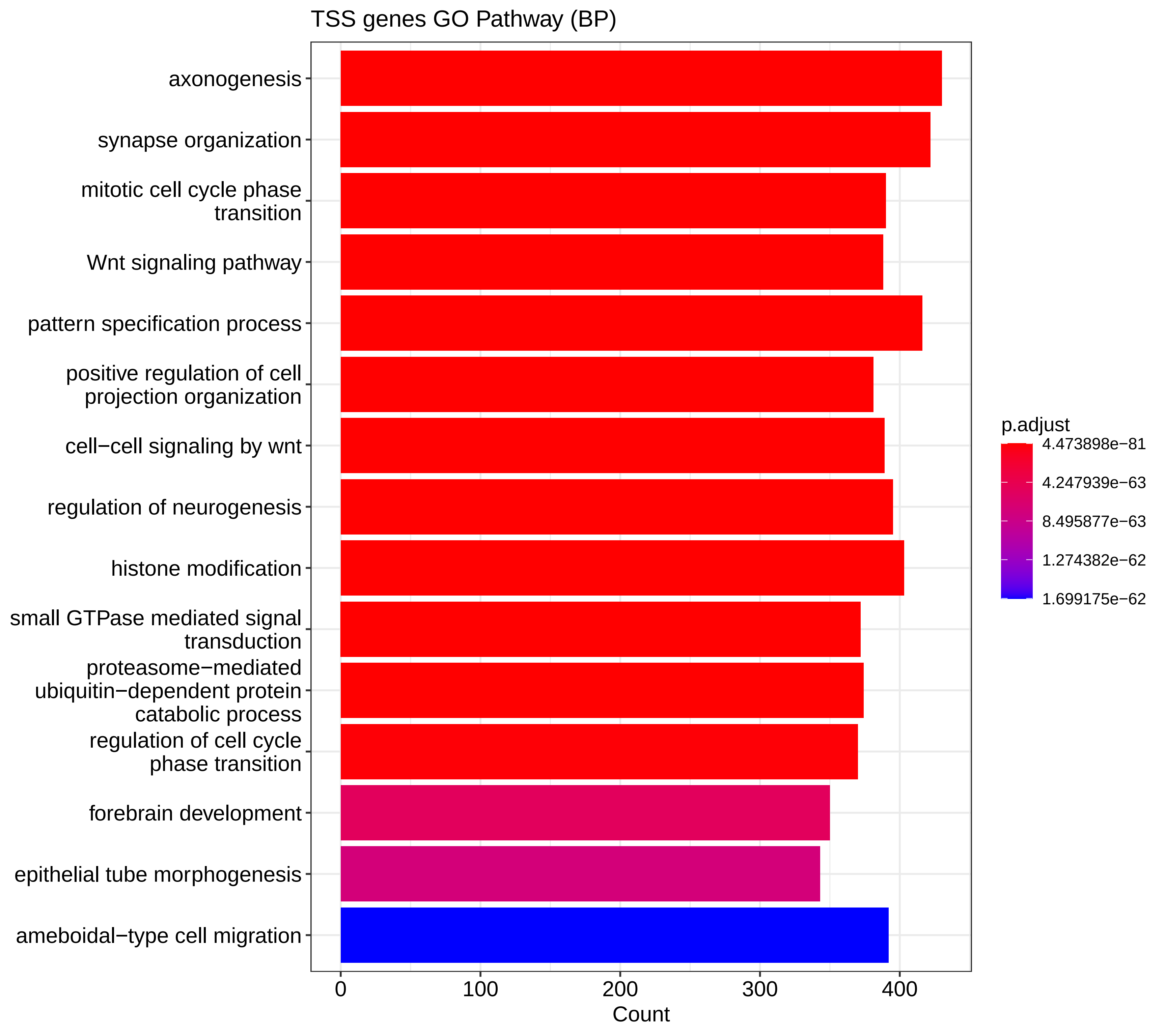
TSS genes GO Pathway (BP)
axonogenesis
synapse organization
mitotic cell cycle phase transition
Wnt signaling pathway
pattern specification process
positive regulation of cell projection organization
cell−cell signaling by wnt
regulation of neurogenesis
histone modification
small GTPase mediated signal transduction
proteasome−mediated ubiquitin−dependent protein catabolic process
regulation of cell cycle phase transition
forebrain development
epithelial tube morphogenesis
ameboidal−type cell migration
0
100
200
300
400
Count
p.adjust
4.473898e−81
4.247939e−63
8.495877e−63
1.274382e−62
1.699175e−62
