# Supplementary material for: Reconstitution of pluripotency from mouse fibroblast through Sall4 overexpression
Source: Nat Commun. 2024 Dec 30;15:10787. doi: 10.1038/s41467-024-54924-5 (PMC11686038; doi:10.1038/s41467-024-54924-5)
Supplement: Supplementary file 4 — Source Data [file 41467_2024_54924_MOESM4_ESM.zip › source data/Supplementary figures/Supplementary figure7/d/direct_effect/all_peaks_annopie.pdf]

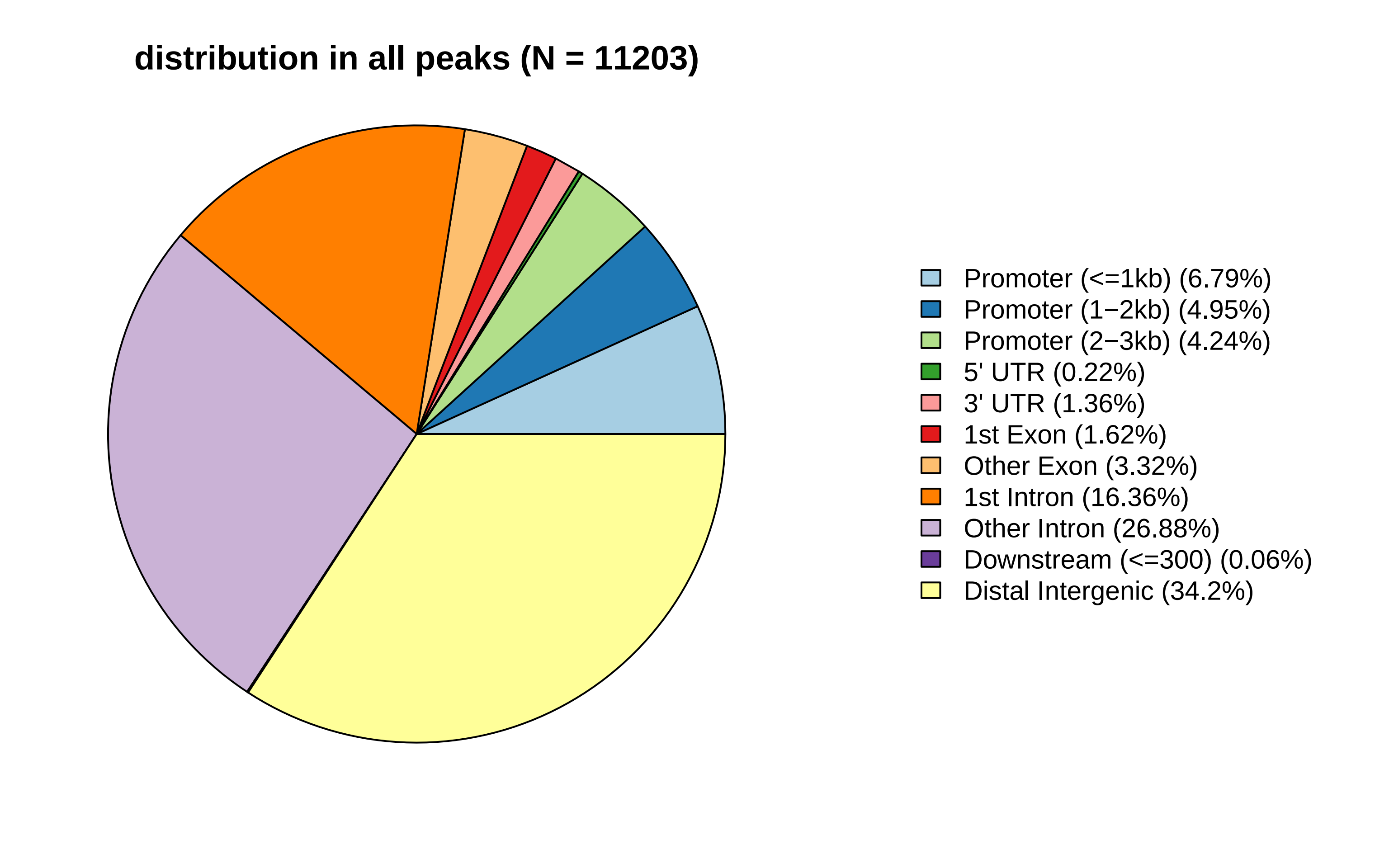
distribution in all peaks (N = 11203)
Promoter (<=1kb) (6.79%)
Promoter (1−2kb) (4.95%)
Promoter (2−3kb) (4.24%)
5' UTR (0.22%)
3' UTR (1.36%)
1st Exon (1.62%)
Other Exon (3.32%)
1st Intron (16.36%)
Other Intron (26.88%)
Downstream (<=300) (0.06%)
Distal Intergenic (34.2%)
