# Supplementary material for: Reconstitution of pluripotency from mouse fibroblast through Sall4 overexpression
Source: Nat Commun. 2024 Dec 30;15:10787. doi: 10.1038/s41467-024-54924-5 (PMC11686038; doi:10.1038/s41467-024-54924-5)
Supplement: Supplementary file 4 — Source Data [file 41467_2024_54924_MOESM4_ESM.zip › source data/Supplementary figures/Supplementary figure7/d/direct_effect/TSS_GObp_res.pdf]

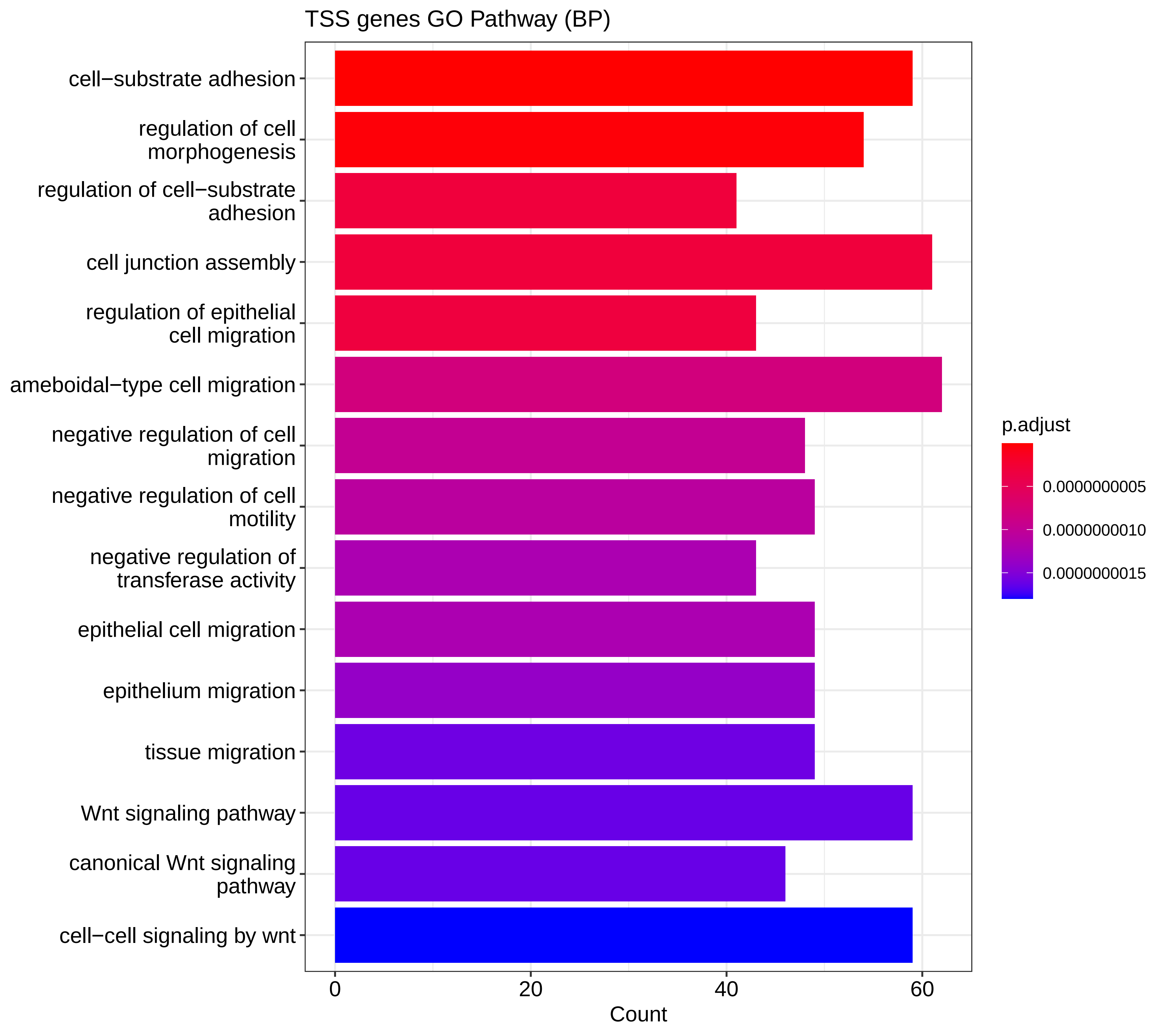
TSS genes GO Pathway (BP)
cell−substrate adhesion
regulation of cell morphogenesis
regulation of cell−substrate adhesion
cell junction assembly
regulation of epithelial cell migration
ameboidal−type cell migration
negative regulation of cell migration
negative regulation of cell motility
negative regulation of transferase activity
epithelial cell migration
epithelium migration
tissue migration
Wnt signaling pathway
canonical Wnt signaling pathway
cell−cell signaling by wnt
0
20
40
60
Count
p.adjust
0.0000000005
0.0000000010
0.0000000015
