# Supplementary material for: Reconstitution of pluripotency from mouse fibroblast through Sall4 overexpression
Source: Nat Commun. 2024 Dec 30;15:10787. doi: 10.1038/s41467-024-54924-5 (PMC11686038; doi:10.1038/s41467-024-54924-5)
Supplement: Supplementary file 4 — Source Data [file 41467_2024_54924_MOESM4_ESM.zip › source data/Supplementary figures/Supplementary figure7/d/SALL4 indirect effect genes GO/all_peaks_annopie.pdf]

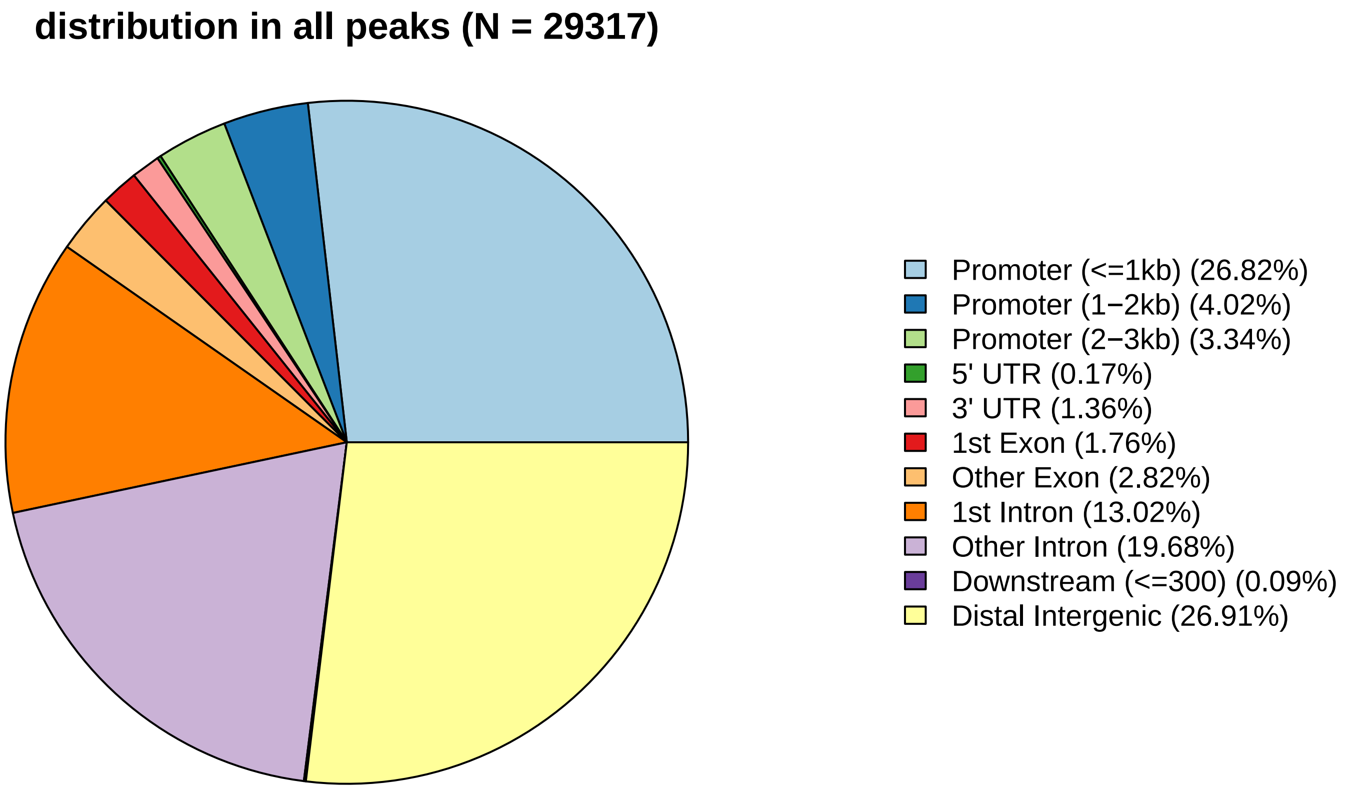
distribution in all peaks (N = 29317)
Promoter (<=1kb) (26.82%)
Promoter (1−2kb) (4.02%)
Promoter (2−3kb) (3.34%)
5' UTR (0.17%)
3' UTR (1.36%)
1st Exon (1.76%)
Other Exon (2.82%)
1st Intron (13.02%)
Other Intron (19.68%)
Downstream (<=300) (0.09%)
Distal Intergenic (26.91%)
