# Supplementary material for: Reconstitution of pluripotency from mouse fibroblast through Sall4 overexpression
Source: Nat Commun. 2024 Dec 30;15:10787. doi: 10.1038/s41467-024-54924-5 (PMC11686038; doi:10.1038/s41467-024-54924-5)
Supplement: Supplementary file 4 — Source Data [file 41467_2024_54924_MOESM4_ESM.zip › source data/Supplementary figures/Supplementary figure7/d/SALL4 indirect effect genes GO/TSS_GObp_res.pdf]

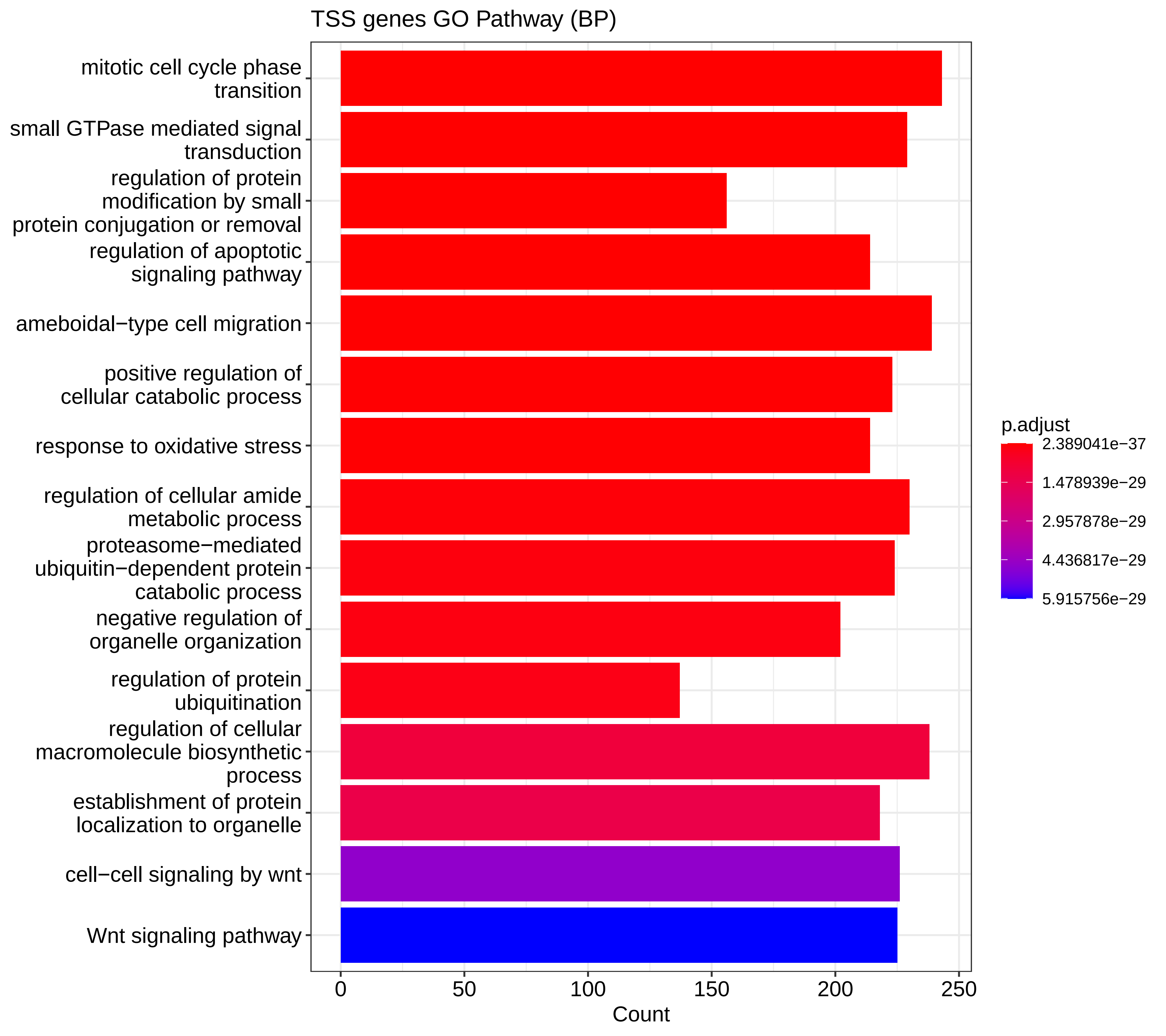
TSS genes GO Pathway (BP)
mitotic cell cycle phase transition
small GTPase mediated signal transduction
regulation of protein modification by small protein conjugation or removal
regulation of apoptotic signaling pathway
ameboidal−type cell migration
positive regulation of cellular catabolic process
response to oxidative stress
regulation of cellular amide metabolic process
proteasome−mediated ubiquitin−dependent protein catabolic process
negative regulation of organelle organization
regulation of protein ubiquitination
regulation of cellular macromolecule biosynthetic process
establishment of protein localization to organelle
cell−cell signaling by wnt
Wnt signaling pathway
0
50
100
150
200
250
Count
p.adjust
2.389041e−37
1.478939e−29
2.957878e−29
4.436817e−29
5.915756e−29
