# Supplementary material for: Reconstitution of pluripotency from mouse fibroblast through Sall4 overexpression
Source: Nat Commun. 2024 Dec 30;15:10787. doi: 10.1038/s41467-024-54924-5 (PMC11686038; doi:10.1038/s41467-024-54924-5)

Figure 1j

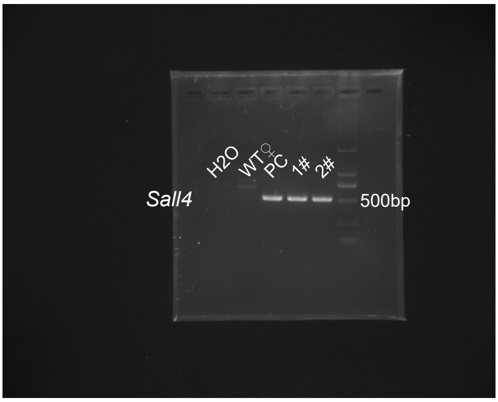

Supplementary 2 b

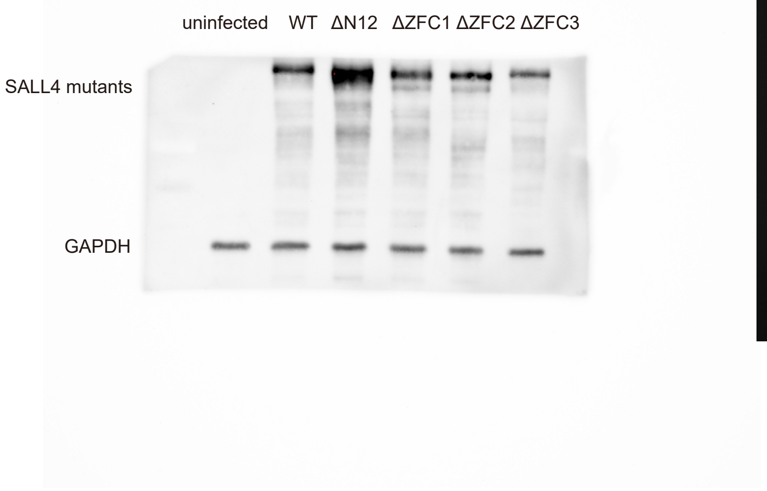

Supplementary 9 f

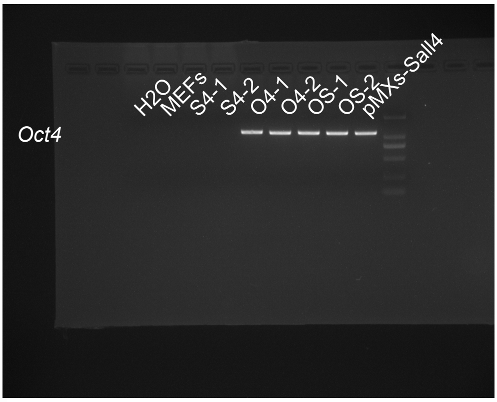

Supplementary 7 f

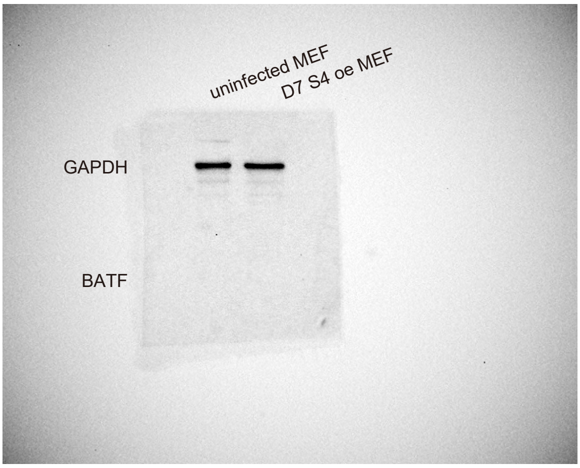

Supplementary 9 f

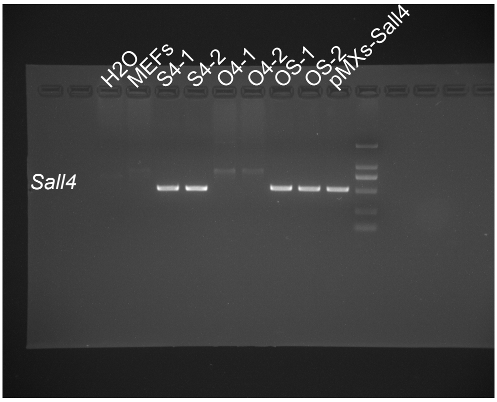

Supplement: Supplementary file 4 — Source Data [file 41467_2024_54924_MOESM4_ESM.zip › source data/uncropped western blot and gels.pdf]
